# Aluminum-catalyzed tunable halodefluorination of trifluoromethyl- and difluoroalkyl-substituted olefins†

**DOI:** 10.1039/d0sc03883k

**Published:** 2020-10-01

**Authors:** Zhong Liu, Xian-Shuang Tu, Le-Tao Guo, Xiao-Chen Wang

**Affiliations:** State Key Laboratory and Institute of Elemento-Organic Chemistry, College of Chemistry, Nankai University 94 Weijin Road Tianjin 300071 China xcwang@nankai.edu.cn

## Abstract

Herein, we report unprecedented aluminum-catalyzed halodefluorination reactions of trifluoromethyl- and difluoroalkyl-substituted olefins with bromo- or chlorotrimethylsilane. The interesting feature of these reactions is that one, two, or three fluorine atoms can be selectively replaced with bromine or chlorine atoms by modification of the reaction conditions. The generated products can undergo a variety of subsequent transformations, thus constituting a valuable stock of building blocks for installing fluorine-containing olefin motifs in other molecules.

## Abstract 2

Aluminum-catalyzed halodefluorination reactions of fluoroalkyl-substituted olefins are developed. The reactions can selectively deliver mono-, di-, or trisubstituted products.

## Graphical abstract

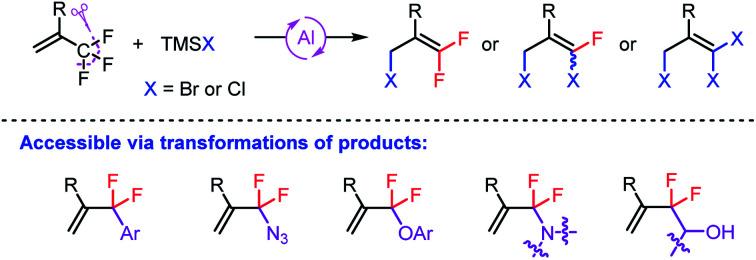

Combined with the use of fluorine-18 for positron emission tomography, the discovery that incorporating fluorine atoms into drug molecules can improve their bioavailability, metabolic stability, and target specificity has driven the rapid development of new methods for generating C–F bonds and forming bond connections with fluorine-containing structural motifs over the past decade.^1^ However, synthesis of compounds bearing fluorovinyl (F–C=C) and *gem*-difluoroallyl (F_2_C–C=C) groups remains a challenge, despite the presence of these structural motifs in numerous drugs, such as tezacitabine,^2^ seletracetam,^3^ and tafluprost^4^ (Scheme 1a). We envisioned that synthesis of fluorovinyls containing an allylic bromine atom (F–C=C–C–Br) would facilitate the preparation of such compounds because the bromine atom would serve as a handle for a wide variety of substitution and cross-coupling reactions. The existing methods for their preparation generally rely on reactions of fluorovinyls containing an allylic hydroxyl group or *gem*-difluorinated vinyloxiranes with brominating reagents.^5^ Direct methods for their synthesis from readily accessible substrates are lacking.

**Scheme 1:**
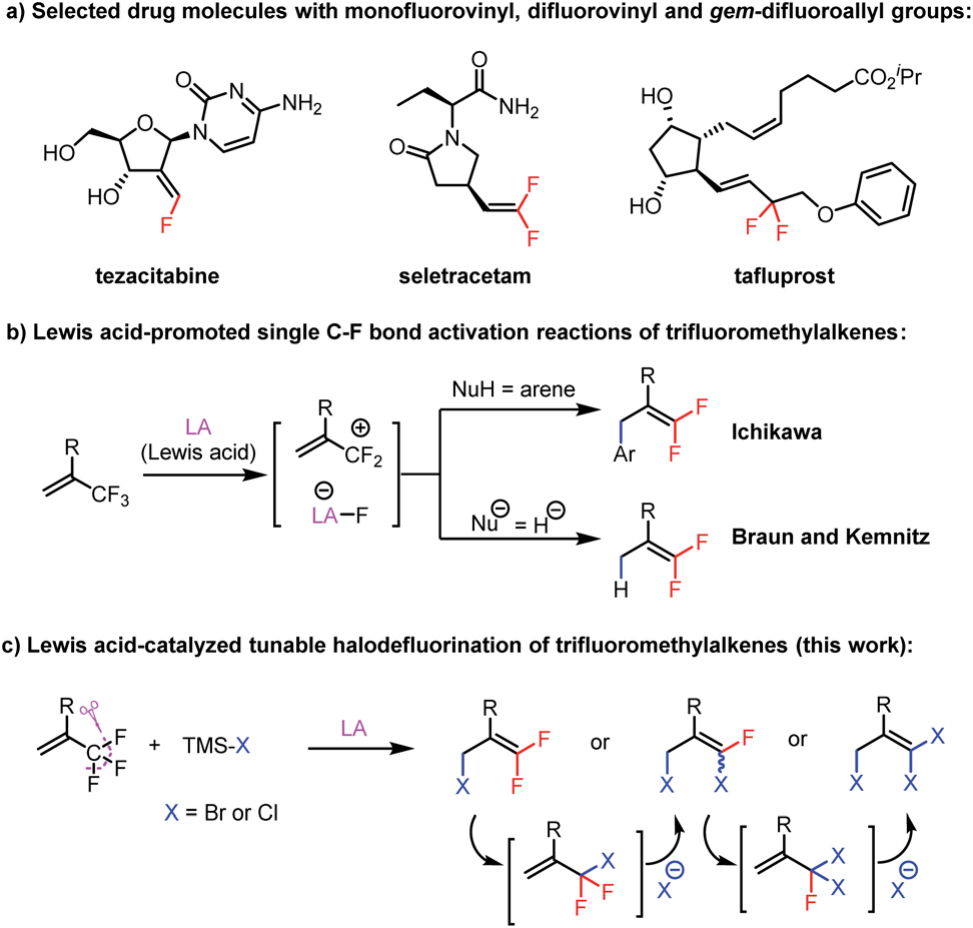
Synthesis of fluorovinyls *via* Lewis acid activation of trifluoromethylalkenes.

Elegant work from the groups of Maruoka,^6^ Oshima,^7^ Ozerov,^8^ Müller,^9^ Stephan,^10^ Oestreich,^11^ Chen,^12^ and Young^13^ on C–F bond activation reactions has proven that Lewis acid-promoted abstraction of fluoride from alkyl fluorides is a powerful tool for generating carbocations that can be trapped by nucleophiles. When trifluoromethylalkenes were studied as substrates, Ichikawa *et al.* reported that aryldefluorination of trifluoromethylalkenes can be accomplished with a stoichiometric amount of EtAlCl_2_*via* fluoride abstraction and subsequent Friedel–Crafts reactions between the resulting allylic carbocation and arenes (Scheme 1b).^14^ In addition, Braun and Kemnitz and colleagues carried out hydrodefluorination reactions of trifluoromethylalkenes with hydrosilanes catalyzed by Lewis acidic nanoscopic aluminum chlorofluoride (Scheme 1b).^15^ In light of these reports and our experiences in developing Lewis acid-catalyzed reactions,^16^ we speculated that 3,3-difluoroallyl bromides (F_2_C=C–C–Br) could be directly prepared from trifluoromethylalkenes and a suitable bromide source *via* Lewis acid activation of the C–F bonds and subsequent nucleophilic attack of the bromide anion at the distal olefinic carbon of the resulting allylic carbocation, a process that has no precedent in the literature.

Herein, we report our discovery that by using an aluminum-based Lewis acid catalyst and bromotrimethylsilane (TMSBr) or chlorotrimethylsilane (TMSCl) as a halide source, we were able to achieve the proposed C–F bond activation/substitution reaction (Scheme 1c). Furthermore, simply by adjusting the stoichiometry of the reactants and the reaction temperature, we could selectively obtain mono-, di-, or trisubstituted products. Mechanistic studies indicated the multi-substitution reaction was achieved by thermally promoted 1,3-halogen migration of the initially formed product, followed by further halodefluorination. Notably, the previously reported defluorination reactions of trifluoromethylalkenes, either Lewis acid-catalyzed^14,15^ or promoted *via* other methods,^17–19^ usually provide monosubstitution products; that is, our finding that we could selectively generate multiply substituted products is also unprecedented.

To test various reaction conditions, we chose α-aryl-substituted trifluoromethylalkene **1a** as a model substrate (Table 1). TMSBr was selected as the bromide source because we expected the generated silyl cation to be an excellent scavenger for the displaced fluoride anion. We began by evaluating several Lewis acid catalysts and found that no reaction occurred when **1a** was treated with B(C_6_F_5_)_3_, Zn(OTf)_2_, Sc(OTf)_3_, Al(OTf)_3_, or ZrCl_4_ (5 mol%) and 3 equiv. of TMSBr in DCE at 80 °C for 24 h (entries 1–5). However, we were encouraged to find that AlCl_3_ would catalyze the proposed bromodefluorination reaction, giving monobrominated product **2a** and dibrominated product **3a** (ref. 20) in 17% and 2% yields, respectively (entry 6). Investigation of additional aluminum-based Lewis acids showed that AlEtCl_2_ and Al(C_6_F_5_)_3_(tol)_0.5_ (ref. 21) had higher activities: AlEtCl_2_ gave **2a** and **3a** in 5% and 38% yields, respectively (entry 7), and Al(C_6_F_5_)_3_(tol)_0.5_ gave 16% and 32% yields, respectively (entry 8). Because Al(C_6_F_5_)_3_(tol)_0.5_ is a solid and therefore easier to store and handle than AlEtCl_2_ (a liquid), we chose Al(C_6_F_5_)_3_(tol)_0.5_ for further investigation. Changing the solvent to toluene inhibited the formation of **3a**, but failed to improve the yield of **2a** (entry 9). Coordinative solvents (acetonitrile and dioxane) shut down the reaction entirely (entries 10 and 11). When the reaction temperature was increased to 120 °C, **2a** and **3a** were obtained in 13% and 68% yields, respectively (entry 13). Gratifyingly, when 4 equiv. of TMSBr relative to **1a** was used, **3a** was generated as the sole reaction product in 90% yield (*Z*/*E* = 55 : 45, entry 14). Next, we tried using TMSBr as the limiting reagent to determine whether we could obtain the monobrominated product (**2a**) as the major product. Indeed, when 3 equiv. of **1a** was treated with 1 equiv. of TMSBr at 80 °C, **2a** was obtained as the sole product, although the yield was only 30% (entry 15). Further screening of reaction conditions revealed that using 9.0 mol% of Al(C_6_F_5_)_3_(tol)_0.5_ and running the reaction at 60 °C for 48 h (entry 16) gave the highest yield of **2a** (76%; the yield of **3a** was 8%).

**Table 1:** Optimization of reaction conditions^a^

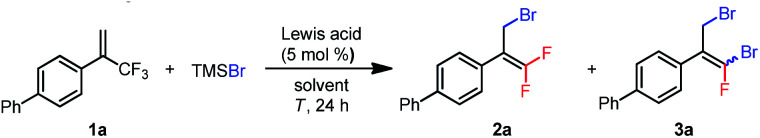						
Entry	Lewis acid	**1a**/TMSBr	*T* (°C)	Solvent	Yield^b^**2a** (%)	Yield^b^**3a** (%)
1	B(C_6_F_5_)_3_	1 : 3	80	DCE	n.d.	n.d.
2	Zn(OTf)_2_	1 : 3	80	DCE	n.d.	n.d.
3	Sc(OTf)_3_	1 : 3	80	DCE	n.d.	n.d.
4	Al(OTf)_3_	1 : 3	80	DCE	n.d.	n.d.
5	ZrCl_4_	1 : 3	80	DCE	Trace	n.d.
6	AlCl_3_	1 : 3	80	DCE	17	2
7	AlEtCl_2_	1 : 3	80	DCE	5	38
8^c^	Al(C_6_F_5_)_3_(tol)_0.5_	1 : 3	80	DCE	16	32
9^c^	Al(C_6_F_5_)_3_(tol)_0.5_	1 : 3	80	Toluene	16	n.d.
10^c^	Al(C_6_F_5_)_3_(tol)_0.5_	1 : 3	80	CH_3_CN	n.d.	n.d.
11^c^	Al(C_6_F_5_)_3_(tol)_0.5_	1 : 3	80	Dioxane	n.d.	n.d.
12^c^	Al(C_6_F_5_)_3_(tol)_0.5_	1 : 3	100	DCE	25	48
13^c^	Al(C_6_F_5_)_3_(tol)_0.5_	1 : 3	120	DCE	13	68
14^c^	Al(C_6_F_5_)_3_(tol)_0.5_	1 : 4	120	DCE	n.d.	90^d^
15^c^	Al(C_6_F_5_)_3_(tol)_0.5_	3 : 1	80	DCE	30	n.d.
16^e^	Al(C_6_F_5_)_3_(tol)_0.5_	3 : 1	60	DCE	76	8

a Unless otherwise specified, reactions were performed with 0.1 mmol of **1a** and 5 mol% of a Lewis acid in 1 mL of solvent for 24 h under N_2_.

b Yields were determined by ^1^H NMR using CH_2_Br_2_ as the internal standard; the **2a**/**3a** ratios were determined by ^19^F NMR; n.d. = not detected.

c 4.5 mol% Al(C_6_F_5_)_3_(tol)_0.5_ was used as catalyst.

d The *Z/E* ratio was 55 : 45.

e The reaction was carried out with 9.0 mol% of Al(C_6_F_5_)_3_(tol)_0.5_ for 48 h.

With the optimal conditions in hand, we first explored the scope of the monosubstitution reaction by testing various trifluoromethyl- and difluoroalkyl-substituted olefins **1** (Table 2, left column). From **1a**, monobrominated product **2a** could be isolated in pure form in 64% yield by means of preparative HPLC. When the α-phenyl ring bore an *ortho*-phenyl substituent, the reaction still afforded **2b** in 58% yield despite the increased steric bulk around the reaction site. When the α substituent was changed to a 9-phenanthryl group (**1c**), monobrominated product **2c** was isolated in 75% yield. We also tested other halogenating reagents with **1c**: TMSI gave iodinated product **2c-I** in 51% yield, whereas TMSCl was poorly reactive, giving a <10% yield of product. Furthermore, substrates with 1-naphthyl (**2d**), 4-dibenzothiophenyl (**2e**), and 4-dibenzofuranyl (**2f**) moieties at the α position were all suitable. Interestingly, even the reaction of conjugated diene **1g** was feasible, giving brominated diene **2g** in 67% isolated yield. In addition, a series of α-alkyl-substituted trifluoromethylalkenes gave the desired products (**2h–2k**) in moderate yields. Difluoroalkyl-substituted alkenes were also reactive; specifically, benzene-fused methylenecycloalkanes **1l–1n** gave the corresponding products (**2l–2n**) in 59–88% yields. Finally, acyclic substrate **1o** afforded (*E*)-**2o** as the predominant isomer (*E*/*Z* > 10 : 1) in 50% yield.

**Table 2:** Scope of the mono and disubstitution reaction^a^

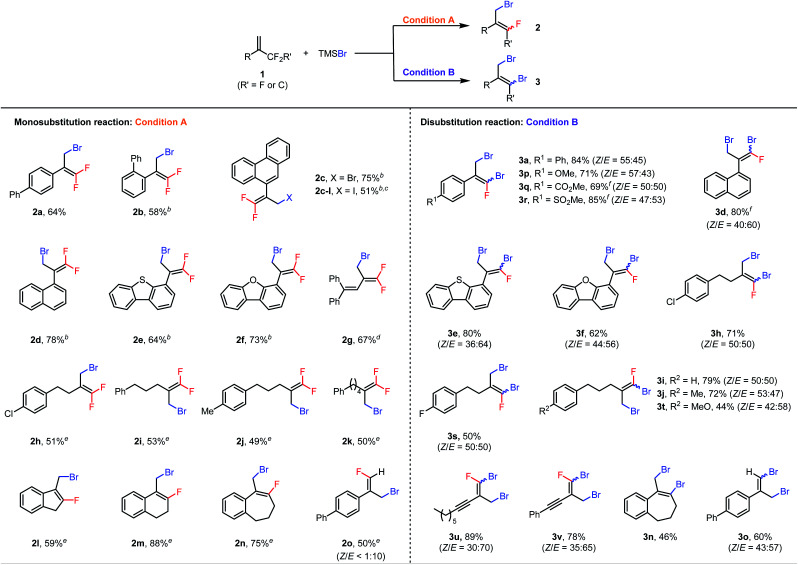

a Condition A: reactions were performed with 0.6 mmol of **1**, 0.2 mmol of TMSBr, and 9.0 mol% of Al(C_6_F_5_)_3_(tol)_0.5_ in 1.5 mL of DCE at 60 °C for 48 h; condition B: reactions were performed with 0.2 mmol of **1**, 0.8 mmol of TMSBr, and 4.5 mol% of Al(C_6_F_5_)_3_(tol)_0.5_ in 1.5 mL of DCE at 120 °C for 24 h; isolated yields are reported.

b The reaction was performed at 80 °C.

c TMSI was used instead of TMSBr.

d The reaction was carried out with 13.5 mol% of Al(C_6_F_5_)_3_(tol)_0.5_.

e 4 equiv. of **1** was used.

f The reaction was performed with 5 equiv. of TMSBr.

Next the scope of the disubstitution reaction was investigated (Table 2, right column). Trifluoromethyl-substituted alkenes bearing electron-donating or electron-withdrawing groups on the α-aryl ring were reactive, affording the corresponding products (**3a** and **3p–3r**) in 69–85% yields with *Z*/*E* ratios of approximately 1 : 1. 1-Naphthyl (**3d**), 4-dibenzothiophenyl (**3e**), 4-dibenzofuranyl (**3f**), and aliphatic (**3h–3j**, **3s**, and **3t**) substituents at the α position were well tolerated. Interestingly, even alkynyl-substituted trifluoromethylalkenes afforded the desired disubstituted products (**3u** and **3v**) in good yields. In addition, difluoroalkyl-substituted alkenes **1n** and **1o** gave completely defluorinated products **3n** and **3o** in 46% and 60% yields, respectively. Notably, under these conditions, the monobrominated products either did not form or formed in only trace amounts, as indicated by GC-MS or NMR spectroscopy. Moreover, the *E* and *Z* isomers of dibrominated products were found interconvertible under the reaction conditions (for details, see the ESI†) so the *Z*/*E* ratios of products might be the result of the thermodynamic equilibrium.

It is also worth mentioning that some substrates shown in Table 2 were not compatible either with the monosubstitution reaction or with the disubstitution reaction. For example, substrates bearing coordinative functional groups, such as methoxy, carbonyl, sulfonyl and alkyne (**1p**, **1q**, **1r**, **1t**, **1u**, and **1v**), gave very low yields (<20%) for monosubstitution, perhaps because the relatively low reaction temperature (60 °C) was not sufficient to break the coordination of these functional groups to the Lewis acid catalyst. Furthermore, Al(C_6_F_5_)_3_(tol)_0.5_ is probably a precatalyst because Al(C_6_F_5_)_3_(tol)_0.5_ rapidly decomposes in DCE to give a mixture of unidentified aluminum species^21*b*^ that are active for the halodefluorination reaction (for details, see the ESI†).

We performed several control experiments to explore the reaction mechanism. When substrate **1a** was treated with mesitylene in the presence of 1 equiv. of Al(C_6_F_5_)_3_(tol)_0.5_, Friedel–Crafts allylation of the aromatic ring generated product **4** in 96% yield (Scheme 2a).^22^ This result demonstrates that the aluminum Lewis acid could abstract fluoride from the trifluoromethylalkene to generate an allylic carbocation. Furthermore, when **2a** was subjected to the conditions used for the disubstitution reaction, **3a** was isolated in 65% yield (Scheme 2b), indicating that the dibrominated products were generated *via* monobrominated intermediates. However, subjecting nonbrominated **5** to the same conditions did not result in substitution of the vinylic fluorine atom by the bromine atom (**6**, Scheme 2c), which excludes the vinylic nucleophilic substitution (S_N_V) mechanism^23^ for the conversion from **2a** to **3a**. We thus suspected that the allylic bromine atom in **2a** was involved in this conversion. Indeed, when **2a** was heated at 120 °C in toluene for 12 h, 1,3-migration of the bromine atom gave bromodifluoromethylalkene **7** in 83% NMR yield (Scheme 2d).^24^ And, treatment of **7** with TMSBr in the presence of the catalyst at 120 °C gave **3a** in 77% yield (Scheme 2e). Taken together, these results indicate that dibrominated products were generated *via* isomerization of the monobrominated product to form bromodifluoromethylalkenes, which then underwent a second bromodefluorination reaction. In addition, silylium Et_3_Si[B(C_6_F_5_)_4_]^25^ was found incapable of catalyzing the bromodefluorination reaction (Scheme 2f). This result suggests that the Lewis acidic aluminum is probably a catalyst, rather than an initiator, and TMS^+^ from TMSBr abstracts the fluoride from the aluminum–fluoride adduct to regenerate the active catalyst.

**Scheme 2:**
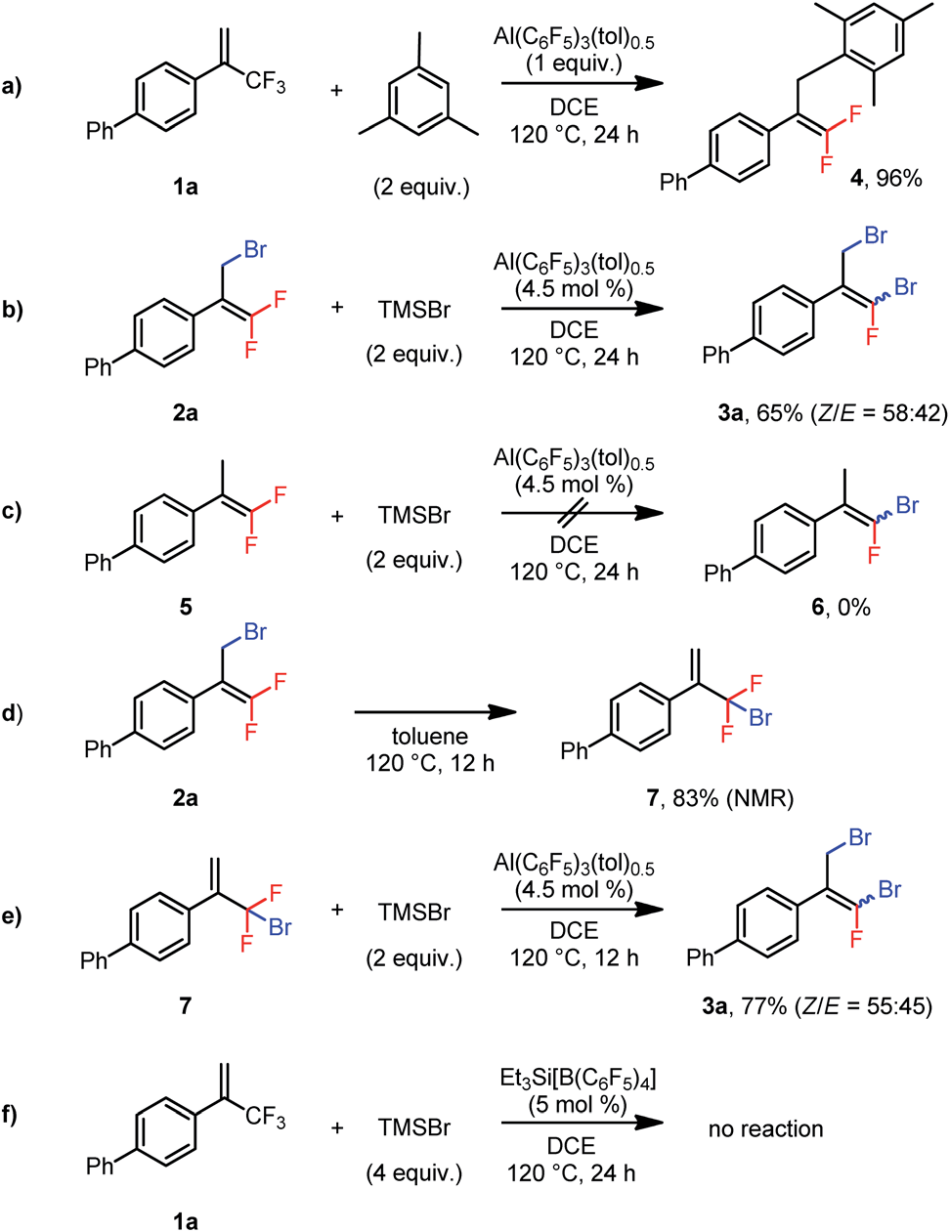
Control experiments.

These results led us to wonder whether all three fluorine atoms of a trifluoromethylalkene could be replaced with bromine atoms *via* a 1,3-bromo migration reaction of the dibrominated product to give a dibromofluoromethylalkene, which would then undergo bromodefluorination. After screening various reaction conditions, we discovered that tribrominated products could be obtained by using a large excess (*e.g.*, 10 equiv.) of TMSBr and extending the reaction time; however, in all cases, substantial amounts of the dibrominated products were always produced as well (see Table S1 in the ESI†), which made separation of the product difficult. However, we were delighted to find that when TMSCl was used in large excess (7 equiv.) and the reaction temperature was 120 °C, trichlorinated compounds were the major or only products (Table 3). However, these conditions were suitable only for substrates bearing α-aryl substituents. The moderate to low yields of these reactions were due mainly to decomposition of the starting materials rather than to the formation of mono- or dichlorinated byproducts.

**Table 3:** Scope of trisubstitution reaction^a^

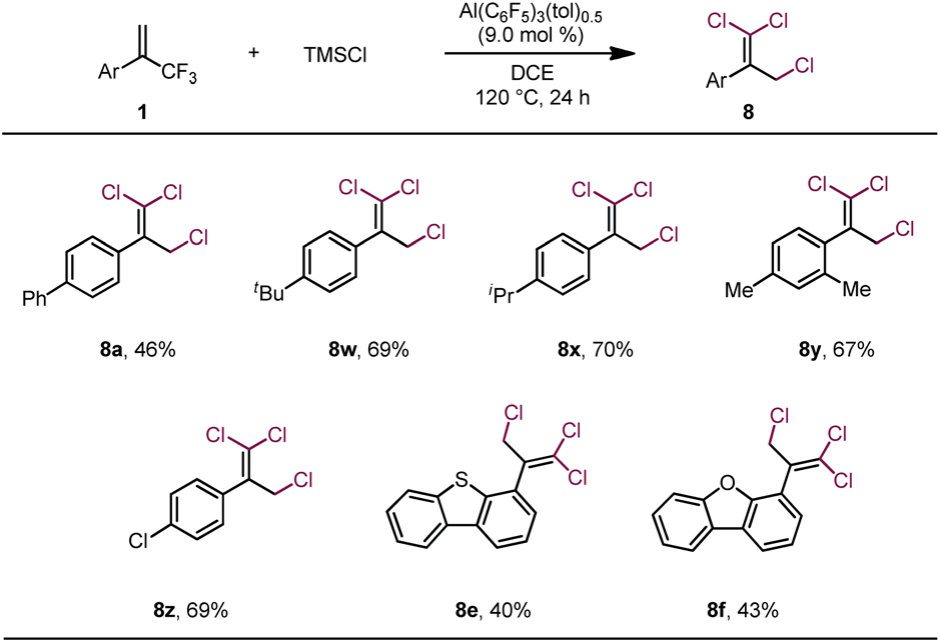

a Unless otherwise specified, reactions were performed with 0.2 mmol of **1**, 1.4 mmol of TMSCl, and 9.0 mol% of Al(C_6_F_5_)_3_(tol)_0.5_ in 1.5 mL of DCE at 120 °C for 24 h; isolated yields are reported.

As mentioned above, bromine atoms are among the most useful substituents for introducing other functional groups. To explore the utility of the above-described reactions, we carried out some transformations of the products (Scheme 3). For example, treatment of monobrominated product **2d** with estrone under basic conditions delivered phenoxy-substituted product **9** in 65% yield *via* an S_N_2′ reaction. Additionally, azide and an indole were also suitable nucleophiles for S_N_2′ reactions, giving **10** and **11** in 50% and 79% isolated yields, respectively. Furthermore, a Suzuki coupling reaction of **2d** with an arylboronic acid delivered coupling product **12** in 61% yield, and treatment of **2d** with hexaldehyde gave alcohol **13** (63% yield) *via* an indium-mediated *gem*-difluoroallylation reaction.^5*b*^ Reaction of dibrominated product **3a** with an allyl Grignard reagent selectively replaced the allylic bromide to give compound **14**. Subsequent electrophilic fluorination of **14** with Selectfluor in the presence of MeOH afforded α-CF_2_Br-substituted ether **15** in 41% yield. In addition, **14** could undergo a Pd-catalyzed intramolecular Heck reaction to generate fluoro-substituted cyclopentene **16** in 54% yield. Notably, both (*Z*)- and (*E*)-**14** underwent these last two transformations to give a single product, thus eliminating the need to separate the isomers.

**Scheme 3:**
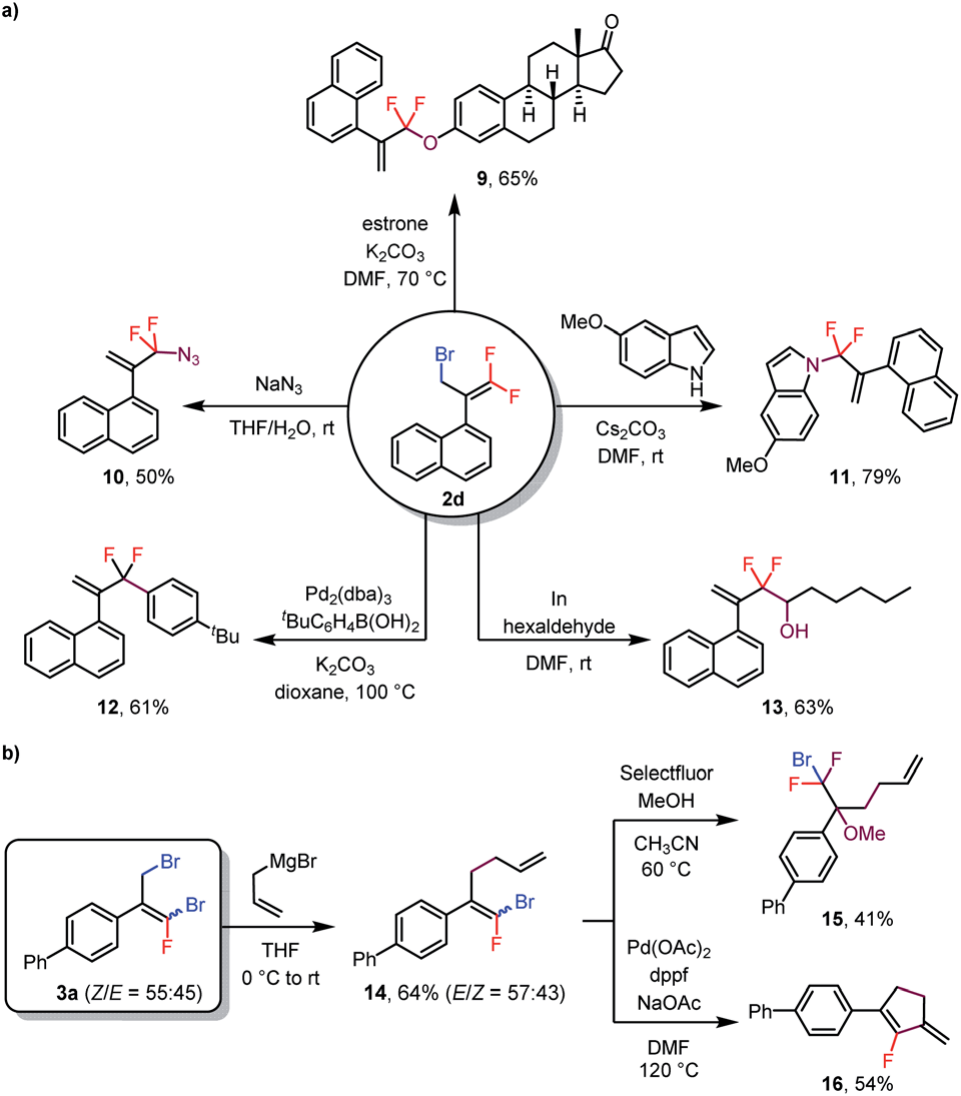
Transformations of products **2d** and **3a**.

## Conclusions

In summary, we have developed a protocol for aluminum-catalyzed halodefluorination reactions of trifluoromethyl- and difluoroalkylalkenes, which provide convenient access to fluorovinyls bearing an allylic halogen atom. Furthermore, di- and trisubstituted products could also be selectively synthesized by 1,3-migration of a halogen atom in the initially formed products and subsequent halodefluorination. The fluorine-containing products are useful building blocks for a variety of transformations. We are currently investigating the use of other nucleophiles for these aluminum-promoted defluorination reactions.

## Conflicts of interest

There are no conflicts to declare.

## Supplementary Material

SC-011-D0SC03883K-s001

SC-011-D0SC03883K-s002
