# Supplementary material for: Aluminum-catalyzed tunable halodefluorination of trifluoromethyl- and difluoroalkyl-substituted olefins
Source: Chem Sci. 2020 Oct 1;11(42):11548–53. doi: 10.1039/d0sc03883k (PMC8162833; doi:10.1039/d0sc03883k)

Electronic Supplementary Material (ESI) for Chemical Science.
This journal is © The Royal Society of Chemistry 2020

SUPPORTING INFORMATION

Aluminum–Catalyzed Tunable Halodefluorination of Trifluoromethyl- and Difluoroalkyl-Substituted Olefins

Zhong Liu, Xian-Shuang Tu, Le-Tao Guo and Xiao-Chen Wang*

State Key Laboratory and Institute of Elemento-Organic Chemistry, College of Chemistry, Nankai University, Tianjin 300071, China

Table of Contents

1. General Information S1

2. Preparation of the Starting Materials S1

3. Preparation of $Al(C_6F_5)_3(tol)_{0.5}$ S5

4. Monosubstitution Reactions S5

5. Disubstitution Reactions S13

6. *E* and *Z* Isomerization of the Dibrominated Products S22

7. Decomposition of $Al(C_6F_5)_3(tol)_{0.5}$ in DCE S22

8. Trisubstitution Reactions S23

   8.1 Optimization of Reaction Conditions S23

   8.2 Scope of the Trichlorination Reaction S24

9. Transformations of Product **2d** and **3a** S26

10. Mechanistic Studies. S33

11. References S36

12. NMR Spectra S37

1. General Information

Unless otherwise noted, all materials were used as received from commercial sources without further purification. Dry solvents were obtained by distillation from drying reagents according to procedures described in *Purification of Laboratory Chemicals (5th Edition)* written by Wilfred L.F. Armarego and Christina L.L. Chai (Elsevier, 2003). Analytical thin layer chromatography (TLC) was performed on Huanghai precoated (0.25 mm thickness) silica gel plates with F254 indicator. Visualization was accomplished with UV light (254 nm) or the potassium permanganate stain solution. Flash chromatography was carried out with silica gel (32–63 μm) supplied by Sanpont Group. ^{1}H NMR spectra were recorded on a Bruker DRX-400 (400 MHz) spectrometer and chemical shifts were reported in ppm. The peak information was described as: br = broad, s = singlet, d = doublet, t = triplet, q = quartet, m = multiplet; coupling constants in Hz. ^{13}C NMR spectra were recorded on a Bruker DRX-400 (101 MHz) spectrometer with complete proton decoupling. Chemical shifts of the NMR spectra were calibrated by the literature values of the solvent residual peaks. High-resolution mass spectra (HRMS) were obtained either on a Waters GCT Premier TOF mass spectrometer or on an Agilent 6520 Q-TOF LC/MS.

2. Preparation of the Starting Materials

Substrates **1a–1f**[1], **1g**[2], **1l–1o**[3,4], **1p–1r**[1], **1u–1v**[2], **1w–1z**[1], **5**[5] were prepared according to the previous literatures. Substrates **1a**[1], **1b**[7], **1d**[1], **1f**[8], **1g**[2], **1i**[9], **1l**[3], **1m**[3], **1n**[4], **1o**[4], **1p**[1], **1q**[1], **1r**[10], **1u**[11], **1v**[11], **1w**[1], **1x**[7], **1y**[1] and **1z**[1] had been fully characterized in the previous literatures.

Preparation of Substrates 1h–1k, 1s and 1t:[1,12]

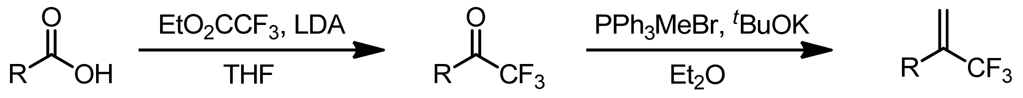

General Procedure: (1) Preparation of trifluoromethylketones: To a 100 mL round bottom flask equipped with a magnetic stir bar was added a solution of carboxylic acid

(10.0 mmol, 1 equiv.) in THF (20 mL) under argon atmosphere. The solution was cooled to -20 °C, and then LDA (2 M in THF/Hexane, 22.0 mmol, 2.2 equiv.) was added dropwise over 10 min. The reaction mixture was allowed to slowly warm to 20 °C and stirred for 4 h. In a separate flask, a solution of ethyl trifluoroacetate (30.0 mmol, 3 equiv.) in THF (10 mL) was cooled to -65 °C under argon atmosphere. The enediolate solution was added dropwise via a syringe to the ethyl trifluoroacetate solution. The resulting mixture was stirred at -65 °C for 2 h and then was quenched with 6 N HCl (20 mL). The reaction mixture was diluted with EtOAc (10 mL), and the separated organic phase was dried over Na_2SO_4, filtered, and concentrated in vacuo. The residue was purified by silica gel flash chromatography using PE/EA as the eluents to obtain the trifluoromethylketone.

(2) Preparation of trifluoromethylalkenes: To a 100 mL round bottom flask equipped with a magnetic stir bar were added Ph_3PMeBr (2.7 g, 7.5 mmol, 1.5 equiv.) and Et_2O (20 mL). The obtained solution was cooled to 0 °C, followed by the addition of ^{t}BuOK (0.84 g, 7.5 mmol, 1.5 equiv.). The mixture was then allowed to warm to room temperature and stirred for 24 h. Subsequently, the mixture was cooled to -78 °C. A solution of trifluoromethylketone (5.0 mmol, 1.0 equiv.) in THF (2 mL) was added to the mixture. The resulting mixture was allowed to warm slowly to room temperature and stirred for 24 h, and then was quenched with saturated aqueous NH_4Cl (5 mL). After extraction of the mixture with Et_2O (3×20 mL), the combined organic phase was dried over Na_2SO_4, filtered and concentrated in vacuo. The residue was purified by silica gel flash chromatography using PE/EA as the eluents to obtain the trifluoromethylalkene.

9-(3,3,3-trifluoroprop-1-en-2-yl)phenanthrene (1c)

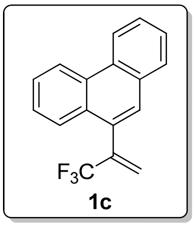

^{1}H NMR (400 MHz, $CDCl_3$) δ 8.68 – 8.64 (m, 2H, Ar**H**), 7.97 (d, $^3J_{H-H}$ = 7.9 Hz, 1H, Ar**H**), 7.85 (d, $^3J_{H-H}$ = 7.8 Hz, 1H, Ar**H**), 7.70 – 7.56 (m, 5H, Ar**H**), 6.35 (s, 1H, =C**H**$_2$), 5.70 (s, 1H, =C**H**$_2$). ^{13}C NMR (101 MHz, $CDCl_3$) δ 137.9 (q, $^2J_{C-F}$ = 31.2 Hz, =**C**CF_3), 131.1, 131.0,

130.7, 130.4, 129.1, 128.9, 127.6, 127.2, 127.1, 127.0, 126.4, 124.7 (q, $^{3}J_{C-F}$ = 5.1 Hz), 123.4 (q, $^{1}J_{C-F}$ = 274.72 Hz, **C**F3), 123.1, 122.8. ^{19}F NMR (376 MHz, $CDCl_3$) δ -66.92 (s, 3F). HRMS-EI: m/z calculated for $C_{17}H_{11}F_3$ [M^+] 272.0807, found 272.0799.

4-(3,3,3-trifluoroprop-1-en-2-yl)dibenzo[b,d]thiophene (1e)

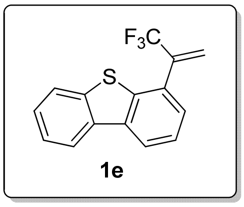

^{1}H NMR (400 MHz, $CDCl_3$) δ 8.13 – 8.09 (m, 2H, Ar**H**), 7.81 – 7.79 (m, 1H, Ar**H**), 7.46 – 7.42 (m, 4H, Ar**H**), 6.30 (s, 1H, =C**H**2), 6.03 (s, 1H, =C**H**2). ^{13}C NMR (101 MHz, $CDCl_3$) δ 140.3, 139.4, 138.0 (q, $^{2}J_{C-F}$ = 31.5 Hz, =**C**CF3), 136.6, 135.9, 128.8, 127.3, 126.7, 124.8, 124.8, 123.8 (q, $^{3}J_{C-F}$ = 5.4 Hz), 123.4 (q, $^{1}J_{C-F}$ = 274.7 Hz, **C**F3), 122.8, 122.2, 121.9. ^{19}F NMR (376 MHz, $CDCl_3$) δ -66.32 (s, 3F). HRMS-EI: m/z calculated for $C_{15}H_9F_3S$ [M^+] 278.0372, found 278.0364.

1-chloro-4-(3-(trifluoromethyl)but-3-en-1-yl)benzene (1h)

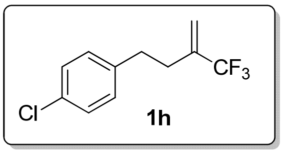

^{1}H NMR (400 MHz, $CDCl_3$) δ 7.24 (d, $^{3}J_{H-H}$ = 8.2 Hz, 2H, Ar**H**), 7.09 (d, $^{3}J_{H-H}$ = 8.2 Hz, 2H, Ar**H**), 5.66 (s, 1H, =C**H**2), 5.24 (s, 1H, =C**H**2), 2.78 (t, $^{3}J_{H-H}$ = 7.9 Hz, 2H, C**H**2), 2.47 (t, $^{3}J_{H-H}$ = 7.9 Hz, 2H, C**H**2). ^{13}C NMR (101 MHz, $CDCl_3$) δ 139.3, 137.6 (q, $^{2}J_{C-F}$ = 29.3 Hz, =**C**CF3), 132.3, 130.1, 128.9, 124.0 (q, $^{1}J_{C-F}$ = 273.7 Hz, **C**F3), 118.8 (q, $^{3}J_{C-F}$ = 5.9 Hz), 33.4, 31.4. ^{19}F NMR (376 MHz, $CDCl_3$) δ -69.46 (s, 3F). HRMS-EI: m/z calculated for $C_{11}H_{10}ClF_3$ [M^+] 234.0418, found 234.0411.

1-methyl-4-(4-(trifluoromethyl)pent-4-en-1-yl)benzene (1j)

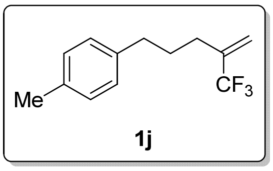

^{1}H NMR (400 MHz, $CDCl_3$) δ 7.08 (q, J = 7.9 Hz, 4H, Ar**H**), 5.66 (s, 1H, =C**H**2), 5.30 (s, 1H, =C**H**2), 2.61 (t, $^{3}J_{H-H}$ = 7.7 Hz, 2H, C**H**2), 2.32 (s, 3H), 2.23 (t, $^{3}J_{H-H}$ = 7.7 Hz, 2H, C**H**2), 1.86 – 1.79 (m, 2H, C**H**2). ^{13}C NMR (101 MHz, $CDCl_3$) δ 138.8, 138.7 (q, $^{2}J_{C-F}$ = 30.3 Hz, =**C**CF3), 135.7, 129.3, 128.5, 124.1 (q, $^{1}J_{C-F}$ = 274.7 Hz, **C**F3), 117.8 (q, $^{3}J_{C-F}$ = 5.8 Hz), 35.0, 29.4, 29.2, 21.2. ^{19}F NMR (376 MHz, $CDCl_3$) δ -68.98 (s, 3F). HRMS-EI: m/z

calculated for $C_{13}H_{15}F_3$ [M^+] 228.1120, found 228.1117.

(5-(trifluoromethyl)hex-5-en-1-yl)benzene (1k)

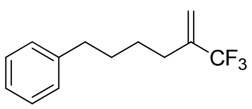

1k

^{1}H NMR (400 MHz, $CDCl_3$) δ 7.26 – 7.23 (m, 2H, Ar**H**), 7.16 – 7.15 (m, 3H, Ar**H**), 5.62 (s, 1H, =C**H**$_2$), 5.24 (s, 1H, =C**H**$_2$), 2.62– 2.59 (m, 2H, C**H**$_2$), 2.20 – 2.20 (m, 2H, C**H**$_2$), 1.67 – 1.63 (m, 2H, C**H**$_2$), 1.55 – 1.53 (m, 2H, C**H**$_2$). ^{13}C NMR (101 MHz, $CDCl_3$) δ 142.4, 138.6 (q, $^2J_{C-F}$ = 29.29 Hz, =**C**CF_3), 128.6, 128.6, 126.0, 124.1 (q, $^1J_{C-F}$ = 274.7 Hz, **C**F_3), 117.6 (q, $^3J_{C-F}$ = 5.8 Hz), 35.9, 31.0, 29.4, 27.2. ^{19}F NMR (376 MHz, $CDCl_3$) δ -69.05 (s, 3F). HRMS-EI: m/z calculated for $C_{13}H_{15}F_3$ [M^+] 228.1120, found 228.1115.

1-fluoro-4-(3-(trifluoromethyl)but-3-en-1-yl)benzene (1s)

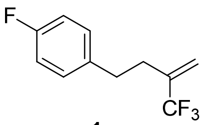

1s

^{1}H NMR (400 MHz, $CDCl_3$) δ 7.16 – 7.12 (m, 2H, Ar**H**), 6.97 (t, $^3J_{H-H}$ = 8.4 Hz, 2H, Ar**H**), 5.68 (s, 1H, =C**H**$_2$), 5.26 (s, 1H, =C**H**$_2$), 2.81 (t, $^3J_{H-H}$ = 7.7 Hz, 2H, C**H**$_2$), 2.49(t, $^3J_{H-H}$ = 7.7 Hz, 2H, C**H**$_2$). ^{13}C NMR (101 MHz, $CDCl_3$) δ 161.7 (d, $^1J_{C-F}$ = 244.0 Hz), 137.7 (q, $^2J_{C-F}$ = 29.2 Hz, =**C**CF_3), 136.4 (d, $^4J_{C-F}$ = 3.2 Hz), 130.0 (d, $^3J_{C-F}$ = 7.7 Hz), 124.0 (q, $^1J_{C-F}$ = 274.7 Hz, **C**F_3), 119.0 (q, $^3J_{C-F}$ = 5.8 Hz), 115.4 (q, $^2J_{C-F}$ = 21.2 Hz), 33.2, 31.6. ^{19}F NMR (376 MHz, $CDCl_3$) δ -68.40 (s, 3F), -117.02 – -117.09 (m, 1F). HRMS-EI: m/z calculated for $C_{11}H_{10}F_4$ [M^+] 218.0713, found 218.0708.

1-methoxy-4-(4-(trifluoromethyl)pent-4-en-1-yl)benzene (1t)

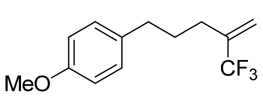

1t

^{1}H NMR (400 MHz, $CDCl_3$) δ 7.13 (d, J = 8.3 Hz, 2H, Ar**H**), 6.87 (d, J = 8.4 Hz, 2H, Ar**H**), 5.70 (s, 1H, =C**H**$_2$), 5.34 (s, 1H, =C**H**$_2$), 3.82 (s, 3H, C**H**$_3$), 2.64 (t, $^3J_{H-H}$ = 7.6 Hz, 2H, C**H**$_2$), 2.26 (t, $^3J_{H-H}$ = 7.7 Hz, 2H, C**H**$_2$), 1.89 – 1.81 (m, 2H, C**H**$_2$). ^{13}C NMR (101 MHz, $CDCl_3$) δ 158.1, 138.6 (q, $^2J_{C-F}$ = 29.1 Hz, =**C**CF_3), 133.9, 129.5, 124.0 (q, $^1J_{C-F}$ = 274.7 Hz, **C**F_3), 117.8 (q, $^3J_{C-F}$ = 5.8 Hz), 114.0, 55.4, 34.5, 29.5, 29.1. ^{19}F NMR (376 MHz, $CDCl_3$) δ -69.00 (s, 3F). HRMS-EI: m/z calculated for $C_{13}H_{15}F_3O$ [M^+] 244.1070,

found 244.1065.

3. Preparation of $Al(C_6F_5)_3(tol)_{0.5}$[13]

In a glovebox, $B(C_6F_5)_3$ (512.0 mg, 1.0 mmol, 1.0 equiv.), toluene (1.0 mL) and hexanes (3.0 mL) were added to a 50 mL Schlenk tube and stirred for 15 minutes at room temperature. $AlMe_3$ (1.0 M in heptanes, 1 mL, 1.0 mmol, 1.0 equiv.) was then added. The mixture was stirred at room temperature overnight, and a white precipitate was formed. The precipitate was isolated by filtration and washed three times with hexanes. The obtained material was dried under vacuum to afford $Al(C_6F_5)_3(tol)_{0.5}$ as a white solid (240.0 mg , 45%). The compound was stored in the freezer of the glovebox. $Al(C_6F_5)_3$(tol)0.5 is heat- and shock-sensitive, so extra caution and use of protective equipment (e.g. a blast shield) is necessary when handling this material.

$Al(C_6F_5)_3(tol)_{0.5}$ ^1H NMR (400 MHz, C_6D_6) δ 7.12 – 6.99 (m, 5H, Ar**H**), 2.09 (s, 3H, C**H**$_3$). ^{19}F NMR (376 MHz, C_6D_6) δ -123.20 (d, $^3J_{F\text{-}F}$ = 18.8 Hz, 6F), -150.82 (t, $^3J_{F\text{-}F}$ = 18.8 Hz, 3F), -160.68 (m, 6F).

4. Monosubstitution Reactions

General procedure: In a glovebox, substrate **1** (0.6 mmol, 3.0 equiv.), TMSBr (30.6 mg, 0.2 mmol, 1.0 equiv.) and $Al(C_6F_5)_3(tol)_{0.5}$ (10.4 mg, 0.018 mmol, 9.0 mol %) were dissolved in DCE (1.5 mL) in an 8 mL vial containing a magnetic stir bar. The vial was sealed. The mixture was stirred at 60 °C in a heating block for 48 h. After being allowed to cool to room temperature, the solution was concentrated in vacuo, and the residue was purified by flash chromatography on silica gel using petroleum ether (PE) as the eluent to give the monobrominated product. The analytical data for these products are listed as follows.

4-(3-bromo-1,1-difluoroprop-1-en-2-yl)-1,1'-biphenyl (2a)

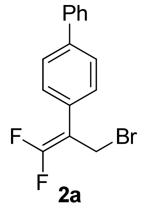

2a

2a was purified by flash chromatography on silica gel using PE as the eluent and preparative HPLC (C18 column with water/ acetonitrile as the eluent). White solid (39 mg, 64% yield). ^{1}H NMR (400 MHz, $CDCl_3$) δ 7.64 – 7.60 (m, 4H, Ar**H**), 7.52 – 7.49 (m, 2H, Ar**H**), 7.45 (t, J = 7.5 Hz, 2H, Ar**H**), 7.39 – 7.34 (m, 1H, Ar**H**), 4.36 (t, $^4J_{H\text{-}F}$= 2.0 Hz, 2H, C**H**$_2$). ^{13}C NMR (101 MHz, $CDCl_3$) δ 154.8 (t, $^1J_{C\text{-}F}$ = 299.0 Hz, =**C**F$_2$), 141.2, 140.5, 130.4 (t, J = 3.0 Hz), 129.1, 128.5 (t, J = 3.0 Hz), 127.8, 127.6, 127.3, 92.1 (t, $^2J_{C\text{-}F}$ = 17.0 Hz, **C**=CF$_2$), 28.0 (d, $^3J_{C\text{-}F}$ = 4.0 Hz, **C**H$_2$Br). ^{19}F NMR (376 MHz, $CDCl_3$) δ -82.76 (d, $^2J_{F\text{-}F}$ = 20.8 Hz, 1F), -84.10 (d, $^2J_{F\text{-}F}$ = 20.8 Hz, 1F). HRMS-EI: m/z calculated for $C_{15}H_{11}BrF_2$ [M$^+$] 308.0007, found 308.0010.

2-(3-bromo-1,1-difluoroprop-1-en-2-yl)-1,1'-biphenyl (2b)

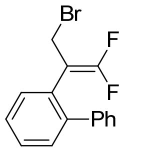

2b

Reaction conditions: **1b** (3.0 equiv.), TMSBr (1.0 equiv.), $Al(C_6F_5)_3(tol)_{0.5}$ (9.0 mol %), DCE, 80 °C. **2b** was purified by flash chromatography on silica gel using PE as the eluent. Colorless oil (35 mg, 58 % yield). ^{1}H NMR (400 MHz, $CDCl_3$) δ 7.47 – 7.34 (m, 9H, Ar**H**), 3.76 (s, 2H, C**H**$_2$). ^{13}C NMR (101 MHz, $CDCl_3$) δ 154.5 (dd, $^1J_{C\text{-}F}$ = 295.9, 292.5 Hz, =**C**F$_2$). 141.8 (d, J = 3.0 Hz), 140.7, 131.6, 130.6, 129.2, 128.8, 128.7, 127.7, 127.7, 92.4 (t, $^2J_{C\text{-}F}$ = 20.0 Hz, **C**=CF$_2$), 28.8 (d, $^3J_{C\text{-}F}$ = 5.6 Hz, **C**H$_2$Br). ^{19}F NMR (376 MHz, $CDCl_3$) δ -89.13 (d, $^2J_{F\text{-}F}$ = 23.5 Hz, 1F), -90.39 (d, $^2J_{F\text{-}F}$ = 23.5 Hz, 1F). HRMS-EI: m/z calculated for $C_{15}H_{11}F_2^+$ [M$^+$ - Br] 229.0823, found [M$^+$ - Br] 229.0823.

9-(3-bromo-1,1-difluoroprop-1-en-2-yl)phenanthrene (2c)

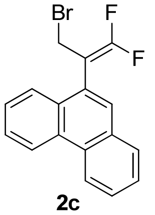

2c

Reaction conditions: **1c** (3.0 equiv.), TMSBr (1.0 equiv.), $Al(C_6F_5)_3(tol)_{0.5}$ (9.0 mol %), DCE, 80 °C. **2c** was purified by flash chromatography on silica gel using PE as the eluent. Colorless oil (50 mg, 75% yield). ^{1}H NMR (400 MHz, $CDCl_3$) δ 8.75 (d, $^3J_{H\text{-}H}$ = 8.2 Hz, 1H, Ar**H**), 8.69 (d, $^3J_{H\text{-}H}$ = 8.2 Hz, 1H, Ar**H**), 7.92 (d, $^3J_{H\text{-}H}$ = 7.8 Hz, 1H,

ArH), 7.86 (d, $^{3}J_{H\text{-}H}$ = 8.0 Hz, 1H, ArH), 7.80 (s, 1H, ArH), 7.71 – 7.67 (m, 2H, ArH), 7.66 – 7.61 (m, 2H, ArH), 4.43 (d, J = 106.5 Hz, 2H, CH$_2$). ^{13}C NMR (101 MHz, $CDCl_3$) δ 154.9 (dd, $^{1}J_{C\text{-}F}$ = 297.8, 293.0 Hz, =CF_2), 131.3, 131.0, 130.7, 130.3 (dd, J = 2.5, 1.0 Hz), 129.9 (dd, J = 3.1, 1.6 Hz), 129.2, 127.7, 127.2, 127.2, 127.1, 125.2, 123.5, 122.8, 90.3 (dd, $^{2}J_{C\text{-}F}$ = 22.6, 19.4 Hz, **C**=CF_2), 29.6 (d, $^{3}J_{C\text{-}F}$ = 5.7 Hz, **C**H_2Br). ^{19}F NMR (376 MHz, $CDCl_3$) δ -82.53 (d, $^{2}J_{F\text{-}F}$ = 21.1 Hz, 1F), -84.62 (d, $^{2}J_{F\text{-}F}$ = 21.4 Hz, 1F). HRMS-EI: m/z calculated for $C_{17}H_{11}BrF_2$ [M$^+$] 332.0007, found 332.0012.

9-(1,1-difluoro-3-iodoprop-1-en-2-yl)phenanthrene (2c-I)

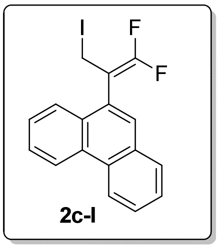

Reaction conditions: **1c** (3.0 equiv.), TMSI (1.0 equiv.), $Al(C_6F_5)_3(tol)_{0.5}$ (9.0 mol %), DCE, 80 °C. **2c-I** was purified by flash chromatography on silica gel using PE as the eluent. Red oil (39 mg, 51% yield). ^{1}H NMR (400 MHz, $CDCl_3$) δ 8.75 (d, $^{3}J_{H\text{-}H}$ = 8.0 Hz, 1H, ArH), 8.69 (d, $^{3}J_{H\text{-}H}$ = 8.2 Hz, 1H, ArH), 7.93 (d, $^{3}J_{H\text{-}H}$ = 7.4 Hz, 1H, ArH), 7.85 (d, $^{3}J_{H\text{-}H}$ = 8.0 Hz, 1H, ArH), 7.79 (s, 1H, ArH), 7.71 – 7.67 (m, 2H, ArH), 7.66 – 7.61 (m, 2H, ArH), 4.33 (d, J = 132.4 Hz, 2H, CH$_2$). ^{13}C NMR (101 MHz, $CDCl_3$) δ 153.6 (dd, $^{1}J_{C\text{-}F}$ = 297.3, 292.8 Hz, =CF_2), 131.3, 131.1, 130.7, 130.2 (dd, J = 2.6, 1.1 Hz), 129.7 (dd, J = 3.1, 1.4 Hz), 129.2, 127.7, 127.5 (dd, J = 3.7, 1.7 Hz), 127.2, 127.1, 125.2, 123.5, 122.8, 91.2 (dd, $^{2}J_{C\text{-}F}$ = 22.7, 19.8 Hz, **C**=CF_2), 1.8 (d, $^{3}J_{C\text{-}F}$ = 4.4 Hz, **C**H_2I). ^{19}F NMR (376 MHz, $CDCl_3$) δ -78.88 (d, $^{2}J_{F\text{-}F}$ = 21.4 Hz, 1F), -80.04 (d, $^{2}J_{F\text{-}F}$ = 21.5 Hz, 1F). HRMS-EI: m/z calculated for $C_{17}H_{11}IF_2$ [M$^+$] 379.9868, found 379.9872.

1-(3-bromo-1,1-difluoroprop-1-en-2-yl)naphthalene (2d)

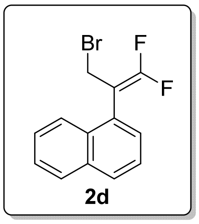

Reaction conditions: **1d** (3.0 equiv.), TMSBr (1.0 equiv.), $Al(C_6F_5)_3(tol)_{0.5}$ (9.0 mol %), DCE, 80 °C. **2d** was purified by flash chromatography on silica gel using PE as the eluent. Colorless oil (43 mg, 78% yield). ^{1}H NMR (400 MHz, $CDCl_3$) δ 7.90 – 7.88 (m, 2H, ArH), 7.81 (d, $^{3}J_{H\text{-}H}$ = 7.7 Hz, 1H, ArH), 7.56 – 7.50 (m, 4H, ArH), 4.38 (s, 2H, CH$_2$). ^{13}C NMR (101 MHz, $CDCl_3$) δ 154.6 (dd, $^{1}J_{C\text{-}F}$ = 297.5, 293.1 Hz, =CF_2), 134.0, 131.7

(dd, J = 2.5, 1.1 Hz), 129.5, 128.9, 128.7 (dd, J = 3.7, 1.8 Hz), 128.4 (dd, J = 3.3, 1.4 Hz), 126.9, 126.4, 125,5, 124.5, 90.1 (dd, $^2J_{C\text{-}F}$ = 22.6, 19.2 Hz, **C**=CF_2), 29.8 (d, $^3J_{C\text{-}F}$ = 5.9 Hz, **C**H_2Br). ^{19}F NMR (376 MHz, $CDCl_3$) δ -82.55 (d, $^2J_{F\text{-}F}$ = 21.4 Hz, 1F), -84.79 (d, $^2J_{F\text{-}F}$ = 21.3 Hz, 1F). HRMS-EI: m/z calculated for $C_{13}H_9BrF_2$ [M^+] 281.9850, found 281.9851.

4-(3-bromo-1,1-difluoroprop-1-en-2-yl)dibenzo[b,d]thiophene (2e)

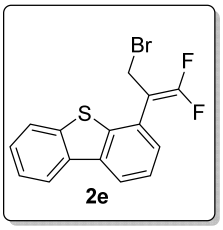

Reaction conditions: **1e** (3.0 equiv.), TMSBr (1.0 equiv.), $Al(C_6F_5)_3(tol)_{0.5}$ (9.0 mol %), DCE, 80 °C. **2e** was purified by flash chromatography on silica gel using PE as the eluent. Colorless oil (43 mg, 64% yield). ^{1}H NMR (400 MHz, $CDCl_3$) δ 8.18 – 8.14 (m, 2H, Ar**H**), 7.86 – 7.84 (m, 1H, Ar**H**), 7.54 – 7.45 (m, 4H, Ar**H**), 4.43 (t, $^4J_{H\text{-}F}$ = 1.9 Hz, 2H, C**H**$_2$). ^{13}C NMR (101 MHz, $CDCl_3$) δ 154.4 (dd, $^1J_{C\text{-}F}$ = 298.2, 295.7 Hz, =**C**F_2), 140.1 (dd, J = 3.0, 1.4 Hz), 139.1, 136.4, 135.8, 128.3 (dd, J = 2.9, 1.6 Hz), 127.3, 126.2 (dd, J = 3.9, 2.3 Hz), 125.0, 124.9, 123.0, 122.2, 122.0, 91.3 (t, $^2J_{C\text{-}F}$ = 20.9 Hz, **C**=CF_2), 27.8 (d, $^3J_{C\text{-}F}$ = 5.6 Hz, **C**H_2Br). ^{19}F NMR (376 MHz, $CDCl_3$) δ -81.67 (d, $^2J_{F\text{-}F}$ = 17.6 Hz, 1F), -84.48 (d, $^2J_{F\text{-}F}$ = 17.5 Hz, 1F). HRMS-EI: m/z calculated for $C_{15}H_9BrF_2S$ [M^+] 337.9571, found 337.9575.

4-(3-bromo-1,1-difluoroprop-1-en-2-yl)dibenzo[b,d]furan (2f)

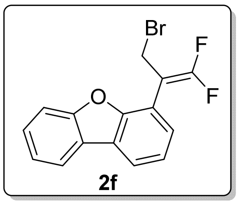

Reaction conditions: **1f** (3.0 equiv.), TMSBr (1.0 equiv.), $Al(C_6F_5)_3(tol)_{0.5}$ (9.0 mol %), DCE, 80 °C. **2f** was purified by flash chromatography on silica gel using PE as the eluent and preparative HPLC (C18 column with water/acetonitrile as the eluents). Colorless oil (47 mg, 73% yield). ^{1}H NMR (400 MHz, $CDCl_3$) δ 7.96 (d, $^3J_{H\text{-}H}$ = 7.7 Hz, 2H, Ar**H**), 7.58 (d, $^3J_{H\text{-}H}$ = 8.2 Hz, 1H, Ar**H**), 7.49 – 7.45 (m, 2H, Ar**H**), 7.34-7.40 (m, 2H, Ar**H**), 4.58 (t, $^4J_{H\text{-}F}$= 2.0 Hz, 2H, C**H**$_2$). ^{13}C NMR (101 MHz, $CDCl_3$) δ 156.2, 154.7 (t, $^1J_{C\text{-}F}$ = 299.0 Hz, =**C**F_2), 153.8, 128.2 (t, J = 2.4 Hz), 127.7, 125.0, 124.1, 123.3, 123.1, 121.3, 121.0, 115.8 (t, J = 3.0 Hz), 112.0, 88.7 (dd, $^2J_{C\text{-}F}$ = 21.6,

20.0 Hz, **C**=CF_2), 28.0 (d, $^3J_{C\text{-}F}$ = 5.5 Hz, **C**H_2Br). ^{19}F NMR (376 MHz, $CDCl_3$) δ -87.14 (d, $^2J_{F\text{-}F}$ = 18.2 Hz, 1F), -88.57 (d, $^2J_{F\text{-}F}$ = 18.2 Hz, 1F). HRMS-EI: m/z calculated for $C_{15}H_9BrF_2O$ [M^+] 321.9799, found 321.9801.

(3-(bromomethyl)-4,4-difluorobuta-1,3-diene-1,1-diyl)dibenzene (2g)

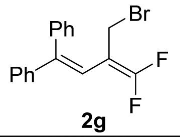

2g

Reaction conditions: **1g** (3.0 equiv.), TMSBr (1.0 equiv.), $Al(C_6F_5)_3(tol)_{0.5}$ (13.5 mol %), DCE, 60 °C. **2g** was purified by flash chromatography on silica gel with PE as the eluent. Colorless oil (37 mg, 67% yield). ^{1}H NMR (400 MHz, $CDCl_3$) δ 7.37 – 7.36 (m, 3H, Ar**H**), 7.31 – 7.25 (m, 7H, Ar**H**), 6.31 (t, J = 2.7 Hz, 1H, =C**H**), 3.70 (t, $^4J_{H\text{-}F}$ = 2.0 Hz, 2H, C**H_2**). ^{13}C NMR (101 MHz, $CDCl_3$) δ 154.9 (t, $^1J_{C\text{-}F}$ = 300. 0 Hz, =**C**F_2), 146.5 (dd, J = 6.0, 2.1 Hz), 142.4, 139.6, 129.8, 128.7, 128.4, 128.3, 128.3, 128.0, 116.5 (t, J = 2.0 Hz), 90.9 (t, $^2J_{C\text{-}F}$ = 18.5 Hz, **C**=CF_2), 26.4 (dd, $^3J_{C\text{-}F}$ = 6.2, 0.9 Hz, **C**H_2Br). ^{19}F NMR (376 MHz, $CDCl_3$) δ -80.92 (d, $^2J_{F\text{-}F}$ = 11.9 Hz, 1F), -84.75 (d, $^2J_{F\text{-}F}$ = 12.2 Hz, 1F). HRMS-EI: m/z calculated for $C_{17}H_{13}F_2^+$ [M^+ - Br] 255.0980, found [M^+ - Br] 255.0982.

1-(3-(bromomethyl)-4,4-difluorobut-3-en-1-yl)-4-chlorobenzene (2h)

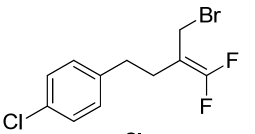

2h

Reaction conditions: **1h** (4.0 equiv.), TMSBr (1.0 equiv.), $Al(C_6F_5)_3(tol)_{0.5}$ (9.0 mol %), DCE, 60 °C. **2h** was purified by flash chromatography on silica gel using PE as the eluent and preparative HPLC (C18 column with water/acetonitrile as the eluents). Colorless oil (30 mg, 51% yield). ^{1}H NMR (400 MHz, $CDCl_3$) δ 7.27 (d, $^3J_{H\text{-}H}$ = 8.0 Hz, 2H, Ar**H**), 7.13 (d, $^3J_{H\text{-}H}$ = 8.4 Hz, 2H, Ar**H**), 3.96 (t, $^4J_{H\text{-}F}$ = 1.9 Hz, 2H, C**H_2**), 2.76 (t, $^3J_{H\text{-}H}$ = 8.0 Hz, 2H, C**H_2**), 2.43 (ddd, J = 9.7, 4.3, 2.1 Hz, 2H, C**H_2**). ^{13}C NMR (101 MHz, $CDCl_3$) δ 154.4 (dd, $^1J_{CF}$ = 292.6, 291.1 Hz, =**C**F_2), 139.1, 132.3, 129.9, 128.9, 88.3 (dd, $^2J_{C\text{-}F}$ = 22.0, 14.5 Hz, **C**=CF_2), 33.1 (t, J = 2.0 Hz), 28.0 (dd, $^3J_{C\text{-}F}$ = 7.1, 1.5 Hz), 27.4. ^{19}F NMR (376 MHz, $CDCl_3$) δ -87.77 (d, $^2J_{F\text{-}F}$ = 33.0 Hz, 1F), -88.78 (d, $^2J_{F\text{-}F}$ = 32.9 Hz, 1F). HRMS-EI: m/z calculated for $C_{11}H_{10}ClF_2^+$ [M^+ - Br] 215.0434, found [M^+ - Br] 215.0431

(4-(bromomethyl)-5,5-difluoropent-4-en-1-yl)benzene (2i)

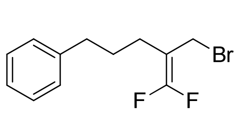

2i

Reaction conditions: **1i** (4.0 equiv.), TMSBr (1.0 equiv.), $Al(C_6F_5)_3(tol)_{0.5}$ (9.0 mol %), DCE, 60 °C. **2i** was purified by flash chromatography on silica gel using PE as the eluent and preparative HPLC (C18 column with water/acetonitrile as the eluents). Colorless oil (29 mg, 53% yield). ^{1}H NMR (400 MHz, $CDCl_3$) δ 7.31 – 7.25 (m, 2H, Ar**H**), 7.21 – 7.17 (m, 3H, Ar**H**), 4.00 (t, $^3J_{H\text{-}F}$ = 1.7 Hz, 2H, C**H**$_2$), 2.63 (t, $^3J_{H\text{-}H}$ = 8.0 Hz, 2H, C**H**$_2$), 2.21 – 2.16 (m, 2H, C**H**$_2$), 1.84 – 1.76 (m, 2H, C**H**$_2$). ^{13}C NMR (101 MHz, $CDCl_3$) δ 154.2 (t, $^1J_{C\text{-}F}$ = 291.3 Hz, =**C**F_2), 141.8, 128.6, 128.5, 126.2, 88.9 (dd, $^2J_{C\text{-}F}$ = 22.2, 13.8 Hz, **C**=CF_2), 35.5, 29.0 (t, J = 2.3 Hz), 28.0 (d, $^3J_{C\text{-}F}$ = 6.1 Hz), 25.3. ^{19}F NMR (376 MHz, $CDCl_3$) δ -88.12 (d, $^2J_{F\text{-}F}$ = 34.4 Hz, 1F), -89.36 (d, $^2J_{F\text{-}F}$ = 34.5 Hz, 1F). HRMS-EI: m/z calculated for $C_{12}H_{13}F_2^+$ [M^+ - Br] 195.0980, found [M^+ - Br] 195.0980.

1-(4-(bromomethyl)-5,5-difluoropent-4-en-1-yl)-4-methylbenzene (2j)

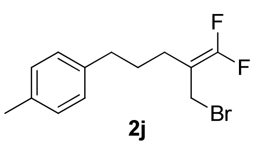

2j

Reaction conditions: **1j** (4.0 equiv.), TMSBr (1.0 equiv.), $Al(C_6F_5)_3(tol)_{0.5}$ (9.0 mol %), DCE, 60 °C. **2j** was purified by flash chromatography on silica gel using PE as the eluent and preparative HPLC (C18 column with water/acetonitrile as the eluents). Colorless oil (28 mg, 49% yield). ^{1}H NMR (400 MHz, $CDCl_3$) δ 7.09 (q, J = 7.9 Hz, 4H, Ar**H**), 4.00 (s, 2H, C**H**$_2$), 2.59 (t, $^3J_{H\text{-}H}$ = 7.8 Hz, 2H, C**H**$_2$), 2.32 (s, 3H, C**H**$_3$), 2.18 (t, $^3J_{H\text{-}H}$ = 7.1 Hz, 2H, C**H**$_2$), 1.81 – 1.74 (m, 2H, C**H**$_2$). ^{13}C NMR (101 MHz, $CDCl_3$) δ 154.2 (dd, $^1J_{C\text{-}F}$ = 292.0, 290.4 Hz, =**C**F_2), 138.7, 135.6, 129.3, 128.4, 88.9 (dd, $^2J_{C\text{-}F}$ = 22.3, 13.8 Hz, **C**=CF_2), 35.0, 29.1 (t, J = 2.4 Hz), 28.0 (dd, $^3J_{C\text{-}F}$ = 7.0, 1.9 Hz), 25.3, 21.2. ^{19}F NMR (376 MHz, $CDCl_3$) δ -87.04 (d, $^2J_{F\text{-}F}$ = 34.5 Hz, 1F), -88.28 (d, $^2J_{F\text{-}F}$ = 34.4 Hz, 1F). HRMS-EI: m/z calculated for $C_{13}H_{15}F_2^+$ [M^+ - Br] 209.1136, found [M^+ - Br] 209.1135.

(5-(bromomethyl)-6,6-difluorohex-5-en-1-yl)benzene (2k)

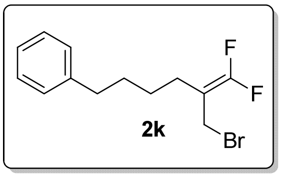

Reaction conditions: **1k** (4.0 equiv.), TMSBr (1.0 equiv.), $Al(C_6F_5)_3(tol)_{0.5}$ (9.0 mol %), DCE, 60 °C. **2k** was purified by flash chromatography on silica gel using PE as the eluent and preparative HPLC (C18 column with water/acetonitrile as the eluents). Colorless oil (28 mg, 50% yield). ^{1}H NMR (400 MHz, $CDCl_3$) δ 7.30 – 7.25 (m, 2H, Ar**H**), 7.20 – 7.16 (m, 3H, Ar**H**), 3.98 (s, 2H, C**H**$_2$), 2.63 (t, $^3J_{H-H}$ = 7.6 Hz, 2H, C**H**$_2$), 2.16 (t, $^3J_{H-H}$ = 7.1 Hz, 2H, C**H**$_2$), 1.67 – 1.60 (m, 2H, C**H**$_2$), 1.50 (dt, J = 14.9, 7.6 Hz, 2H, C**H**$_2$). ^{13}C NMR (101 MHz, $CDCl_3$) δ 154.2 (t, $^1J_{C-F}$ = 291.9 Hz, =**C**F$_2$), 142.4, 128.6, 128.6, 126.0, 89.0 (dd, $^2J_{C-F}$ = 22.3, 13.7 Hz, **C**=CF$_2$), 35.7, 31.0， 28.0 (dd, $^3J_{C-F}$ = 7.2, 1.8 Hz), 26.8 (t, J = 2.3 Hz), 25.2. ^{19}F NMR (376 MHz, $CDCl_3$) δ -88.20 (d, $^2J_{F-F}$ = 35.2 Hz, 1F), -89.55 (d, $^2J_{F-F}$ = 34.5 Hz, 1F). HRMS-EI: m/z calculated for $C_{13}H_{15}BrF_2$ [M$^+$] 288.0320, found 288.0321.

3-(bromomethyl)-2-fluoro-1H-indene (2l)

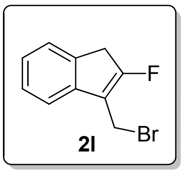

Reaction conditions: **1l** (4.0 equiv.), TMSBr (1.0 equiv.), $Al(C_6F_5)_3(tol)_{0.5}$ (9.0 mol %), DCE, 60 °C. **2l** was purified by flash chromatography on silica gel using PE as the eluent. White solid (27 mg, 59% yield). ^{1}H NMR (400 MHz, $CDCl_3$) δ 7.41 – 7.40 (m, 1H, Ar**H**), 7.36 – 7.31 (m, 2H, Ar**H**), 7.25 – 7.20 (m, 1H, Ar**H**), 4.40 (s, 2H, C**H**$_2$), 3.53 (s, 2H, C**H**$_2$). ^{13}C NMR (101 MHz, $CDCl_3$) δ 164.5 (d, $^1J_{C-F}$ = 288.7 Hz, =**C**F), 140.5 (d, J = 4.8 Hz) 134.5 (d, J = 8.0 Hz), 127.1, 125.2 (d, J = 4.1 Hz), 123.9, 119.6 (d, J = 6.5 Hz), 115.5 (d, J = 9.2 Hz), 35.2 (d, $^2J_{C-F}$ = 19.6 Hz, **C**H$_2$CF=), 19.9. ^{19}F NMR (376 MHz, $CDCl_3$) δ -119.36 (s, 1F). HRMS-EI: m/z calculated for $C_{10}H_8BrF$ [M$^+$] 225.9788, found 225.9786.

4-(bromomethyl)-3-fluoro-1,2-dihydronaphthalene (2m)

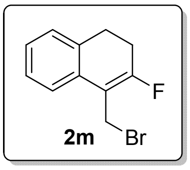

Reaction conditions: **1m** (4.0 equiv.), TMSBr (1.0 equiv.), $Al(C_6F_5)_3(tol)_{0.5}$ (9.0 mol %), DCE, 60 °C. **2m** was purified by flash chromatography on silica gel using PE as the eluent. White solid (42 mg, 88 % yield). ^{1}H NMR (400 MHz, $CDCl_3$) δ 7.37 (d, $^3J_{H-H}$ = 7.7 Hz, 1H, Ar**H**), 7.28 – 7.24 (m, 1H, Ar**H**), 7.18 – 7.12 (m, 2H, Ar**H**), 4.44 (s, 2H, C**H**$_2$), 2.98 (t, $^3J_{H-H}$ = 7.5 Hz, 2H, C**H**$_2$), 2.62 – 2.57 (m, 2H, C**H**$_2$). ^{13}C NMR (101 MHz, $CDCl_3$) δ 161.7 (d, $^1J_{C-F}$ = 276.6 Hz, =**C**F), 132.8, 131.8 (d, J = 4.6 Hz), 127.8, 127.1, 126.9 (d, J = 1.6 Hz), 123.2 (d, J = 6.7 Hz), 112.3 (d, $^3J_{C-F}$ = 11.1 Hz), 28.2 (d, J = 7.3 Hz), 25.3 (d, $^2J_{C-F}$ = 23.2 Hz, **C**H$_2$CF=), 23.0 (d, J = 8.6 Hz). ^{19}F NMR (376 MHz, $CDCl_3$) δ -101.54 (s, 1F). HRMS-EI: m/z calculated for $C_{11}H_{10}BrF$ [M^+] 239.9944, found 239.9943

9-(bromomethyl)-8-fluoro-6,7-dihydro-5H-benzo[7]annulene (2n)

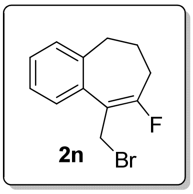

Reaction conditions: **1n** (4.0 equiv.), TMSBr (1.0 equiv.), $Al(C_6F_5)_3(tol)_{0.5}$ (9.0 mol %), DCE, 60 °C. **2n** was purified by flash chromatography on silica gel using PE as the eluent. White solid (38 mg, 75% yield). ^{1}H NMR (400 MHz, $CDCl_3$) δ 7.45 (d, $^3J_{H-H}$ = 7.6 Hz, 1H, Ar**H**), 7.31 – 7.27 (m, 1H, Ar**H**), 7.24 – 7.20 (m, 2H, Ar**H**), 4.45 (d, $^3J_{H-F}$ = 2.9 Hz, 2H, C**H**$_2$), 2.70 (t, $^3J_{H-H}$ = 6.7 Hz, 2H, C**H**$_2$), 2.28 – 2.22 (m, 4H, C**H**$_2$). ^{13}C NMR (101 MHz, $CDCl_3$) δ 162.9 (d, $^1J_{C-F}$ = 272.2 Hz, =**C**F), 141.0, 135.9 (d, J = 7.4 Hz), 129.5, 127.7, 126.8, 126.7 (d, J = 4.9 Hz), 114.8 (d, J = 14.3 Hz), 32.8, 32.1, 28.2 (d, J = 9.1 Hz), 28.0 (d, $^2J_{C-F}$ = 27.3 Hz, **C**H$_2$CF=). ^{19}F NMR (376 MHz, $CDCl_3$) δ -89.07 (t, $^3J_{F-H}$ = 18.0 Hz, 1F). HRMS-EI: m/z calculated for $C_{12}H_{12}BrF$ [M^+] 254.0101, found 254.0102.

(*E*)-4-(3-bromo-1-fluoroprop-1-en-2-yl)-1,1'-biphenyl (2o)

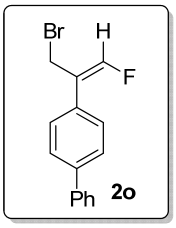

Reaction conditions: **1o** (4.0 equiv.), TMSBr (1.0 equiv.), $Al(C_6F_5)_3(tol)_{0.5}$ (9.0 mol %), DCE, 60 °C. **2o** was purified by flash chromatography on silica gel using PE as the eluent. Colorless oil (27 mg, 50% yield). ^{1}H NMR (400 MHz, $CDCl_3$) δ 7.66 – 7.59 (m, 6H, Ar**H**), 7.45 (t, *J* = 7.6 Hz, 2H, Ar**H**), 7.36 (t, *J* = 7.3 Hz, 1H, Ar**H**), 7.04 (d, $^2J_{H\text{-}F}$ = 80.8 Hz, 1H, =C**H**), 4.28 (d, $^2J_{H\text{-}F}$ = 4.0 Hz, 2H, C**H**$_2$). ^{13}C NMR (101 MHz, $CDCl_3$) δ 148.2 (d, $^1J_{C\text{-}F}$ = 274.5 Hz, =**C**F), 141.3, 140.7, 131.2 (d, *J* = 1.6 Hz), 129.0, 128.7 (d, *J* = 5.2 Hz), 127.8, 127.4, 127.3, 120.5 (d, *J* = 4.9 Hz), 31.1 (d, $^3J_{C\text{-}F}$ = 11.6 Hz, **C**H$_2$Br). ^{19}F NMR (376 MHz, $CDCl_3$) δ -130.23 (dt, *J* = 80.8, 3.8 Hz, 1F). HRMS-EI: m/z calculated for $C_{15}H_{12}BrF$ [M^+] 290.0101, found 290.0105.

5. Disubstitution Reactions

General procedure: In a glovebox, substrate **1** (0.2 mmol, 1.0 equiv.), TMSBr (122.4 mg, 0.8 mmol, 4.0 equiv.) and $Al(C_6F_5)_3(tol)_{0.5}$ (5.2 mg, 0.009 mmol, 4.5 mol %) were dissolved in DCE (1.5 mL) in an 8 mL vial containing a magnetic stir bar. The vial was sealed. The mixture was stirred at 120 °C in a heating block for 24 h. After being allowed to cool to room temperature, the solution was concentrated in vacuo. The residue was purified by flash chromatography on silica gel using PE as the eluent to give the desired product. The analytical data for these products are listed as follows.

(*Z*/*E*)-4-(1,3-dibromo-1-fluoroprop-1-en-2-yl)-1,1'-biphenyl (3a, *Z*/*E* = 55:45)

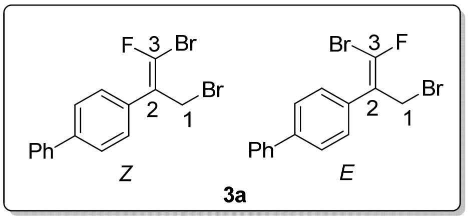

3a was purified by flash chromatography on silica gel using PE as the eluent. White foam (62 mg, 84% yield). ^{1}H NMR (400 MHz, $CDCl_3$) (*E*+*Z*) δ 7.63 – 7.59 (m, 4H, Ar**H**), 7.51 – 7.41 (m, 4H, Ar**H**), 7.38 – 7.34 (m, 1H, Ar**H**), 4.35 – 4.33 (m, 2H, C**H**$_2$). ^{13}C NMR (101 MHz, $CDCl_3$) (*E*+*Z*) δ 141.6, 141.6, 140.5, 140.5, 137.2 (d, $^1J_{C\text{-}F}$ = 326.2 Hz, C_3), 136.6 (d, $^1J_{C\text{-}F}$ = 327.2 Hz, C_3), 134.4 (d, *J* = 3.4 Hz), 132.3 (d, *J* = 1.0 Hz), 129.5 (d, *J* = 3.2 Hz), 129.1, 129.0, 128.7 (d, *J* = 3.8 Hz), 127.8, 127.5, 127.5, 127.3, 127.3, 121.1 (d, $^2J_{C\text{-}F}$ = 13.1 Hz, C_2), 120.1 (d, $^2J_{C\text{-}F}$ = 12.8 Hz, *C*$_2$), 34.1 (d, $^3J_{C\text{-}F}$ = 4.0 Hz, *Z*, C_1), 28.7

(d, $^3J_{C\text{-}F}$ = 5.8 Hz, *E*, C_1). ^{19}F NMR (376 MHz, $CDCl_3$) δ -66.73 (s, *Z*, 1F), -67.13 (t, $^4J_{F\text{-}H}$= 3.2 Hz, *E*, 1F). HRMS-EI: m/z calculated for $C_{15}H_{11}Br_2F$ [M^+] 367.9206, found 367.9208.

(*Z*/*E*)-1-(1,3-dibromo-1-fluoroprop-1-en-2-yl)-4-methoxybenzene (3p, *Z*/*E* = 57:43)

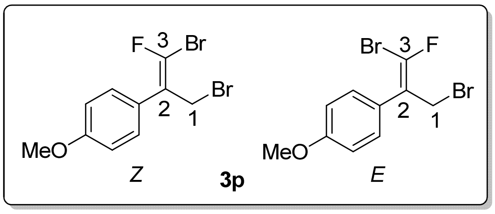

3p was purified by flash chromatography on silica gel using PE/EA (20:1) as the eluents. Colorless oil (46 mg, 71% yield). ^{1}H NMR (400 MHz, $CDCl_3$) (*E*+*Z*) δ 7.35 (d, $^3J_{H\text{-}H}$ = 8.5 Hz, 1H, Ar**H**), 7.27 (d, $^3J_{H\text{-}H}$ = 8.6 Hz, 1H, Ar**H**), 6.98 – 6.91 (m, 2H, Ar**H**), 4.30 – 4.28 (m, 2H, C**H**$_2$), 3.85 – 3.82 (m, 3H, OC**H**$_3$). ^{13}C NMR (101 MHz, $CDCl_3$) (*E*+*Z*) δ 160.0, 159.8, 136.4 (d, $^1J_{C\text{-}F}$ = 325.8 Hz, C_3), 130.3, 130.3, 129.6, 129.5, 129.4 (d, $^1J_{C\text{-}F}$ = 331.28 Hz, C_3), 125.6, 121.0 (d, $^2J_{C\text{-}F}$ = 12.7 Hz, C_2), 120.0 (d, $^2J_{C\text{-}F}$ = 13.0 Hz, C_2), 114.3, 114.2, 55.5, 34.4 (d, $^3J_{C\text{-}F}$ = 3.9 Hz, *Z*, C_1), 29.0 (d, $^3J_{C\text{-}F}$ = 5.6 Hz, *E*, C_1). ^{19}F NMR (376 MHz, $CDCl_3$) δ -67.79 (t, $^4J_{F\text{-}H}$ = 3.2 Hz, *E*, 1F), -68.16 (s, *Z*, 1F). HRMS-EI: m/z calculated for $C_{10}H_9Br_2FO$ [M^+] 321.8999, found 321.9001.

(*Z*/*E*)-methyl 4-(1,3-dibromo-1-fluoroprop-1-en-2-yl)benzoate (3q, *Z*/*E* = 50:50)

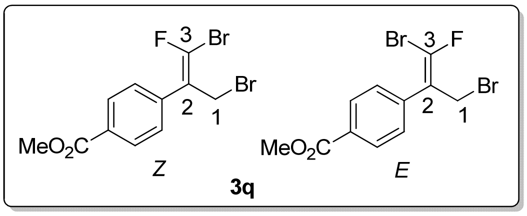

Reaction conditions: **1q** (1.0 equiv.), TMSBr (5.0 equiv.), $Al(C_6F_5)_3(tol)_{0.5}$ (4.5 mol %), DCE, 120 °C. **3q** was purified by flash chromatography on silica gel using PE/EA (10:1) as the eluents. Colorless oil (48 mg, 69% yield). ^{1}H NMR (400 MHz, $CDCl_3$) (*E*+*Z*) δ 8.09 – 8.06 (m, 2H, Ar**H**), 7.51 (d, $^3J_{H\text{-}H}$ = 8.2 Hz, 1H, Ar**H**), 7.44 (d, $^3J_{H\text{-}H}$ = 8.1 Hz, 1H, Ar**H**), 4.32 – 4.31 (m, 2H, C**H**$_2$), 3.93 (s, 3H, C**H**$_3$). ^{13}C NMR (101 MHz, $CDCl_3$)(*E*+*Z*) δ 166.5, 166.5, 140.0, 138.3, 137.8 (d, $^1J_{C\text{-}F}$ = 327.7 Hz, C_3), 136.7 (d, $^1J_{C\text{-}F}$ = 327.9 Hz, C_3), 130.4, 130.1, 129.9, 129.1, 129.0, 128.2, 128.1, 120.6 (d, $^2J_{C\text{-}F}$ = 13.7 Hz, C_2), 119.7 (d, $^2J_{C\text{-}F}$ = 12.8 Hz, C_2), 52.3, 33.2 (d, $^3J_{C\text{-}F}$ = 3.5 Hz, *Z*, C_1), 27.8 (d, $^3J_{C\text{-}F}$ = 5.6 Hz, *E*, C_1). ^{19}F NMR (376 MHz, $CDCl_3$) δ -65.22 (s, *Z*, 1F), -66.37 (t, $^4J_{F\text{-}}$

$_H$ = 3.1 Hz, *E*, 1F). HRMS-EI: m/z calculated for $C_{11}H_9Br_2FO_2$ [M$^+$] 349.8948, found 349.8947.

(*Z*/*E*)-1-(1,3-dibromo-1-fluoroprop-1-en-2-yl)-4-(methylsulfonyl)benzene (3r, *Z*/*E* = 47:53)

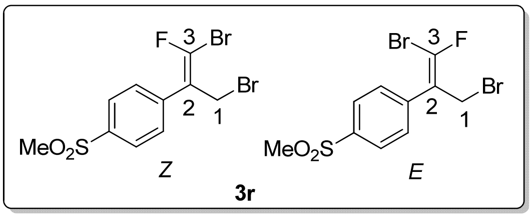

Reaction conditions: **1r** (1.0 equiv.), TMSBr (5.0 eq.), $Al(C_6F_5)_3(tol)_{0.5}$ (4.5 mol %), DCE, 120 °C. **3r** was purified by flash chromatography on silica gel using PE/EA (4:1) as the eluents. White foam (63 mg, 85% yield). ^{1}H NMR (400 MHz, $CDCl_3$)(*E*+*Z*) δ 8.00 – 7.97 (m, 2H, Ar**H**), 7.64 (d, *J* = 8.0 Hz, 1H, Ar**H**), 7.58 (d, *J* = 8.4 Hz, 1H, Ar**H**), 4.32 – 4.31 (m, 2H, C**H**$_2$), 3.10 – 3.09 (m, 3H, C**H**$_3$). ^{13}C NMR (101 MHz, $CDCl_3$)(*E*+*Z*) δ 141.2, 141.2, 140.9, 140.6, 139.1, 138.6 (d, $^1J_{C\text{-}F}$ = 328.3 Hz, C_3), 137.3 (d, $^1J_{C\text{-}F}$ = 329.9 Hz, C_3), 130.3, 130.3, 129.3, 129.3, 128.0, 128.0, 120.2 (d, $^2J_{C\text{-}F}$ = 14.5 Hz, C_2), 119.2 (d, $^2J_{C\text{-}F}$ = 12.8 Hz, C_2), 44.6, 44.6, 33.0 (d, $^3J_{C\text{-}F}$ = 3.5 Hz, *Z*, C_1), 27.6 (d, $^3J_{C\text{-}F}$ = 5.7 Hz, *E*, C_1). ^{19}F NMR (376 MHz, $CDCl_3$) δ -64.51 (s, *Z*, 1F), -65.38 (t, $^4J_{F\text{-}H}$ = 3.0 Hz, *E*, 1F). HRMS-EI: m/z calculated for $C_{10}H_9Br_2FO_2S$ [M$^+$] 369.8669, found 369.8669.

(*Z*/*E*)-1-(1,3-dibromo-1-fluoroprop-1-en-2-yl)naphthalene (3d, *Z*/*E* = 40:60)

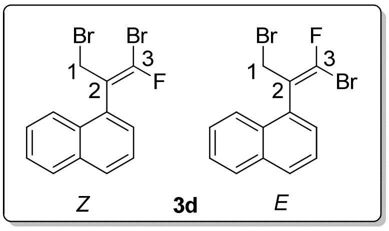

Reaction conditions: **1d** (1.0 equiv.), TMSBr (5.0 equiv.), $Al(C_6F_5)_3(tol)_{0.5}$ (4.5 mol %), DCE, 120 °C. **3d** was purified by flash chromatography on silica gel using PE as the eluent. Colorless oil (55 mg, 80% yield). ^{1}H NMR (400 MHz, $CDCl_3$) (*E*+*Z*) δ 7.90 – 7.87 (m, 2H, Ar**H**), 7.80 – 7.75 (m, 1H, Ar**H**), 7.55 – 7.45 (m, 4H, Ar**H**), 4.62 – 4.18 (m, 2H, C**H**$_2$). ^{13}C NMR (101 MHz, $CDCl_3$) (*E*+*Z*) δ 138.0 (d, $^1J_{C\text{-}F}$ = 329.3 Hz, C_3), 136.5 (d, $^1J_{C\text{-}F}$ = 323.2 Hz, C_3), 134.0 (d, *J* = 3.1 Hz), 132.8 (d, *J* = 3.1 Hz), 131.0 (d, *J* = 11.4 Hz), 130.9 (d, *J* = 2.8 Hz), 129.5, 128.9, 128.9, 128.2, 128.2, 127.3, 127.3, 127.0, 126.9, 126.4, 125.6, 125.5, 124.7,

124.6, 119.5 (d, $^2J_{C-F}$ = 12.7 Hz, C_2), 119.2 (d, $^2J_{C-F}$ = 16.8 Hz, C_2), 34.8 (d, $^3J_{C-F}$ = 3.8 Hz, *Z*, C_1), 28.9 (d, $^3J_{C-F}$ = 4.7 Hz, *E*, C_1). ^{19}F NMR (376 MHz, $CDCl_3$) δ -63.43 (s, *Z*, 1F), -67.12 (t, $^4J_{F-H}$ = 2.9 Hz, *E*, 1F). HRMS-EI: m/z calculated for $C_{13}H_9Br_2F$ [M^+] 341.9050, found 341.9050.

(*Z*/*E*)-4-(1,3-dibromo-1-fluoroprop-1-en-2-yl)dibenzo[b,d]thiophene (3e, *Z*/*E* = 36/64)

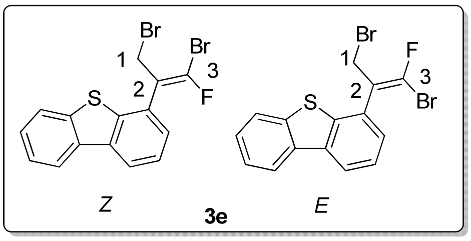

3e was purified by flash chromatography on silica gel using PE as the eluent. Colorless oil (64 mg, 80% yield). ^{1}H NMR (400 MHz, $CDCl_3$) (*E*+*Z*) δ 8.19 – 8.14 (m, 2H, Ar**H**), 7.87 – 7.82 (m, 1H, Ar**H**), 7.54 – 7.43 (m, 4H, Ar**H**), 4.43 (m, 2H, C**H**$_2$). ^{13}C NMR (101 MHz, $CDCl_3$) (*E*+*Z*) δ 139.5 (d, *J* = 10.10 Hz), 139.4 (d, *J* = 13.13 Hz), 139.1, 137.8 (d, $^1J_{C-F}$ = 277.75 Hz, C_3), 137.7 (d, $^1J_{C-F}$ = 284.82 Hz, C_3), 135.8, 135.8, 130.2, 130.1, 128.4, 128.2, 128.2, 127.4, 127.4, 127.4, 125.0 (d, *J* = 11.3 Hz), 124.9, 123.0 (d, *J* = 8.5 Hz), 122.1, 122.0, 120.1 (d, $^2J_{C-F}$ = 14.3 Hz, C_2), 119.7 (d, $^2J_{C-F}$ = 15.7 Hz, C_2), 33.0 (d, $^3J_{C-F}$ = 3.7 Hz, *Z*, C_1), 27.3 (d, $^3J_{C-F}$ = 4.6 Hz, *E*, C_1). ^{19}F NMR (376 MHz, $CDCl_3$) δ -61.46 (s, *Z*, 1F), -65.99 (t, $^4J_{F-H}$ = 2.9 Hz, *E*, 1F). HRMS-EI: m/z calculated for $C_{15}H_9Br_2FS$ [M^+] 397.8770, found 397.8779.

(*Z*/*E*)-4-(1,3-dibromo-1-fluoroprop-1-en-2-yl)dibenzo[b,d]furan (3f, *Z*/*E* = 44/56)

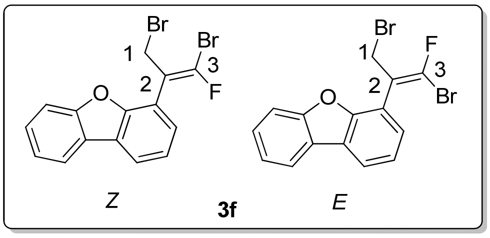

3f was purified by flash chromatography on silica gel using PE as the eluent. Colorless oil (47 mg, 62% yield). ^{1}H NMR (400 MHz, $CDCl_3$) (*E*+*Z*) δ 8.03 – 7.96 (m, 2H, Ar**H**), 7.60 – 7.57 (m, 1H, Ar**H**), 7.50 – 7.45 (m, 2H, Ar**H**), 7.41 – 7.34 (m, 2H, Ar**H**), 4.58 – 4.55 (m, 2H, C**H**$_2$). ^{13}C NMR (101 MHz, $CDCl_3$) (*E*+*Z*) δ 156.2, 156.2, 153.4 (d, *J* = 3.0 Hz), 153.0 (d, *J* = 1.1 Hz), 137.7 (d, $^1J_{C-F}$ = 328.3 Hz, C_3), 137.5 (d, $^1J_{C-F}$ = 326.2 Hz, C_3), 129.0 (d, *J* = 2.7 Hz), 128.1 (d, *J* = 2.9 Hz), 127.8, 127.8, 125.1 (d, *J* = 4.8 Hz),

124.1 (d, J = 5.6 Hz), 123.3 (d, J = 2.2 Hz), 123.1 (d, J = 2.2 Hz), 121.6, 121.5, 121.1, 121.0, 119.5, 119.4, 117.5 (d, $^2J_{C\text{-}F}$ = 16.2 Hz, *E*, C_2), 117.5, 116.7 (d, $^2J_{C\text{-}F}$ = 15.1 Hz, *Z*, C_2), 112.0, 112.0, 33.3 (d, $^3J_{C\text{-}F}$ = 3.6 Hz, *Z*, C_1), 27.8 (d, $^3J_{C\text{-}F}$ = 5.3 Hz, *E*, C_1). ^{19}F NMR (376 MHz, $CDCl_3$) δ -64.05 (s, *Z*, 1F), -66.51 (t, $^4J_{F\text{-}H}$= 3.1 Hz, *E*, 1F). HRMS-EI: m/z calculated for $C_{15}H_9Br_2FO$ [M^+] 381.8999, found 381.9003.

(*Z*/*E*)-1-(4-bromo-3-(bromomethyl)-4-fluorobut-3-en-1-yl)-4-chlorobenzene (3h, *Z*/*E* = 50:50)

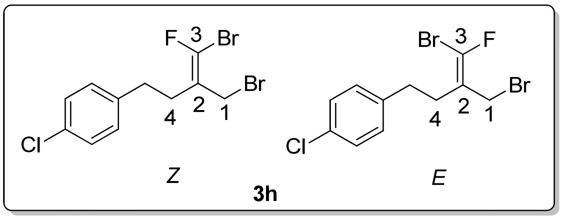

3h was purified by flash chromatography on silica gel using PE as the eluent. Colorless oil (50 mg, 71% yield). ^{1}H NMR (400 MHz, $CDCl_3$) (*E*+*Z*) δ 7.28 – 7.26 (m, 2H, Ar**H**), 7.15 – 7.11 (m, 2H, Ar**H**), 4.01 – 3.95 (m, 2H, C$\mathbf{H}_2$), 2.80 – 2.76 (m, 2H, C$\mathbf{H}_2$), 2.61 – 2.57 (m, 1H, C$\mathbf{H}_2$), 2.50 – 2.45 (m, 1H, C$\mathbf{H}_2$). ^{13}C NMR (101 MHz, $CDCl_3$) (*E*+*Z*) δ 139.1, 139.0, 135.8 (d, $^1J_{C\text{-}F}$ = 323.2 Hz, C_3), 135.0 (d, $^1J_{C\text{-}F}$ = 320.2 Hz, C_3), 132.4, 132.3, 130.0, 129.9, 128.9, 128.9, 118.7 (d, $^2J_{C\text{-}F}$ = 16.1 Hz, C_2), 117.8 (d, $^2J_{C\text{-}F}$ = 8.1 Hz, C_2), 33.4 (d, J = 2.4 Hz,), 33.1 (d, $^3J_{C\text{-}F}$ = 3.2 Hz, *Z*, C_1), 33.1 (d, J = 2.4 Hz,), 32.8 (d, $^3J_{C\text{-}F}$ = 3.6 Hz, *Z*, C_4), 30.1 (d, $^3J_{C\text{-}F}$ = 2.9 Hz, *E*, C_4), 27.0 (d, $^3J_{C\text{-}F}$ = 7.8 Hz, *E*, C_1). ^{19}F NMR (376 MHz, $CDCl_3$) δ -74.31(s, 1F), -74.42 (s, 1F). HRMS-EI: m/z calculated for $C_{11}H_{10}BrF_2^+$ [M^+ - Br] 274.9633, found [M^+ - Br] 274.9635.

(*Z*/*E*)-1-(4-bromo-3-(bromomethyl)-4-fluorobut-3-en-1-yl)-4-fluorobenzene (3s, *Z*/*E* = 50:50)

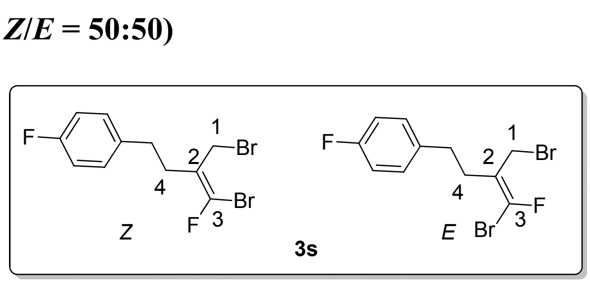

3s was purified by flash chromatography on silica gel using PE as the eluent. Colorless oil (35 mg, 50% yield). ^{1}H NMR (400 MHz, $CDCl_3$) (*E*+*Z*) δ 7.18 – 7.13 (m, 2H, Ar**H**), 7.01 – 6.97 (m, 2H, Ar**H**), 4.01 – 3.95 (m, 2H, C$\mathbf{H}_2$),

2.80 – 2.76 (m, 2H, C**H**$_2$), 2.61 – 2.57 (m, 1H, C**H**$_2$), 2.49 – 2.45 (m, 1H, C**H**$_2$). ^{13}C NMR (101 MHz, CDCl$_3$) (*E*+*Z*) δ 163.0 (d, *J* = 2.3 Hz), 160.6 (d, *J* = 2.3 Hz), 136.3 (d, *J* = 3.3 Hz), 136.2 (d, *J* = 3.3 Hz), 135.8 (d, $^1J_{C\text{-}F}$ = 322.2 Hz, C$_3$), 134.9 (d, $^1J_{C\text{-}F}$ = 321.18 Hz, C$_3$), 130.0 (t, *J* = 7.8 Hz), 118.8 (d, $^2J_{C\text{-}F}$ = 16.1 Hz, C$_2$), 117.9 (d, $^2J_{C\text{-}F}$ = 7.8 Hz, C$_2$), 115.7 (d, *J* = 2.2 Hz), 115.5 (d, *J* = 1.9 Hz). 33.3, 33.2 (d, $^3J_{C\text{-}F}$ = 2.0 Hz,), 33.1 (d, $^3J_{C\text{-}F}$ = 5.5 Hz, *Z*, C$_4$), 32.7 (d, $^3J_{C\text{-}F}$ = 3.5 Hz, *Z*, C$_1$), 30.3 (d, $^3J_{C\text{-}F}$ = 2.5 Hz, *E*, C$_4$), 27.0 (d, $^3J_{C\text{-}F}$ = 7.9 Hz, *E*, C$_1$). ^{19}F NMR (376 MHz, CDCl$_3$) δ -66.46 (s, 1F), -66.59 (s, 1F), -113.42 – 113.54 (m). HRMS-EI: m/z calculated for $C_{11}H_{10}BrF_2^+$ [M$^+$ - Br] 258.9928, found [M$^+$ - Br] 258.9931.

(*Z*/*E*)-(5-bromo-4-(bromomethyl)-5-fluoropent-4-en-1-yl)benzene (3i, *Z*/*E* = 50:50)

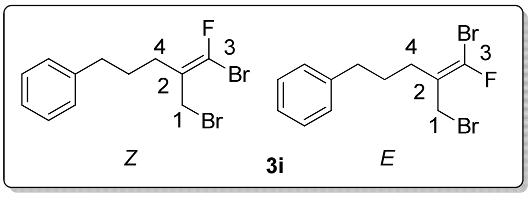

3i was purified by flash chromatography on silica gel using PE as the eluent. Colorless oil (56 mg, 79% yield).^{1}H NMR (400 MHz, CDCl$_3$) (*E*+*Z*) δ 7.30 – 7.25 (m, 2H, Ar**H**), 7.20 – 7.17 (m, 3H, Ar**H**), 4.06 – 4.00 (m, 2H, C**H**$_2$), 2.67 – 2.62 (m, 2H, C**H**$_2$), 2.39 – 2.35 (m, 1H, C**H**$_2$), 2.26 – 2.23 (m, 1H, C**H**$_2$), 1.87 – 1.79 (m, 2H, C**H**$_2$). ^{13}C NMR (101 MHz, CDCl$_3$) (*E*+*Z*) δ 141.7, 135.2 (d, $^1J_{C\text{-}F}$ = 322.2 Hz, C$_3$), 134.6 (d, $^1J_{C\text{-}F}$ = 320.2 Hz, C$_3$), 128.6, 128.6, 126.2, 119.4 (d, $^2J_{C\text{-}F}$ = 16.1 Hz, C$_2$), 118.6 (d, $^2J_{C\text{-}F}$ = 7.3 Hz, C$_2$), 35.6 (d, $^3J_{C\text{-}F}$ = 3.9 Hz, *Z*, C$_1$), 33.0 (d, $^3J_{C\text{-}F}$ = 5.5 Hz, *Z*, C$_4$), 31.1, 29.3 (d, *J* = 1.7 Hz), 28.9 (d, *J* = 3.0 Hz), 28.0 (d, $^3J_{C\text{-}F}$ = 3.0 Hz, *E*, C$_4$), 26.8 (d, $^3J_{C\text{-}F}$ = 7.8 Hz, *E*, C$_1$). ^{19}F NMR (376 MHz, CDCl$_3$) δ -70.43 (s, 1F), -70.66 (s, 1F). HRMS-EI: m/z calculated for $C_{12}H_{13}BrF^+$ [M$^+$ - Br] 255.0179, found [M$^+$ - Br] 255.0180.

(*Z*/*E*)-1-(5-bromo-4-(bromomethyl)-5-fluoropent-4-en-1-yl)-4-methylbenzene (3j, *Z*/*E* = 53:47)

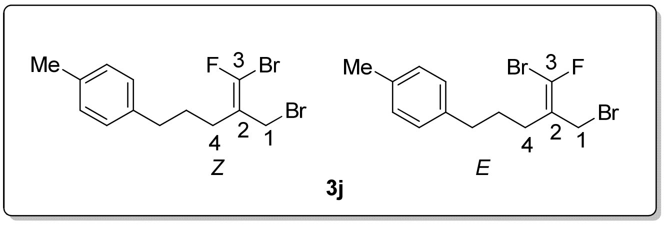

3j was purified by flash chromatography on silica gel using PE as the eluent. Colorless oil (50 mg, 72% yield). ^{1}H NMR (400 MHz, $CDCl_3$) (*E*+*Z*) δ 7.11 – 7.06 (m, 4H, Ar**H**), 4.07 – 4.00 (m, 2H, C**H**$_2$), 2.63 – 2.57 (m, 2H, C**H**$_2$), 2.38 – 2.34 (m, 1H, C**H**$_2$), 2.32 (s, 3H, C**H**$_3$), 2.26 – 2.22 (m, 1H, C**H**$_2$), 1.84 – 1.76 (m, 2H, C**H**$_2$). ^{13}C NMR (101 MHz, $CDCl_3$) (*E*+*Z*) δ 138.6, 135.2 (d, $^{1}J_{C\text{-}F}$ = 321.2 Hz, C_3), 135.7, 135.6, 134.5 (d, $^{1}J_{C\text{-}F}$ = 320.2 Hz, C_3), 129.3, 128.4, 119.4 (d, $^{2}J_{C\text{-}F}$ = 16.0 Hz, C_2), 118.6 (d, $^{2}J_{C\text{-}F}$ = 7.5 Hz, C_2), 35.2 (d, $^{3}J_{C\text{-}F}$ = 5.2 Hz, *Z*, C_1), 33.0 (d, $^{3}J_{C\text{-}F}$ = 5.6 Hz, *Z*, C_4), 31.0, 29.4 (d, J = 2.2 Hz), 29.0 (d, J = 3.2 Hz), 28.0 (d, $^{3}J_{C\text{-}F}$ = 3.2 Hz, *E*, C_4), 26.8 (d, $^{3}J_{C\text{-}F}$ = 7.9 Hz, *E*, C_1), 21.2. ^{19}F NMR (376 MHz, $CDCl_3$) δ -70.28 (s, 1F), -70.50 (s, 1F). HRMS-EI: m/z calculated for $C_{13}H_{15}BrF^{+}$ [M^{+} - Br] 269.0336, found [M^{+} - Br] 269.0338.

(*Z*/*E*)-1-(5-bromo-4-(bromomethyl)-5-fluoropent-4-en-1-yl)-4-methoxybenzene (3t, *Z*/*E* = 42:58)

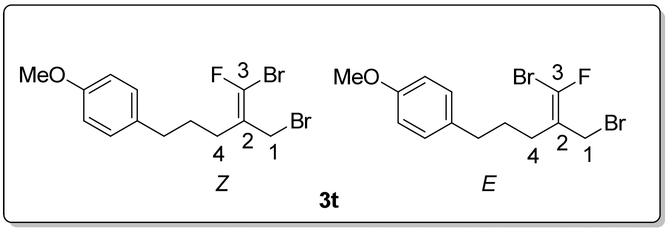

3t was purified by flash chromatography on silica gel using PE/EA (20:1) as the eluents. Colorless oil (32 mg, 44% yield). ^{1}H NMR (400 MHz, $CDCl_3$) (*E*+*Z*) δ 7.12 – 7.08 (m, 2H, Ar**H**), 6.84 – 6.82 (m, 2H, Ar**H**), 4.06 – 3.99 (m, 2H, C**H**$_2$), 3.79 (s, 3H, OC**H**$_3$), 2.58 (dd, J = 15.6, 7.9 Hz, 2H, C**H**$_2$), 2.37 – 2.32 (m, 1H, C**H**$_2$), 2.25 – 2.20 (m, 1H, C**H**$_2$), 1.78 (dq, J = 15.5, 7.8 Hz, 2H, C**H**$_2$). ^{13}C NMR (101 MHz, $CDCl_3$) (*E*+*Z*) δ 158.0, 135.1 (d, $^{1}J_{C\text{-}F}$ = 321.2 Hz, C_3), 134.5 (d, $^{1}J_{C\text{-}F}$ = 326.2 Hz, C_3), 133.7, 129.4, 119.4 (d, $^{2}J_{C\text{-}F}$ = 15.9 Hz, C_2), 118.6 (d, $^{2}J_{C\text{-}F}$ = 7.5 Hz, C_2), 114.0, 55.4, 34.7 (d, $^{3}J_{C\text{-}F}$ = 4.5 Hz, *Z*, C_1), 33.0 (d, $^{3}J_{C\text{-}F}$ = 5.6 Hz, *Z*, C_4), 30.9, 29.5 (d, J = 2.2 Hz), 29.1 (d, J = 3.2 Hz), 27.9 (d, $^{3}J_{C\text{-}F}$ = 3.3 Hz, *E*,

C4), 26.8 (d, $^{3}J_{C-F}$ = 8.0 Hz, *E*, C1). ^{19}F NMR (376 MHz, $CDCl_3$) δ -69.73 (s, 1F), -69.95 (s, 1F). HRMS-EI: m/z calculated for $C_{13}H_{15}BrFO^+$ [M^+ - Br] 285.0285, found [M^+ - Br] 285.0286.

(*Z*/*E*)-1-bromo-2-(bromomethyl)-1-fluorodec-1-en-3-yne (3u, *Z*/*E* = 30:70)

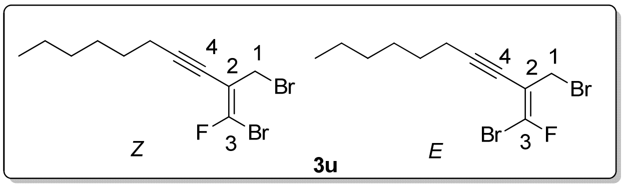

3u was purified by flash chromatography on silica gel using PE as the eluent. Colorless oil (58 mg, 89% yield). ^{1}H NMR (400 MHz, $CDCl_3$) (*E*+*Z*) δ 4.04 – 4.00 (m, 2H, C**H**2), 2.37 (td, *J* = 7.0, 1.5 Hz, 2H, C**H**2), 1.61 – 1.53 (m, 2H, C**H**2), 1.48 – 1.40 (m, 2H, C**H**2), 1.35 – 1.26 (m, 4H, C**H**2), 0.90 (d, J = 8.0 Hz, 3H, C**H**3). ^{13}C NMR (101 MHz, $CDCl_3$) (*E*+*Z*) δ 142.6 (d, $^{1}J_{C-F}$ = 329.3 Hz, *Z*, C3), 141.3 (d, $^{1}J_{C-F}$ = 330.3 Hz, *E*, C3), 108.3 (d, $^{2}J_{C-F}$ = 25.3 Hz, *E*, C2), 106.1 (d, $^{2}J_{C-F}$ = 16.2 Hz, *Z*, C2), 99.7 (d, $^{3}J_{C-F}$ = 7.1 Hz, *Z*, C4), 98.4 (d, $^{3}J_{C-F}$ = 8.1 Hz, *E*, C4), 74.8 (d, *J* = 5.1 Hz), 71.9, 31.9 (*Z*, C1), 31.5, 28.6, 28.4, 26.6 (d, $^{3}J_{C-F}$ = 2.0 Hz, *E*, C1), 22.8, 22.7, 19.8, 14.3, 14.2. ^{19}F NMR (376 MHz, $CDCl_3$) δ -60.11 (s, *Z*, 1F), -67.19 (s, *E*, 1F). HRMS-EI: m/z calculated for $C_{11}H_{15}Br_2F$ [M^+] 323.9519, found 323.9513.

(*Z*/*E*)-(4-bromo-3-(bromomethyl)-4-fluorobut-3-en-1-yn-1-yl)benzene (3v, *Z*/*E* = 35 :65)

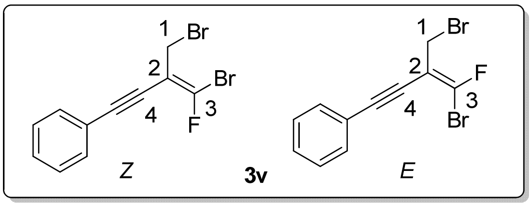

3v was purified by flash chromatography on silica gel using PE as the eluent. Colorless oil (48 mg, 78 % yield). ^{1}H NMR (400 MHz, $CDCl_3$) (*E*+*Z*) δ 7.52 – 7.48 (m, 2H, Ar**H**), 7.36 – 7.33 (m, 3H, Ar**H**), 4.14 – 4.11 (m, 2H, C**H**2). ^{13}C NMR (101 MHz, $CDCl_3$) (*E*+*Z*) δ 143.2 (d, $^{1}J_{C-F}$ = 331.3 Hz, *Z*, C3), 142.2 (d, $^{1}J_{C-F}$ = 333.3 Hz, *E*, C3), 131.9 (d, *J* = 5.8 Hz), 129.3, 128.6, 122.4 (d, *J* = 7.7 Hz). 108.4 (d, $^{2}J_{C-F}$ = 25.6 Hz, *E*, C2), 106.0 (d, $^{2}J_{C-F}$ = 15.2 Hz, *Z*, C2), 97.6 (d, $^{3}J_{C-F}$ = 7.7 Hz, *E*, C4), 96.3 (d, $^{3}J_{C-F}$ = 7.9 Hz, *Z*, C4), 83.4 (d, *J* = 6.3 Hz, *Z*), 80.4, 31.3 (*Z*, C1), 26.0 (d, $^{3}J_{C-F}$ = 2.8 Hz, *E*, C1). ^{19}F

NMR (376 MHz, $CDCl_3$) δ -56.42 (s, *Z*, 1F), -63.51 (s, *E*, 1F). HRMS-EI: m/z calculated for $C_{11}H_7Br_2F$ [M^+] 315.8893, found 315.8890.

8-bromo-9-(bromomethyl)-6,7-dihydro-5H-benzo[7]annulene (3n)

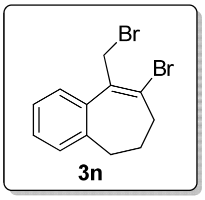

3n was purified by flash chromatography on silica gel using PE as the eluent. Colorless oil (36 mg, 46% yield). ^{1}H NMR (400 MHz, $CDCl_3$) δ 7.41 – 7.39 (m, 1H, Ar**H**), 7.34 – 7.23 (m, 3H, Ar**H**), 4.52 (s, 2H, C**H**$_2$), 2.68 (t, $^3J_{H-H}$ = 7.1 Hz, 2H, C**H**$_2$), 2.49 (t, $^3J_{H-H}$ = 7.0 Hz, 2H, C**H**$_2$), 2.32 – 2.25 (m, 2H, C**H**$_2$). ^{13}C NMR (101 MHz, $CDCl_3$) δ 140.8, 138.3, 135.4, 129.4, 128.8, 128.4, 126.8, 126.2, 37.6, 35.7, 34.1, 31.3. HRMS-EI: m/z calculated for $C_{12}H_{12}Br_2$ [M^+] 313.9300, found 313.9302.

(*Z*)-4-(1,3-dibromoprop-1-en-2-yl)-1,1'-biphenyl (3o-*Z*)

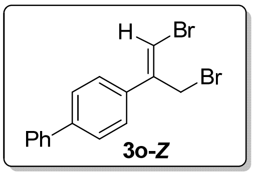

3o was purified by flash chromatography on silica gel using PE as the eluent. White solid (24 mg, 34% yield). ^{1}H NMR (400 MHz, $CDCl_3$) δ 7.62 – 7.59 (m, 4H, Ar**H**), 7.51 – 7.43 (m, 4H, Ar**H**), 7.39 – 7.35 (m, 1H, Ar**H**), 6.73 (s, 1H, =C**H**), 4.52 (s, 2H, C**H**$_2$). ^{13}C NMR (101 MHz, $CDCl_3$) δ 141.7, 141.6, 140.4, 136.7, 129.1, 127.9, 127.7, 127.2, 126.9, 110.7, 30.7. HRMS-EI: m/z calculated for $C_{15}H_{12}Br_2$ [M^+] 349.9300, found 349.9303.

(*E*)-4-(1,3-dibromoprop-1-en-2-yl)-1,1'-biphenyl (3o-*E*)

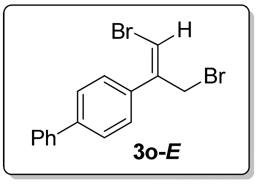

3o was purified by flash chromatography on silica gel using PE as the eluent. White solid (18 mg, 26% yield). ^{1}H NMR (400 MHz, $CDCl_3$) δ 7.66 – 7.62 (m, 4H, Ar**H**), 7.47 – 7.43 (m, 4H, Ar**H**), 7.38 – 7.34 (m, 1H, Ar**H**), 6.79 (s, 1H, =C**H**), 4.31 (s, 2H, C**H**$_2$). ^{13}C NMR (101 MHz, $CDCl_3$) δ 142.1, 141.5, 140.7, 135.7, 129.0, 128.9, 127.8, 127.3, 127.3, 109.7, 35.9. HRMS-EI: m/z calculated for $C_{15}H_{12}Br_2$ [M^+] 349.9300, found 349.9303.

6. *E* and *Z* Isomerization of the Dibrominated Products

(1)

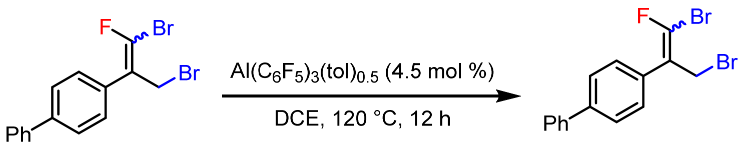

3a
Z/*E* = 10:90

90% recovery
Z/*E* = 37:63

(2)

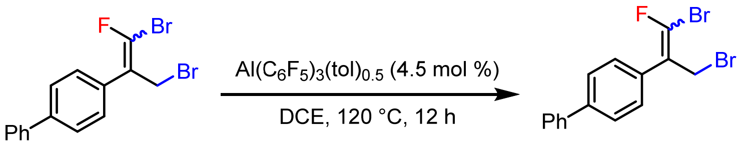

3a
Z/*E* = 93:7

87% recovery
Z/*E* = 78:22

We monitored the dibromination reaction of **1a** with ^{1}H NMR to see whether the *Z*/*E* ratio of the product (**3a**) changed over time. The ratio remained approximately 1:1 over 6 h. In addition, purification of **3a** (*Z*/*E* = 55:45) with preparative HPLC (C18 column with water/acetonitrile as the eluents) was able to provide a product mixture with *Z*/*E* = 10:90 and another product mixture with *Z*/*E* = 93:7. When these two mixtures were subjected to the reaction conditions in the absence of TMSBr (See the equations above), isomerization occurred; the mixture starting with *Z*/*E* = 10:90 isomerized to *Z*/*E* = 37:63 (90% recovery) after 12 h, and the mixture starting with *Z*/*E* = 93:7 isomerized to *Z*/*E* = 78:22 (87% recovery) after 12 h. Therefore, the *E* and *Z* isomers are interconvertible, and the *Z*/*E* ratios of the products from the dibromination reactions might be the result of the thermodynamic equilibrium.

7. Decomposition of $Al(C_6F_5)_3(tol)_{0.5}$ in DCE

$Al(C_6F_5)_3(tol)_{0.5}$ was known to undergo decompositions in chlorinated alkanes. Chen[14] reported that the addition of 0.34 equiv. of DCM to $Al(C_6F_5)_3(tol)_{0.5}$ at -30 °C produced $(C_6F_5)_2AlCl$ in 65% yield, while the addition of excess amounts of DCM generated a mixture of unidentified aluminum species. Since DCE is also a chlorinated alkane and is the optimal reaction solvent for our study, we investigated the decompositions of $Al(C_6F_5)_3(tol)_{0.5}$ in DCE. When $Al(C_6F_5)_3(tol)_{0.5}$ (5 mg) was added to DCE (1.5 mL) at room temperature, the solution turned from colorless to light

yellowish immediately. NMR analyses indicated the formation of a mixture of unidentified aluminum species. Use of the resulting mixture for the dibromination reaction of **1d** gave a similar yield (90%) and *Z*/*E* ratio (40:60) as that of the standard reaction conditions. Therefore, we conclude $Al(C_6F_5)_3(tol)_{0.5}$ is probably a precatalyst, and the mixture of unidentified aluminum species resulting from the decomposition of $Al(C_6F_5)_3(tol)_{0.5}$ in DCE is the real catalyst. However, use of ^{1}H NMR, ^{19}F NMR and ^{27}Al NMR failed to fully characterize the mixture.

8. Trisubstitution Reactions

8.1 Optimization of Reaction Conditions

Table S1 Optimization of the tribromination reaction[a]

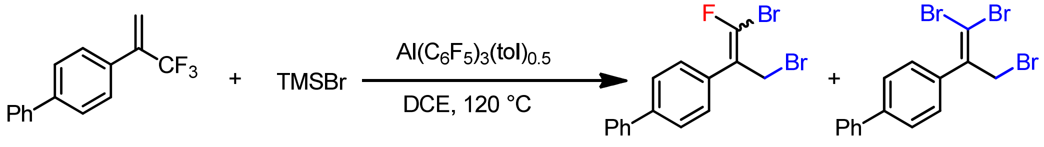

entry	$Al(C_6F_5)_3(tol)_{0.5}$	TMSBr	time (h)	**3a** (%)	**8a-Br** (%)
1	4.5 mol %	10.0 equiv.	24	44	40
2	4.5 mol %	10.0 equiv.	48	0	trace
3	9 mol %	10.0 equiv.	24	0	trace

[a] All reactions were performed with 0.1 mmol of **1a**, 1.0 mmol of TMSBr in 1 mL of DCE at 120 °C; Yields were determined by NMR spectroscopy with CH_2Br_2 as the internal standard; The ratios of **3a** to **8a-Br** were determined by ^{19}F NMR and ^{1}H NMR spectroscopy.

4-(1,1,3-tribromoprop-1-en-2-yl)-1,1'-biphenyl (8a-Br)

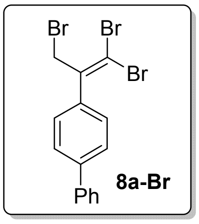

^{1}H NMR (400 MHz, $CDCl_3$) δ 7.65 – 7.61 (m, 4H, Ar**H**), 7.48 – 7.44 (m, 2H, Ar**H**), 7.41 – 7.35 (m, 3H, Ar**H**), 4.39 (s, 2H, C**H**$_2$). ^{13}C NMR (101 MHz, $CDCl_3$) δ 142.7, 141.6, 140.5, 137.9, 129.1, 128.8, 127.9, 127.5, 127.3, 96.3, 35.6. HRMS-EI: m/z calculated for $C_{15}H_{11}Br_3$ [M^+] 427.8405, found 427.8400.

Table S2 Optimization of trichlorination reaction[a]

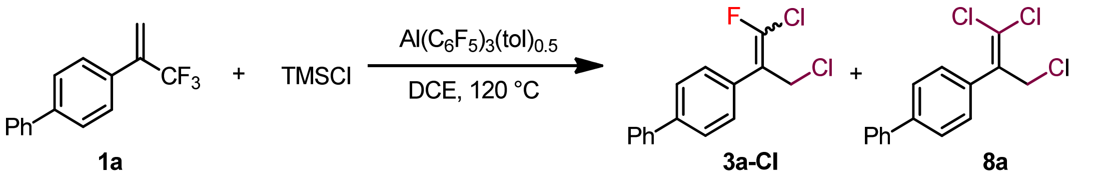

entry	$Al(C_6F_5)_3(tol)_{0.5}$	TMSCl	time (h)	**3a-Cl** (%)	**8a** (%)
1	9 mol %	5.0 equiv.	24	9	25
**2**	**9 mol %**	**7.0 equiv.**	**24**	**0**	**46[b]**
3	7.3 mol %	7.0 equiv.	24	11	45
4	4.5 mol %	7.0 equiv.	24	10	30

[a] All reactions were performed with 0.1 mmol of **1a**, 0.5–0.7 mmol of TMSCl in 1 mL of DCE at 120 °C. Yields determined by NMR spectroscopy with CH_2Br_2 as the internal standard. The ratios of **3a-Cl** to **8a** were determined by ^{19}F NMR and ^{1}H NMR spectroscopy. [b] Isolated yield.

8.2 Scope of the Trichlorination Reaction

General procedure: In a glove box, substrate **1** (0.2 mmol, 1.0 equiv.), TMSCl (153.0 mg, 1.4 mmol, 7.0 equiv.), $Al(C_6F_5)_3(tol)_{0.5}$ (10.4 mg, 9.0 mol %) were dissolved in DCE (1.5 mL) in an 8 mL vial containing a magnetic stir bar. The vial was sealed. The mixture was stirred at 120 °C in a heating block for 24 h. After being allowed to cool to room temperature, the solution was concentrated in vacuo. The residue was purified by column chromatography on silica gel using PE as the eluent. The analytical data for these products are listed as follows.

4-(1,1,3-trichloroprop-1-en-2-yl)-1,1'-biphenyl (8a)

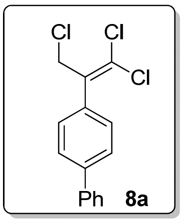

8a was purified by flash chromatography on silica gel using PE as the eluent. White solid (27 mg, 46% yield). ^{1}H NMR (400 MHz, $CDCl_3$) δ 7.61 (t, J = 8.1 Hz, 4H, Ar**H**), 7.46 – 7.34 (m, 5H, Ar**H**), 4.50 (s, 2H, CH_2). ^{13}C NMR (101 MHz, $CDCl_3$) δ 141.7, 140.5, 135.7, 129.1, 129.0, 127.9, 127.5, 127.3, 124.8, 45.9. HRMS-EI: m/z calculated for $C_{15}H_{11}Cl_3$ [M^+] 295.9921, found 295.9920.

1-(tert-butyl)-4-(1,1,3-trichloroprop-1-en-2-yl)benzene (8w)

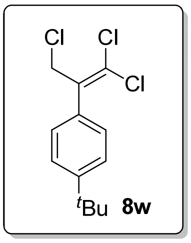

8w was purified by flash chromatography on silica gel using PE as the eluent. Colorless oil (38 mg, 69% yield). ^{1}H NMR (400 MHz, $CDCl_3$) δ 7.41 (d,$^3J_{H-H}$ = 8.4 Hz, 2H, Ar**H**), 7.27 (d, $^3J_{H-H}$ = 8.4 Hz, 2H, Ar**H**), 4.47 (s, 2H, C**H**$_2$), 1.33 (s, 9H, C**H**$_3$). ^{13}C NMR (101 MHz, $CDCl_3$) δ 151.8, 136.0, 133.9, 128.1, 125.7, 124.5, 46.1, 34.9, 31.5. HRMS-EI: m/z calculated for $C_{13}H_{15}Cl_3$ [M^+] 276.0234, found 276.0232.

1-isopropyl-4-(1,1,3-trichloroprop-1-en-2-yl)benzene (8x)

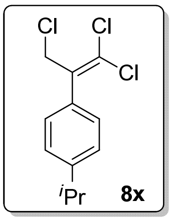

8x was purified by flash chromatography on silica gel using PE as the eluent. Colorless oil (37 mg, 70% yield). ^{1}H NMR (400 MHz, $CDCl_3$) δ 7.27 (s, 4H, Ar**H**), 4.48 (s, 2H, C**H**$_2$), 2.99 – 2.89 (m, 1H, C**H**), 1.28 (d, $^3J_{H-H}$ = 6.9 Hz, 6H, C**H**$_3$). ^{13}C NMR (101 MHz, $CDCl_3$) δ 149.5, 136.0, 134.2, 128.4, 126.8, 124.4, 46.1, 34.1, 24.0. HRMS-EI: m/z calculated for $C_{12}H_{13}Cl_3$ [M^+] 262.0077, found 262.0073.

2,4-dimethyl-1-(1,1,3-trichloroprop-1-en-2-yl)benzene (8y)

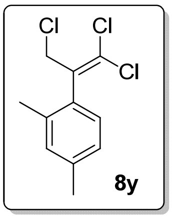

8y was purified by flash chromatography on silica gel using PE as the eluent. Colorless oil (33 mg, 67% yield). ^{1}H NMR (400 MHz, $CDCl_3$) δ 7.07 – 7.01 (m, 3H, Ar**H**), 4.43 (dd, J = 53.3, 11.1 Hz, 2H, C**H**$_2$), 2.33 (s, 3H, C**H**$_3$), 2.25 (s, 3H, C**H**$_3$). ^{13}C NMR (101 MHz, $CDCl_3$) δ 138.8, 136.0, 135.5, 133.4, 131.4, 128.5, 127.0, 124.5, 46.0, 21.4, 19.4. HRMS-EI: m/z calculated for $C_{11}H_{11}Cl_3$ [M^+] 247.9921, found 247.9918.

1-chloro-4-(1,1,3-trichloroprop-1-en-2-yl)benzene (8z)

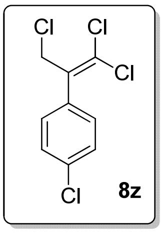

8z was purified by flash chromatography on silica gel using PE as the eluent and preparative HPLC (C18 column with water/acetonitrile as the eluents). Colorless oil (35 mg, 69% yield). ^{1}H NMR (400 MHz, $CDCl_3$) δ 7.40 – 7.37 (m, 2H, Ar**H**), 7.29 – 7.27 (m, 2H, Ar**H**), 4.45 (s, 2H, C**H**$_2$).

^{13}C NMR (101 MHz, $CDCl_3$) δ 135.2, 135.0, 134.9, 130.0, 129.1, 125.3, 45.7. HRMS-EI: m/z calculated for $C_9H_6Cl_4$ [M^+] 253.9218, found 253.9218.

4-(1,1,3-trichloroprop-1-en-2-yl)dibenzo[b,d]thiophene (8e)

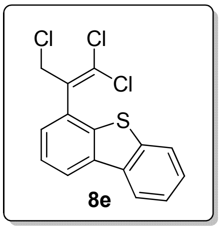

8e was purified by flash chromatography on silica gel using PE as the eluent. Colorless oil (26 mg, 40% yield). ^1H NMR (400 MHz, $CDCl_3$) δ 8.18 – 8.14 (m, 2H, Ar**H**), 7.87 – 7.82 (m, 1H, Ar**H**), 7.54 – 7.45 (m, 3H, Ar**H**), 7.40 (dd, J = 7.4, 1.0 Hz, 1H, Ar**H**), 4.60 (s, 2H, C**H**$_2$). ^{13}C NMR (101 MHz, $CDCl_3$) δ 139.2, 138.7, 136.4, 135.8, 134.7, 131.5, 127.4, 127.3, 126.8, 125.0, 124.9, 123.0, 122.0, 44.6. HRMS-EI: m/z calculated for $C_{15}H_9Cl_3S$ [M^+] 325.9485, found 325.9484.

4-(1,1,3-trichloroprop-1-en-2-yl)dibenzo[b,d]thiophene (8f)

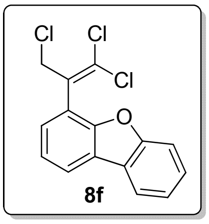

8f was purified by flash chromatography on silica gel using PE as the eluent. Colorless oil (23 mg, 43% yield). ^1H NMR (400 MHz, $CDCl_3$) δ 7.99 – 7.95 (m, 2H, Ar**H**), 7.60 – 7.58 (d, $^3J_{H\text{-}H}$ = 8.2 Hz, 1H, Ar**H**), 7.50 – 7.44 (m, 2H, Ar**H**), 7.41 – 7.35 (m, 2H, Ar**H**), 4.72 (s, 2H, C**H**$_2$). ^{13}C NMR (101 MHz, $CDCl_3$) δ 156.3, 152.9, 131.8, 128.3, 127.8, 126.2, 125.1, 124.1, 123.3, 123.0, 121.6, 121.0, 120.6, 112.1, 44.9. HRMS-EI: m/z calculated for $C_{15}H_9Cl_3O$ [M+] 309.9713, found 309.9713.

9. Transformations of Product 2d and 3a

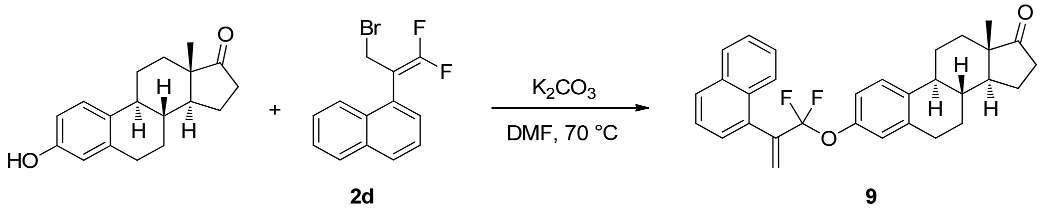

To an 8 mL vial containing a magnetic stir were added estrone (54 mg, 0.2 mmol, 2.0 equiv.), K_2CO_3 (20.7 mg, 0.15 mmol, 1.5 equiv.) and DMF (1 mL). A solution of **2d**

(28 mg, 0.1 mmol, 1.0 equiv.) in DMF (1.0 mL) was then added via a syringe at room temperature. The vial was sealed. The mixture was stirred at 70 °C for 12 h. After being allowed to cool to room temperature, the mixture was concentrated in vacuo. The residue was purified by column chromatography on silica gel using PE/EA (10:1) as the eluents to afford **9** as a white solid (30 mg, 65% yield).

(8*R*,9*S*,13*S*,14*S*)-3-((1,1-difluoro-2-(naphthalen-1-yl)allyl)oxy)-13-methyl-7,8,9,11,12,13,15,16-octahydro-6H-cyclopenta[a]phenanthren-17(14H)-one (9)

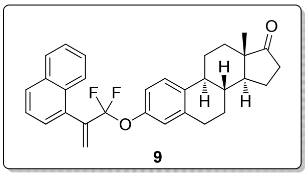

^{1}H NMR (400 MHz, $CDCl_3$) δ 8.07 – 8.05 (m, 1H, Ar**H**), 7.89 – 7.85 (m, 2H, Ar**H**), 7.54 – 7.47 (m, 4H, Ar**H**), 7.20 (d, $^3J_{H\text{-}H}$ = 8.6 Hz, 1H, Ar**H**), 6.92 (d, $^3J_{H\text{-}H}$ = 8.6 Hz, 1H, Ar**H**), 6.84 (s, 1H, Ar**H**), 6.38 (s, 1H, =C**H**$_2$), 5.61 (s, 1H, =C**H**$_2$), 2.85 – 2.82 (m, 2H, C**H**$_2$), 2.54 – 2.47 (m, 1H), 2.40 – 2.35 (m, 1H), 2.26 – 2.21 (m, 1H), 2.18 – 2.09 (m, 1H), 2.07 – 1.94 (m, 3H), 1.66 – 1.51 (m, 4H, C**H**$_2$), 1.49 – 1.35 (m, 2H, C**H**$_2$), 0.90 (s, 3H, C**H**$_3$). ^{13}C NMR (101 MHz, $CDCl_3$) δ 221.1, 148.3, 140.3 (t, $^2J_{C\text{-}F}$ = 31.1 Hz), 138.0, 137.2, 133.8, 133.6, 132.6, 128.8, 128.3, 127.5, 126.3, 126.1, 126.0, 125.1, 123.0 (t, $^3J_{C\text{-}F}$ = 5.0 Hz), 122.2, 121.4 (t, $^1J_{C\text{-}F}$ = 264.6 Hz, **C**F$_2$), 119.3, 50.6, 48.1, 44.2, 38.2, 36.0, 31.7, 29.5, 26.5, 25.9, 21.8, 14.0. ^{19}F NMR (376 MHz, $CDCl_3$) δ -68.86 (s, 2F). HRMS calculated for $C_{31}H_{30}F_2NaO_2^+$ [M+Na]$^+$,495.2106; found, 495.2083.

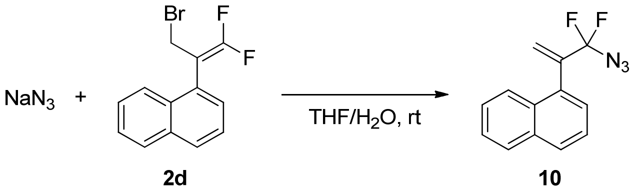

To a Schlenk tube equipped with a magnetic stir bar were added **2d** (79 mg, 0.28 mmol, 1.0 equiv.) and THF-H_2O (4:1) (1.3 mL), then NaN_3 (23 mg, 0.36 mmol, 1.3 eq.) was added at room temperature. The mixture was stirred at room temperature overnight under the flow of nitrogen. Then, the mixture was extracted with Et_2O (3×10 mL). The combined organic phase was washed with brine (40 mL), dried over Na_2SO_4, filtered and concentrated in vacuo. The residue was purified by column chromatography on silica gel using PE as the eluent to afford **10** as a colorless liquid (34 mg, 50% yield).

1-(3-azido-3,3-difluoroprop-1-en-2-yl)naphthalene (10)

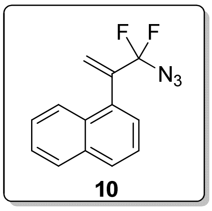

^{1}H NMR (400 MHz, $CDCl_3$) δ 7.94 – 7.92 (m, 1H, Ar**H**), 7.89 – 7.86 (m, 2H, Ar**H**), 7.53 – 7.41 (m, 4H, Ar**H**), 6.27 (s, 1H, =C**H**$_2$), 5.58 (s, 1H, =C**H**$_2$). ^{13}C NMR (101 MHz, $CDCl_3$) 139.3 (t, $^2J_{C\text{-}F}$ = 27.9 Hz), 133.8, 132.5, 132.4, 129.3, 128.5, 127.6, 126.6, 126.2, 125.8, 125.1, 123.6 (t, $^3J_{C\text{-}F}$ = 5.7 Hz), 120.7 (t, $^1J_{C\text{-}F}$ = 264.6 Hz, **C**F$_2$). ^{19}F NMR (376 MHz, $CDCl_3$) δ -76.62 (s, 2F). HRMS-CI: m/z calculated for $C_{15}H_{14}F_2N^+$ [M^+-N_2+C_2H_5] 246.1089, found 246.1088.

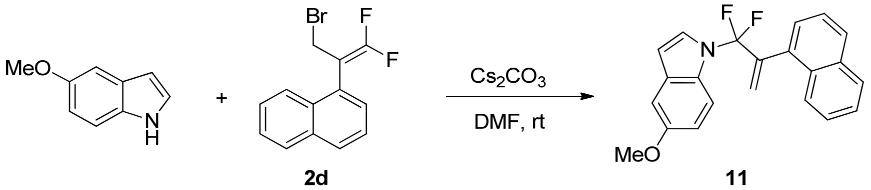

To a Schlenk tube equipped with a magnetic stir bar were added 5-methoxyindole (32 mg, 0.22 mmol, 1.1 equiv.), Cs_2CO_3 (78 mg, 0.24 mmol, 1.2 equiv.) and DMF (1.0 mL). The resulting mixture was stirred at room temperature for 0.5 h. A solution of **2d** (56.4 mg, 0.2 mmol, 1.0 equiv.) in DMF (1.0 mL) was added dropwise via a syringe. The mixture was stirred at room temperature overnight under the flow of nitrogen. Then, the mixture was concentrated in vacuo. The residue was purified by column chromatography on basic silica gel (washed by Et_3N) using PE/EA (10:1) as the eluents to afford **11** as a white solid (55 mg, 79% yield).

1-(1,1-difluoro-2-(naphthalen-1-yl)allyl)-5-methoxy-1H-indole (11)

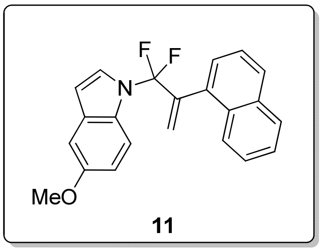

^{1}H NMR (400 MHz, $CDCl_3$) δ 7.89 (d, $^3J_{H\text{-}H}$ = 7.8 Hz, 1H, Ar**H**), 7.84 – 7.79 (m, 2H, Ar**H**), 7.52 – 7.42 (m, 3H, Ar**H**), 7.30 (dd, J = 8.1, 7.2 Hz, 1H, Ar**H**), 7.06 (dd, J = 7.6, 2.9 Hz, 2H, Ar**H**), 6.98 (d, $^3J_{H\text{-}H}$ = 7.1 Hz, 1H, Ar**H**), 6.92 (dd, J = 9.0, 2.5 Hz, 1H, Ar**H**), 6.40 (d, $^3J_{H\text{-}H}$ = 3.4 Hz, 1H, Ar**H**), 6.03 (s, 1H, =C**H**$_2$), 5.60 (s, 1H, =C**H**$_2$), 3.85 (s, 3H, OC**H**$_3$). ^{13}C NMR (101 MHz, $CDCl_3$) δ 155.3, 140.4 (t, $^2J_{C\text{-}F}$ = 32.9 Hz), 133.8, 132.5 (d, J = 4.2 Hz) 130.8, 129.9, 129.1, 128.4, 127.0, 126.5, 126.1, 125.6, 125.6, 125.5, 125.0, 123.8 (t, J = 5.8 Hz),

118.9 (t, $^{1}J_{C\text{-}F}$ = 254.5 Hz, **C**F_2), 113.6 (t, $^{3}J_{C\text{-}F}$ = 13.1 Hz), 113.2, 104.6, 103.2, 55.9. ^{19}F NMR (376 MHz, $CDCl_3$) δ -71.29 (s, 2F). HRMS-EI: m/z calculated for $C_{22}H_{17}F_2NO$ [M^+] 349.1273, found 349.1270.

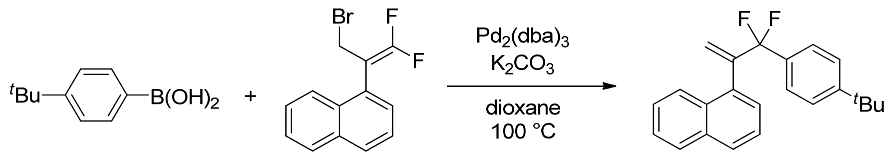

2d **12**

To an 8 mL vial containing a magnetic stir bar were added 4-*tert*-butylphenylboronic acid (71.2 mg, 0.4 mmol, 2.0 equiv.), $Pd_2(dba)_3$ (9.2 mg, 0.01 mmol, 5 mol %), K_2CO_3 (82.8 mg, 0.6 mmol, 3 equiv.), **2d** (56.4 mg, 0.2 mmol, 1 equiv.) and dry dioxane (1.5 mL). The vial was sealed. The mixture was heated at 100 °C in heating block for 24 h. The mixture was then concentrated in vacuo. The residue was purified by column chromatography on silica gel using PE as the eluent to afford **12** as a colorless liquid (41 mg, 61% yield).

1-(3-(4-(tert-butyl)phenyl)-3,3-difluoroprop-1-en-2-yl)naphthalene (12)

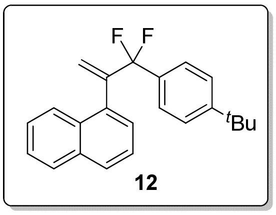

12

^{1}H NMR (400 MHz, $CDCl_3$) δ 7.86 – 7.79 (m, 3H, Ar**H**), 7.45 – 7.35 (m, 7H, Ar**H**), 7.17 (d, $^{3}J_{H\text{-}H}$ = 7.0 Hz, 1H, Ar**H**), 5.96 (d, = 0.8 Hz, 1H, =C**H**$_2$), 5.44 (d, *J* = 0.5 Hz, 1H, =C**H**$_2$), 1.30 (s, 9H, C(C**H**$_3$)$_3$). ^{13}C NMR (101 MHz, $CDCl_3$) δ 153.1 (t, *J* = 1.5 Hz), 144.5 (t, $^{2}J_{C\text{-}F}$ = 28.6 Hz), 134.3, 133.7, 133.3 (t, $^{2}J_{C\text{-}F}$ = 28.0 Hz), 132.8, 128.5, 128.2, 127.6, 126.1, 126.1, 125.9, 125.8, 125.8, 125.7, 125.3, 124.9, 121.8 (t, $^{3}J_{C\text{-}F}$ = 7.3 Hz), 120.8 (t, $^{1}J_{C\text{-}F}$ = 244.4 Hz, **C**F_2), 34.9, 31.4. ^{19}F NMR (376 MHz, $CDCl_3$) δ -94.36(br 2F). HRMS-EI: m/z calculated for $C_{23}H_{22}F_2$ [M^+] 336.1684, found 336.1688.

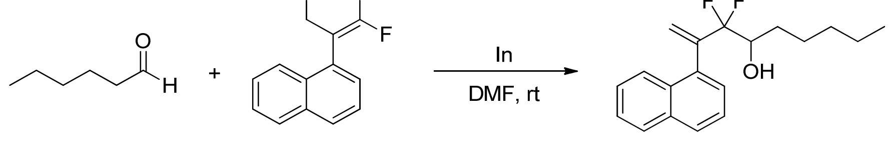

2d **13**

To a Schlenk tube equipped with a magnetic stir bar were added a solution of **2d** (56.4 mg, 0.2 mmol, 1.0 equiv.) in DMF (1.0 mL) and indium powder (35 mg, 0.3 mmol, 1.5

equiv.). The mixture was stirred for 10 min, followed by the addition of hexanal (44 mg, 0.44 mmol, 2.2 equiv.). The resulting mixture was stirred at room temperature for 14 h under the flow of nitrogen, and then the reaction was quenched with aqueous HCl (10%, 10 mL). The mixture was extracted with Et_2O (3×10 mL), and the organic extracts were washed with brine (40 mL), dried over Na_2SO_4, filtered and concentrated in vacuo. The residue was purified by column chromatography on silica gel using PE/EA (10:1) as the eluents to afford **13** as a colorless liquid (38 mg, 63% yield).

3,3-difluoro-2-(naphthalen-1-yl)non-1-en-4-ol (13)

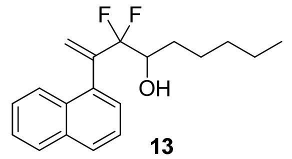

13

^{1}H NMR (400 MHz, $CDCl_3$) δ 8.08 – 8.06 (m, 1H, Ar**H**), 7.86 – 7.83 (m, 2H, Ar**H**), 7.51 – 7.44 (m, 4H, Ar**H**), 6.16 (s, 1H, C=C**H**$_2$), 5.56 (s, C=C**H**$_2$), 3.74 – 3.64 (m, 1H, C**H**), 1.77 – 1.68 (m, 2H, C**H**$_2$), 1.58 – 1.48 (m, 2H, C**H**$_2$), 1.23 (br, 4H, C**H**$_2$), 0.84 (t, J = 6.2 Hz, 3H, C**H**$_3$). ^{13}C NMR (101 MHz, $CDCl_3$) δ 141.3 (t, $^2J_{C\text{-}F}$ = 24.0 Hz, =**C**CF$_2$), 134.5, 133.9, 132.5, 128.9, 128.5, 127.2, 126.5, 126.1, 125.1, 123.1 (t, $^3J_{C\text{-}F}$ = 8.2 Hz), 121.1 (t, $^1J_{C\text{-}F}$ = 246.0 Hz, **C**F$_2$), 72.4 (t, $^2J_{C\text{-}F}$ = 28.4 Hz, **C**OH), 31.8, 29.9, 25.4, 22.6, 14.2. ^{19}F NMR (376 MHz, DMSO) δ -106.48 (d, $^2J_{F\text{-}F}$ = 235.7 Hz, 1F), -112.43 (d, $^2J_{F\text{-}F}$ = 250.7 Hz, 1F). HRMS calculated for $C_{19}H_{22}F_2NaO_2^+$ $[M+Na]^+$, 327.1531; found, 327.1513.

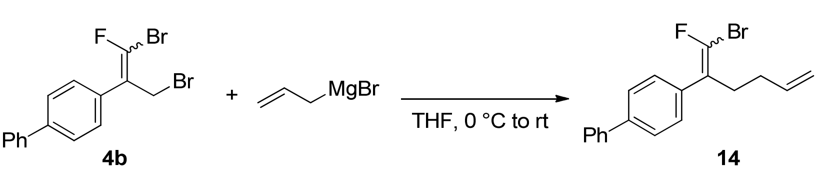

To a Schlenk tube equipped with a magnetic stir bar were added a solution of **4b** (478 mg, 1.3 mmol, 1.0 equiv.) in THF (5.0 mL), followed by dropwise addition of allylmagnesium bromide (1M in THF, 1.95 mmol, 1.5 equiv.) at 0 °C. The mixture was allowed to slowly warm to room temperature and stirred overnight under the flow of nitrogen. The mixture was then diluted with cold aqueous saturated NH_4Cl(3 mL), and extracted with EtOAc (3 × 10 mL). The combined organic phase was dried over $MgSO_4$, filtered and concentrated in vacuo. The residue was purified by column chromatography on silica gel using PE as the eluents to afford **14** (210 mg, 64% yield)

as a white foam.

(*Z*/*E*)-4-(1-bromo-1-fluorohexa-1,5-dien-2-yl)-1,1'-biphenyl (14, *Z*/*E* = 43:57)

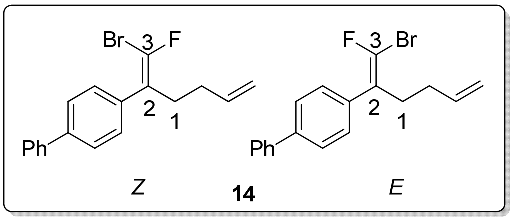

^{1}H NMR (400 MHz, $CDCl_3$) δ 7.62 – 7.58 (m, 4H, Ar**H**), 7.44 (t, $^{3}J_{H\text{-}H}$ = 7.6 Hz, 2H, Ar**H**), 7.37 – 7.30 (m, 3H, Ar**H**), 5.85 – 5.72 (m, 1H, =C**H**), 5.03 – 4.98 (m, 2H, =C**H**$_2$), 2.65 – 2.56 (m, 2H, C**H**$_2$), 2.17 – 2.08 (m, 2H, C**H**$_2$). ^{13}C NMR (101 MHz, $CDCl_3$) δ 140.7 (d, *J* = 3.7 Hz), 140.7, 137.3 (d, *J* = 5.8 Hz), 136.6 (d, *J* = 4.8 Hz), 134.3, 132.8 (d, $^{1}J_{C\text{-}F}$ = 319.2 Hz, *Z*, C_3), 131.8 (d, $^{1}J_{C\text{-}F}$ = 318.2 Hz, *E*, C_3), 129.5 (d, *J* = 3.2 Hz), 129.0, 128.9 (d, *J* = 3.3 Hz). 127.7, 127.3, 127.3, 127.3, 127.2, 123.3 (d, $^{2}J_{C\text{-}F}$ = 15.2 Hz, *Z*, C_2), 121.7 (d, $^{2}J_{C\text{-}F}$ = 7.7 Hz, *E*, C_2), 115.8, 115.8, 33.8 (s, *E*), 31.8 (d, *J* = 2.5 Hz), 31.7 (d, $^{3}J_{C\text{-}F}$ = 3.6 Hz, *Z*, C_1), 31.2 (d, $^{3}J_{C\text{-}F}$ = 3.2 Hz, *E*, C_1). ^{19}F NMR (376 MHz, $CDCl_3$) δ -78.18 (*E*, 1F), -79.02 (*Z*, 1F). HRMS-EI: m/z calculated for $C_{18}H_{16}BrF$ [M^+] 330.0414, found 330.0406.

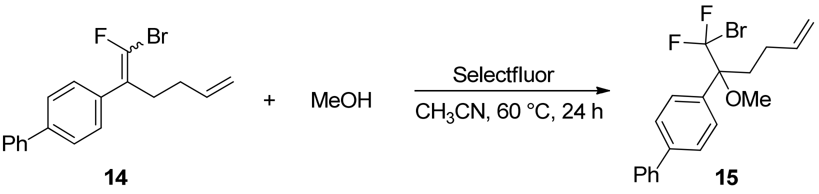

In a glove box, Selectfluor (85 mg, 0.24 mmol, 1.5 equiv.), **14** (53 mg, 0.16 mmol, 1.0 equiv.) and CH_3CN (1.5 mL) were added to an 8 mL vial equipped with a magnetic stir . MeOH (51 mg, 1.6 mmol, 10 equiv.) was subsequently added. The vial was sealed. The mixture was stirred at 60 °C for 24 h, and then was diluted with ethyl acetate (10 mL). The mixture was then washed with brine (10 mL), dried over Na_2SO_4, filtered and concentrated in vacuo. The residue was purified by column chromatography on silica gel using PE/EA (20:1) to afford product **15** (25 mg, 41% yield) as a colorless liquid.

4-(1-bromo-1,1-difluoro-2-methoxyhex-5-en-2-yl)-1,1'-biphenyl (15)

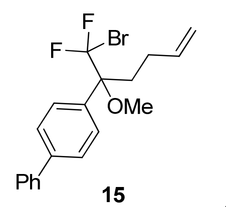

15

^{1}H NMR (400 MHz, $CDCl_3$) δ 7.63 – 7.59 (m, 6H, Ar**H**), 7.44 (t, $^{3}J_{H\text{-}H}$ = 7.4 Hz, 2H, Ar**H**), 7.35 (t, $^{3}J_{H\text{-}H}$ = 7.3 Hz, 1H, Ar**H**), 5.80 (dq, J = 11.8, 6.5 Hz, 1H, =C**H**), 5.00 (dd, J = 20.6, 13.7 Hz, 2H, =C**H**$_2$), 3.54 (s, 3H, OC**H**$_3$), 2.51 – 2.43 (m, 1H, C**H**$_2$), 2.28 – 2.20 (m, 1H, C**H**$_2$), 2.10 – 2.01 (m, 1H, C**H**$_2$), 1.92 – 1.83 (m, 1H, C**H**$_2$). ^{13}C NMR (101 MHz, $CDCl_3$) δ 141.2, 140.6, 137.6, 136.3, 129.0, 128.5, 128.3 (t, $^{1}J_{C\text{-}F}$ = 320.2 Hz, **C**F$_2$), 127.7, 127.3, 126.8, 115.3, 85.2 (t, $^{2}J_{C\text{-}F}$ = 20.2 Hz), 52.5, 29.2, 27.6. ^{19}F NMR (376 MHz, $CDCl_3$) δ -50.91 (d, $^{2}J_{F\text{-}F}$ = 14.0 Hz, 2F). HRMS calculated for $C_{19}H_{19}BrF_2NaO^+$ $[M+Na]^+$,403.0480; found,403.0462.

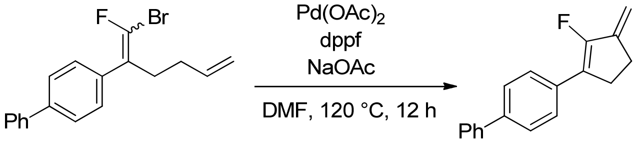

14 **16**

In a glove box, $Pd(OAc)_2$ (2.2 mg, 0.01 mmol, 10 mol %), dppf (13.5 mg, 0.025 mmol, 25 mol %), NaOAc (27.2 mg, 0.2 mmol, 2 equiv.), **14** (33.0 mg 0.1 mmol, 1.0 eq.) and DMF (2mL) were added to an 8 mL vial equipped with a magnetic stir bar. The vial was sealed. The mixture was stirred at 120 °C in heating block for 12 h. After being allowed to cool to room temperature, the mixture was concentrated in vacuo. The mixture was purified by column chromatography on silica gel using PE as the eluent to afford **16** as a white solid (14.0 mg, 54% yield).

4-(2-fluoro-3-methylenecyclopent-1-en-1-yl)-1,1'-biphenyl (16)

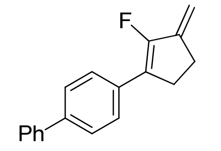

16

^{1}H NMR (400 MHz, $CDCl_3$) δ 7.68 – 7.61 (m, 6H, Ar**H**), 7.43 (t, J = 7.5 Hz, 2H, Ar**H**), 7.37 – 7.33(m, 1H, Ar**H**), 5.13 (s, 1H, =C**H**$_2$), 4.89 (s, 1H, =C**H**$_2$), 2.81 – 2.80 (m, 2H, C**H**$_2$), 2.79 – 2.68 (m, 2H, C**H**$_2$). ^{13}C NMR (101 MHz, $CDCl_3$) δ 156.8 (d, $^{1}J_{C\text{-}F}$ = 277.1 Hz, =**C**F), 144.9 (d, $^{2}J_{C\text{-}F}$ = 20.2 Hz), 140.8, 140.4 (d, J = 2.0 Hz), 132.7 (d, $^{3}J_{C\text{-}F}$ = 5.4 Hz), 129.0, 127.6, 127.6, 127.3, 127.2, 121.9 (d, J = 4.4 Hz), 101.8 (d, J = 2.5 Hz), 26.9 (d, J = 7.1 Hz), 25.1 (d, J = 6.3 Hz). ^{19}F NMR (376

MHz, $CDCl_3$) δ -131.90(s, 1F). HRMS-EI: m/z calculated for $C_{18}H_{15}F$ [M^+] 250.1152, found 250.1152.

10. Mechanistic Studies.

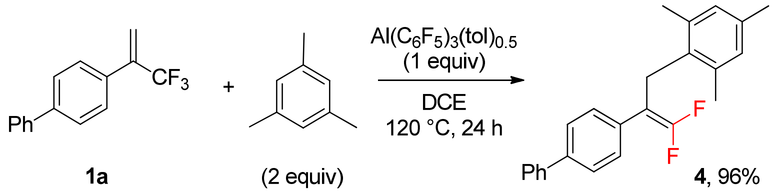

In a glovebox, **1a** (12.4 mg, 0.05 mmol, 1.0 equiv.), mesitylene (12.0 mg, 0.1 mmol, 2.0 equiv.), $Al(C_6F_5)_3(tol)_{0.5}$ (26.4 mg, 0.05 mmol, 1.0 equiv.) and DCE (1.5 mL) were added to an 8 mL vial containing a magnetic stir bar. The vial was sealed. The mixture was stirred at 120 °C in a heating block for 24 h. After being allowed to cool to room temperature, the mixture was concentrated in vacuo. The residue was purified by column chromatography on silica gel using PE as the eluent to afford pure **4** as a white solid (17 mg, 96% yield).

4-(1,1-difluoro-3-mesitylprop-1-en-2-yl)-1,1'-biphenyl (4)

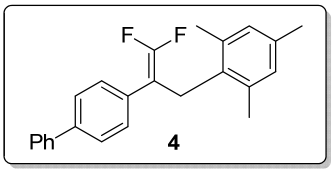

^{1}H NMR (400 MHz, $CDCl_3$) δ 7.56 (d, $^3J_{H\text{-}H}$ = 7.2 Hz, 2H, Ar**H**), 7.46 – 7.39 (m, 4H, Ar**H**), 7.34 – 7.30(m, 1H, Ar**H**), 7.02 (d, $^3J_{H\text{-}H}$ = 7.9 Hz, 2H, Ar**H**), 6.77 (s, 2H, Ar**H**), 3.66 (s, 2H, C**H**$_2$), 2.23 (s, 3H, C**H**$_3$), 2.13 (s, 6H, C**H**$_3$). ^{13}C NMR (101 MHz, $CDCl_3$) δ 153.3 (dd, $^1J_{C\text{-}F}$ = 288.9, 286.9 Hz, =**C**F$_2$), 140.8, 140.4, 137.3, 136.1, 132.9, 132.9, 129.7, 129.2, 129.0, 127.6, 127.2, 126.9, 91.2 (dd, $^2J_{C\text{-}F}$ = 19.2, 15.7 Hz, **C**=CF$_2$), 28.7, 21.0, 20.3.^{19}F NMR (376 MHz, $CDCl_3$) δ -71.15 (d, $^2J_{F\text{-}F}$ = 45.1 Hz, 1F), -72.99 (d, $^2J_{F\text{-}F}$ = 46.8 Hz, 1F). HRMS-EI: m/z calculated for $C_{24}H_{22}F_2$ [M^+] 348.1684, found 348.1685.

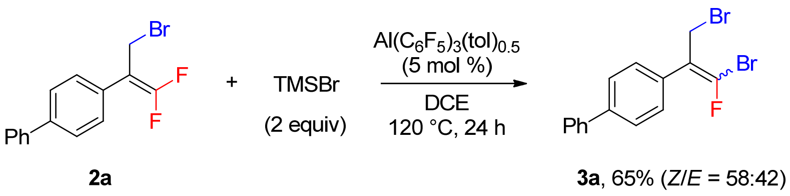

In a glovebox, **2a** (31 mg, 0.1 mmol, 1.0 equiv.), TMSBr (31 mg, 0.2 mmol, 2.0 equiv.), $Al(C_6F_5)_3(tol)_{0.5}$ (2.6 mg, 4.5 mol %) and DCE (1.5 mL) were added to an 8 mL vial containing a magnetic stir bar. The vial was sealed. The mixture was stirred at 120 °C in a heating block for 24 h. After being allowed to cool to room temperature, the mixture was concentrated in vacuo. The residue was purified by column chromatography on silica gel using PE as the eluent to afford **3a** as a white foam (24 mg, 65% yield).

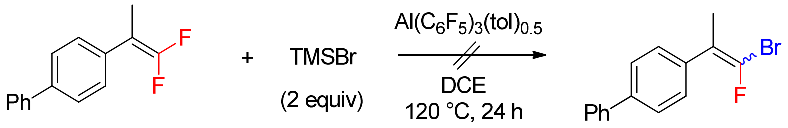

5 **6**, 0%

In a glovebox, **5** (46 mg, 0.2 mmol, 1.0 equiv.), TMSBr (61 mg, 0.4 mmol, 2.0 equiv.), $Al(C_6F_5)_3(tol)_{0.5}$ (5.2 mg, 4.5 mol %) and DCE (1.5 mL) were added to an 8 mL vial containing a magnetic stir bar. The vial was sealed. The mixture was stirred at 120 °C in a heating block for 24 h. After being allowed to cool to room temperature, the mixture was concentrated in vacuo. Analysis of the mixture by NMR spectroscopy indicated that no reaction occurred.

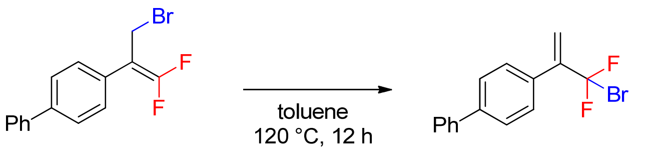

2a **7**, 83% (NMR)

In a glovebox, **2a** (31 mg, 0.1 mmol, 1.0 equiv.), toluene (1.5 mL) were added to an 8 mL vial containing a magnetic stir bar. The vial was sealed. The mixture was stirred at 120 °C in a heating block for 12 h. After being allowed to cool to room temperature, the mixture was concentrated in vacuo. ^1H NMR and ^{19}F NMR analyses of the residue suggested that **7** was produced in 83% NMR yield. Because the residue also contained unreacted **2a**, which is inseparable from **7**, we were unable to obtain pure **7** via this method. Instead, pure **7** was prepared with a reported procedure.[6]

4-(3-bromo-3,3-difluoroprop-1-en-2-yl)-1,1'-biphenyl (7)

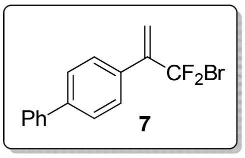

^{1}H NMR (400 MHz, $CDCl_3$) δ 7.64 – 7.58(m, 6H, Ar**H**), 7.48 – 7.36 (t, $^3J_{H\text{-}H}$ = 7.1 Hz, 2H, Ar**H**), 7.38(t, $^3J_{H\text{-}H}$ = 7.1 Hz, 1H, Ar**H**), 5.91 (s, 1H, =C**H**$_2$), 5.59 (s, 1H, =C**H**$_2$). ^{13}C NMR (101 MHz, $CDCl_3$) δ 145.4 (t, $^2J_{C\text{-}F}$ = 20.8 Hz), 142.0, 140.4, 133.6, 129.1, 128.9, 127.9, 127.3, 118.4 (t, $^3J_{C\text{-}F}$ = 7.1 Hz), 118.3 (t, $^1J_{C\text{-}F}$ = 308.1 Hz, **C**F$_2$). ^{19}F NMR (376 MHz, $CDCl_3$) δ -46.43(s, 2F). HRMS-EI: m/z calculated for $C_{15}H_{11}BrF_2$ [M$^+$] 308.0007, found 308.0009.

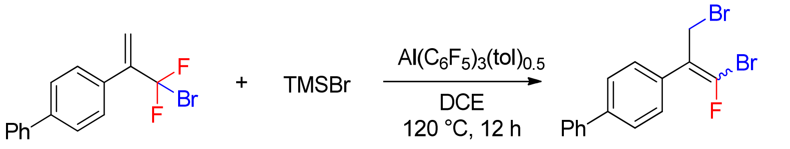

7 **3a**, 77% (*Z*/*E* = 55:45)

In a glove box, **7** (62 mg, 0.2 mmol, 1.0 equiv.), TMSBr (62 mg, 0.4 mmol, 2.0 equiv.), $Al(C_6F_5)_3(tol)_{0.5}$ (5.2 mg, 4.5 mol %) and DCE (2.0 mL) were added to an 8 mL vial containing a magnetic stir bar. The vial was sealed. The mixture was stirred at 120 °C in a heating block for 12 h. After being allowed to cool to room temperature, the mixture was concentrated in vacuo. The residue was purified by column chromatography on silica gel using PE as the eluent to afford **3a** as a white foam (57 mg, 77% yield).

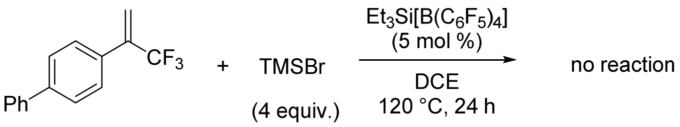

1a

In a glovebox, **1a** (50 mg, 0.2 mmol, 1.0 equiv.), TMSBr (122.4 mg, 0.8 mmol, 4.0 equiv.), $Et_3Si[B(C_6F_5)_4]$[15](7.9 mg, 5.0 mol %) and DCE (1.5 mL) were added to an 8 mL vial containing a magnetic stir bar. The vial was sealed. The mixture was stirred at 120 °C in a heating block for 24 h. After being allowed to cool to room temperature, the mixture was concentrated in vacuo. Analysis of the mixture by NMR spectroscopy indicated that no reaction occurred.

11. References

(1) Y. Liu, Y. Zhou, Y.-L. Zhao, J.-P. Qu, *Org. Lett.* **2017**, *19*, 946.

(2) B. M. Trost, L. Debien, *J. Am. Chem. Soc.* **2015**, *137*, 11606.

(3) X. Pigeon, M. Bergeron, F. Barabé, P. Dubé, H.N. Frost, J.-F. Paquin, *Angew. Chem. Int. Ed.* **2010**, *49*, 1123.

(4) M. Bergeron, T. Johnson, J.-F. Paquin, *Angew. Chem. Int. Ed.* **2011**, *50*, 11112.

(5) H. Sakaguchi, Y. Uetake, M. Ohashi, T. Niwa, S. Ogoshi, T. Hosoya, *J. Am. Chem. Soc.* **2017**, *139*, 12855.

(6) T. Fujita, R. Morioka, T. Arita, J. Ichikawa, *Chem.Commum.* **2018**, *54*, 12938.

(7) Y. Li, B. Zhao, K. Dai, D.-H. Tu, B. Wang, Y.-Y. Wang, Z.-T. Liu, Z.-W. Liu, J. Lu, *Tetrahedron* **2016**, *72*, 5684.

(8) X. Lu, X.-X. Wang, T.-J. Gong, J.-J. Pi, S.-J. He, R. Fu, *Chem. Sci.*, **2019**, *10*, 809.

(9) S. B. Lang, R. J. Wiles, C. B. Kelly, G. A. Molander, *Angew. Chem. Int. Ed.* **2017**, *56*, 15073.

(10) Y. Lan, F. Yang, C. Wang, *ACS Catal.* **2018**, *8*, 9245.

(11) O. V. Zatolochnaya, V. Gevorgyan, *Org. Lett.* **2013**, *15*, 2562.

(12) J. T. Reeves, J. J. Song, Z. Tan, H. Lee, N. K. Yee, C. H. Senanayake, *J. Org. Chem.* **2008**, *73*, 9476.

(13) S. Feng, G. R. Roof, E. Y.-X. Chen. *Organometallics* **2002**, *21*, 832.

(14) D. Chakraborty, E. Y.-X. Chen, *Inorg. Chem. Commun.*, **2002**, *5*, 698.

(15) J. B. Lambert, S. Zhang, *J. Chem. Soc., Chem. Commun.* **1993**, 383.

12. NMR Spectra

^{1}H NMR Spectrum (in $CDCl_3$, 400 MHz)

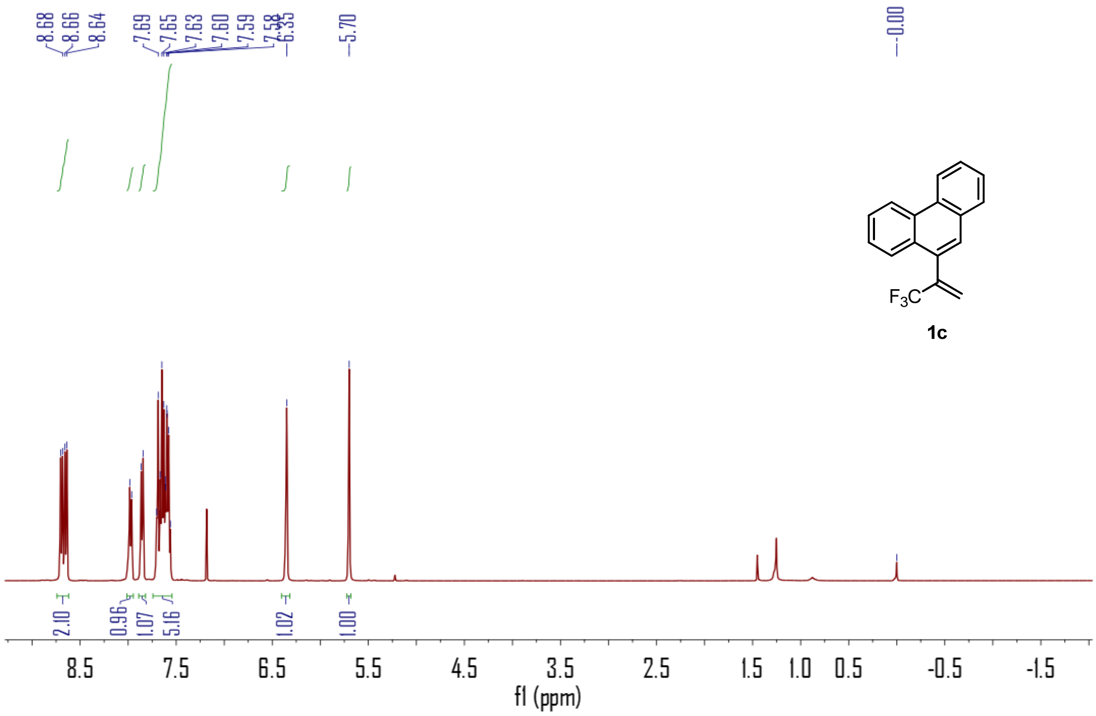

^{13}C NMR Spectrum (in $CDCl_3$, 101 MHz)

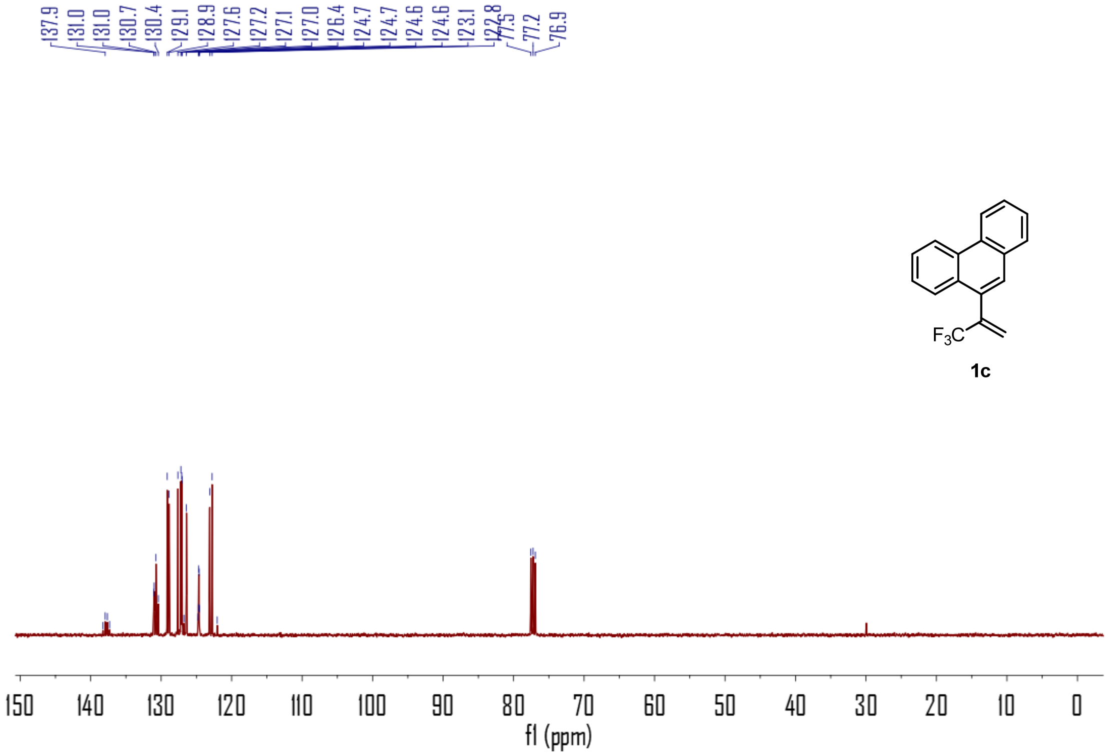

^{19}F NMR Spectrum (in $CDCl_3$, 376 MHz)

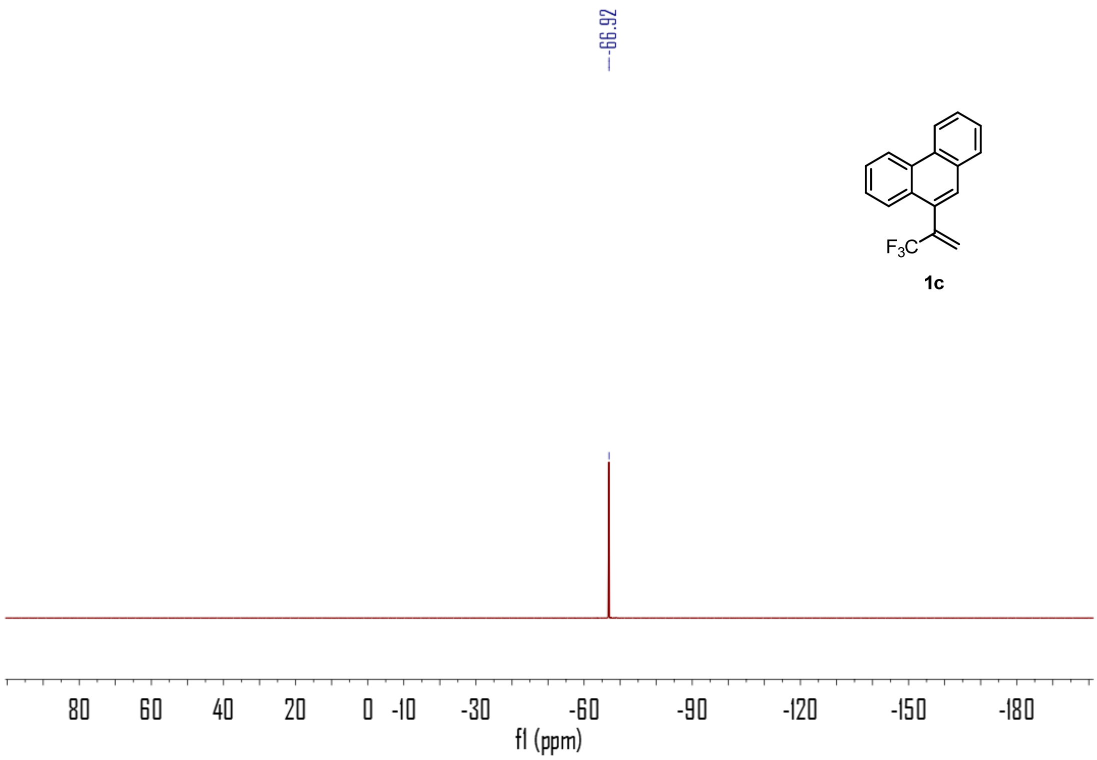

^{1}H NMR Spectrum (in $CDCl_3$, 400 MHz)

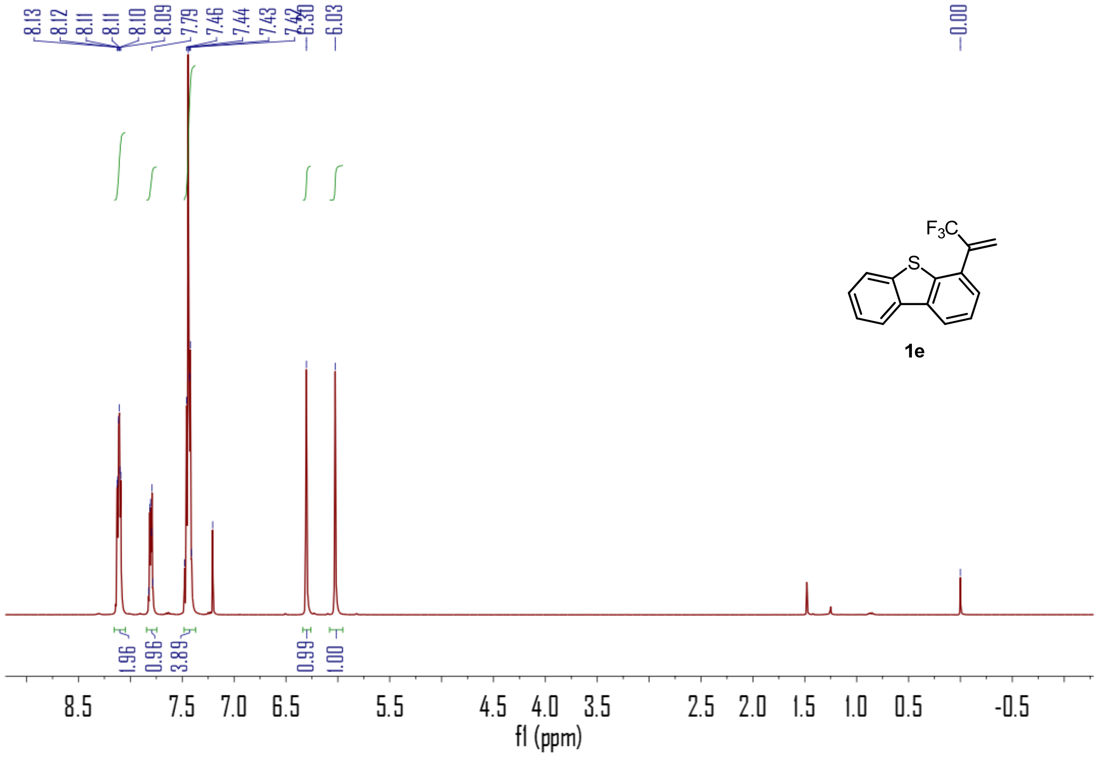

^{13}C NMR Spectrum (in CDCl3, 101 MHz)

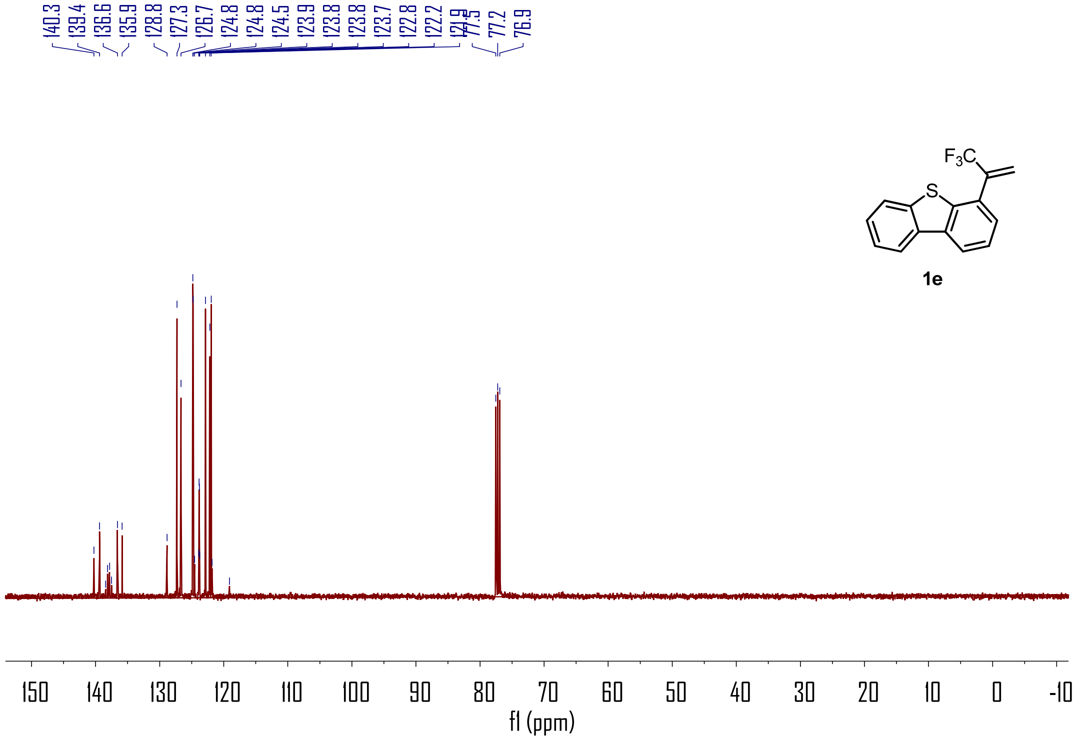

^{19}F NMR Spectrum (in CDCl3, 376 MHz)

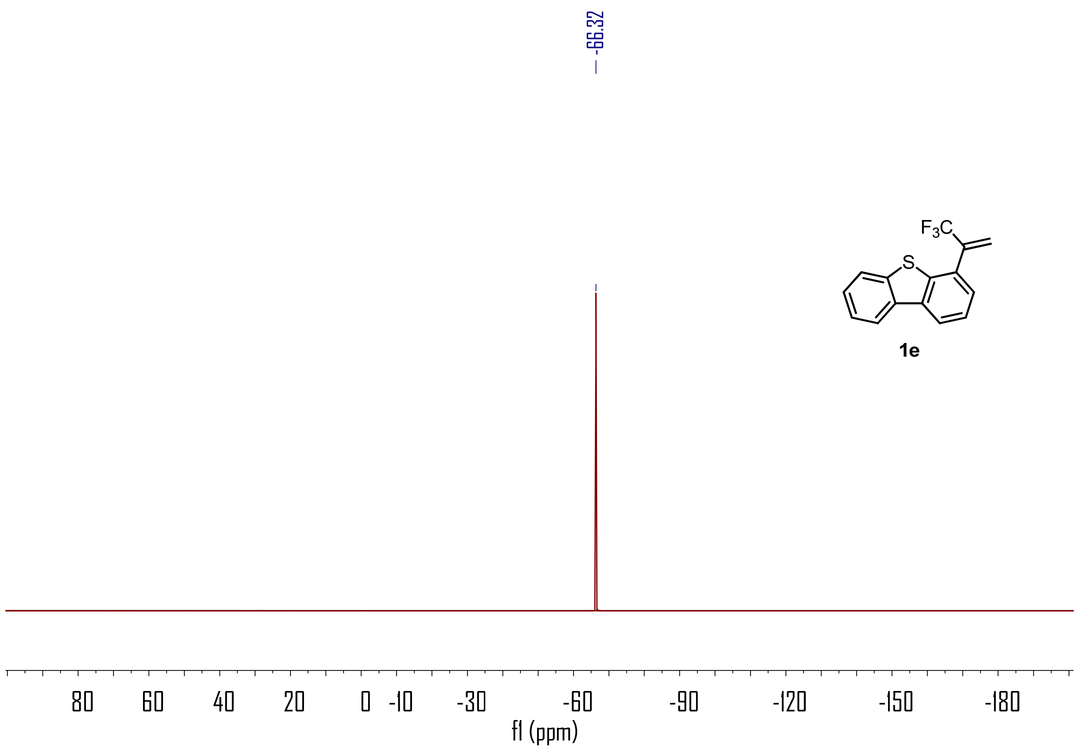

^{1}H NMR Spectrum (in $CDCl_3$, 400 MHz)

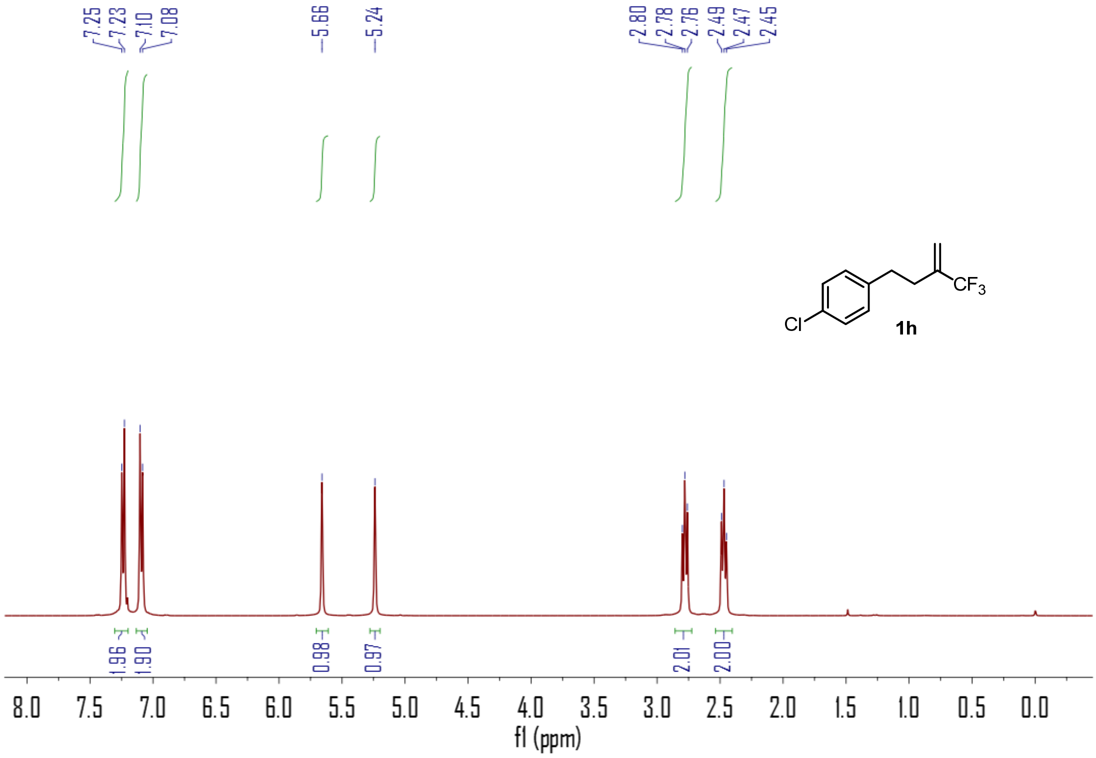

^{13}C NMR Spectrum (in $CDCl_3$, 101 MHz)

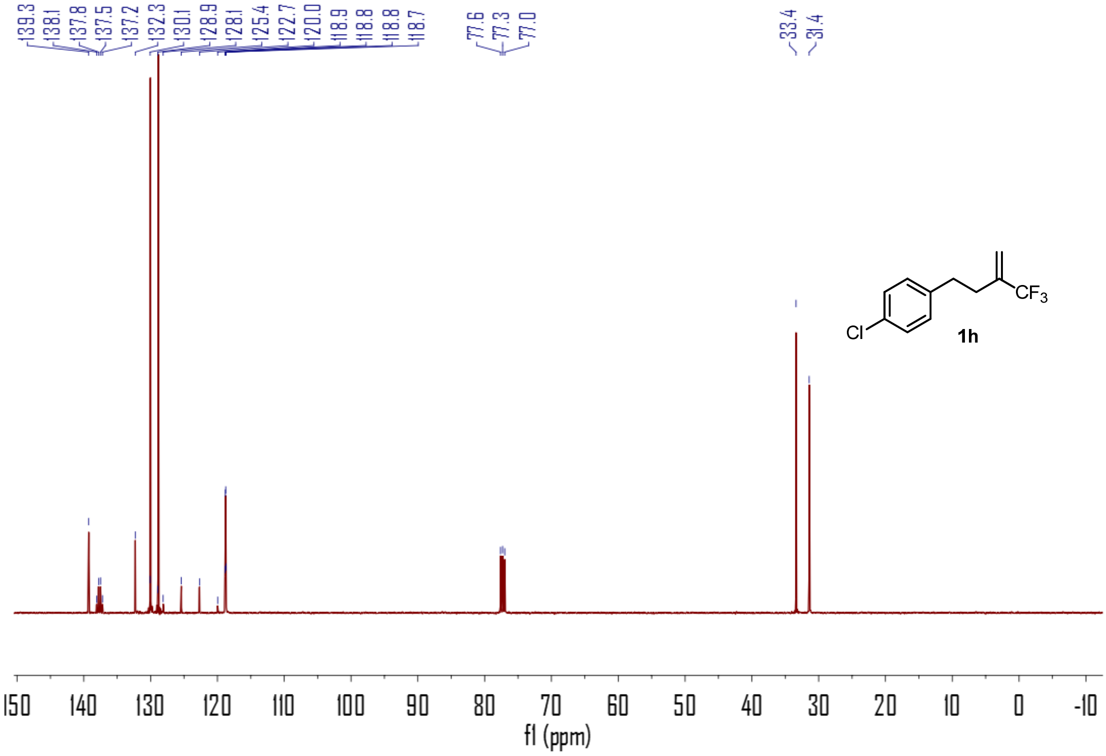

^{19}F NMR Spectrum (in $CDCl_3$, 376 MHz)

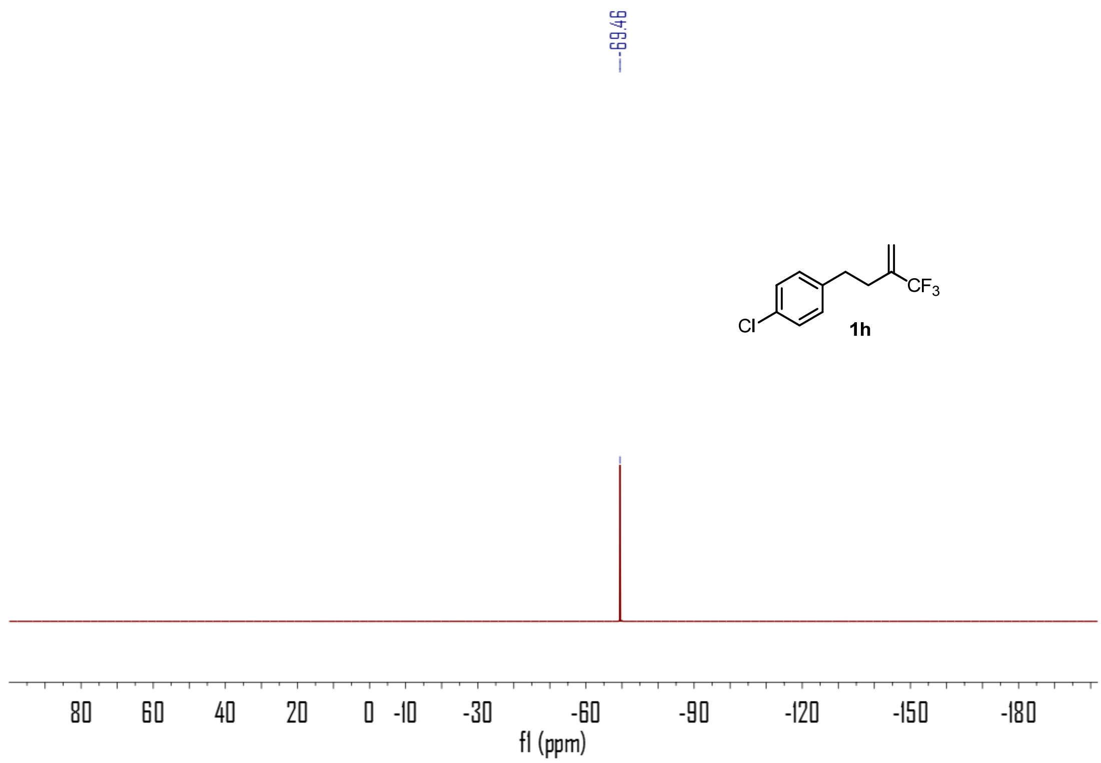

^{1}H NMR Spectrum (in $CDCl_3$, 400 MHz)

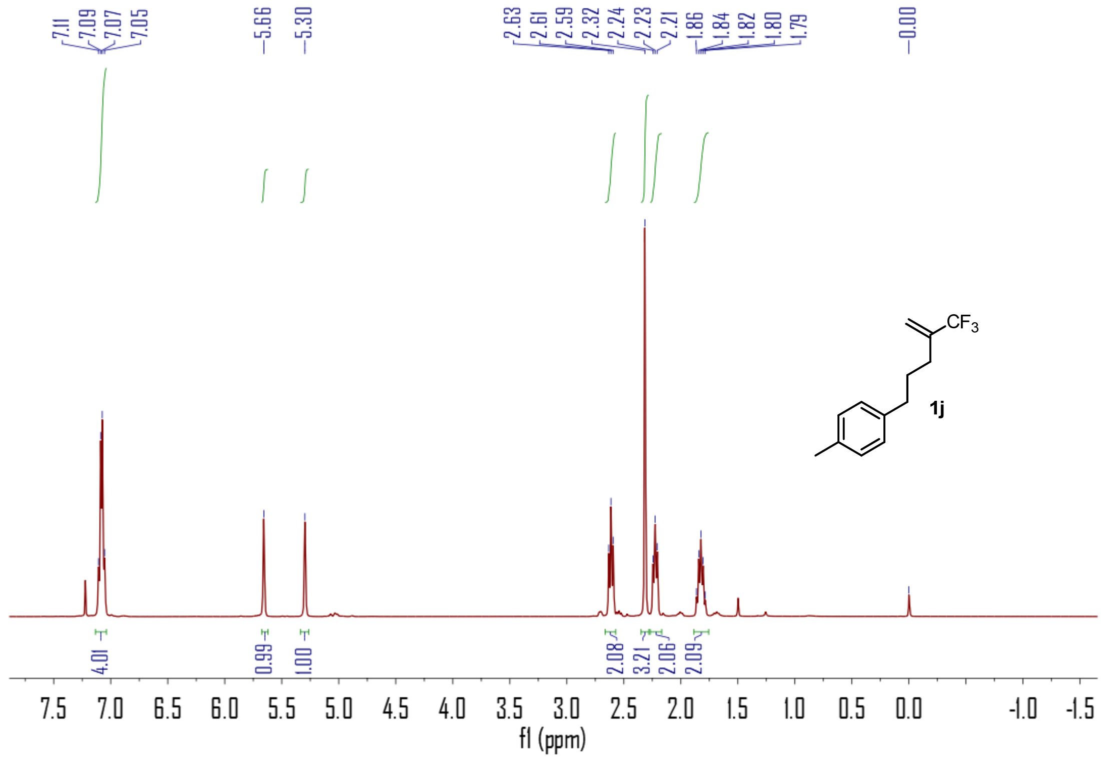

^{13}C NMR Spectrum (in $CDCl_3$, 101 MHz)

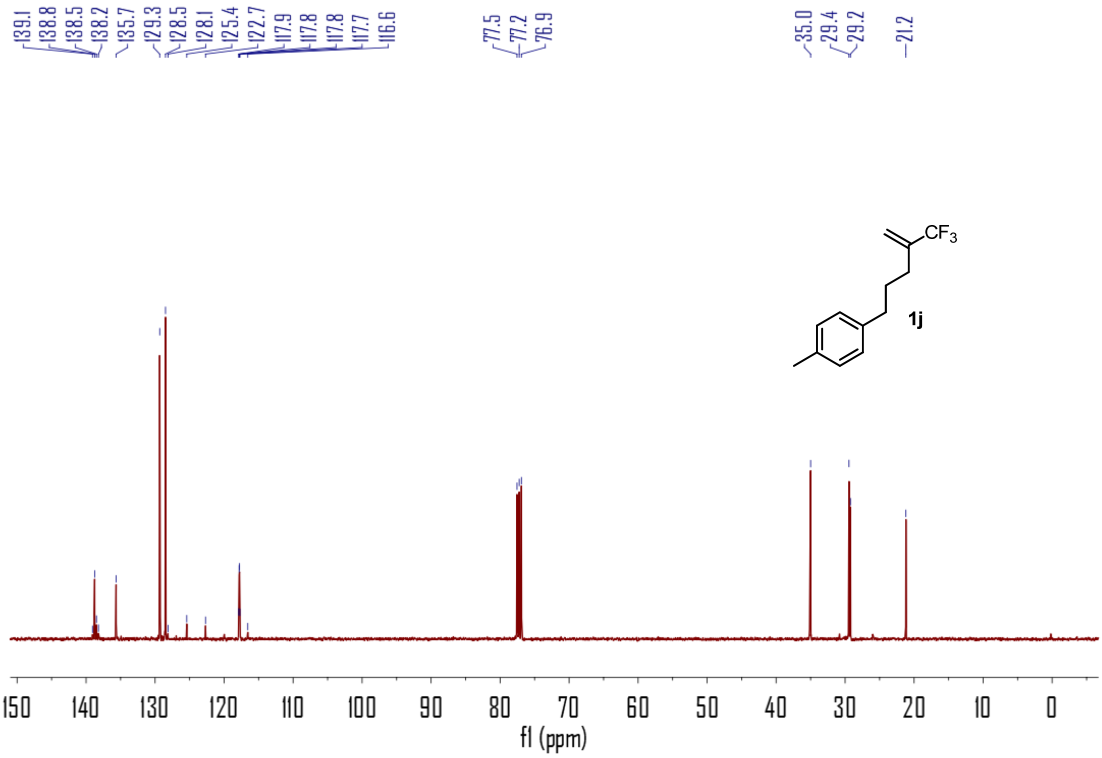

^{19}F NMR Spectrum (in $CDCl_3$, 376 MHz)

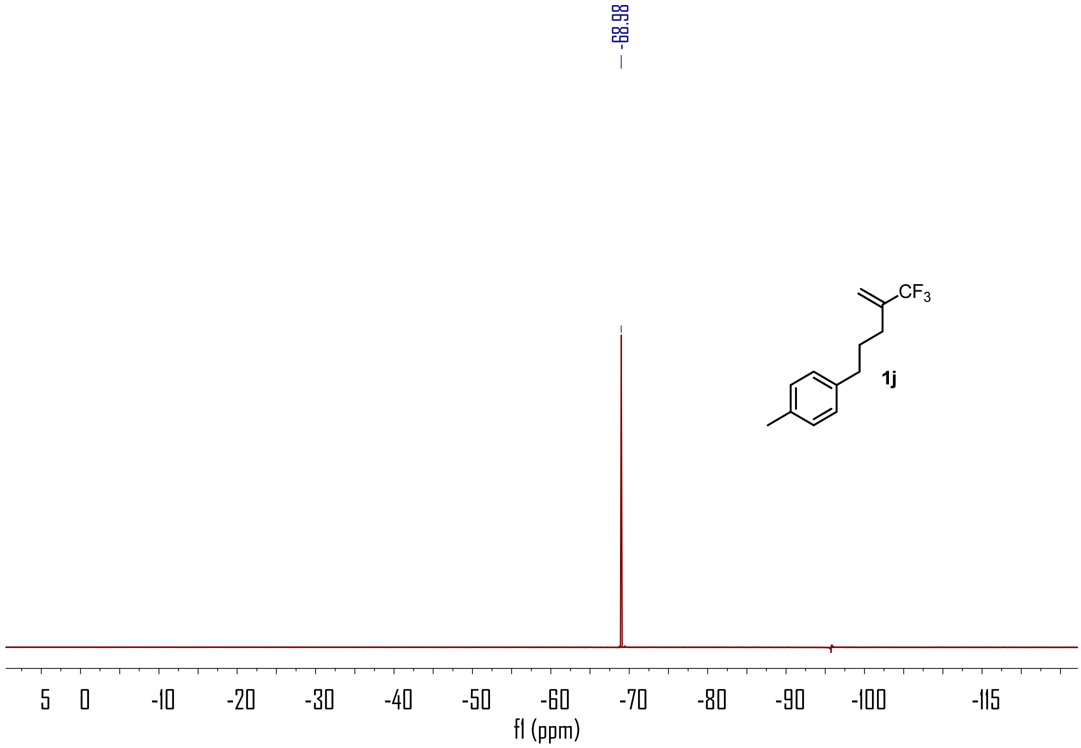

^{1}H NMR Spectrum (in $CDCl_3$, 400 MHz)

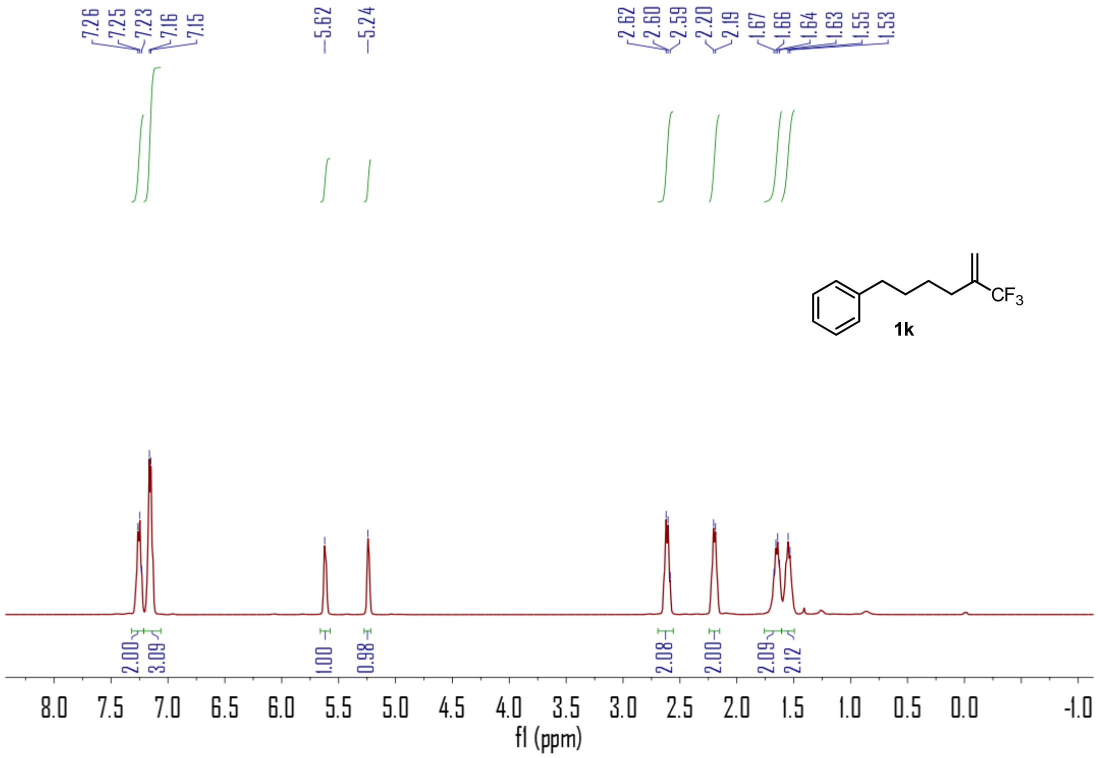

^{13}C NMR Spectrum (in $CDCl_3$, 101 MHz)

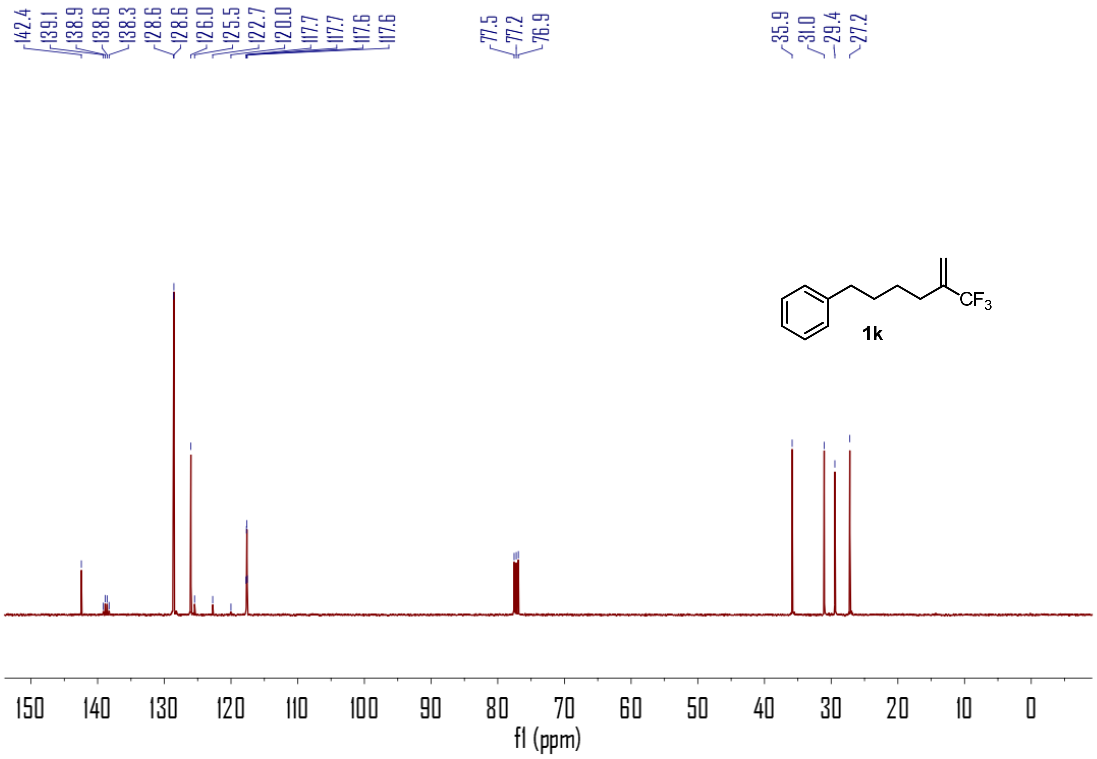

^{19}F NMR Spectrum (in $CDCl_3$, 376 MHz)

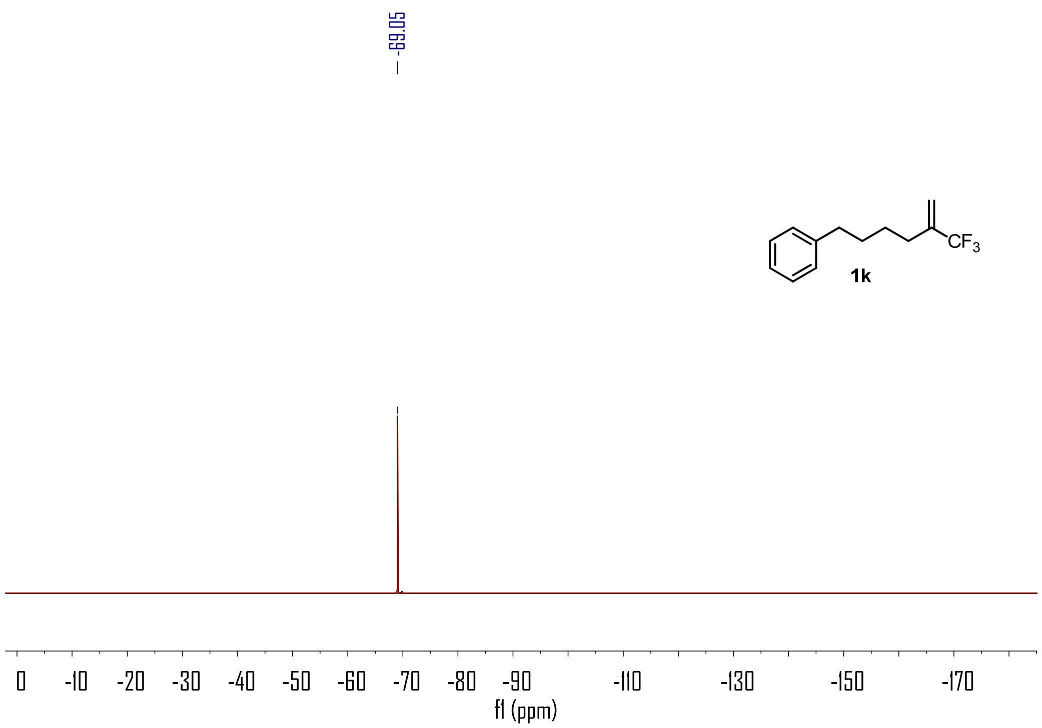

^{1}H NMR Spectrum (in $CDCl_3$, 400 MHz)

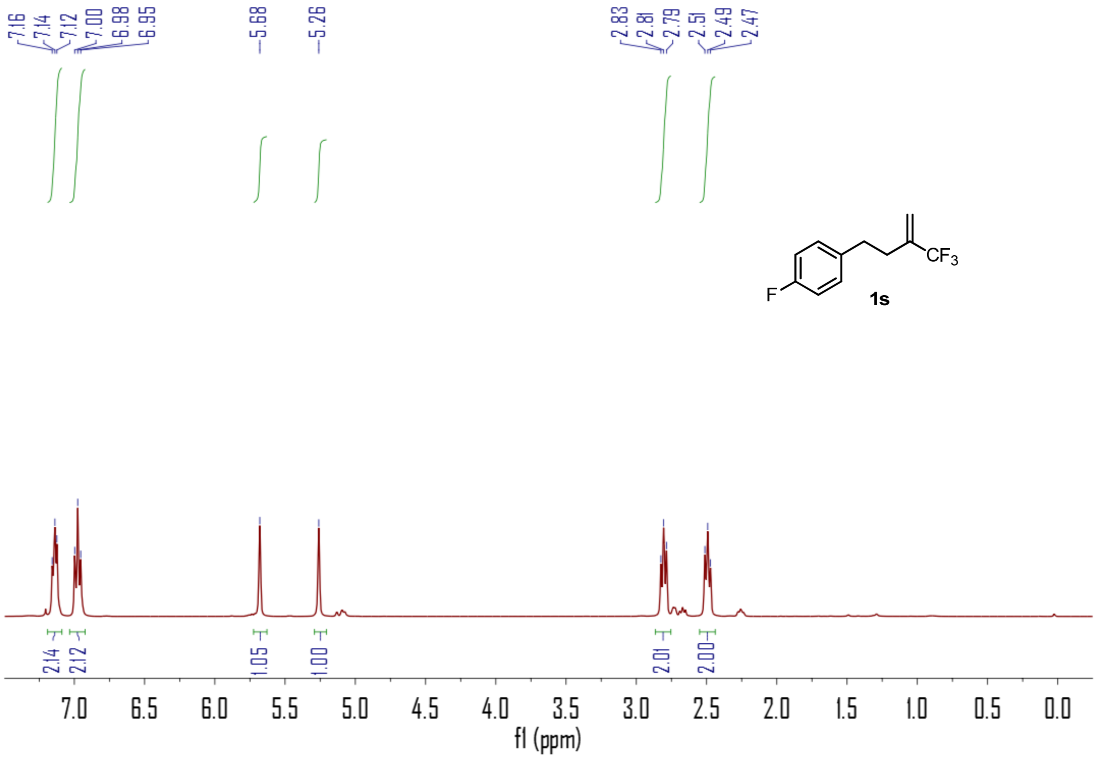

^{13}C NMR Spectrum (in $CDCl_3$, 101 MHz)

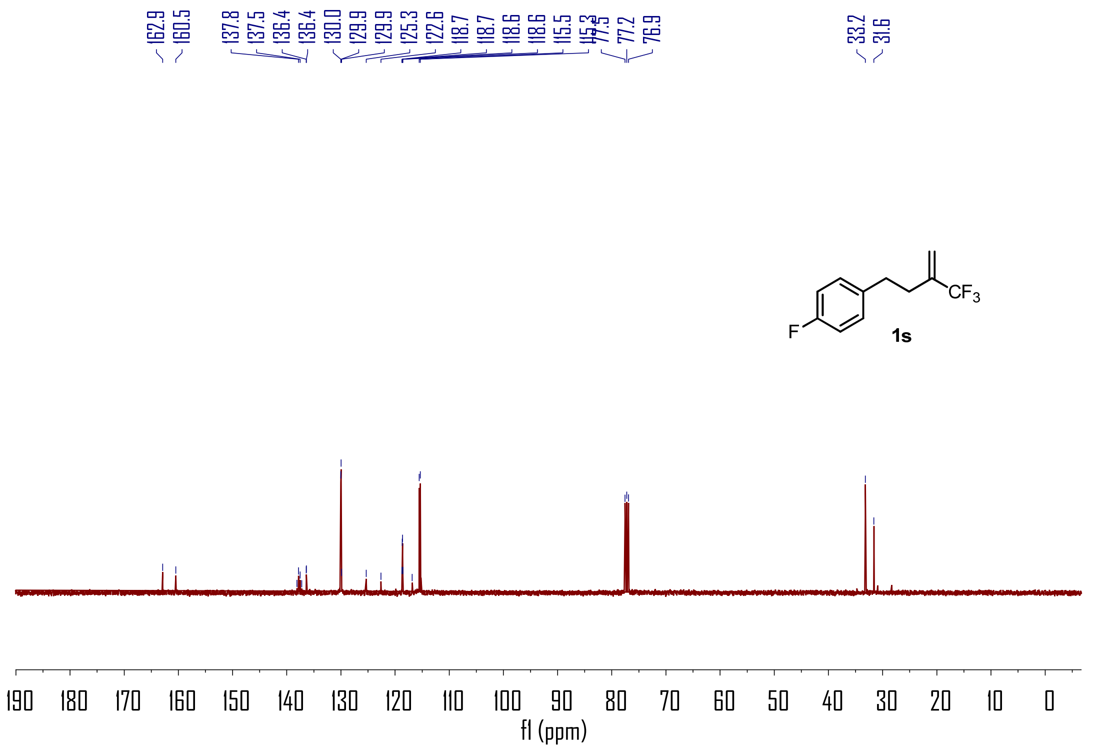

^{19}F NMR Spectrum (in $CDCl_3$, 376 MHz)

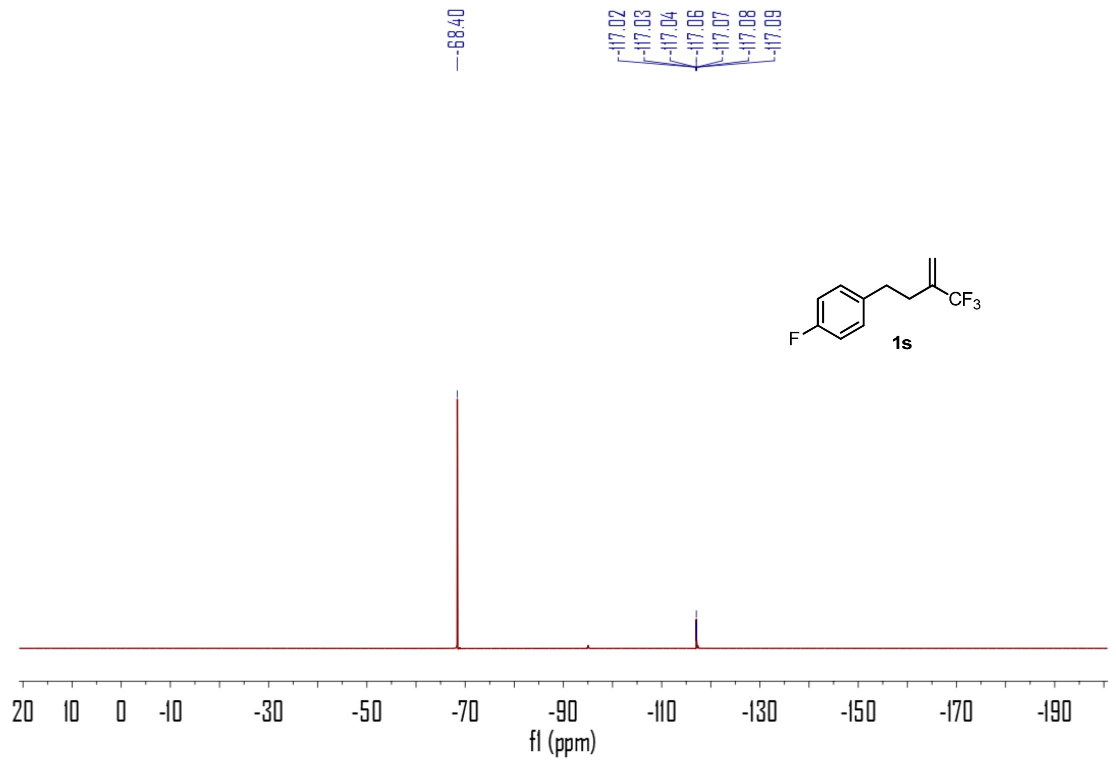

^1H NMR Spectrum (in $CDCl_3$, 400 MHz)

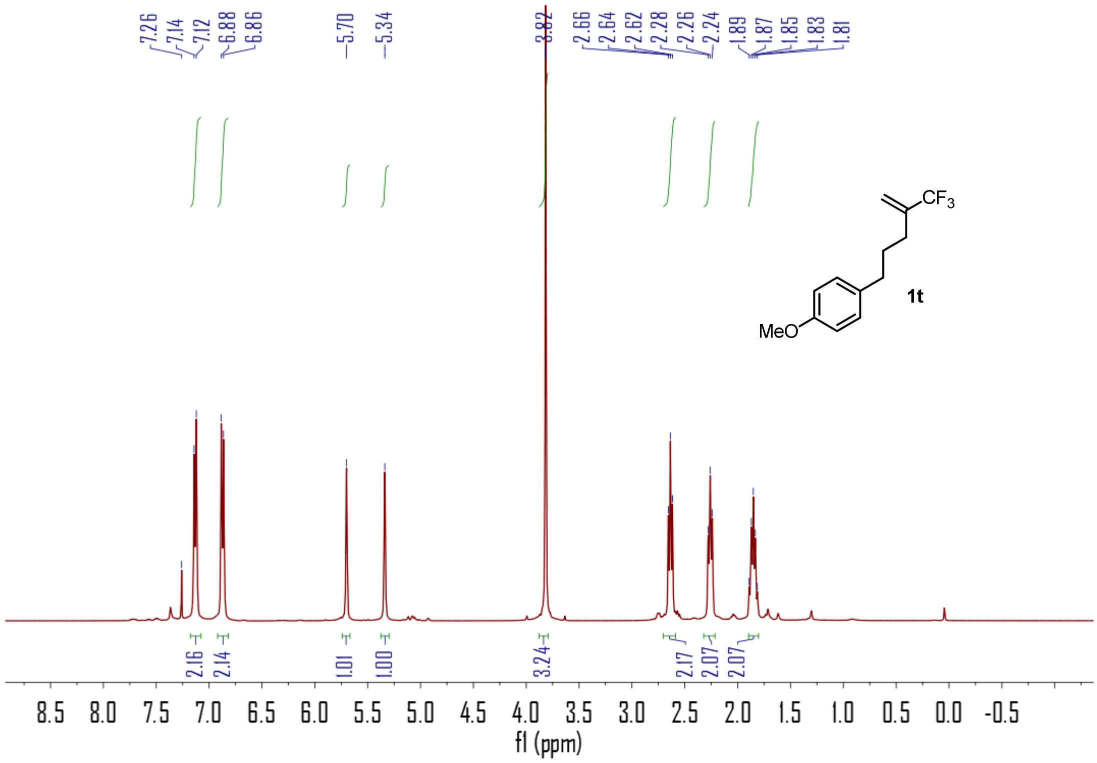

^{13}C NMR Spectrum (in $CDCl_3$, 101 MHz)

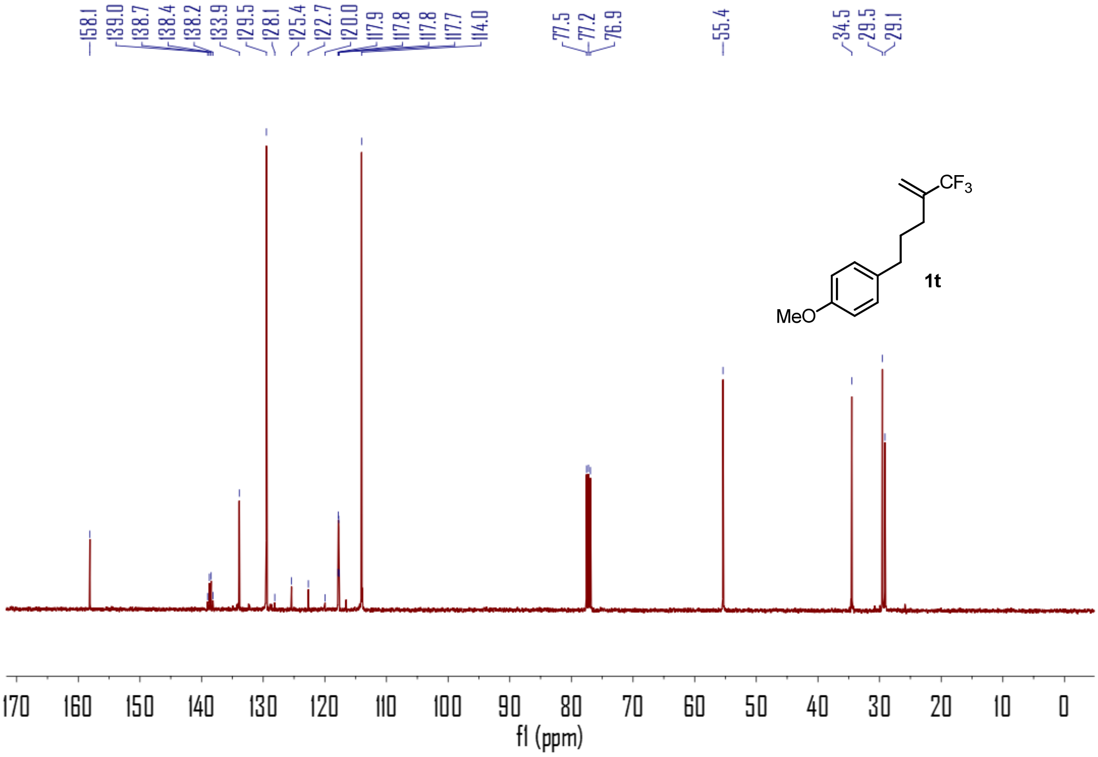

^{19}F NMR Spectrum (in $CDCl_3$, 376 MHz)

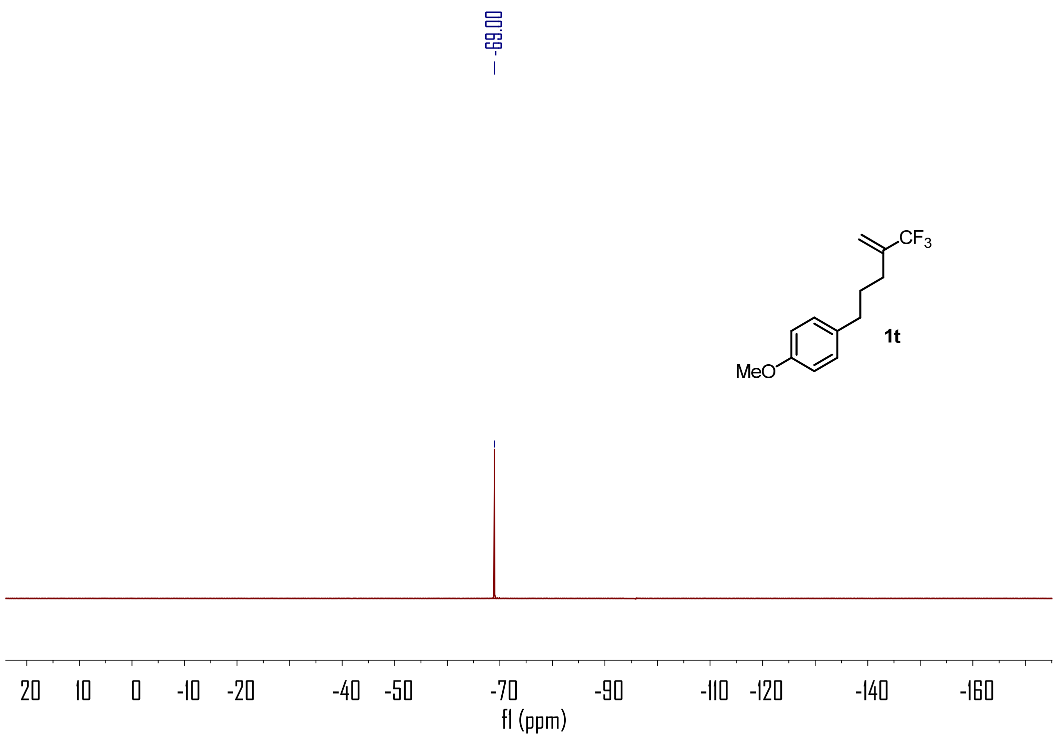

^{1}H NMR Spectrum (in C_6D_6, 400 MHz)

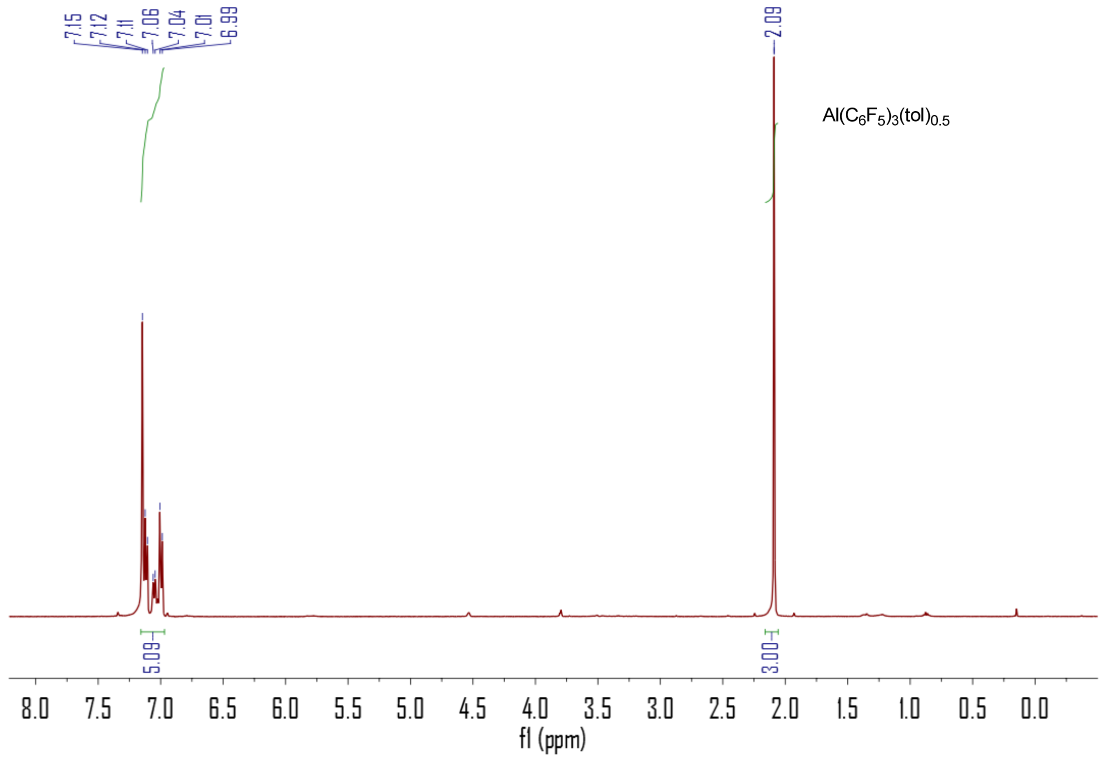

^{19}F NMR Spectrum (in C_6D_6, 376 MHz)

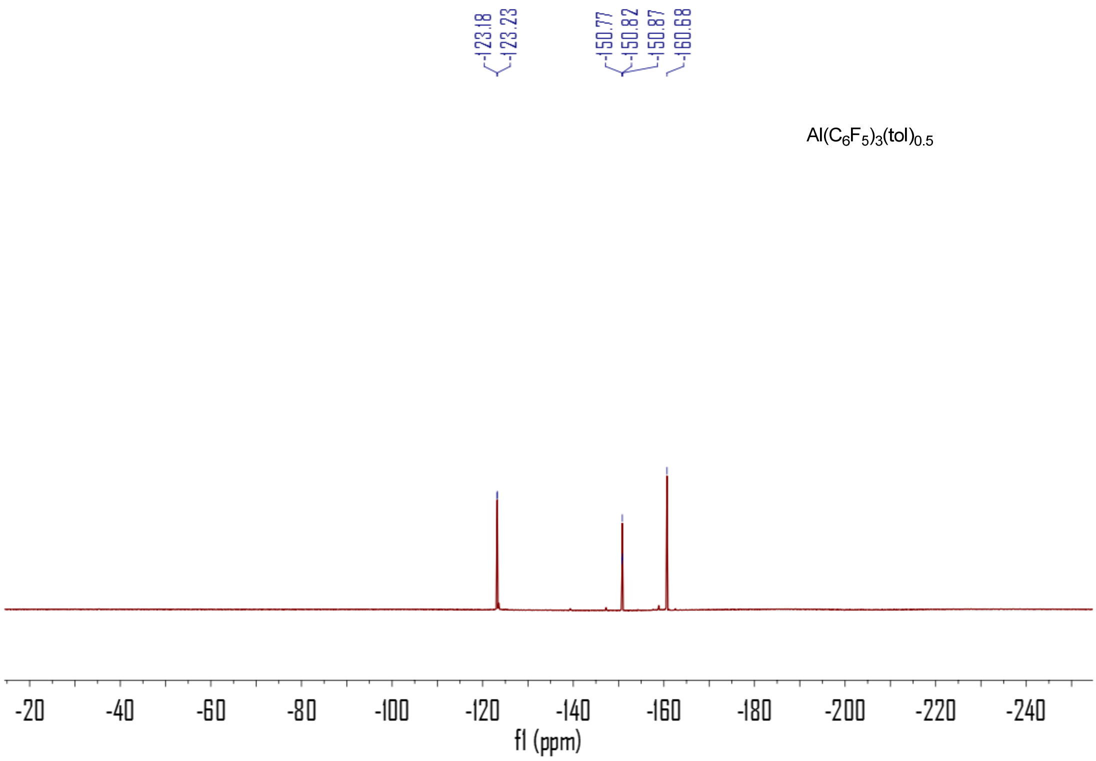

^{1}H NMR Spectrum (in $CDCl_3$, 400 MHz)

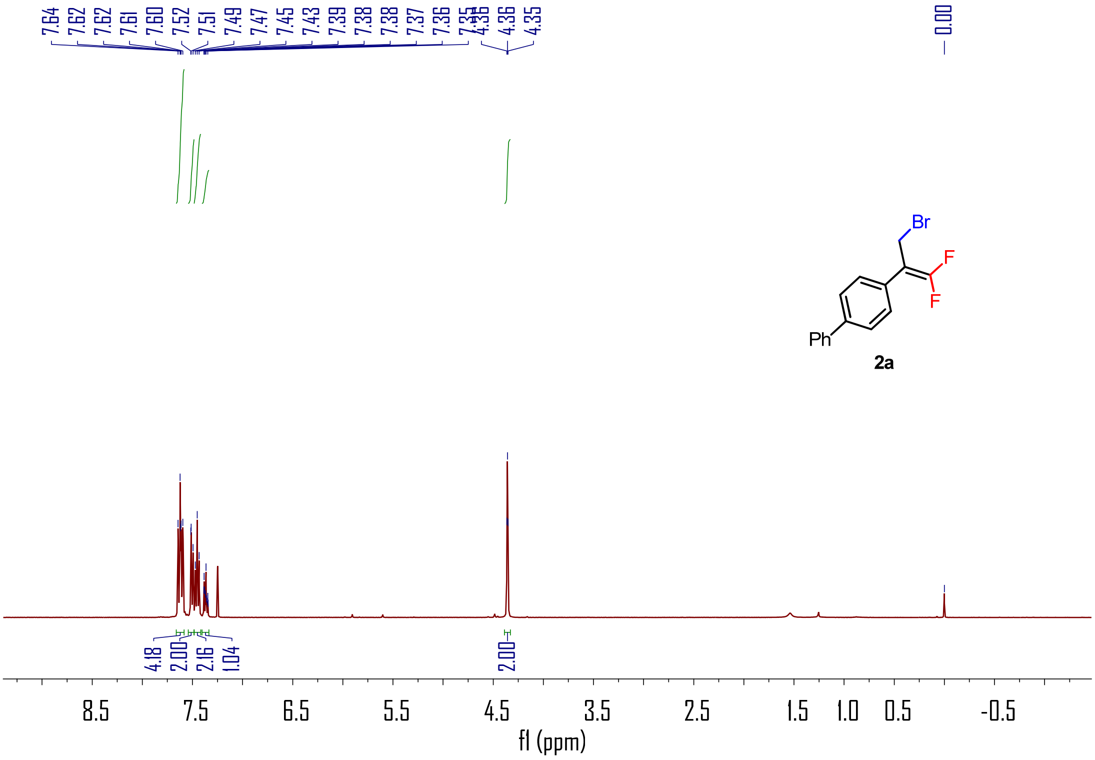

^{13}C NMR Spectrum (in CDCl$_3$, 101 MHz)

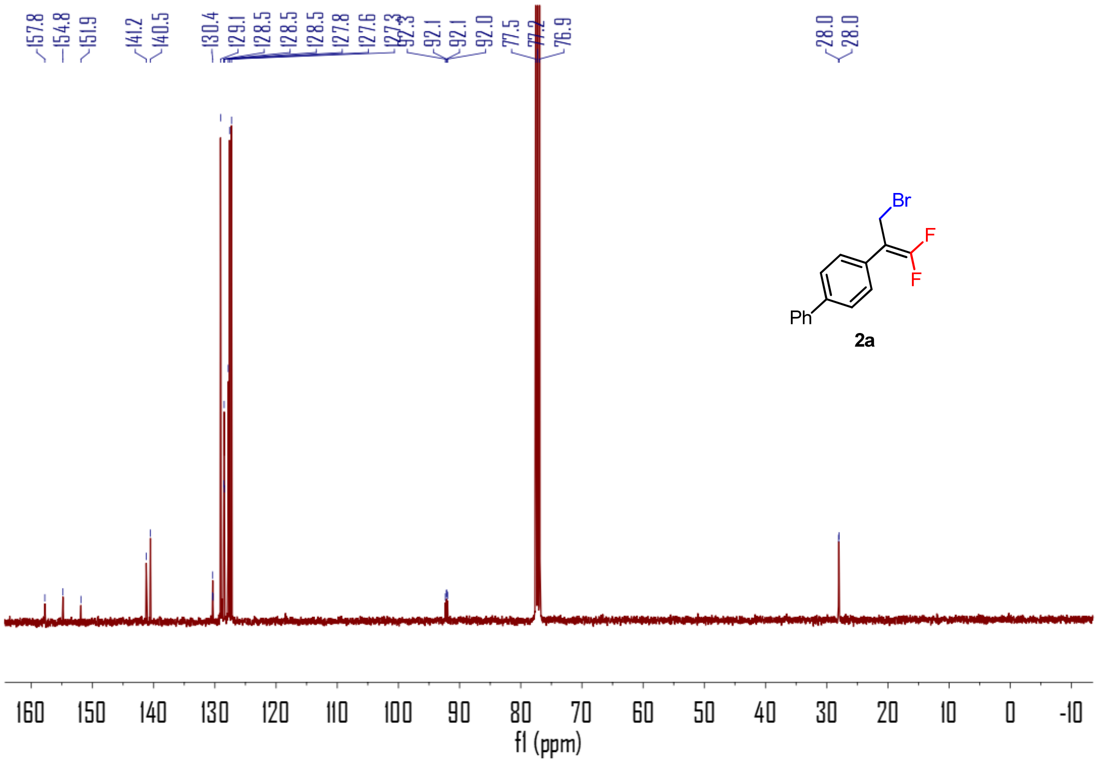

^{19}F NMR Spectrum (in CDCl$_3$, 376 MHz)

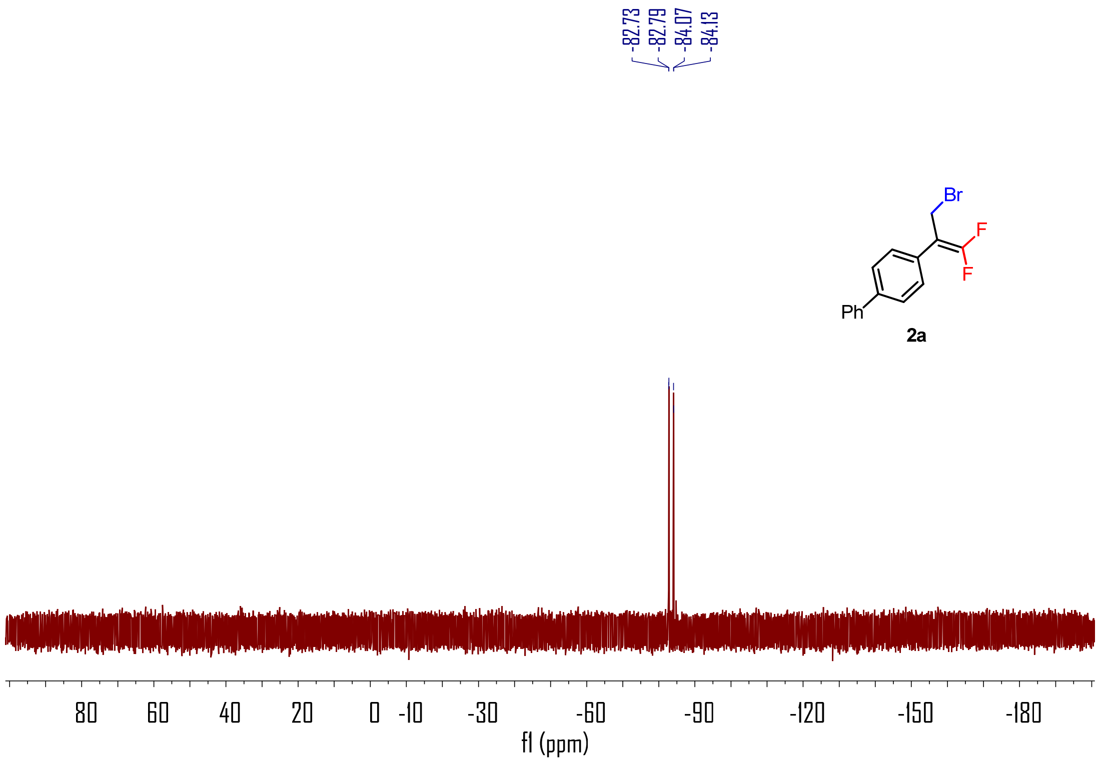

^{1}H NMR Spectrum (in $CDCl_3$, 400 MHz)

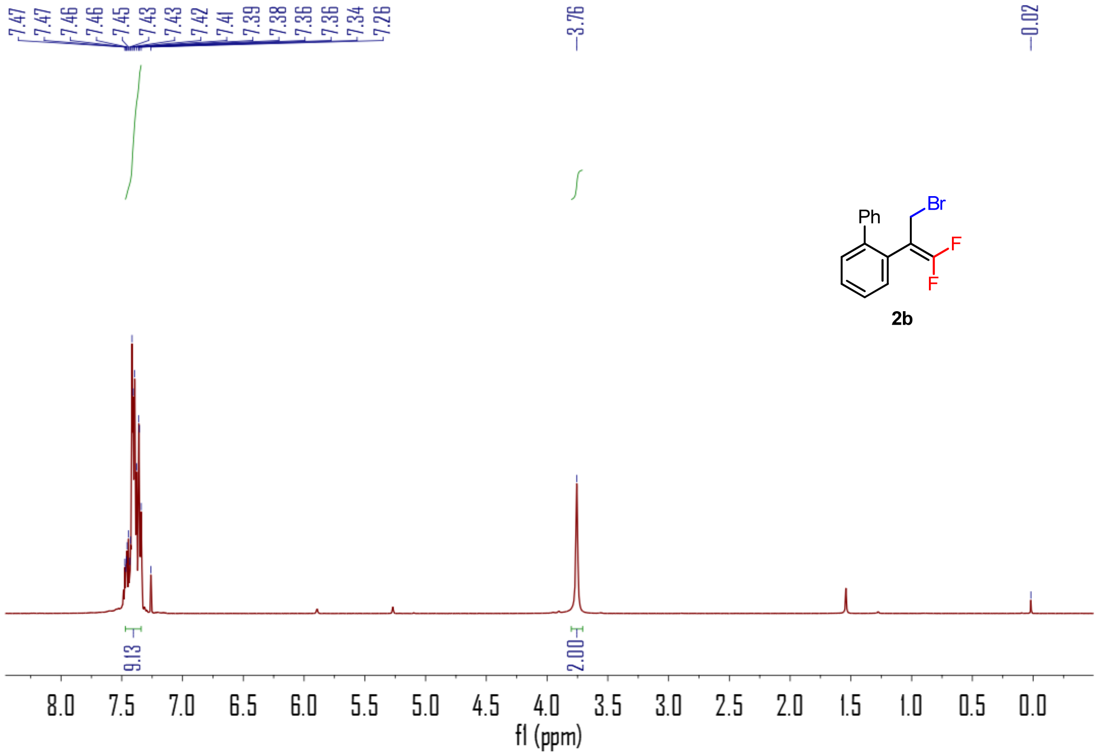

^{13}C NMR Spectrum (in $CDCl_3$, 101 MHz)

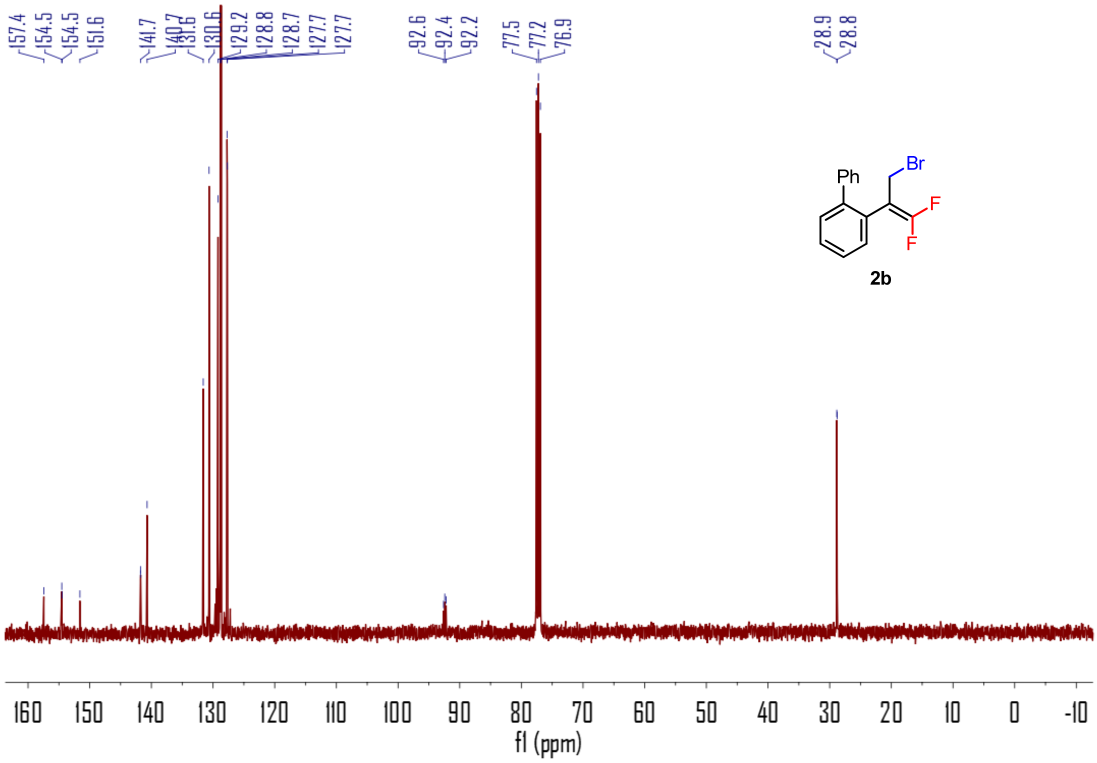

^{19}F NMR Spectrum (in $CDCl_3$, 376 MHz)

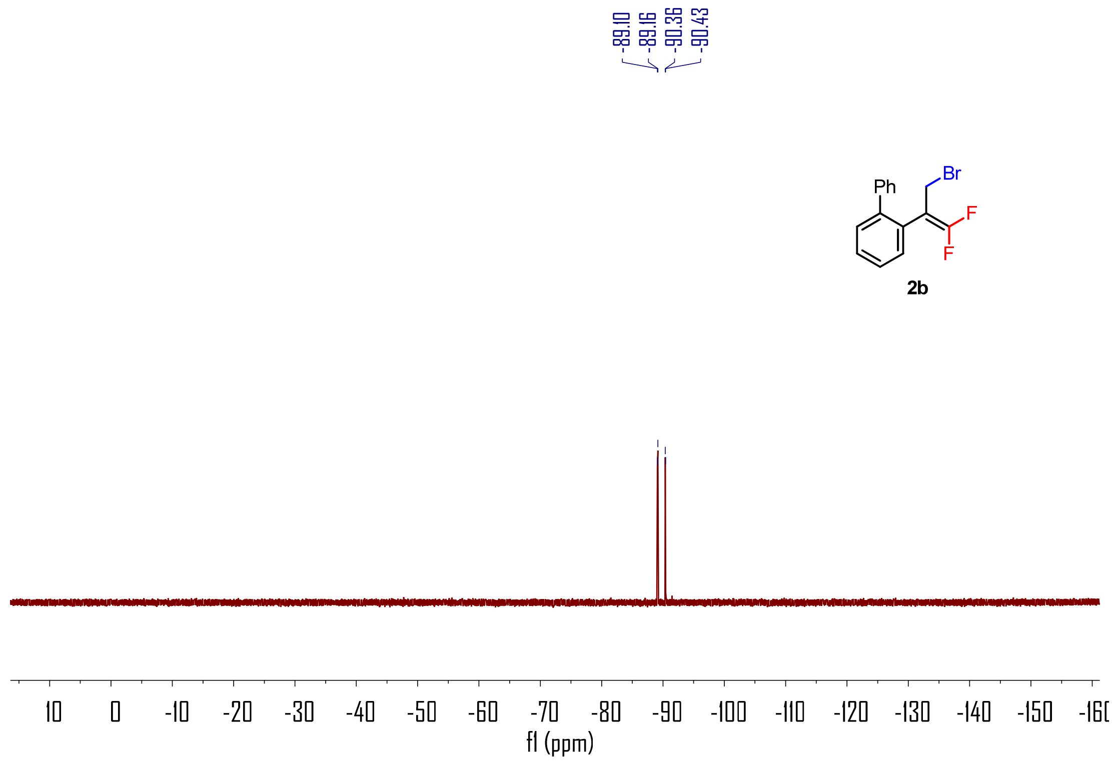

^{1}H NMR Spectrum (in $CDCl_3$, 400 MHz)

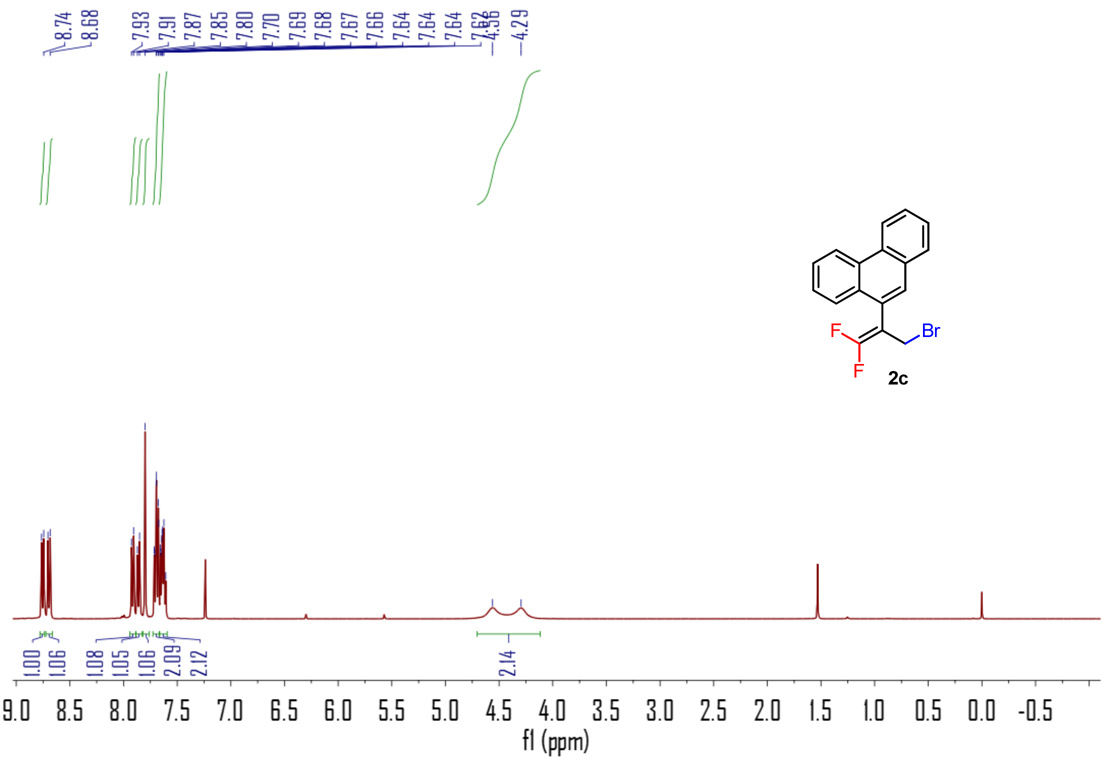

^{13}C NMR Spectrum (in $CDCl_3$, 101 MHz)

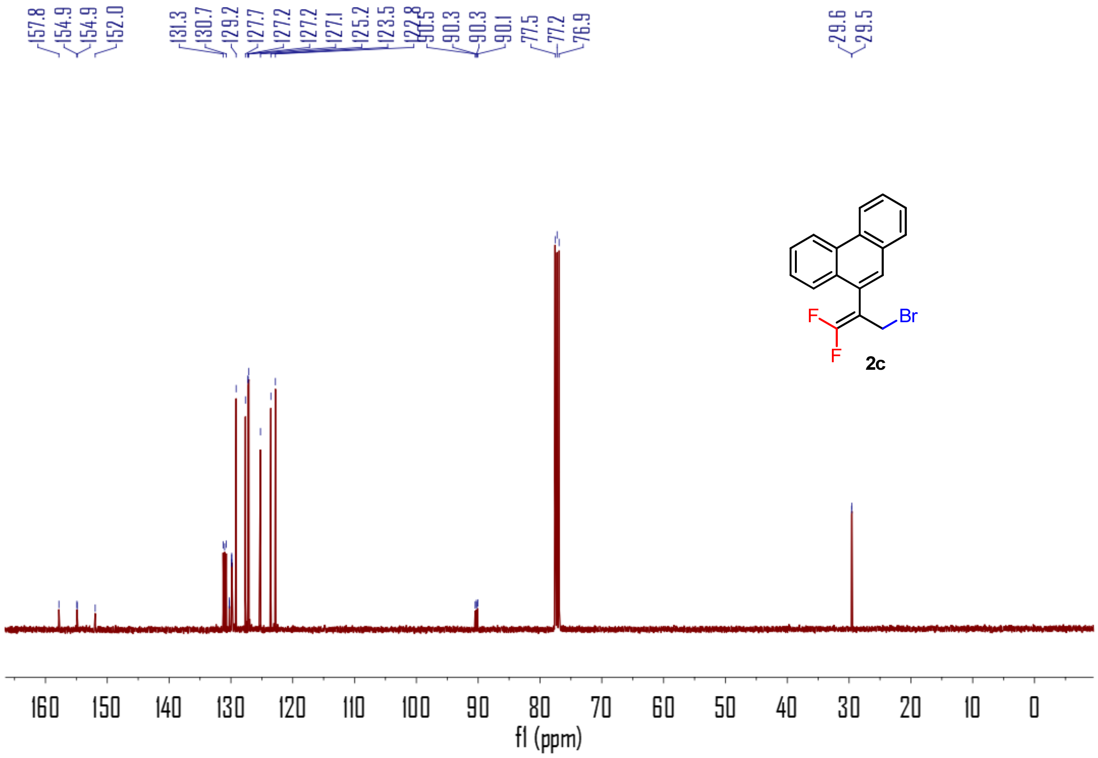

^{19}F NMR Spectrum (in $CDCl_3$, 376 MHz)

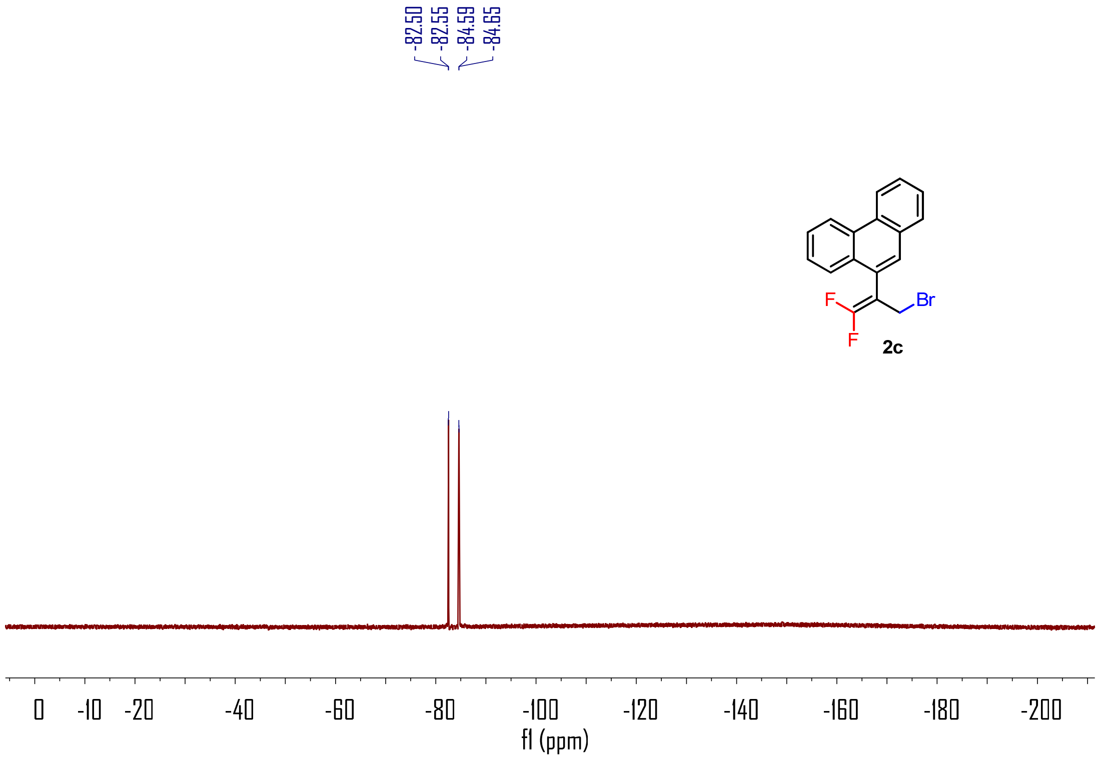

^{1}H NMR Spectrum (in $CDCl_3$, 400 MHz)

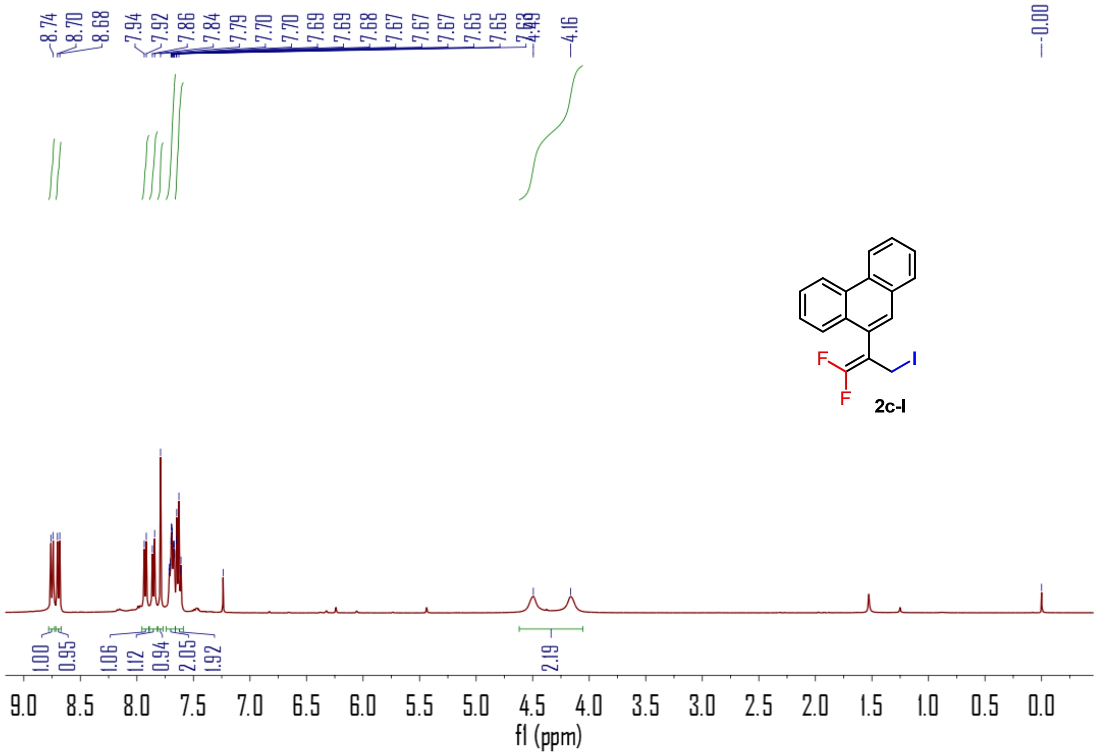

^{13}C NMR Spectrum (in $CDCl_3$, 101 MHz)

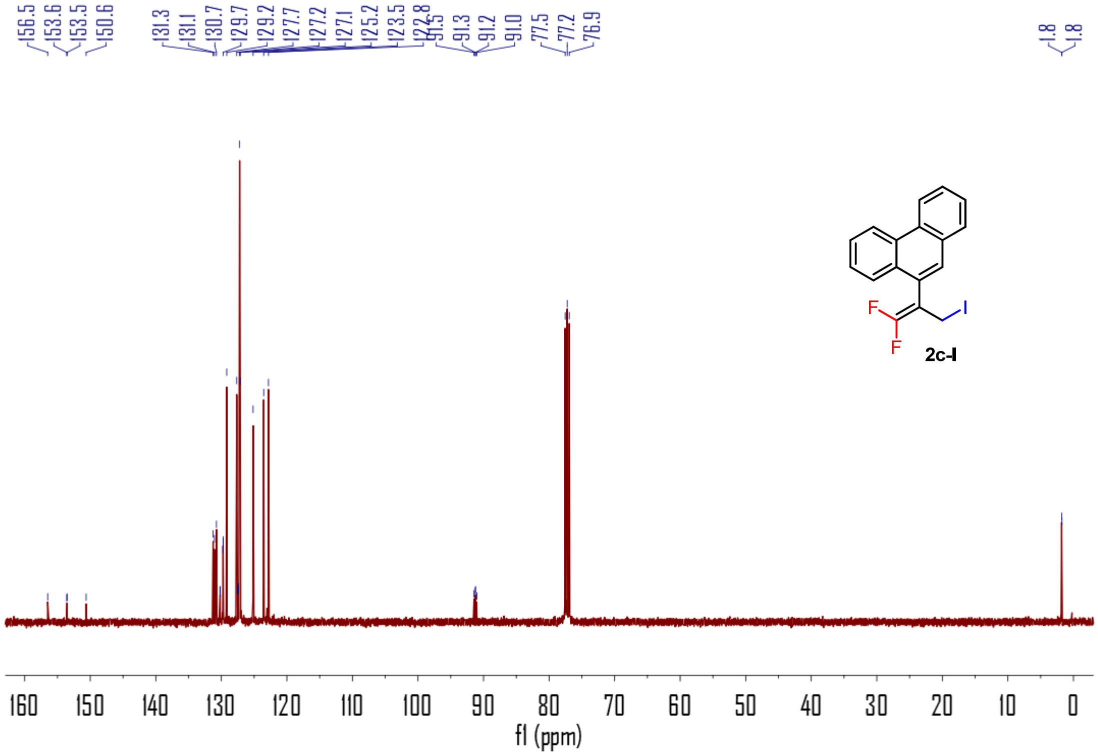

^{19}F NMR Spectrum (in $CDCl_3$, 376 MHz)

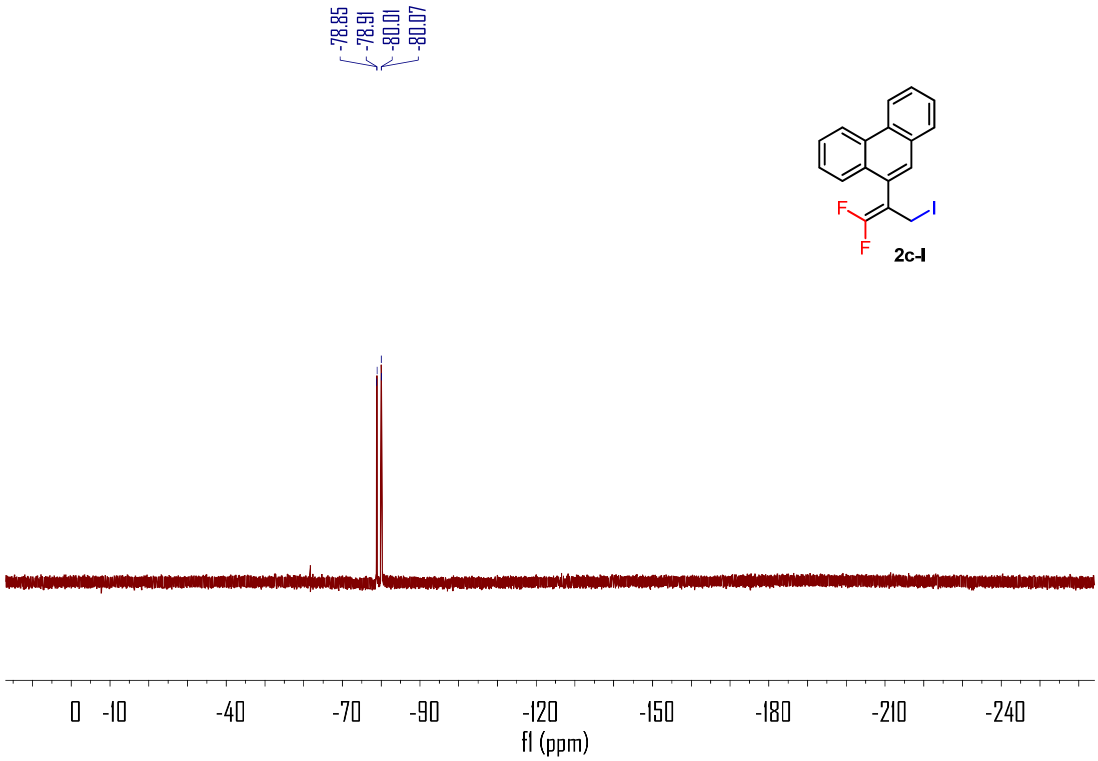

^{1}H NMR Spectrum (in $CDCl_3$, 400 MHz)

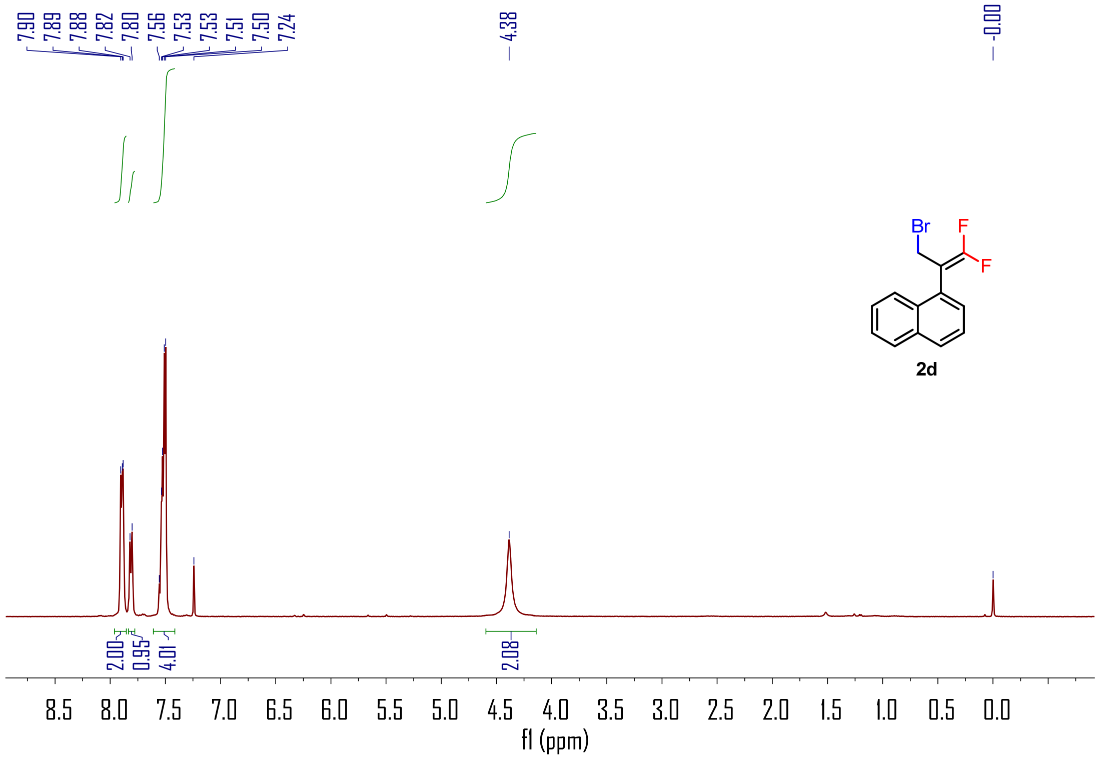

^{13}C NMR Spectrum (in $CDCl_3$, 101 MHz)

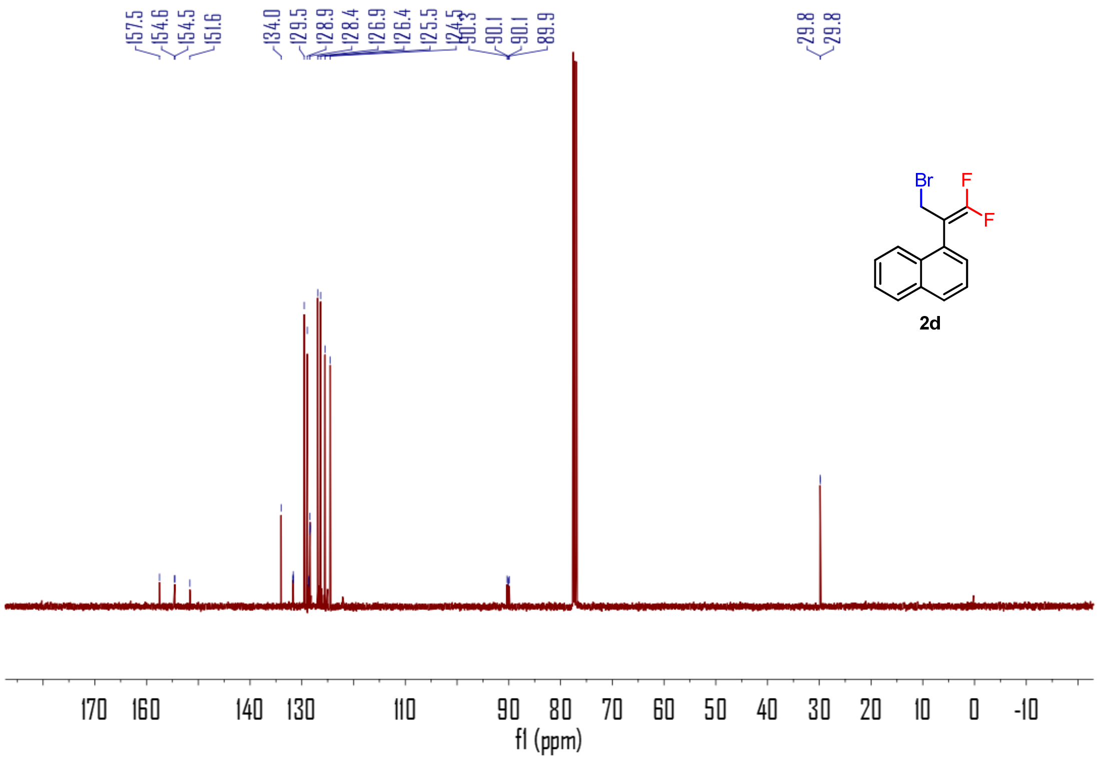

^{19}F NMR Spectrum (in $CDCl_3$, 376 MHz)

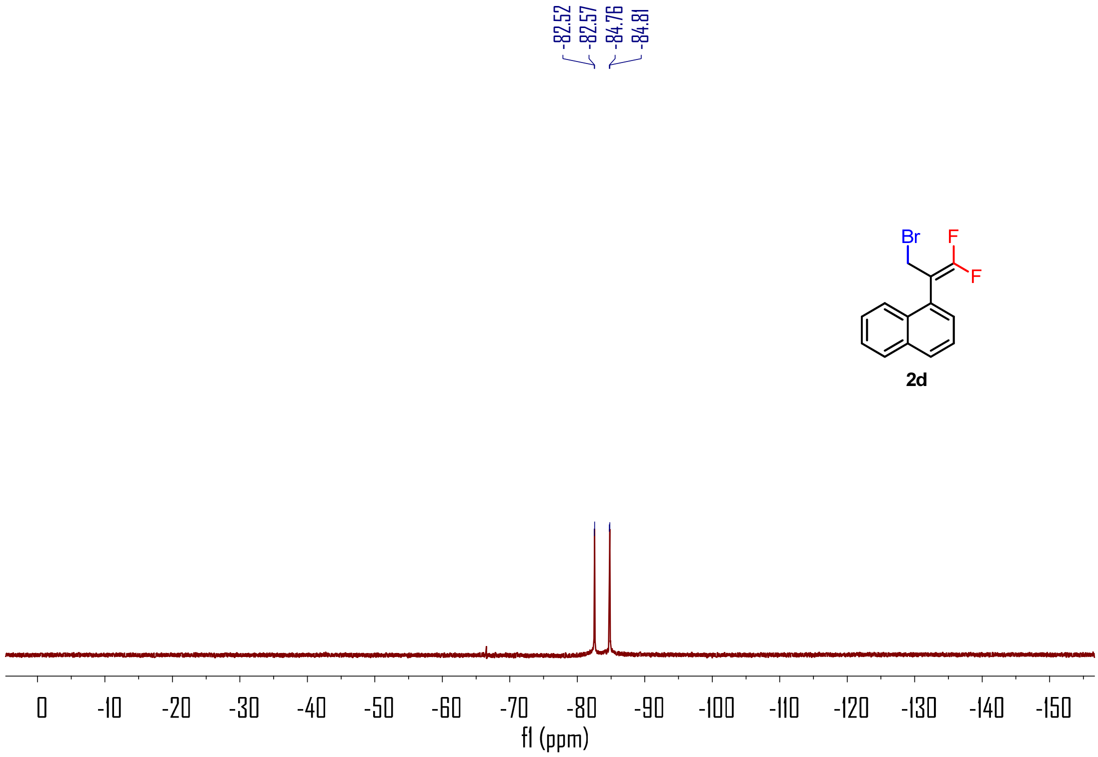

^{1}H NMR Spectrum (in $CDCl_3$, 400 MHz)

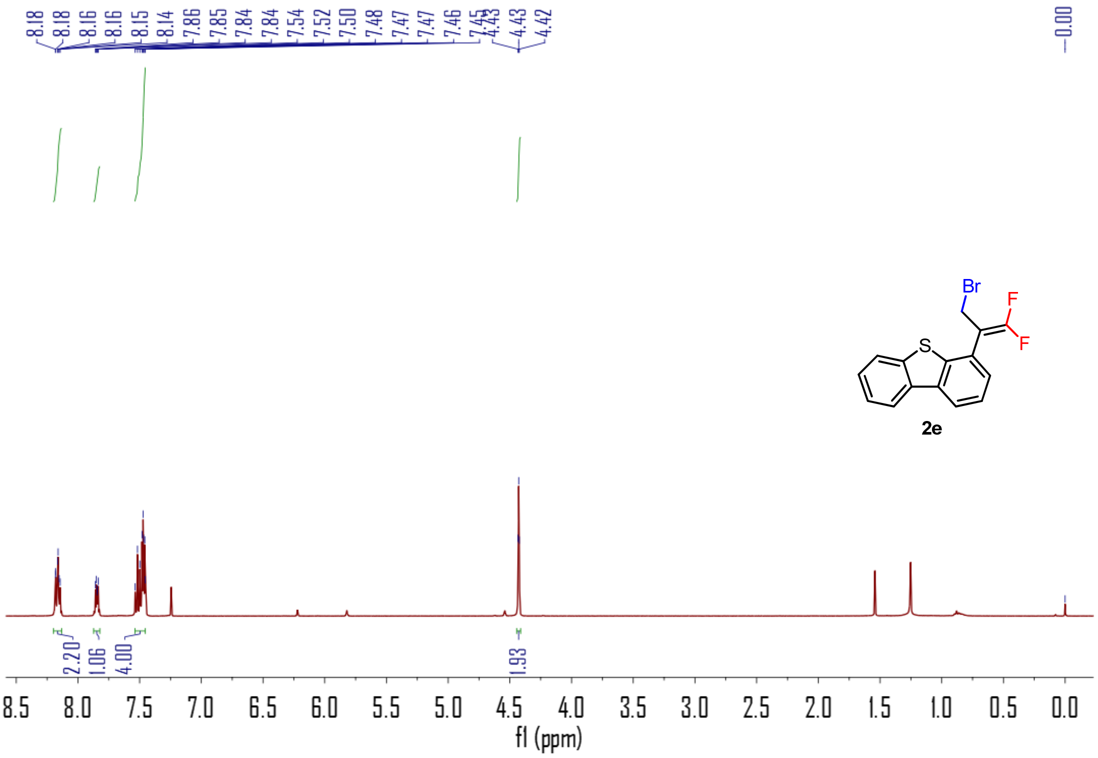

^{13}C NMR Spectrum (in $CDCl_3$, 101 MHz)

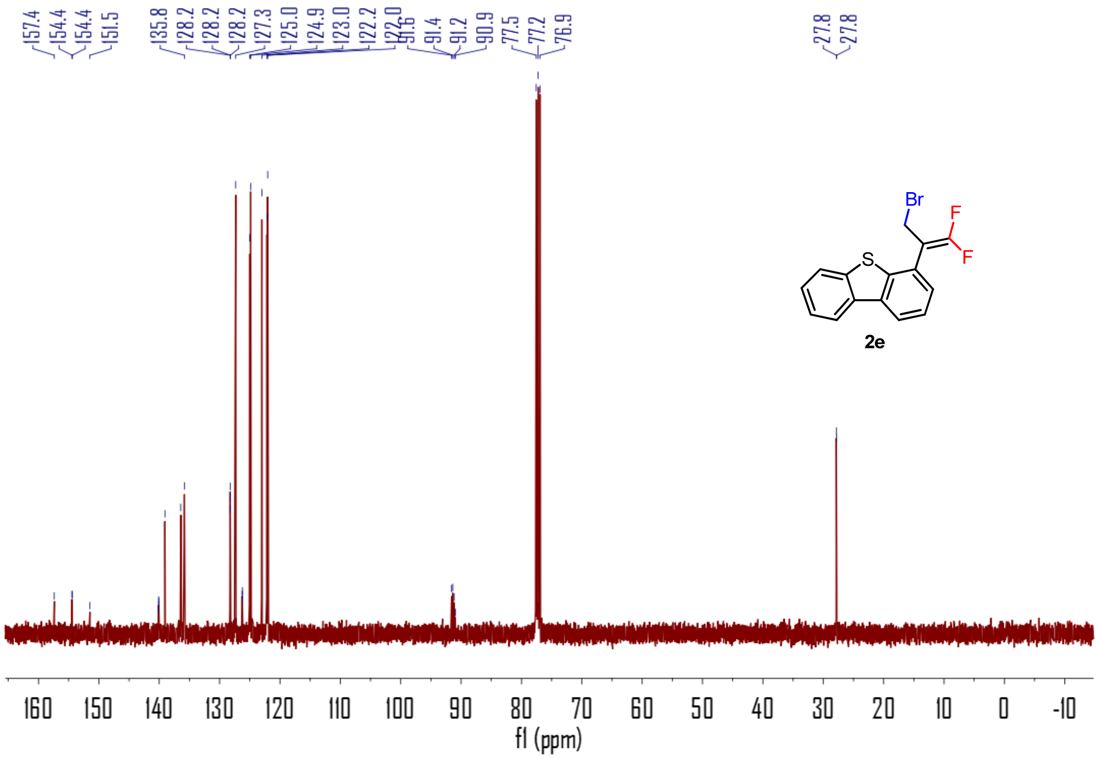

^{19}F NMR Spectrum (in $CDCl_3$, 376 MHz)

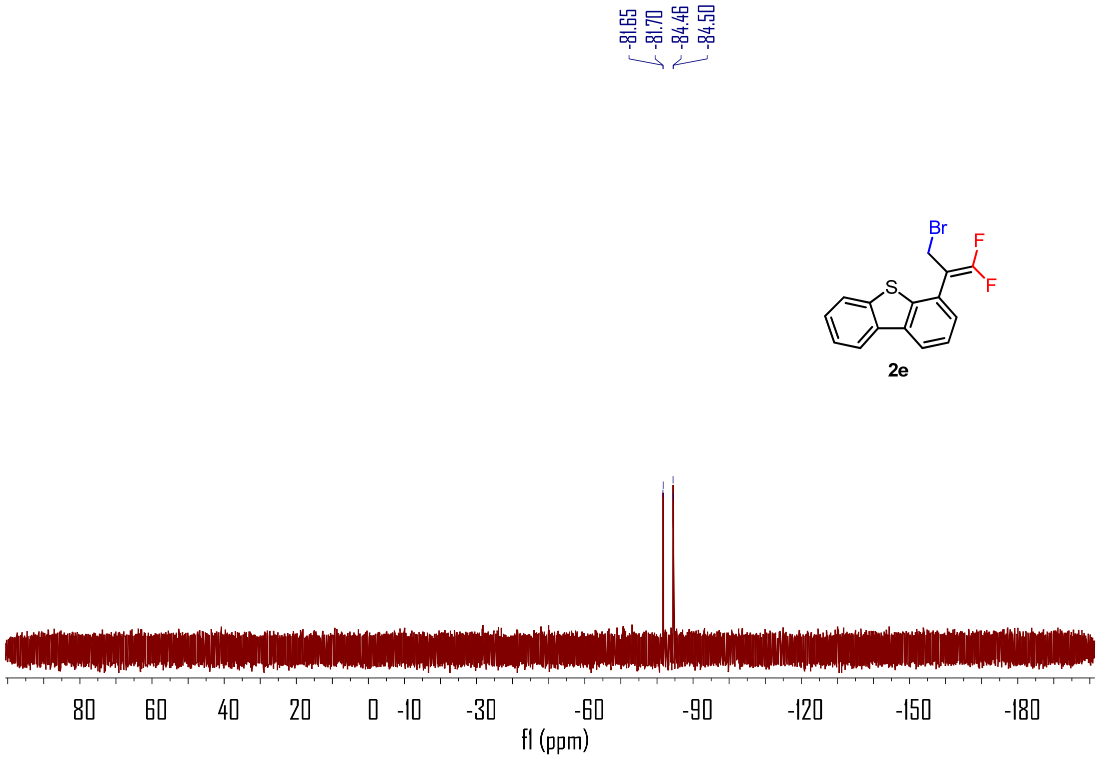

^{1}H NMR Spectrum (in $CDCl_3$, 400 MHz)

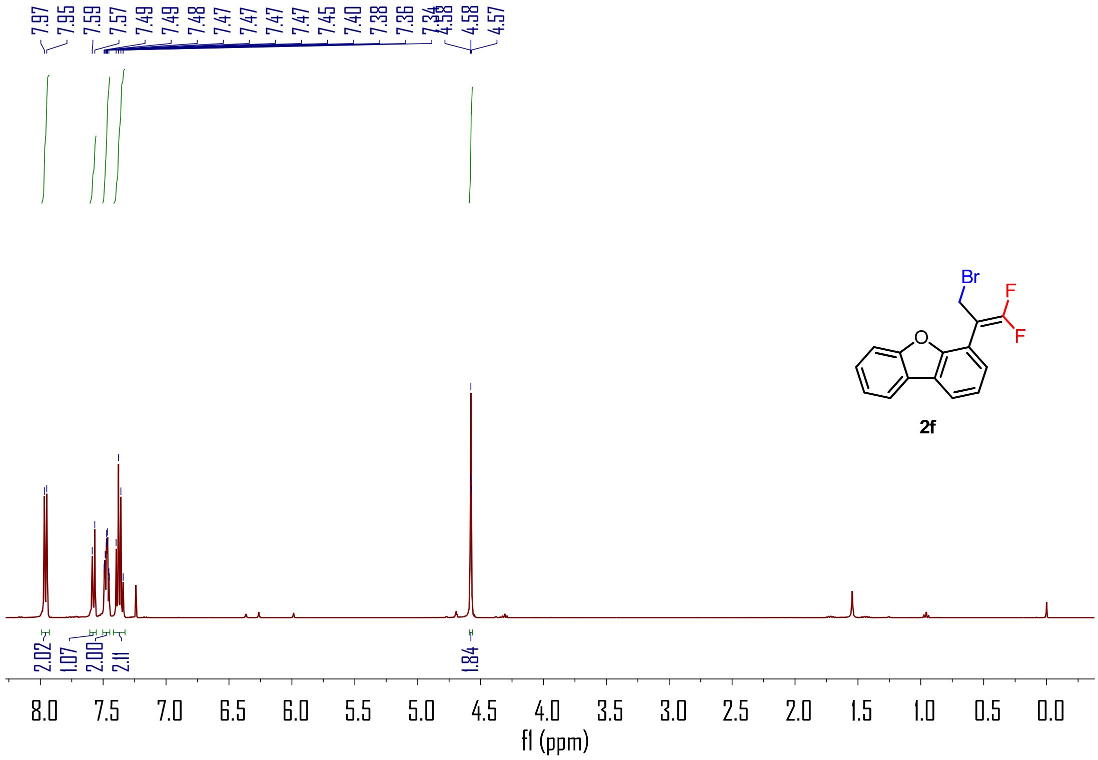

^{13}C NMR Spectrum (in $CDCl_3$, 101 MHz)

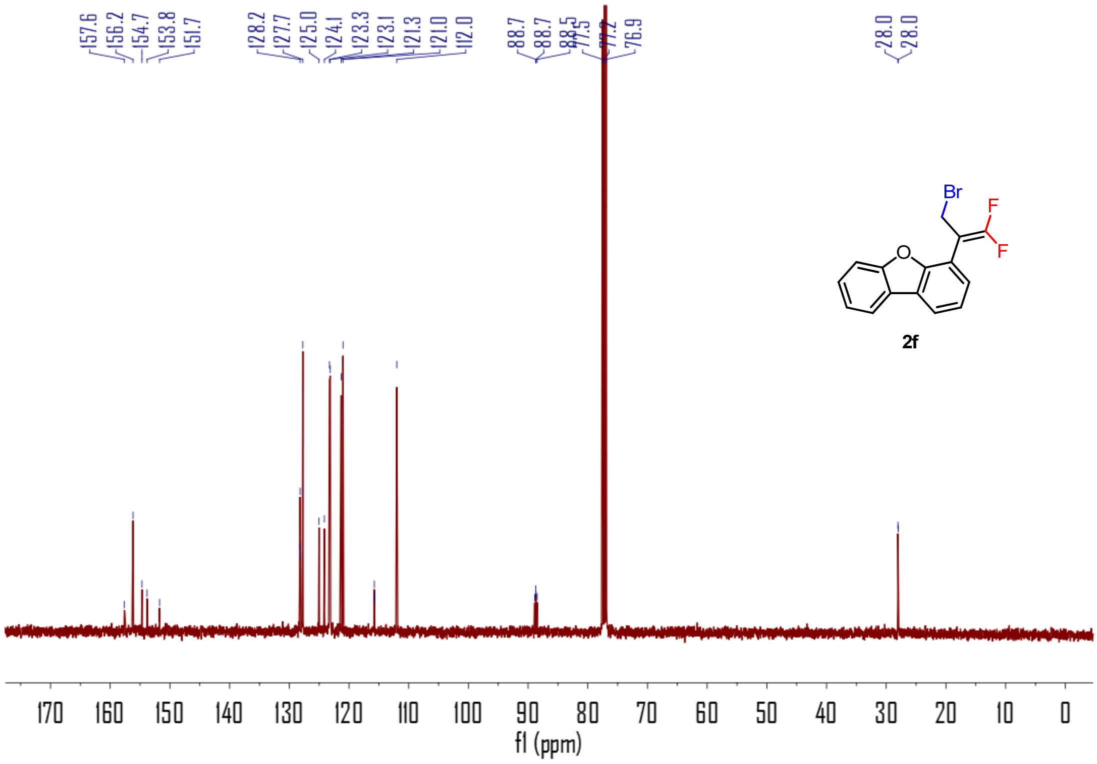

^{19}F NMR Spectrum (in $CDCl_3$, 376 MHz)

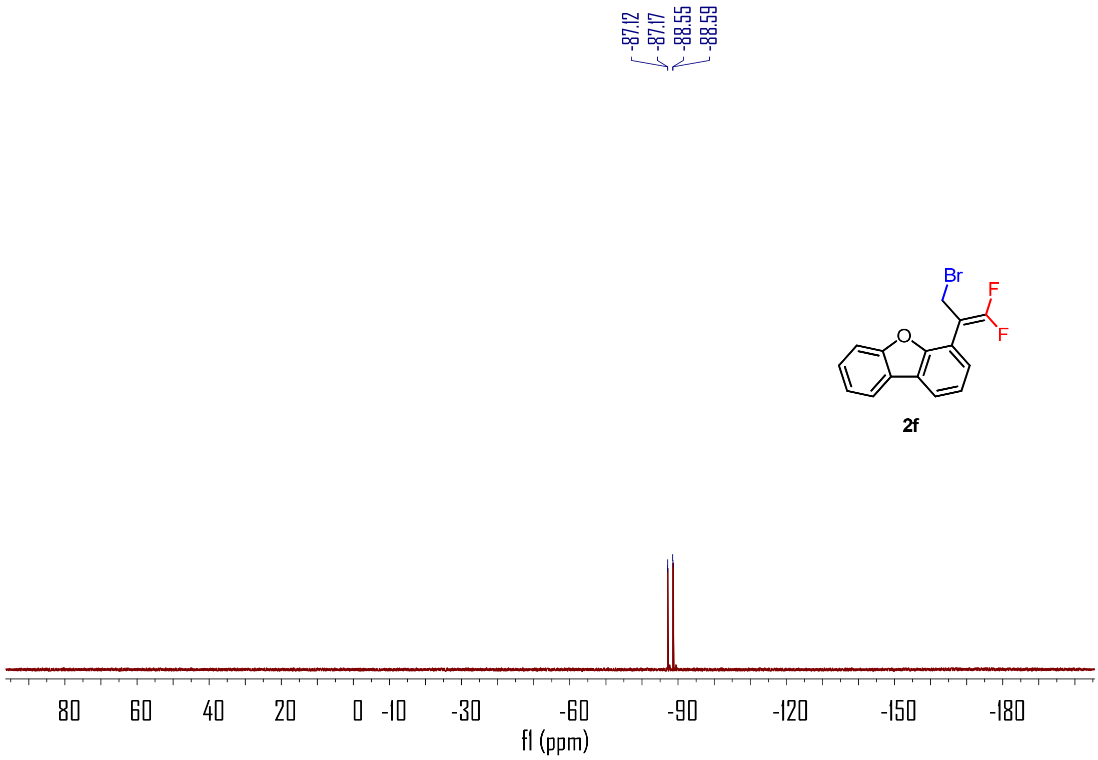

^{1}H NMR Spectrum (in $CDCl_3$, 400 MHz)

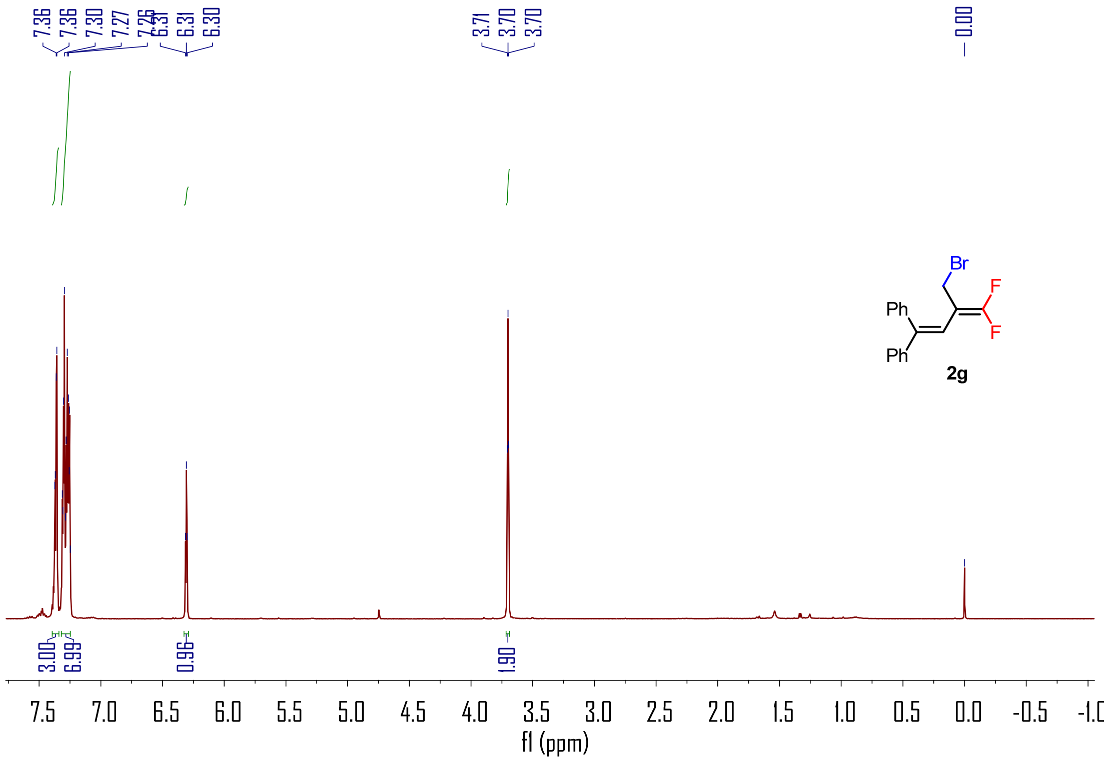

^{13}C NMR Spectrum (in $CDCl_3$, 101 MHz)

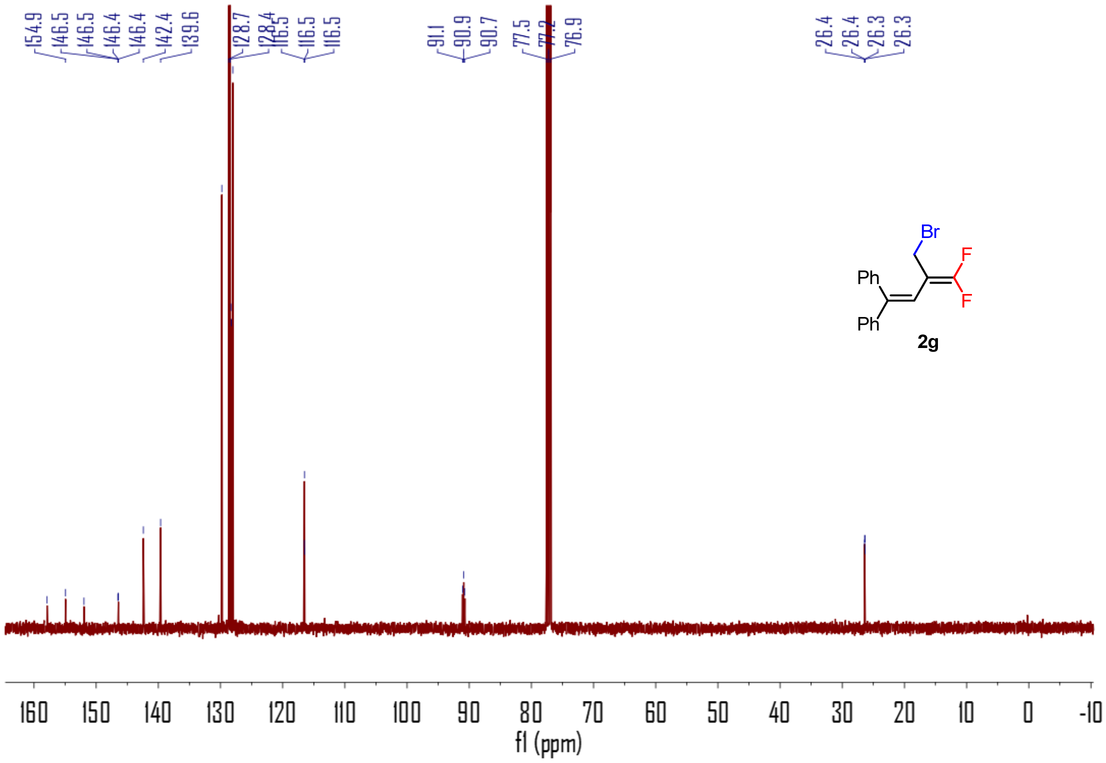

^{19}F NMR Spectrum (in $CDCl_3$, 376 MHz)

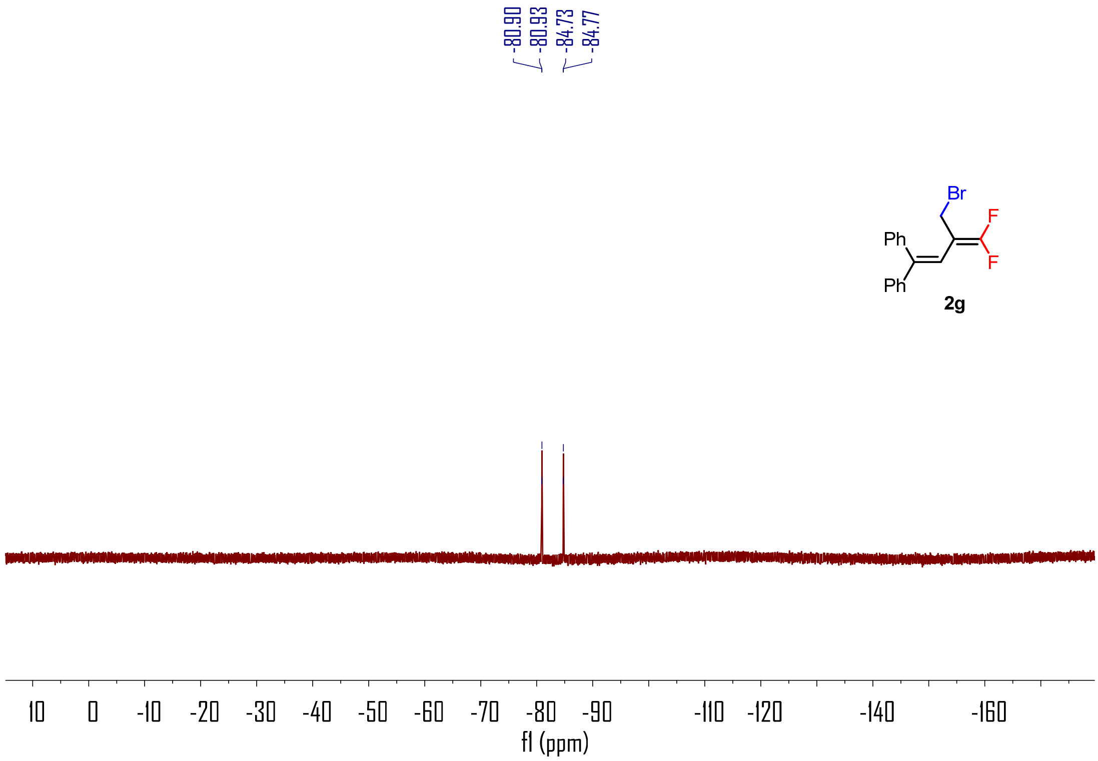

^{1}H NMR Spectrum (in $CDCl_3$, 400 MHz)

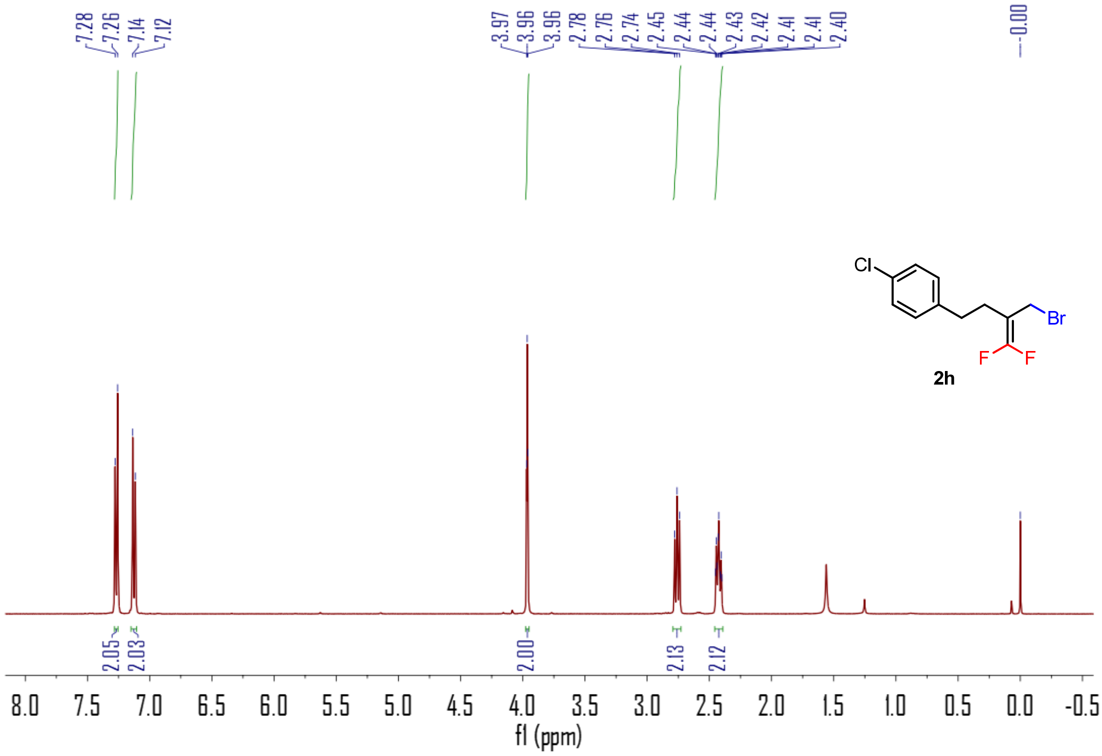

^{13}C NMR Spectrum (in CDCl3, 101 MHz)

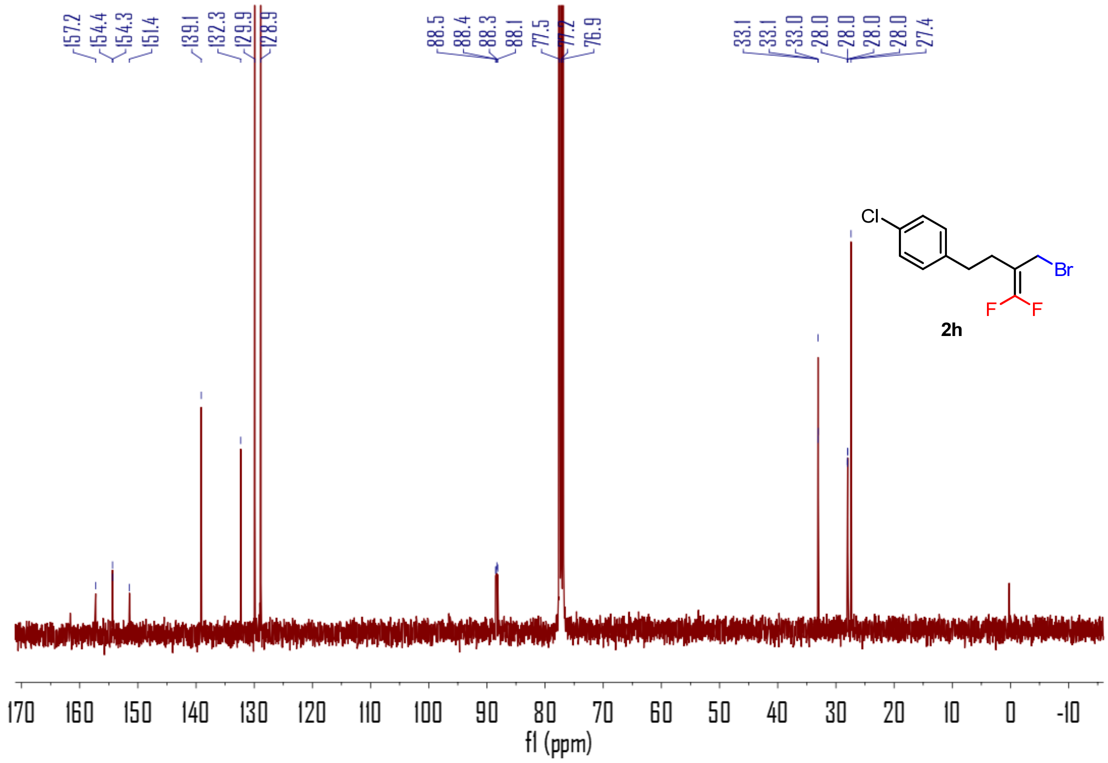

^{19}F NMR Spectrum (in CDCl3, 376 MHz)

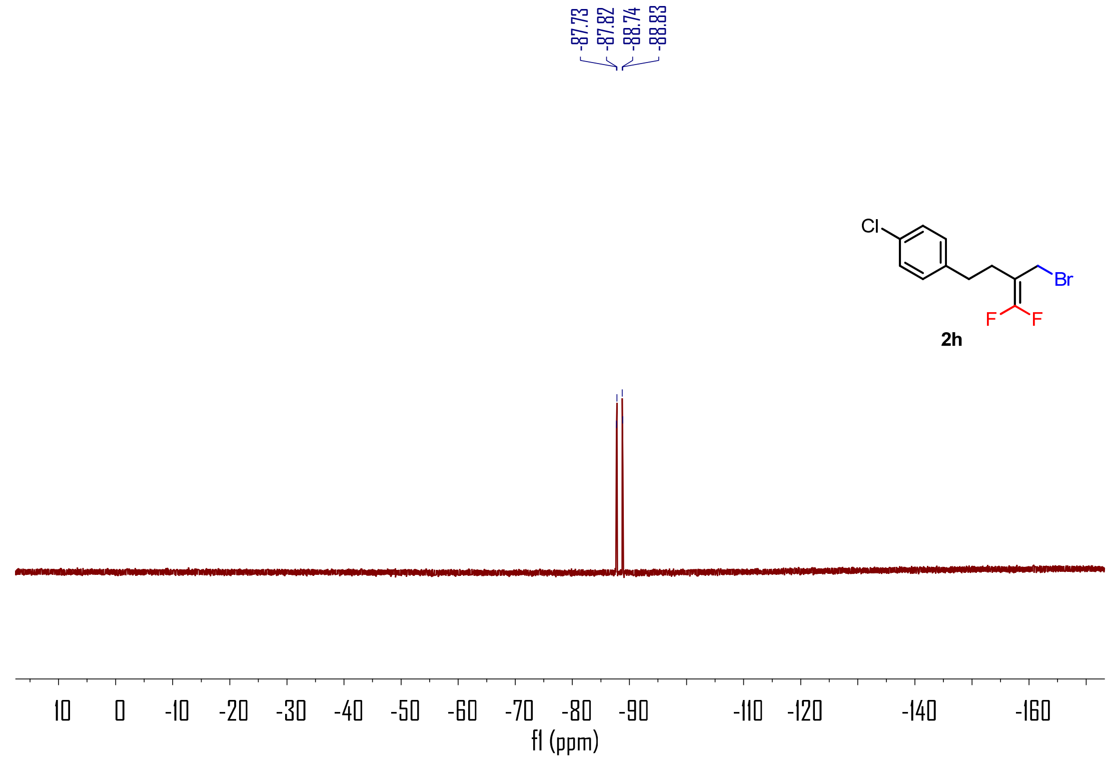

^{1}H NMR Spectrum (in CDCl3, 400 MHz)

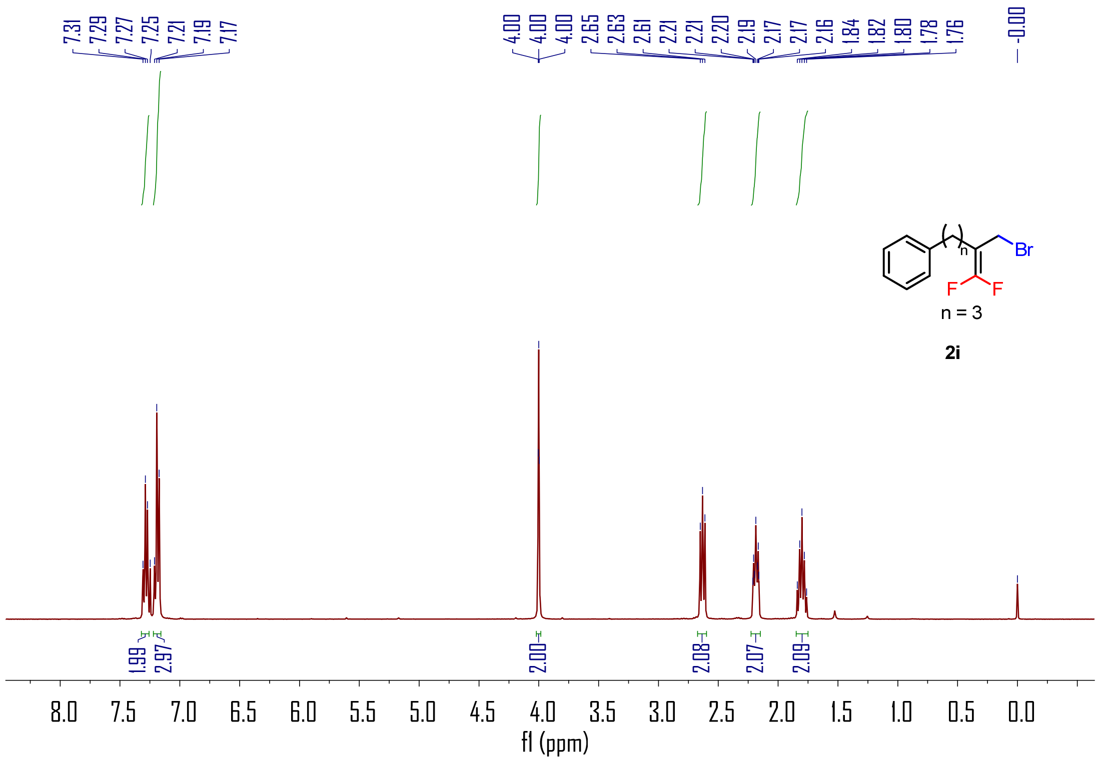

^{13}C NMR Spectrum (in CDCl3, 101 MHz)

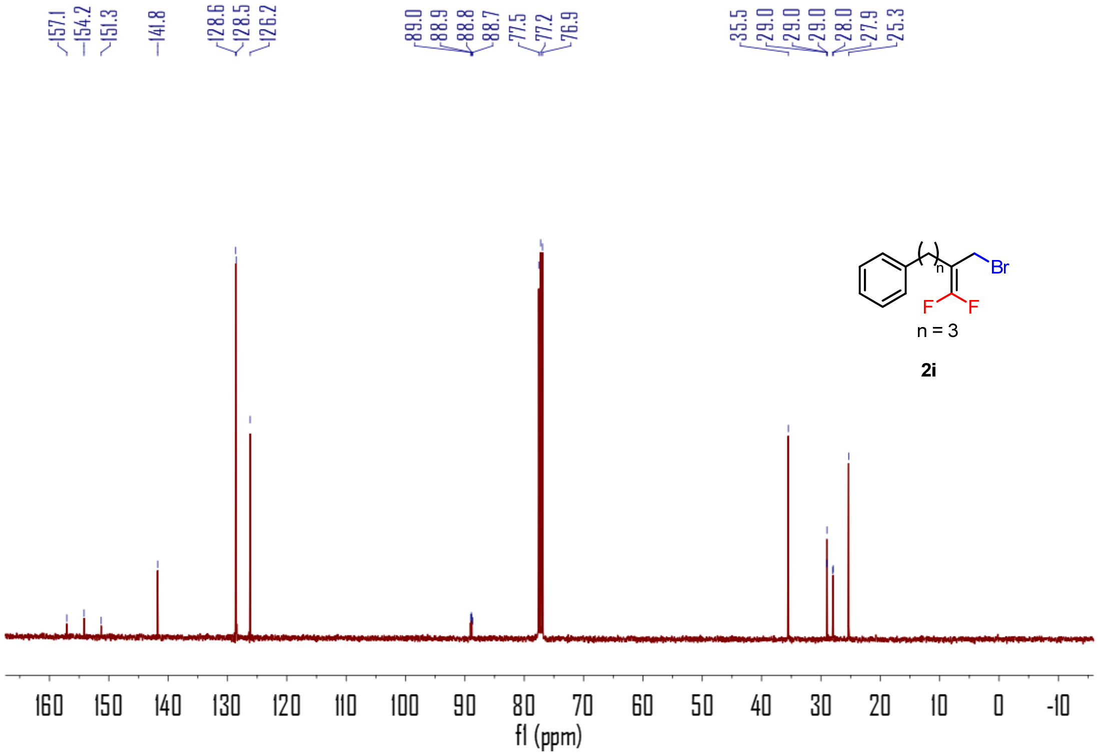

^{19}F NMR Spectrum (in $CDCl_3$, 376 MHz)

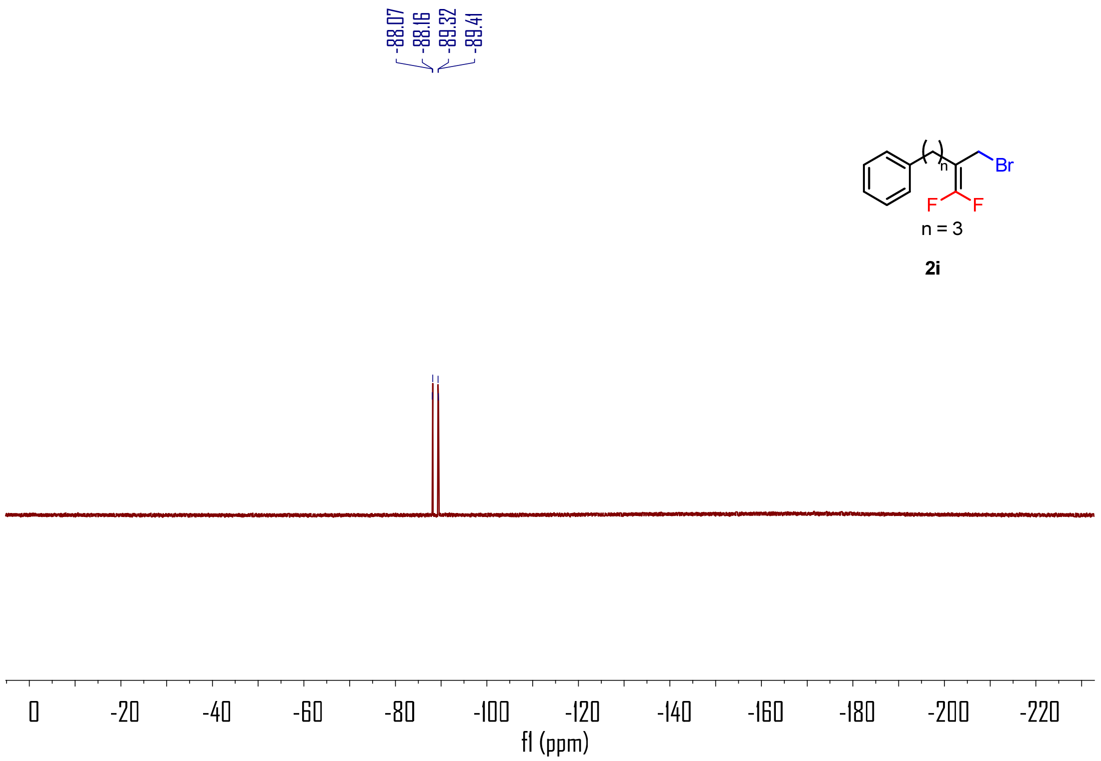

^{1}H NMR Spectrum (in $CDCl_3$, 400 MHz)

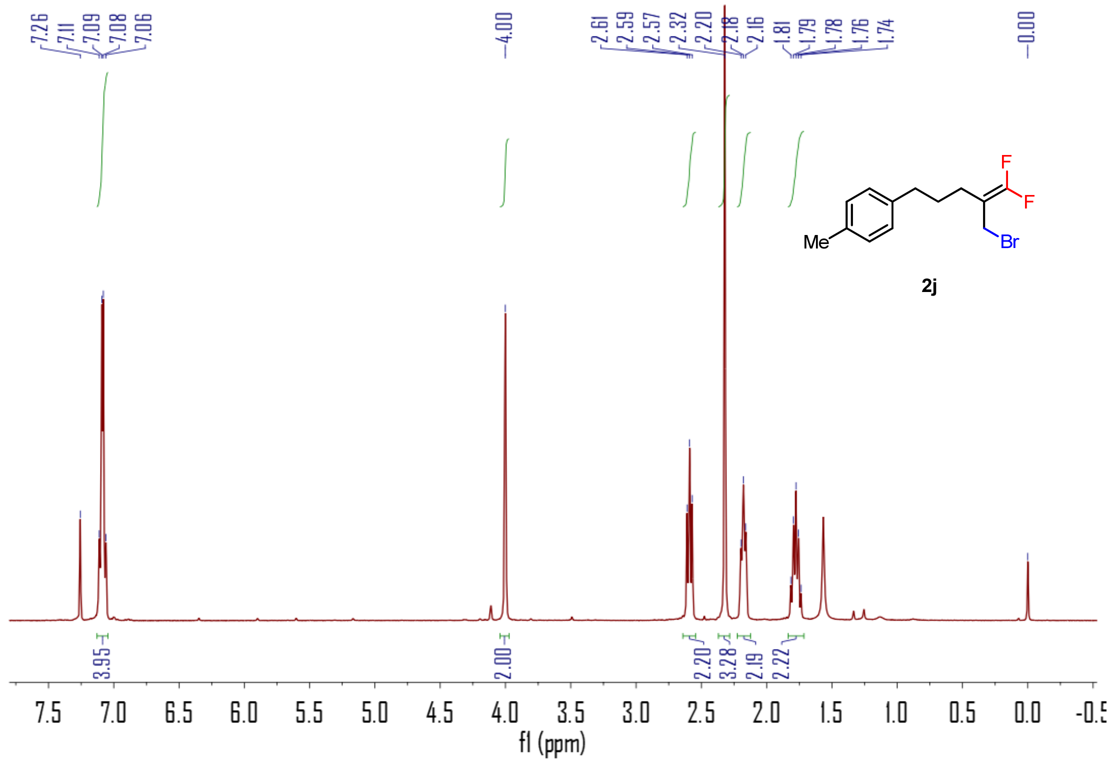

^{13}C NMR Spectrum (in $CDCl_3$, 101 MHz)

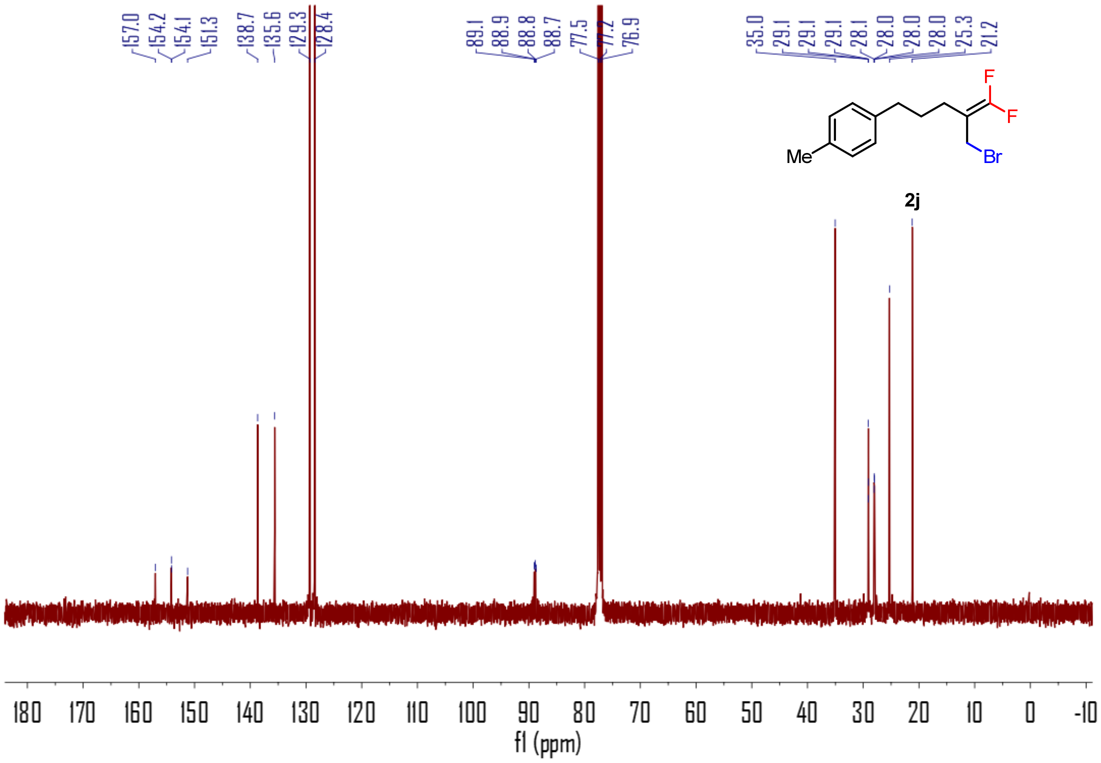

^{19}F NMR Spectrum (in $CDCl_3$, 376 MHz)

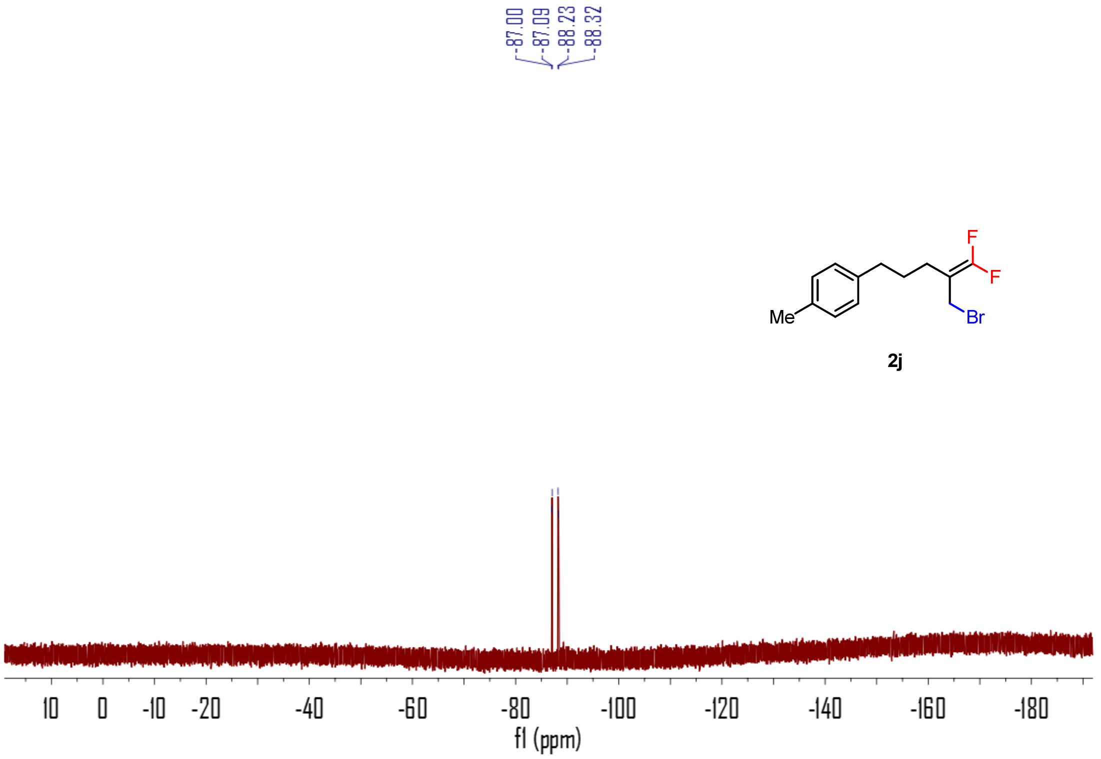

^{1}H NMR Spectrum (in $CDCl_3$, 400 MHz)

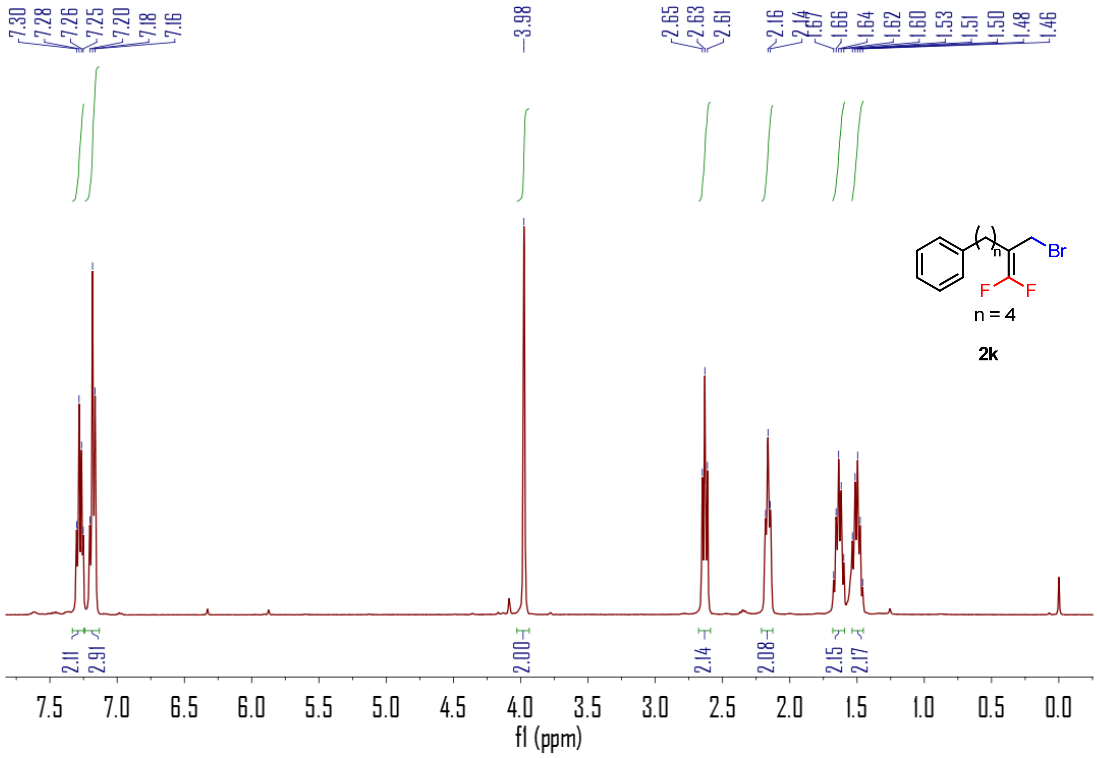

^{13}C NMR Spectrum (in $CDCl_3$, 101 MHz)

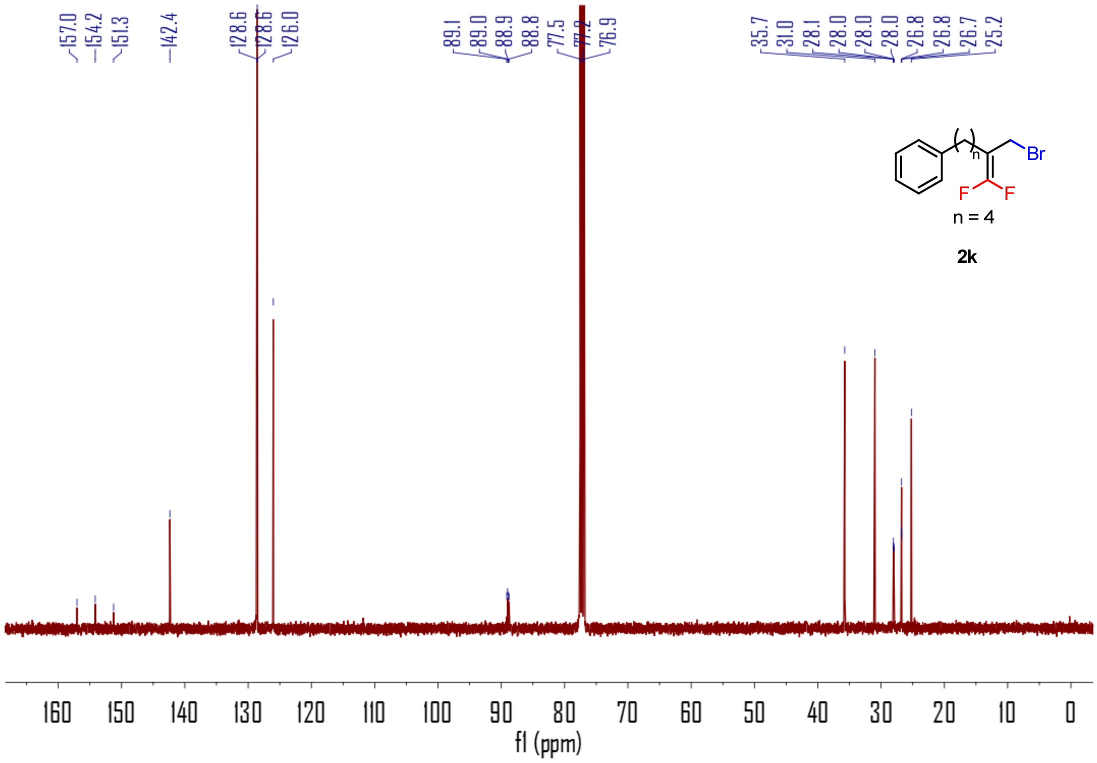

^{19}F NMR Spectrum (in $CDCl_3$, 376 MHz)

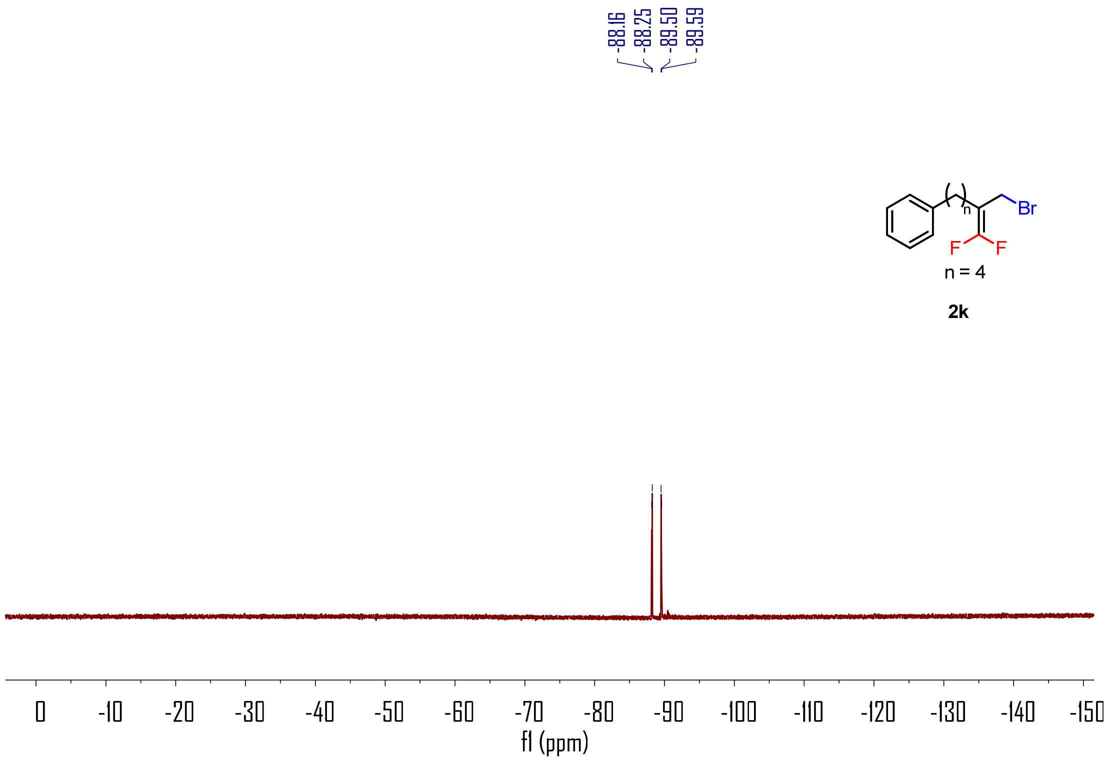

^{1}H NMR Spectrum (in $CDCl_3$, 400 MHz)

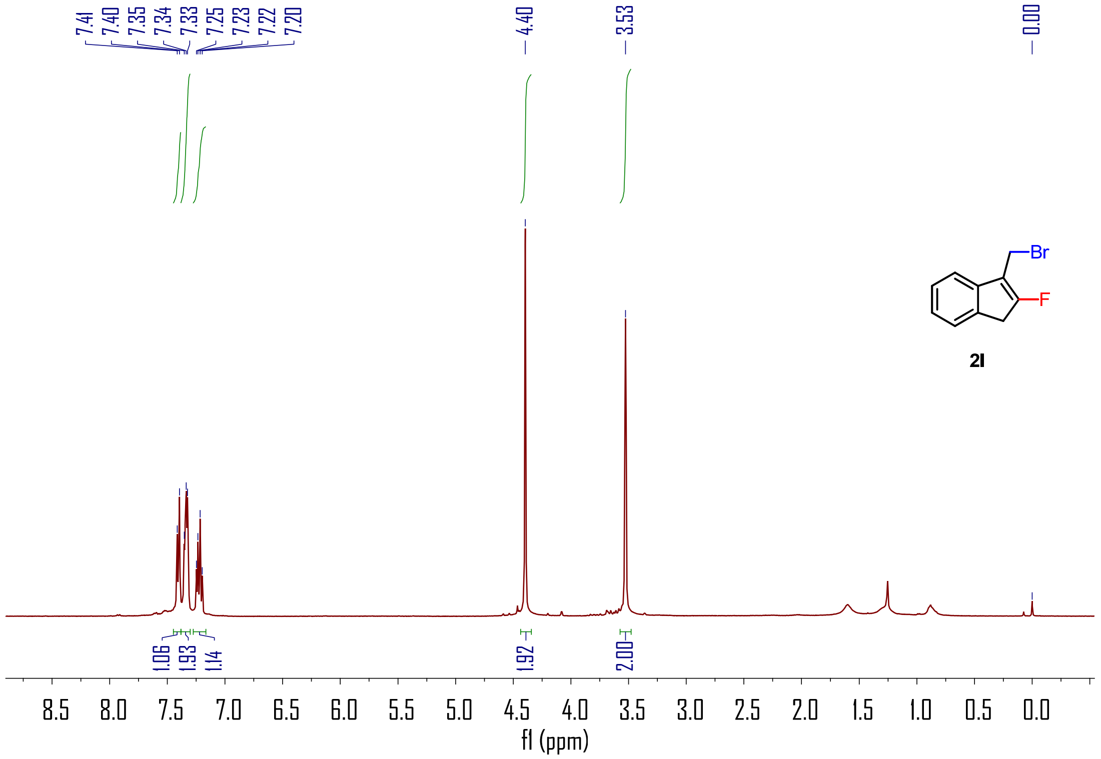

^{13}C NMR Spectrum (in $CDCl_3$, 101 MHz)

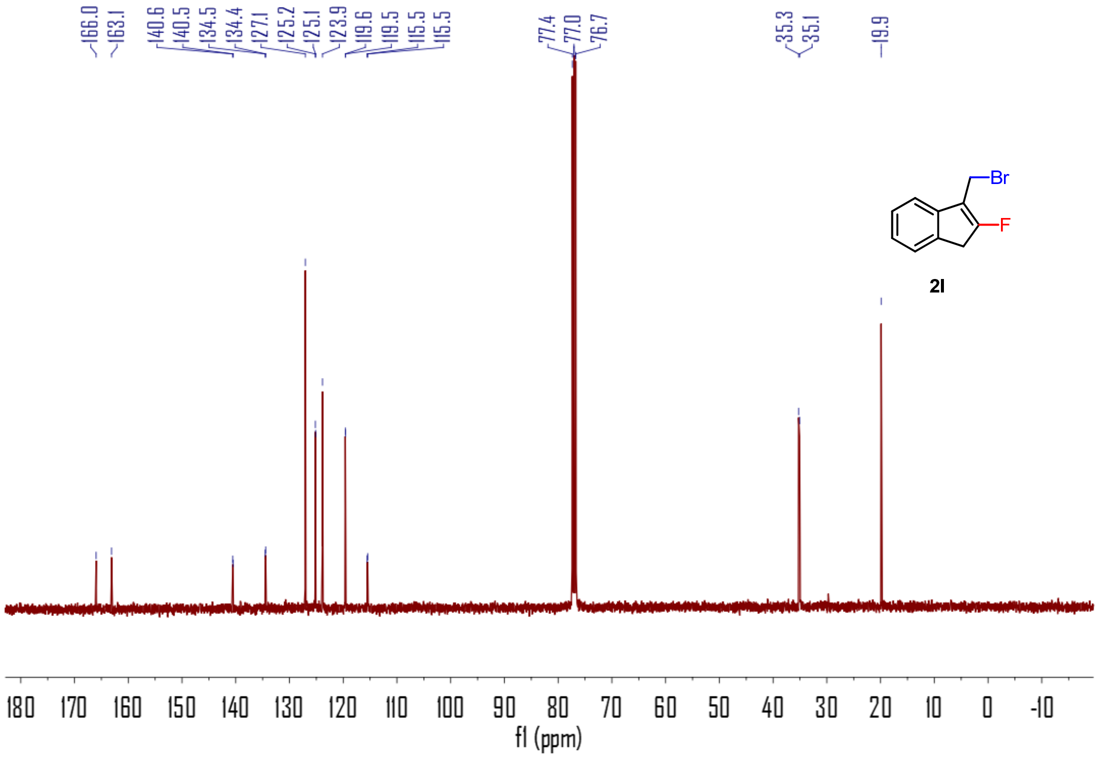

^{19}F NMR Spectrum (in $CDCl_3$, 376 MHz)

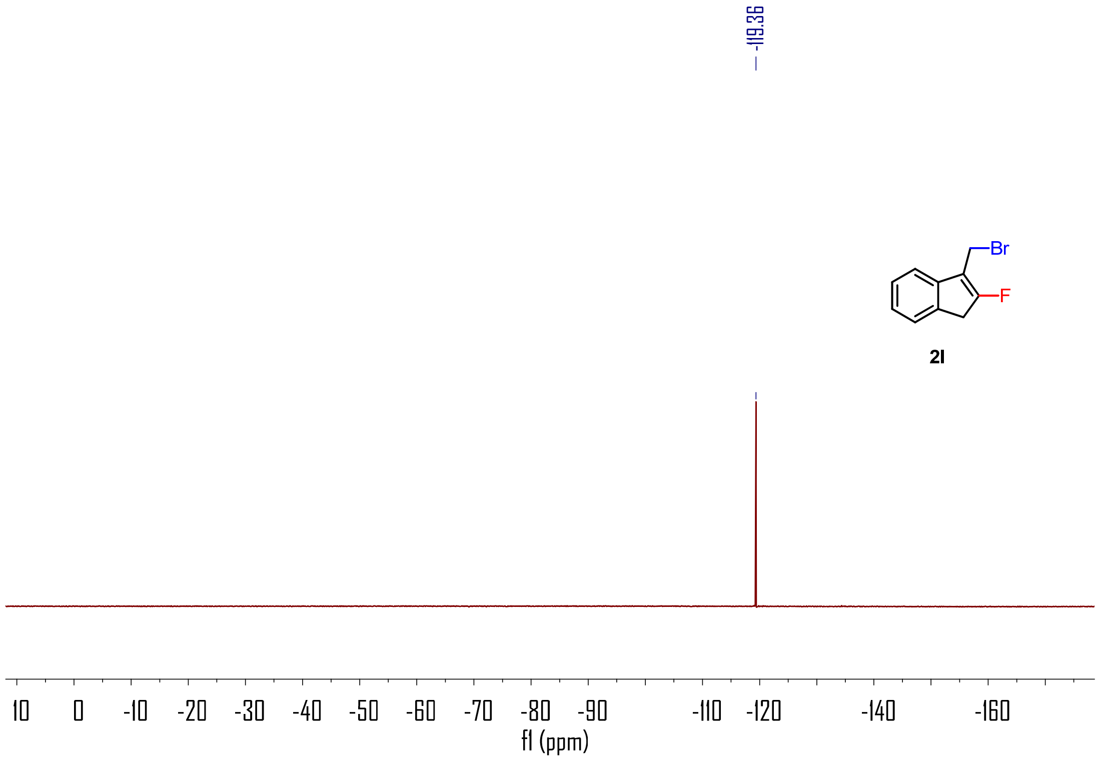

^{1}H NMR Spectrum (in $CDCl_3$, 400 MHz)

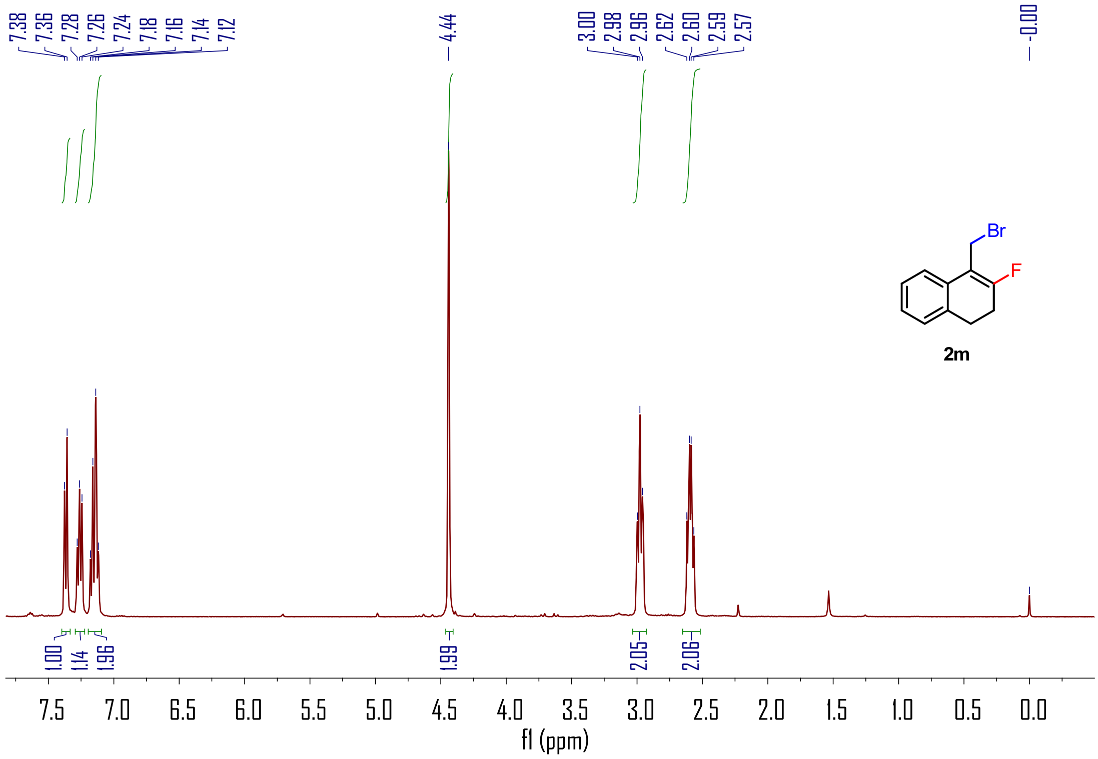

^{13}C NMR Spectrum (in $CDCl_3$, 101 MHz)

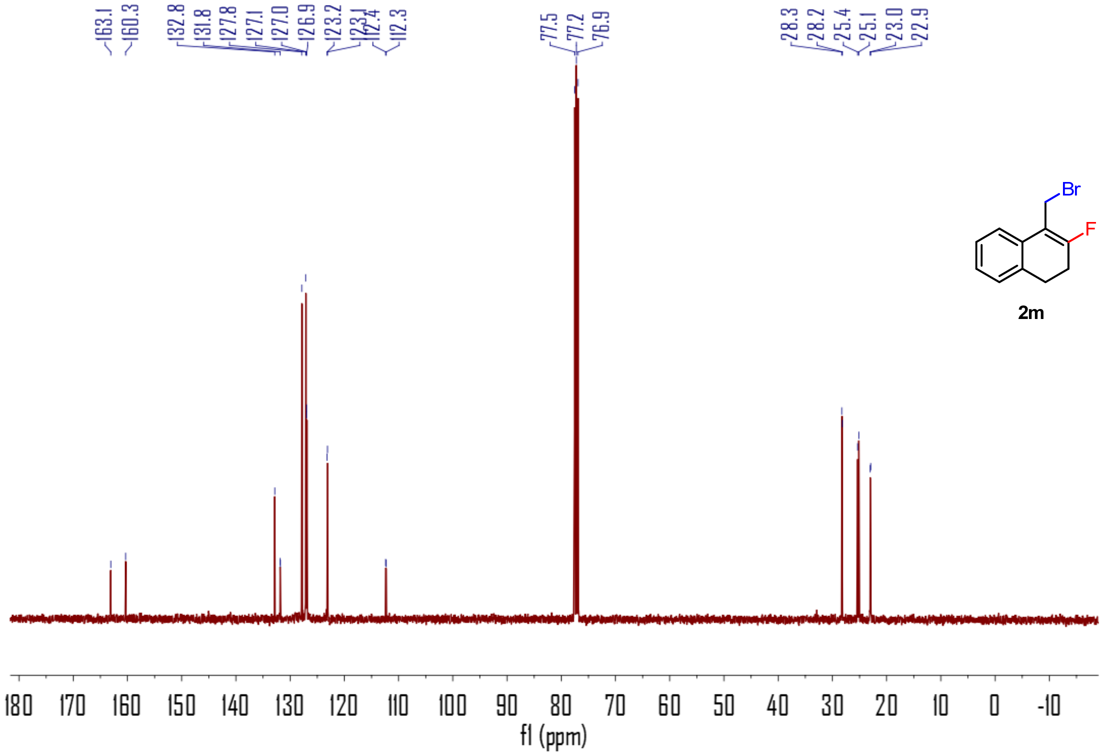

^{19}F NMR Spectrum (in $CDCl_3$, 376 MHz)

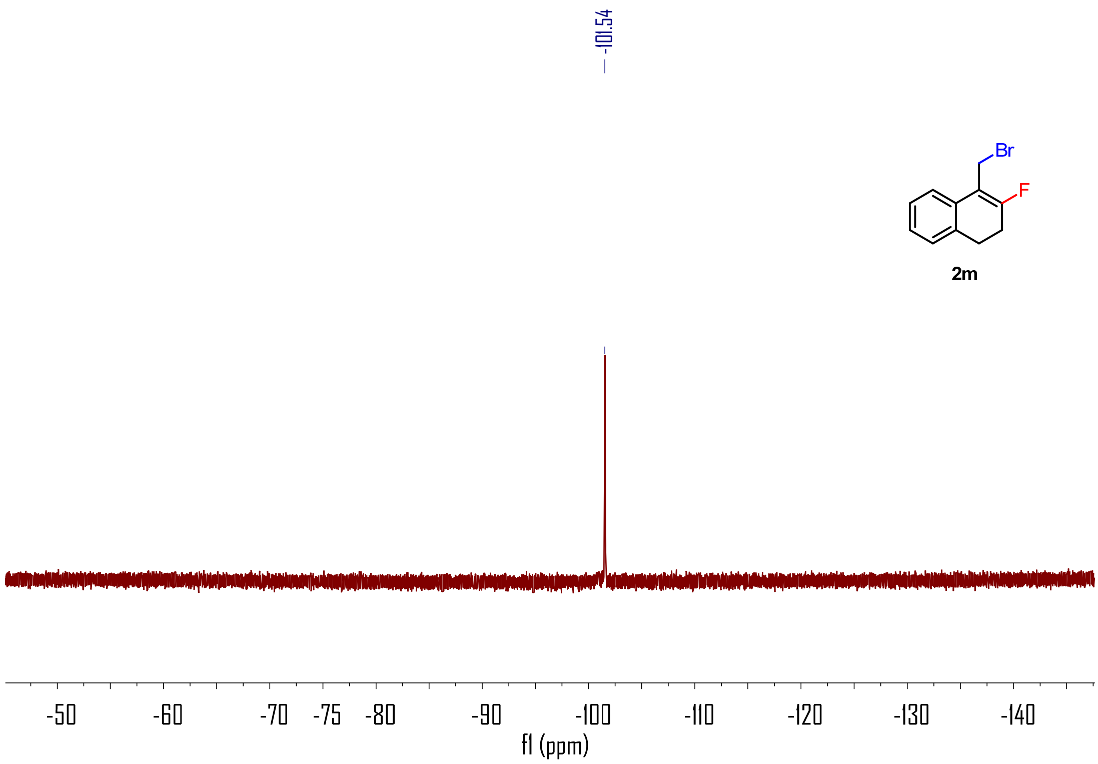

^{1}H NMR Spectrum (in $CDCl_3$, 400 MHz)

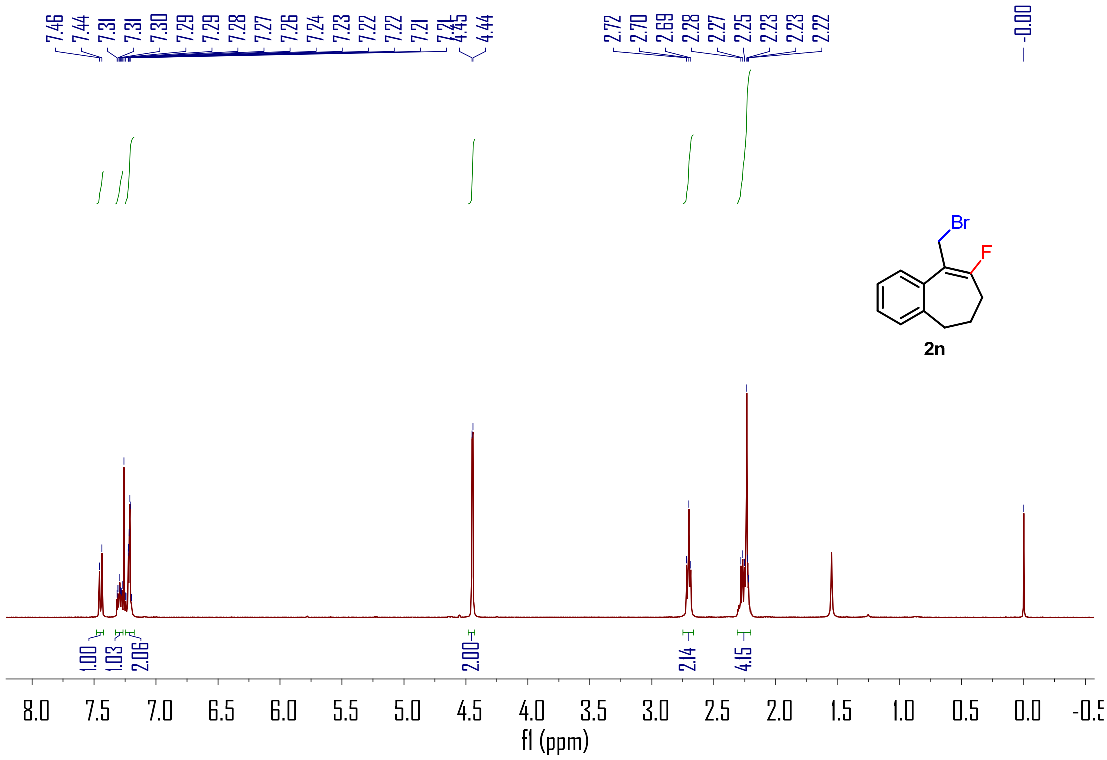

^{13}C NMR Spectrum (in $CDCl_3$, 101 MHz)

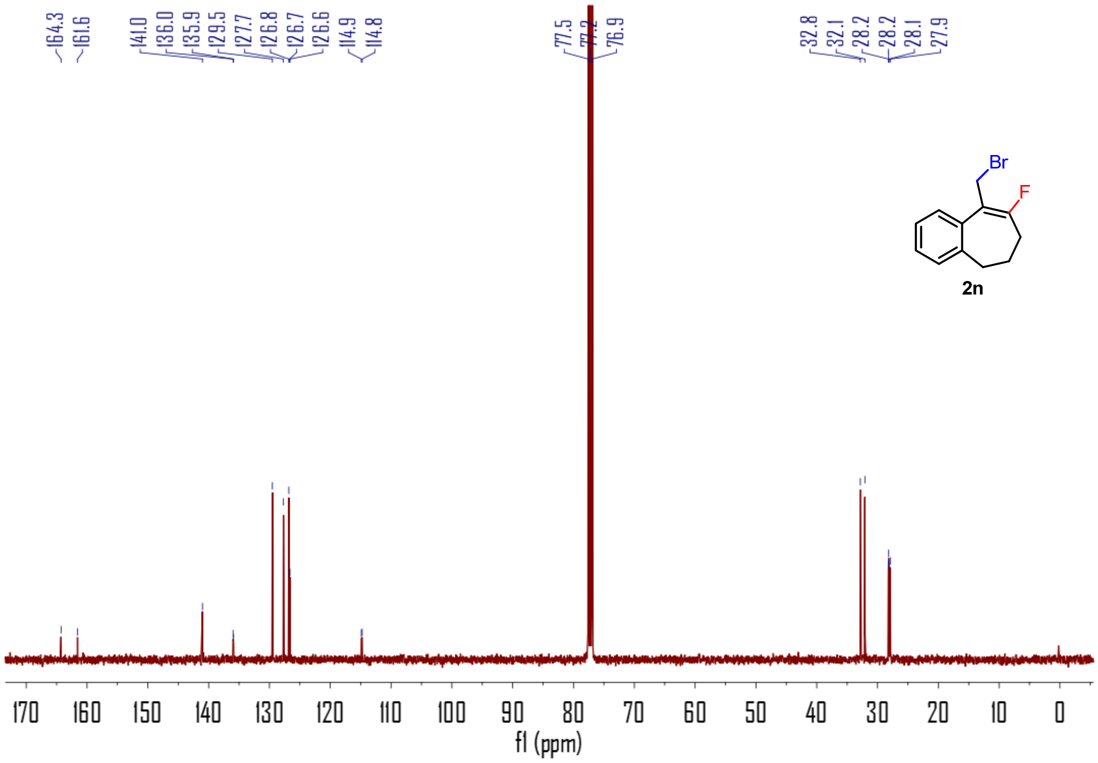

^{19}F NMR Spectrum (in $CDCl_3$, 376 MHz)

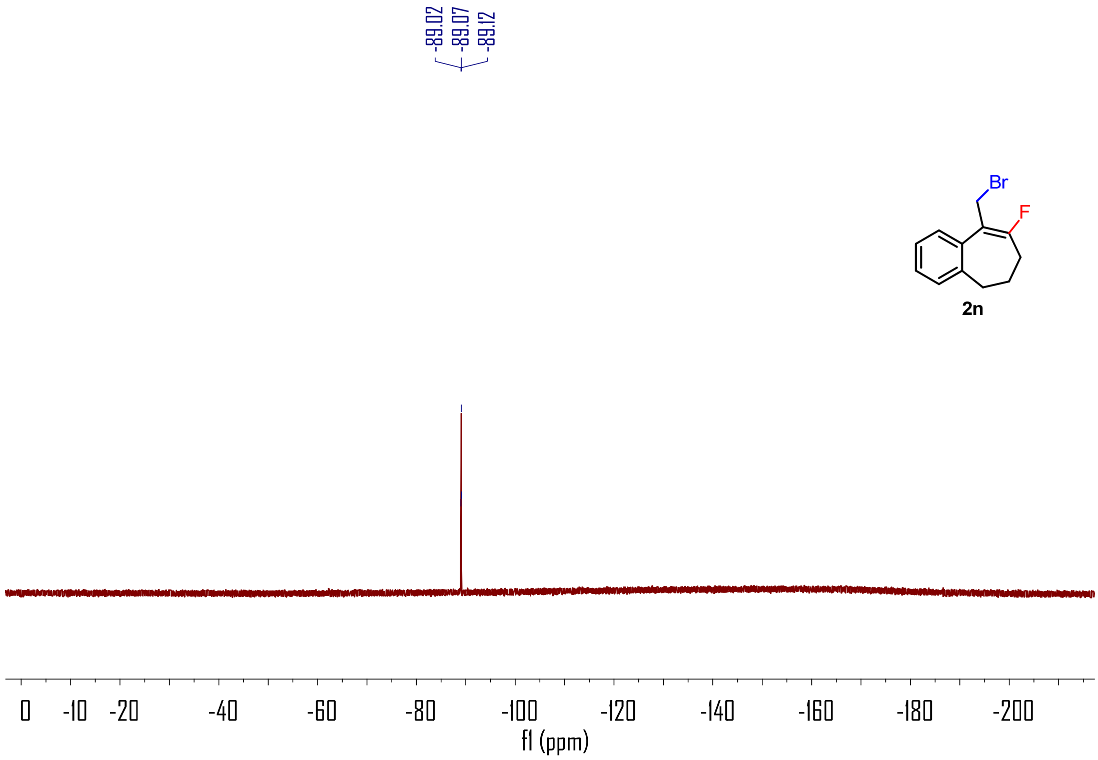

^{1}H NMR Spectrum (in $CDCl_3$, 400 MHz)

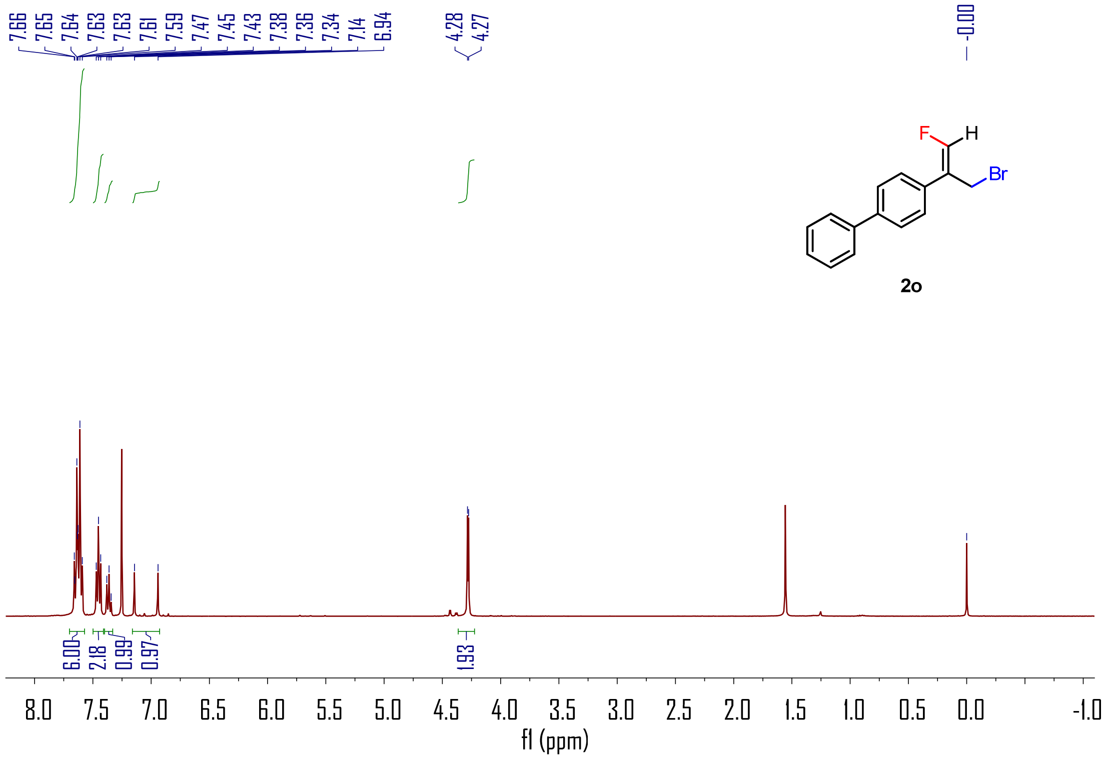

^{13}C NMR Spectrum (in $CDCl_3$, 101 MHz)

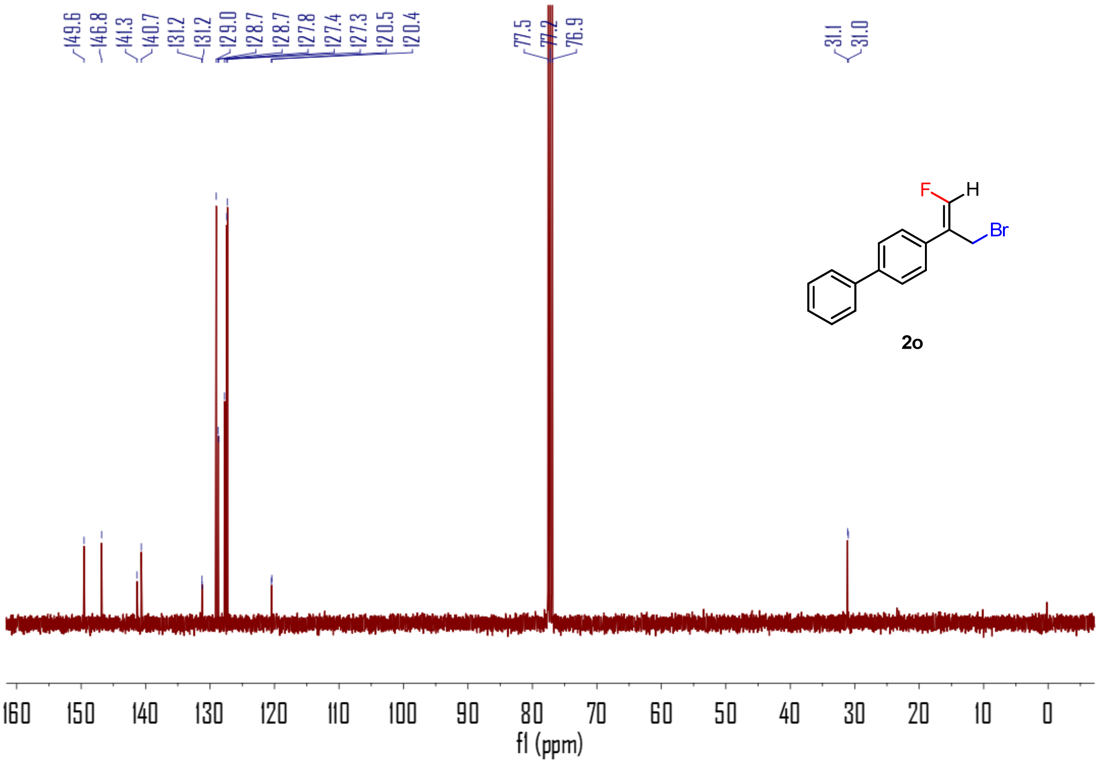

^{19}F NMR Spectrum (in $CDCl_3$, 376 MHz)

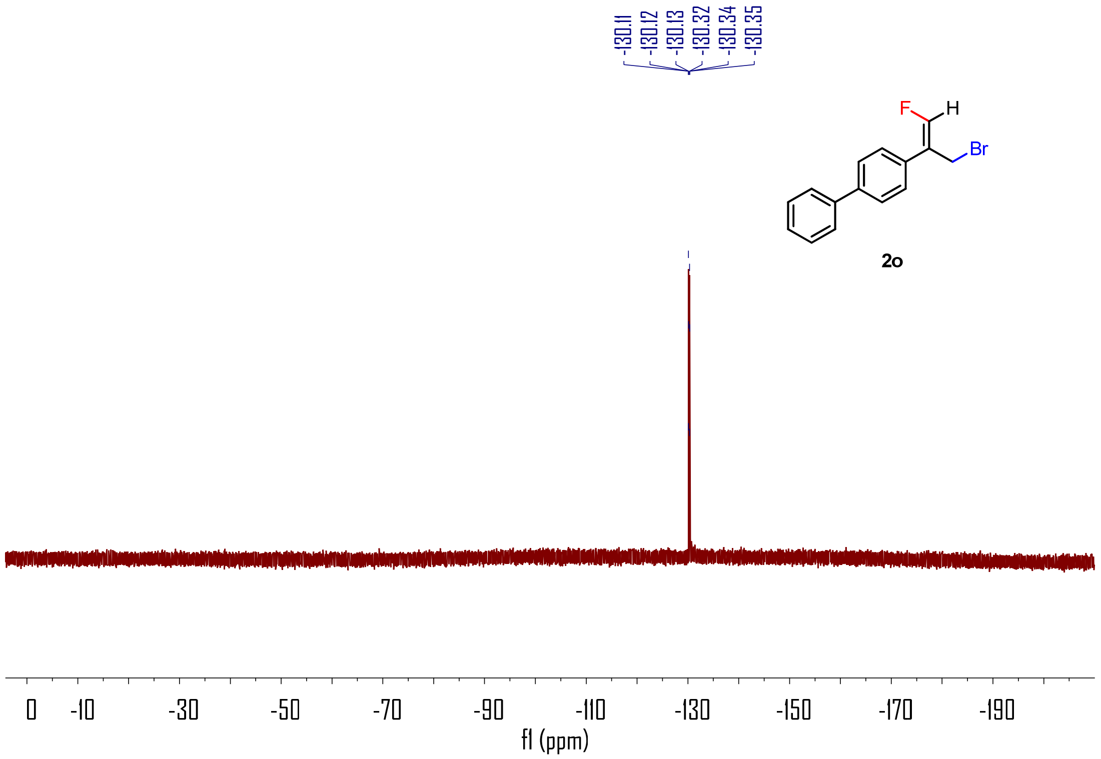

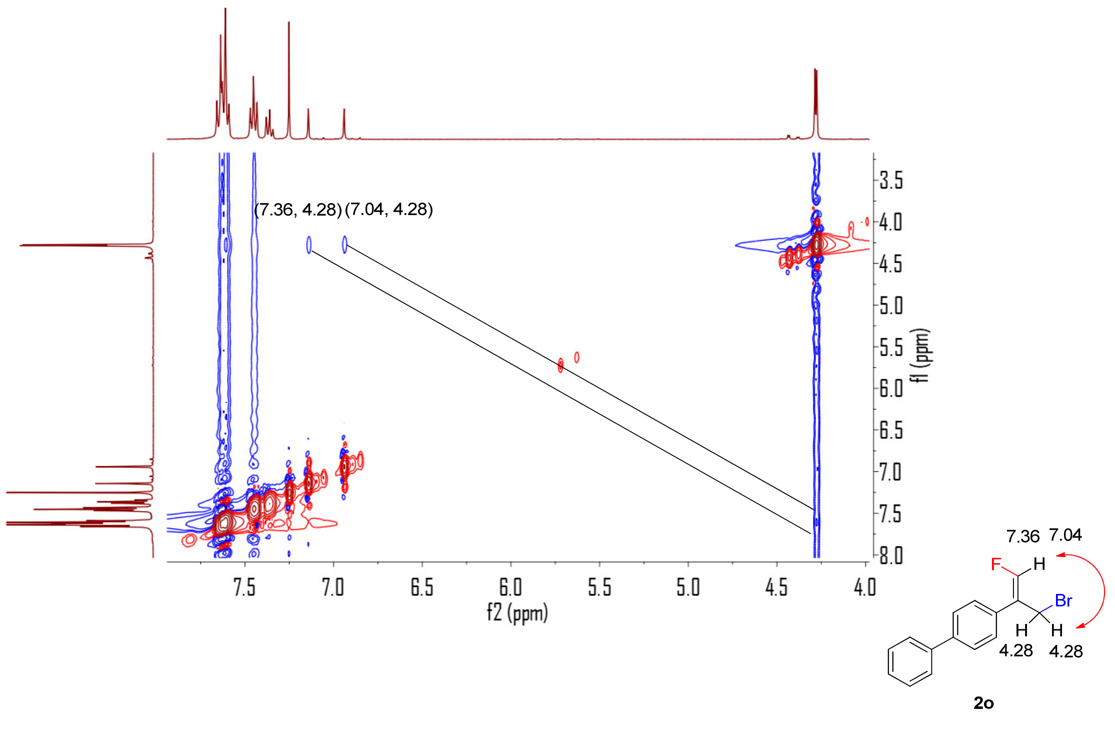

^{1}H NMR Spectrum (in $CDCl_3$, 400 MHz)

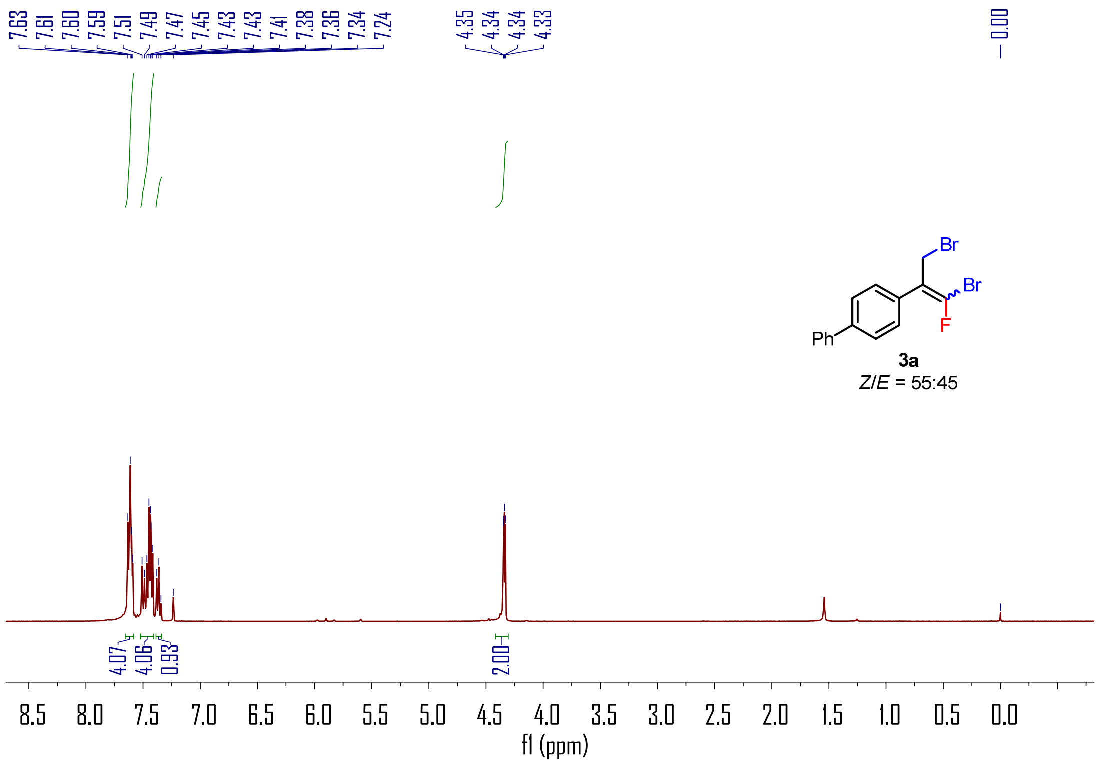

^{13}C NMR Spectrum (in $CDCl_3$, 101 MHz)

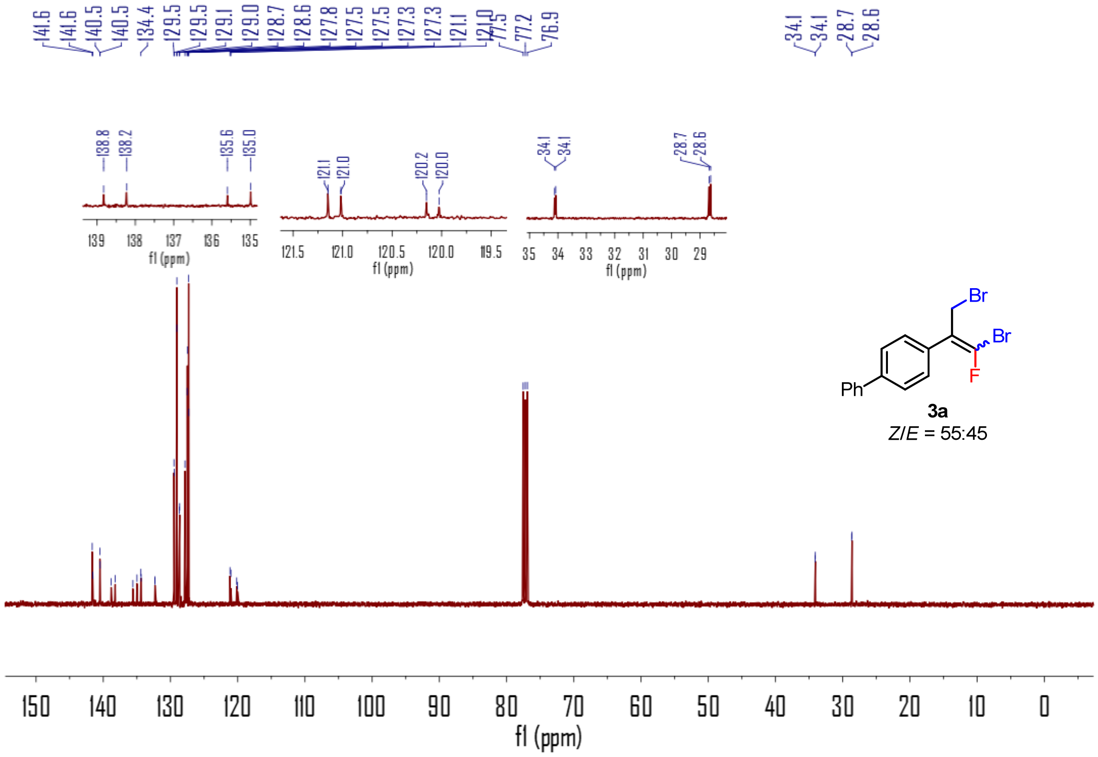

^{19}F NMR Spectrum (in $CDCl_3$, 376 MHz)

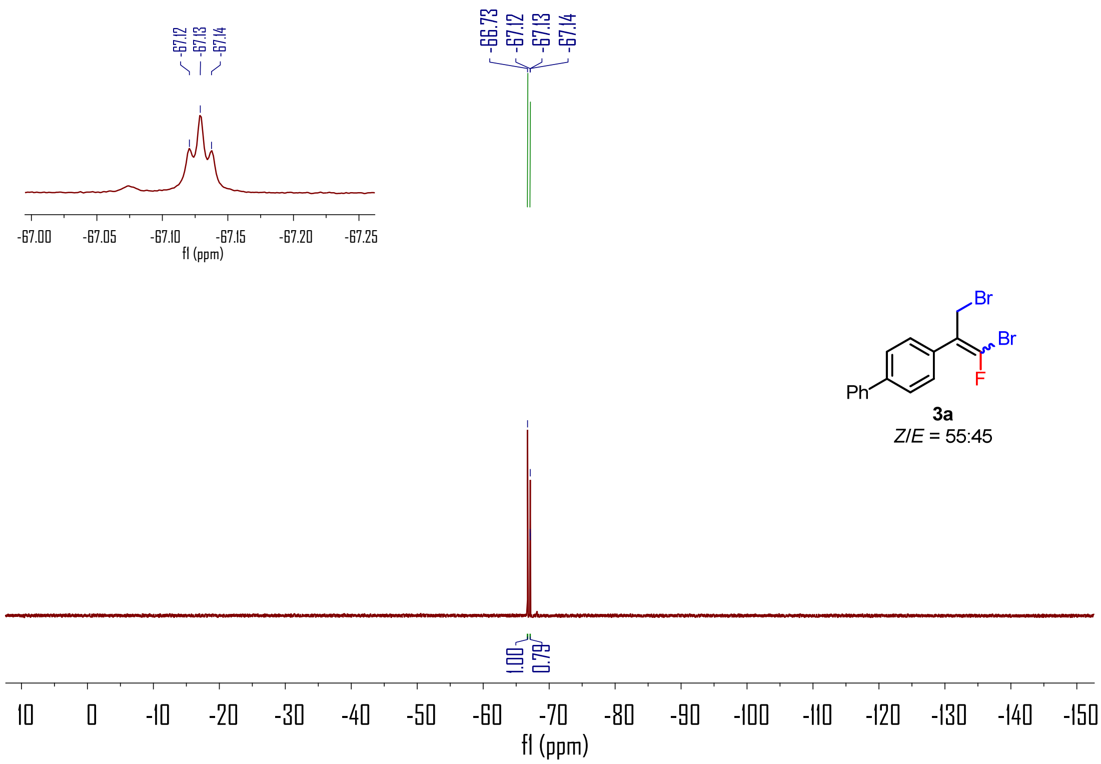

^{1}H NMR Spectrum (in $CDCl_3$, 400 MHz)

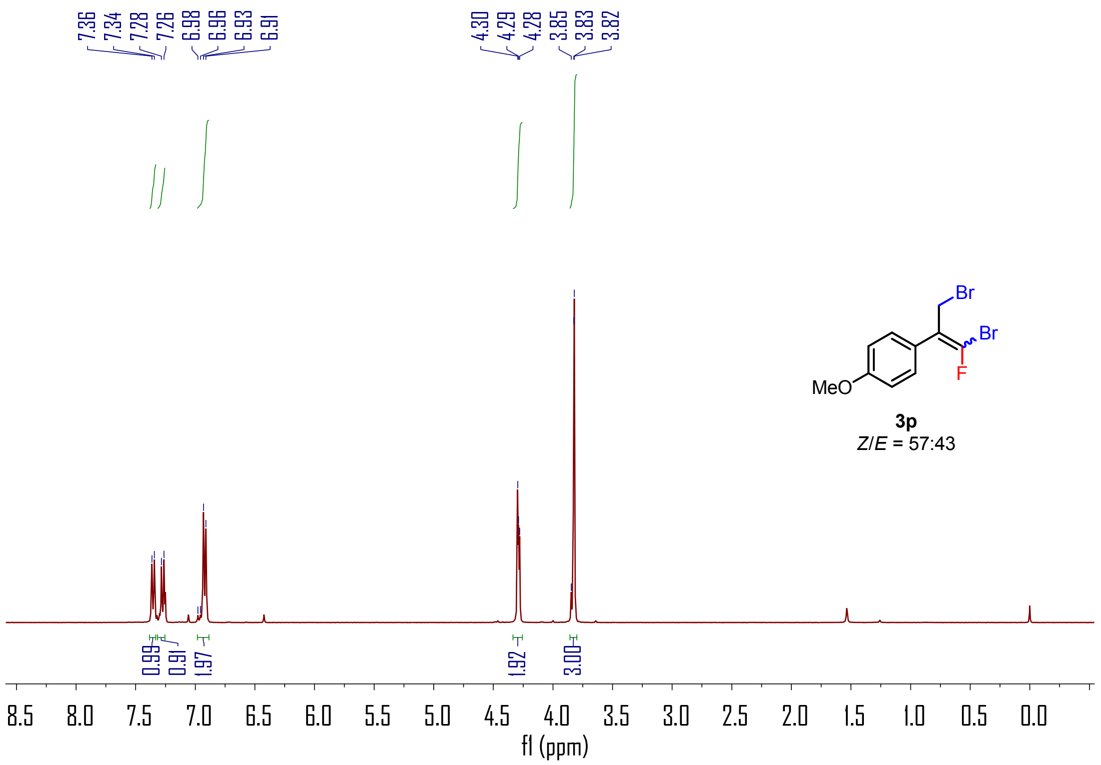

^{13}C NMR Spectrum (in $CDCl_3$, 101 MHz)

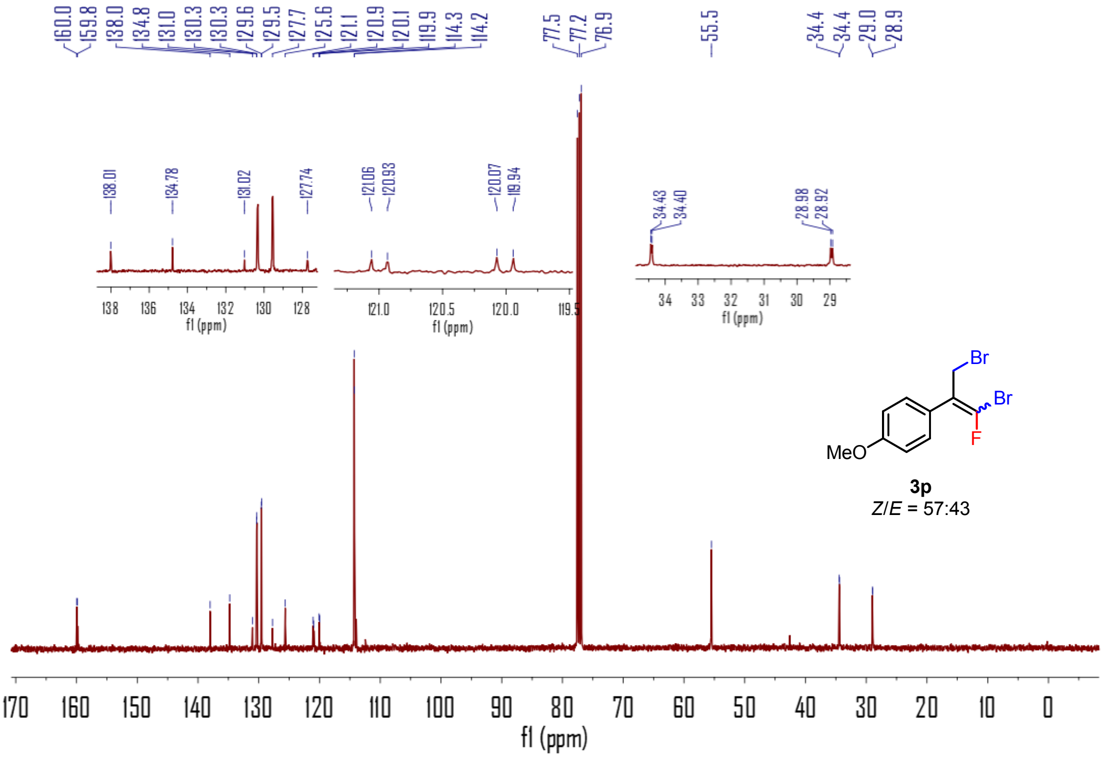

^{19}F NMR Spectrum (in $CDCl_3$, 376 MHz)

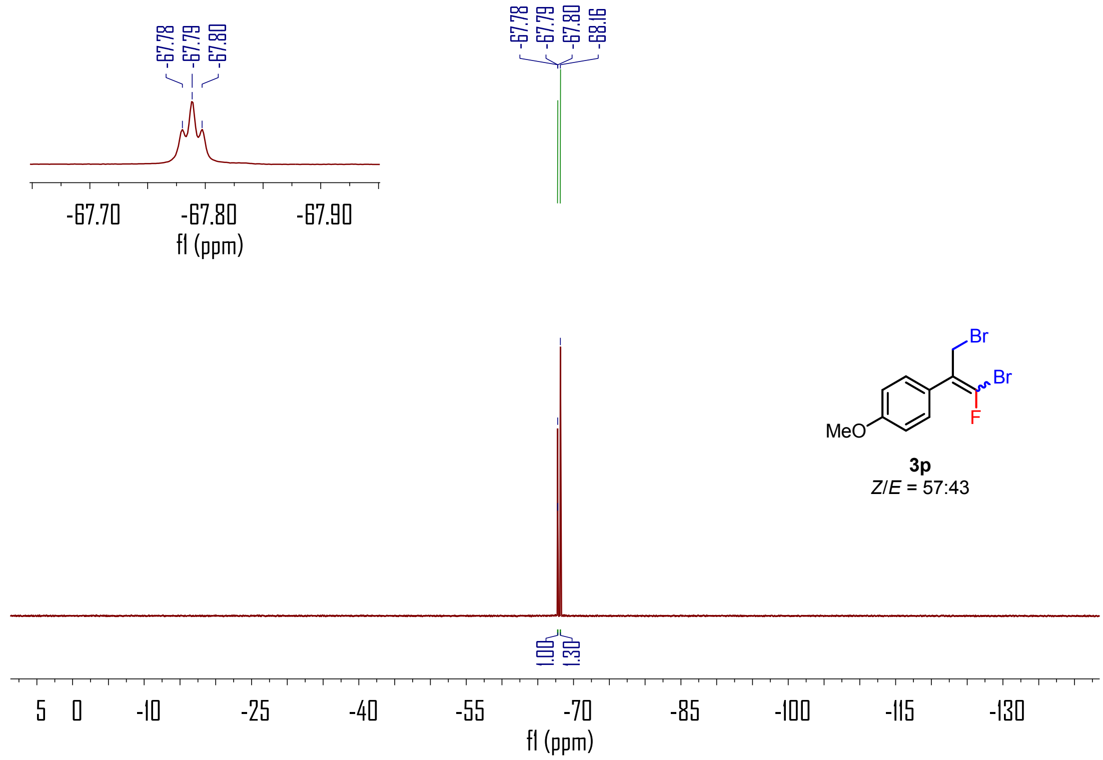

^{1}H NMR Spectrum (in $CDCl_3$, 400 MHz)

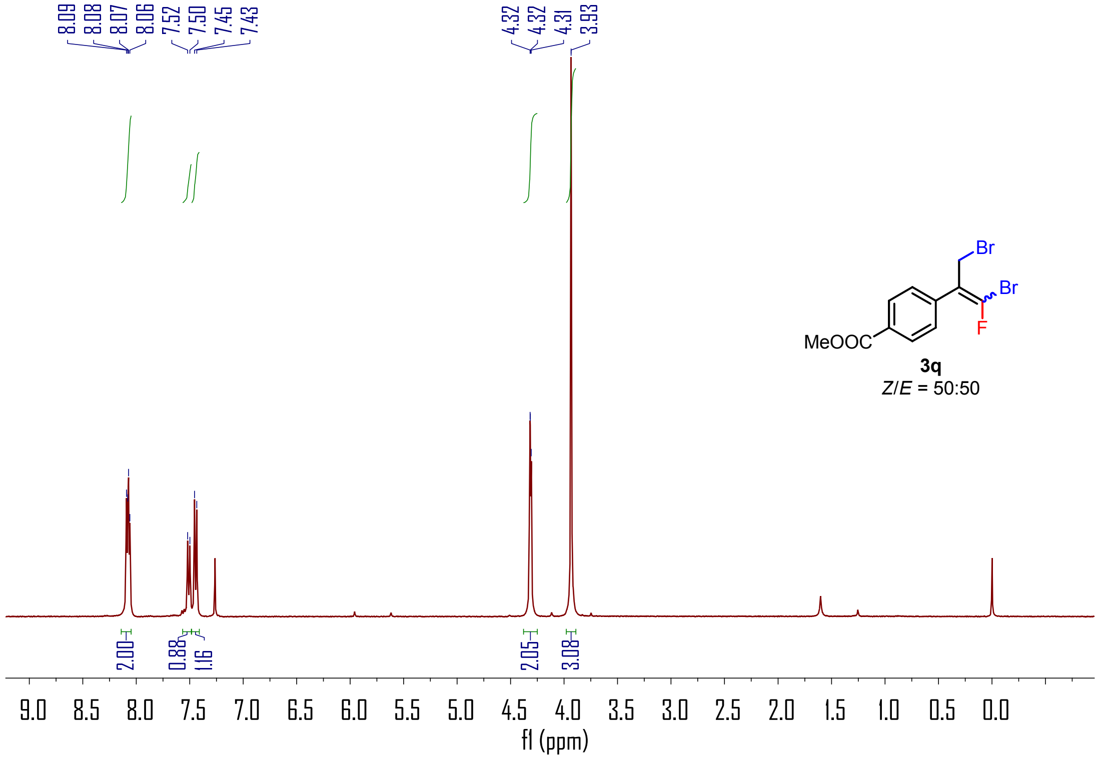

^{13}C NMR Spectrum (in $CDCl_3$, 101 MHz)

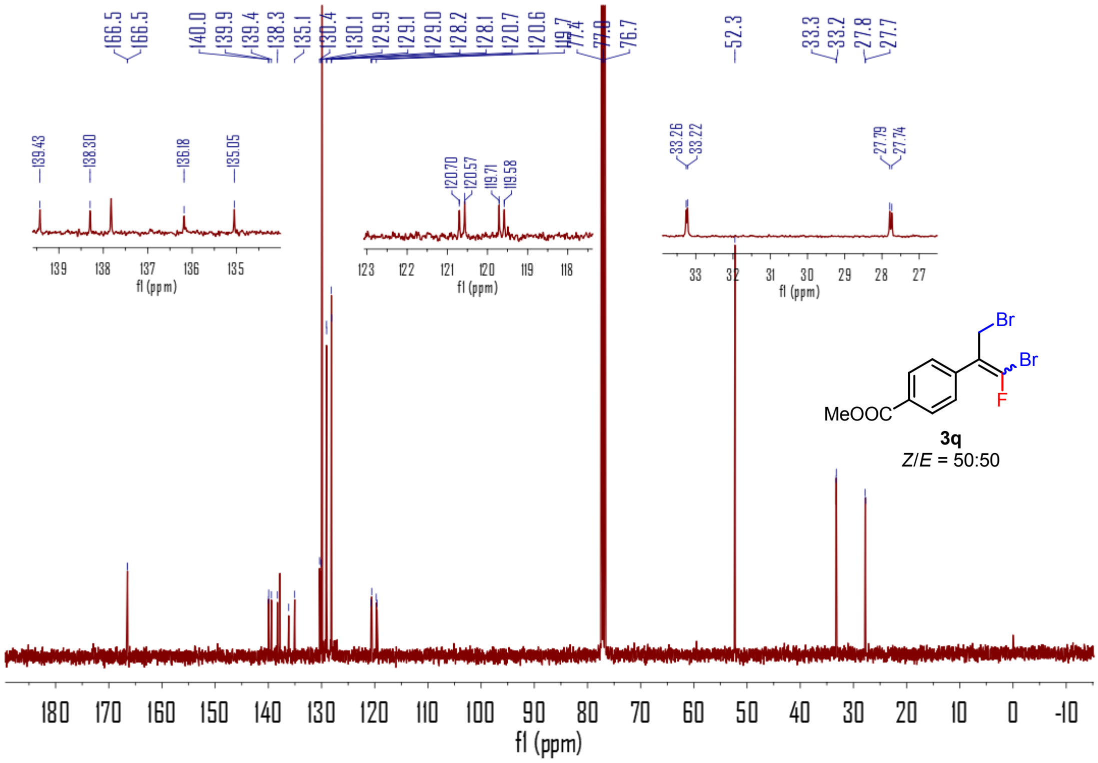

^{19}F NMR Spectrum (in $CDCl_3$, 376 MHz)

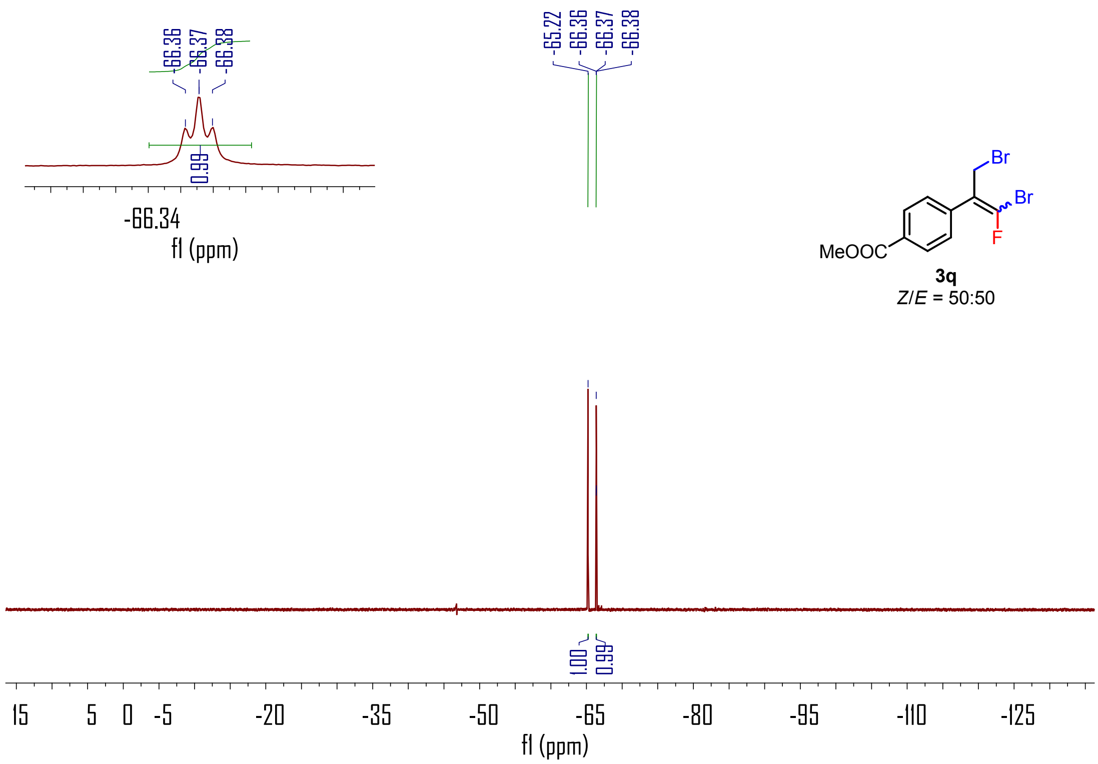

^{1}H NMR Spectrum (in $CDCl_3$, 400 MHz)

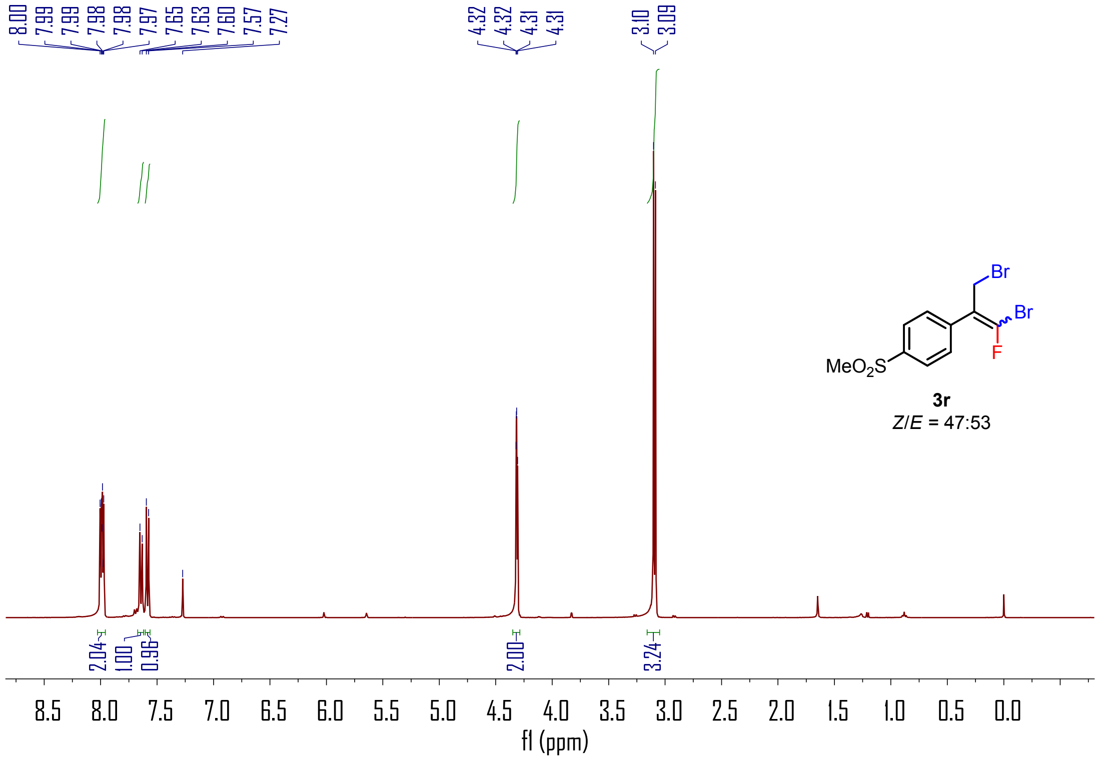

^{13}C NMR Spectrum (in $CDCl_3$, 101 MHz)

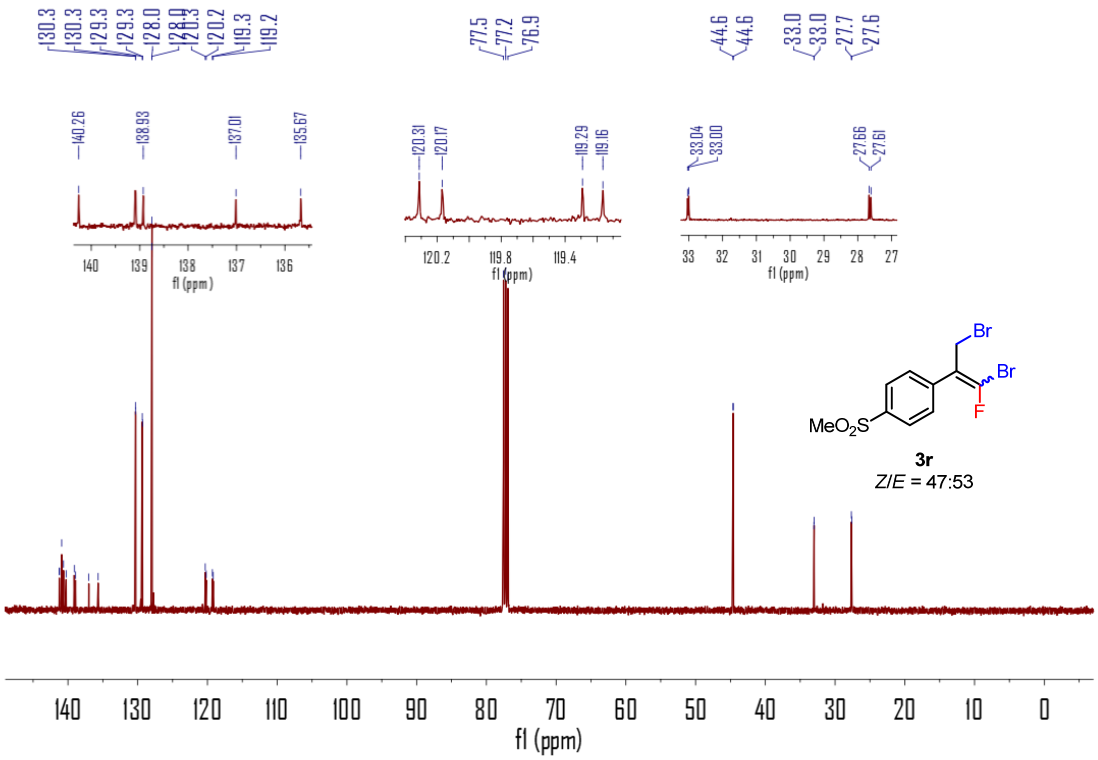

^{19}F NMR Spectrum (in $CDCl_3$, 376 MHz)

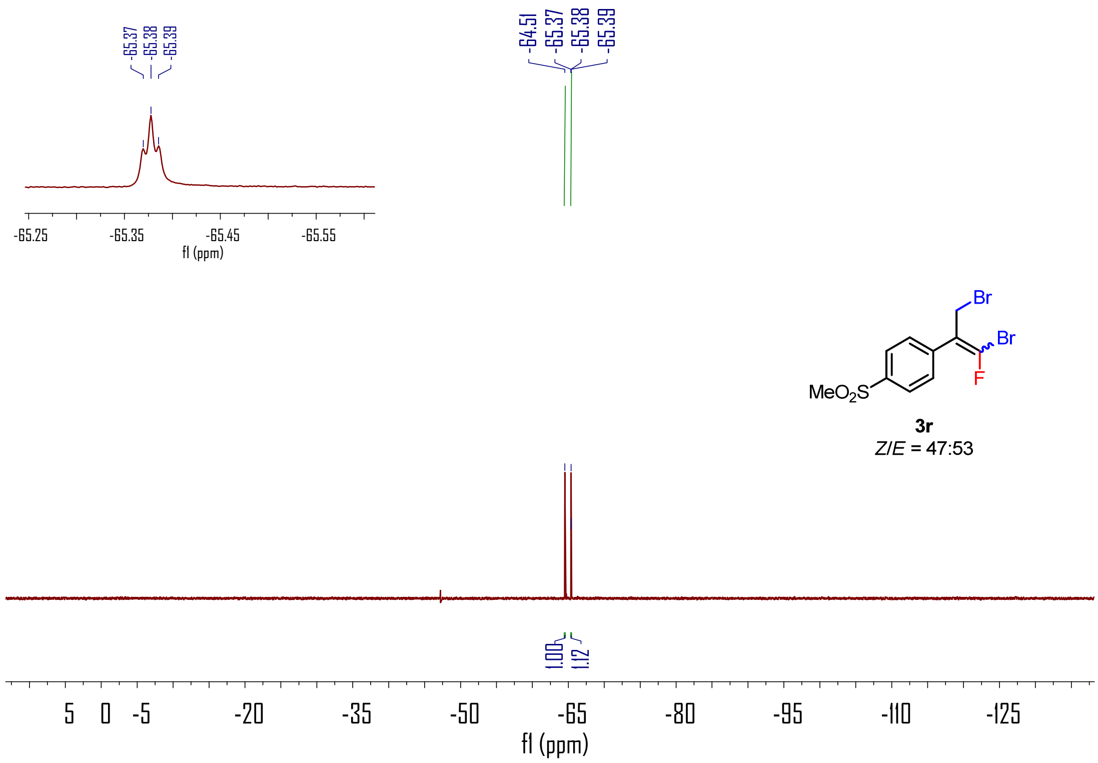

^{1}H NMR Spectrum (in $CDCl_3$, 400 MHz)

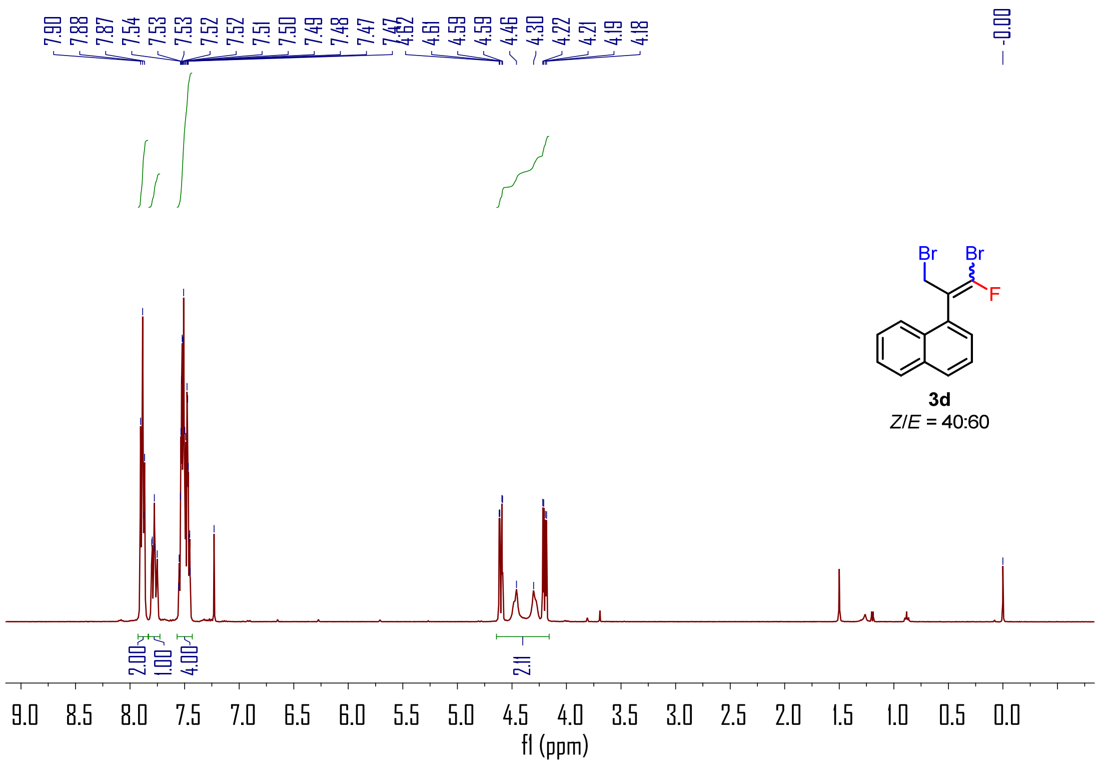

^{13}C NMR Spectrum (in $CDCl_3$, 101 MHz)

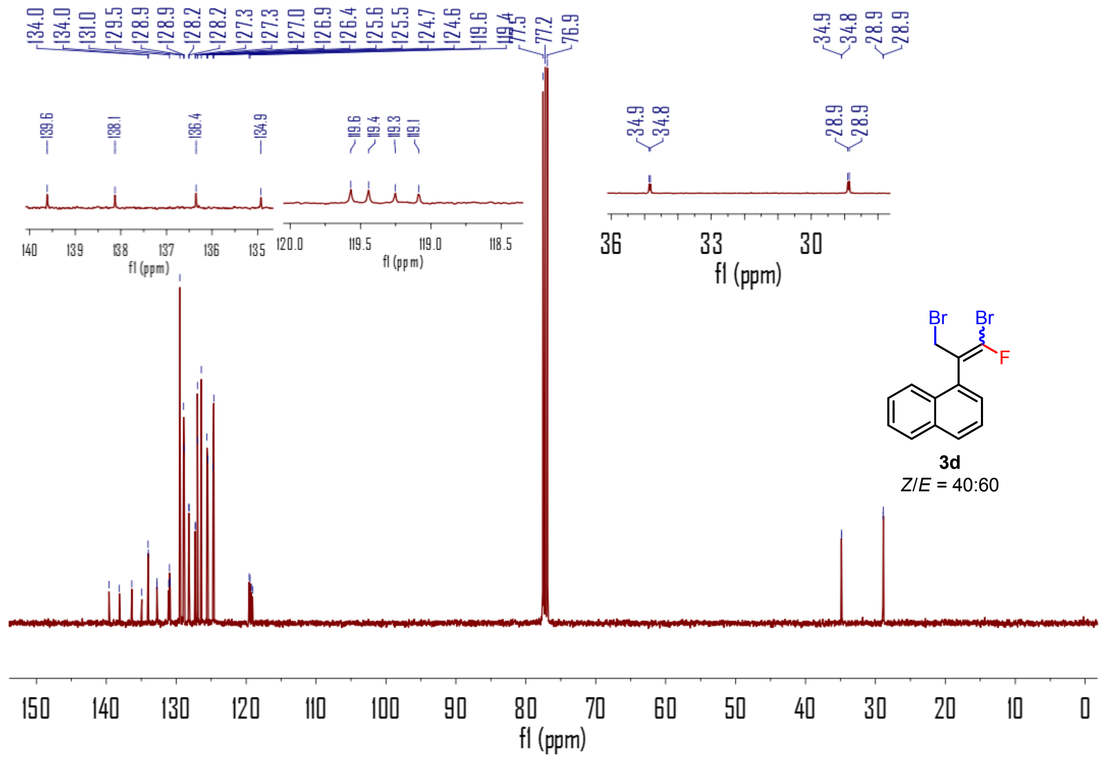

^{19}F NMR Spectrum (in $CDCl_3$, 376 MHz)

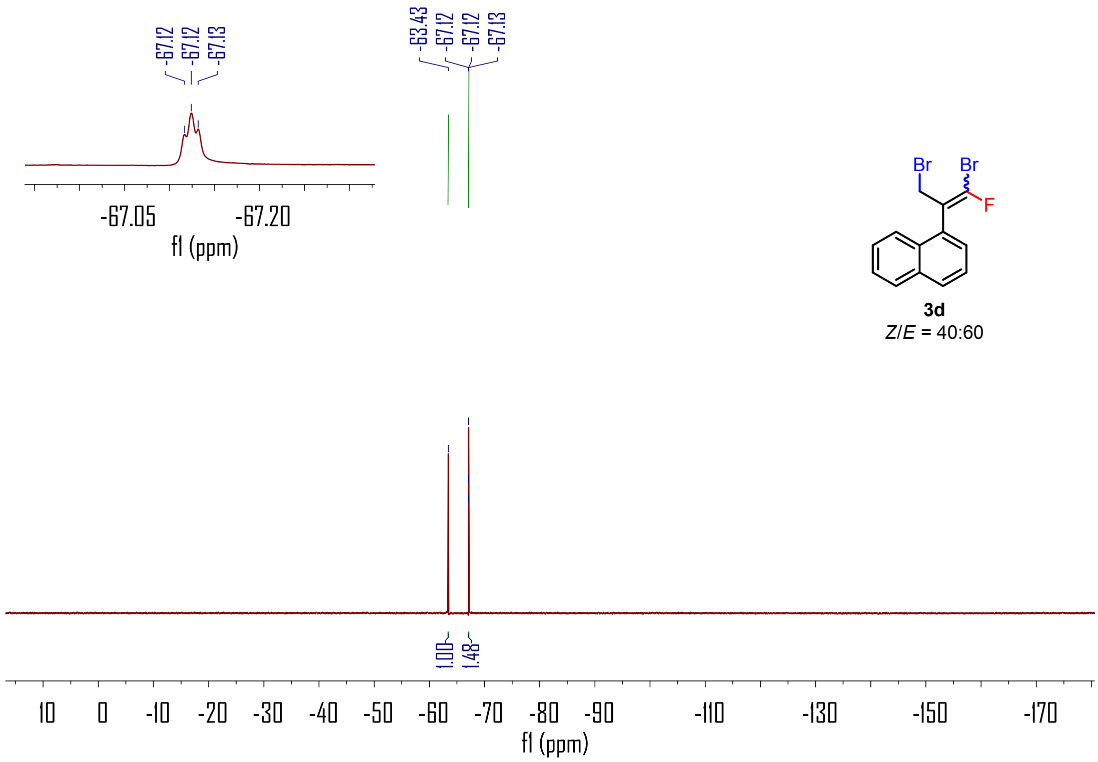

^{1}H NMR Spectrum (in $CDCl_3$, 400 MHz)

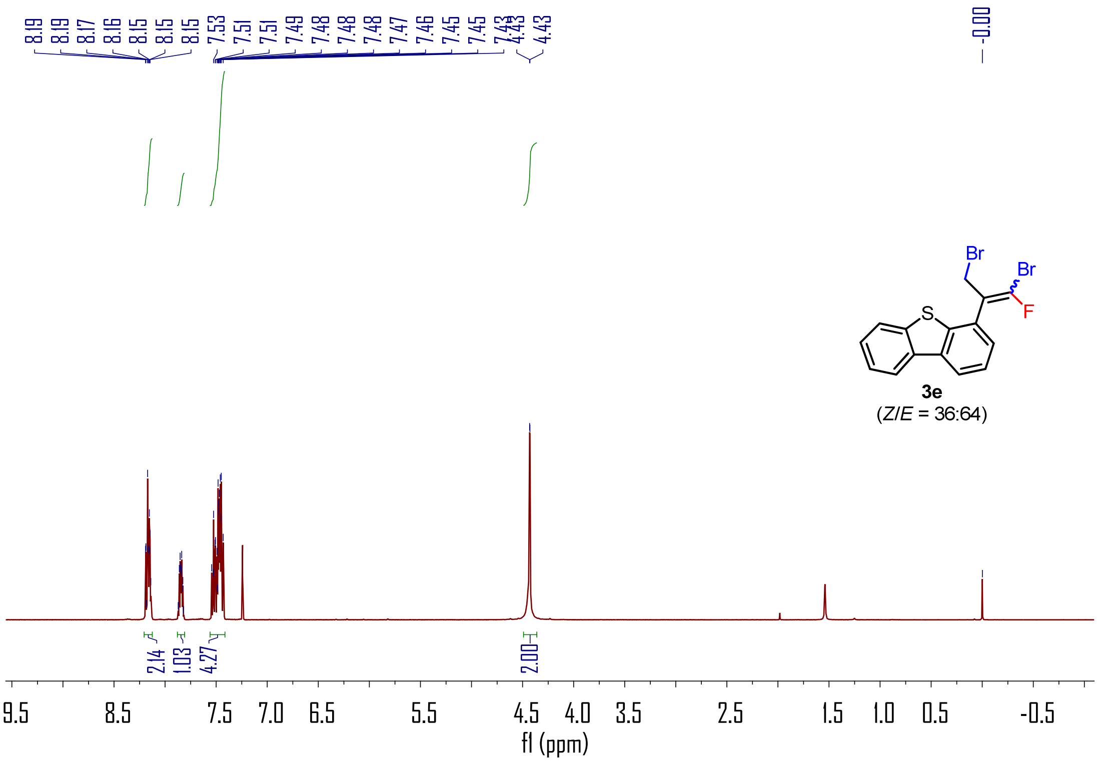

^{13}C NMR Spectrum (in $CDCl_3$, 101 MHz)

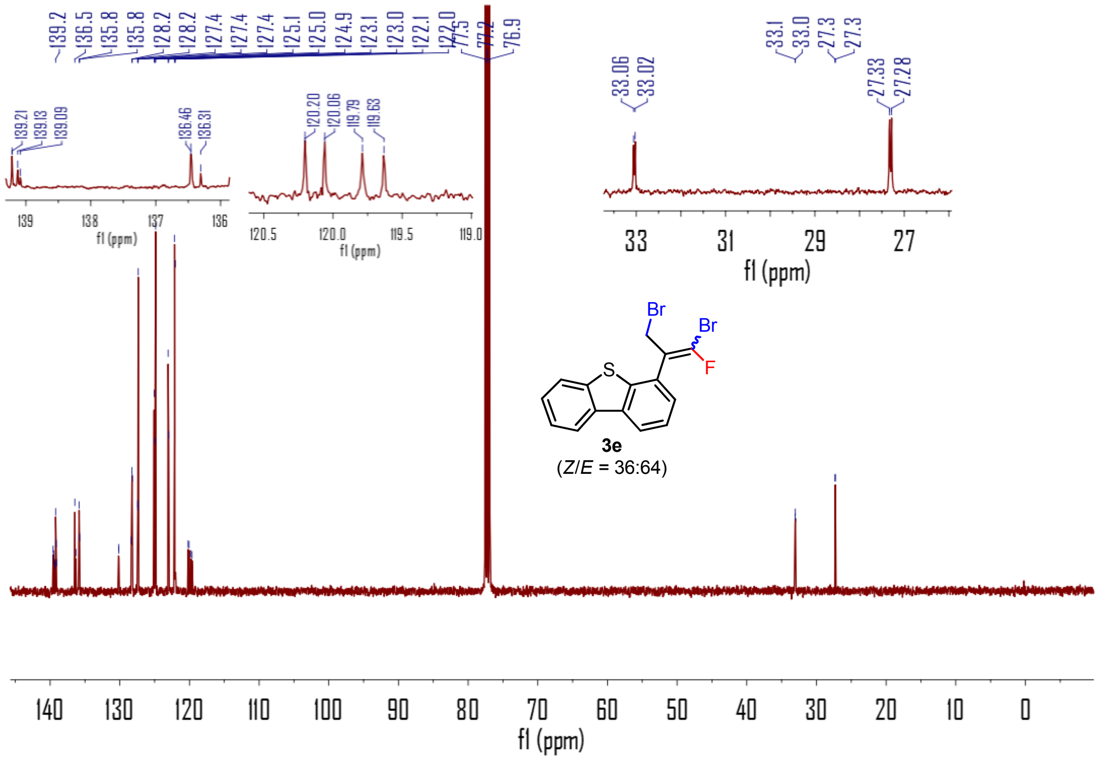

^{19}F NMR Spectrum (in $CDCl_3$, 376 MHz)

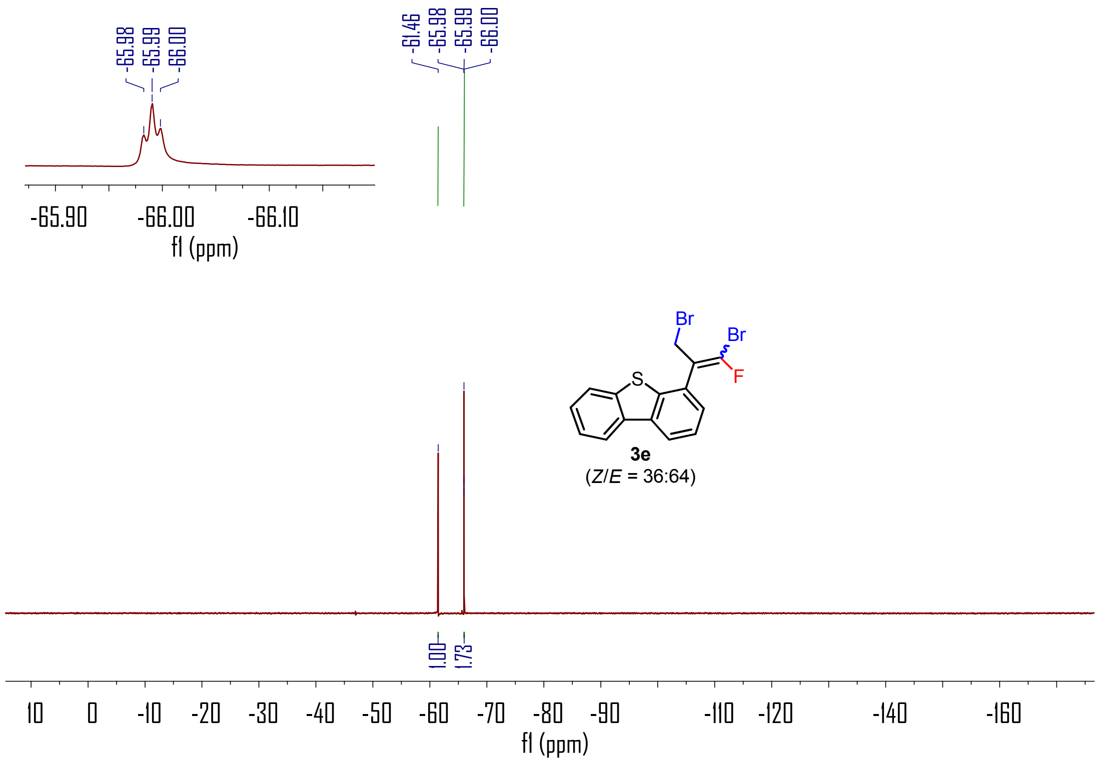

^{1}H NMR Spectrum (in CDCl3, 400 MHz)

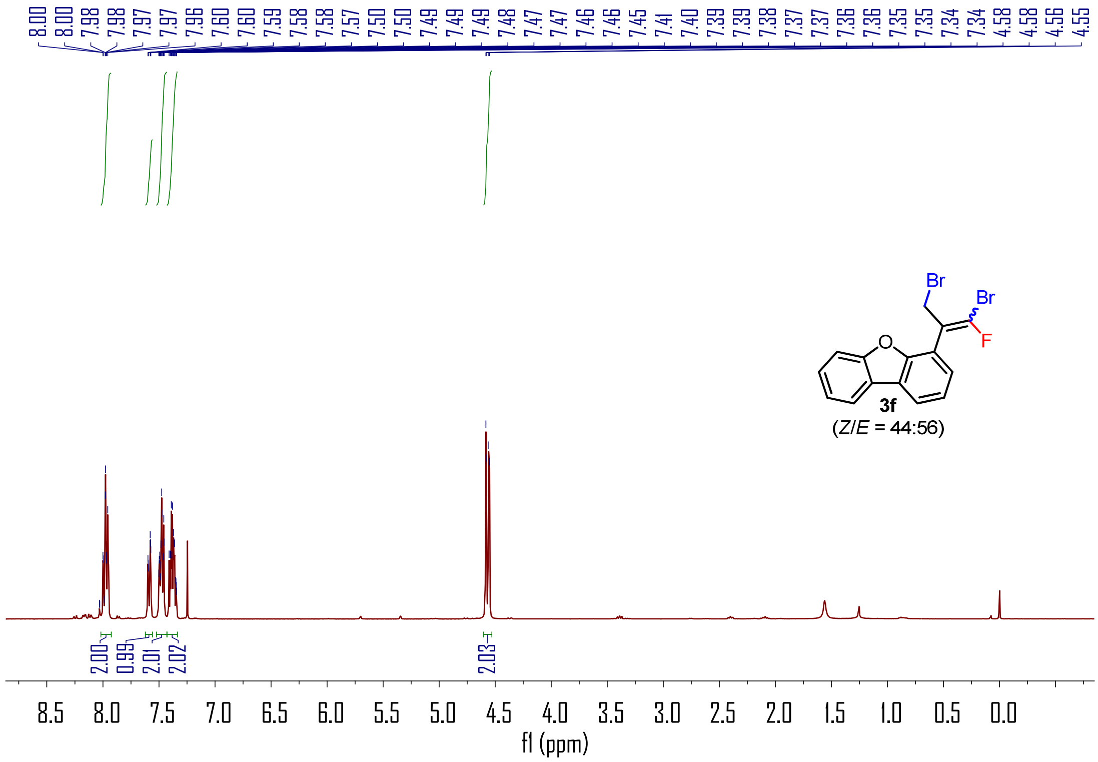

^{13}C NMR Spectrum (in CDCl3, 101 MHz)

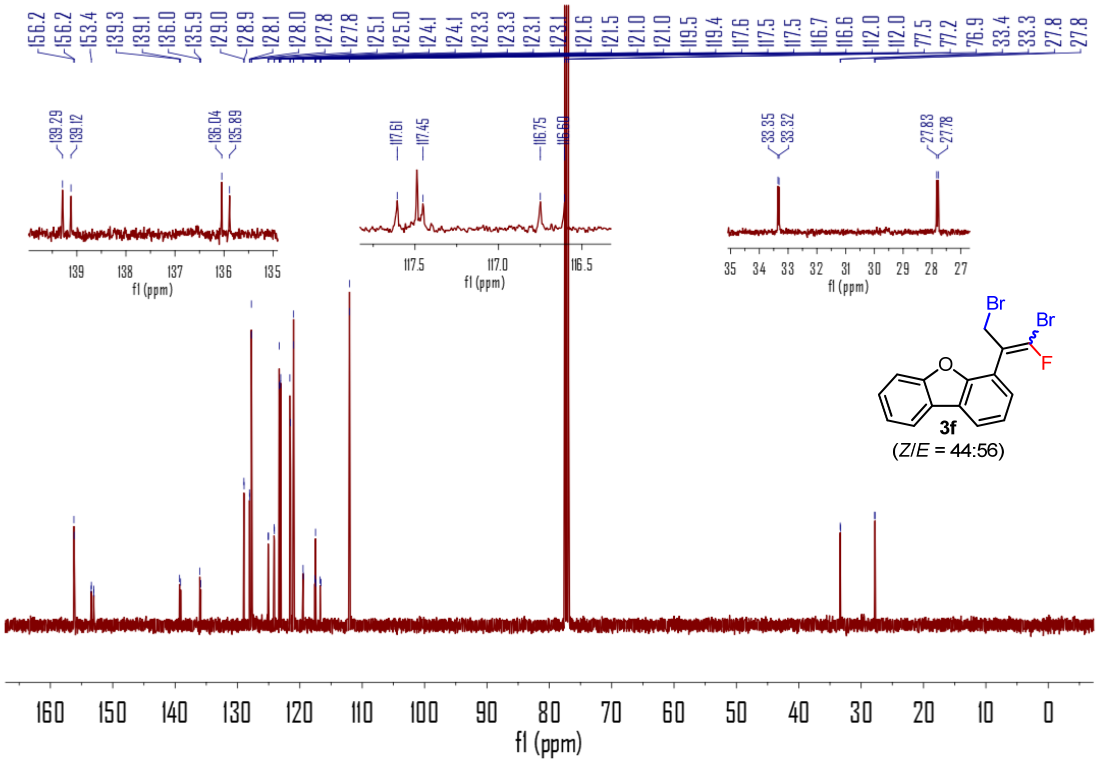

^{19}F NMR Spectrum (in $CDCl_3$, 376 MHz)

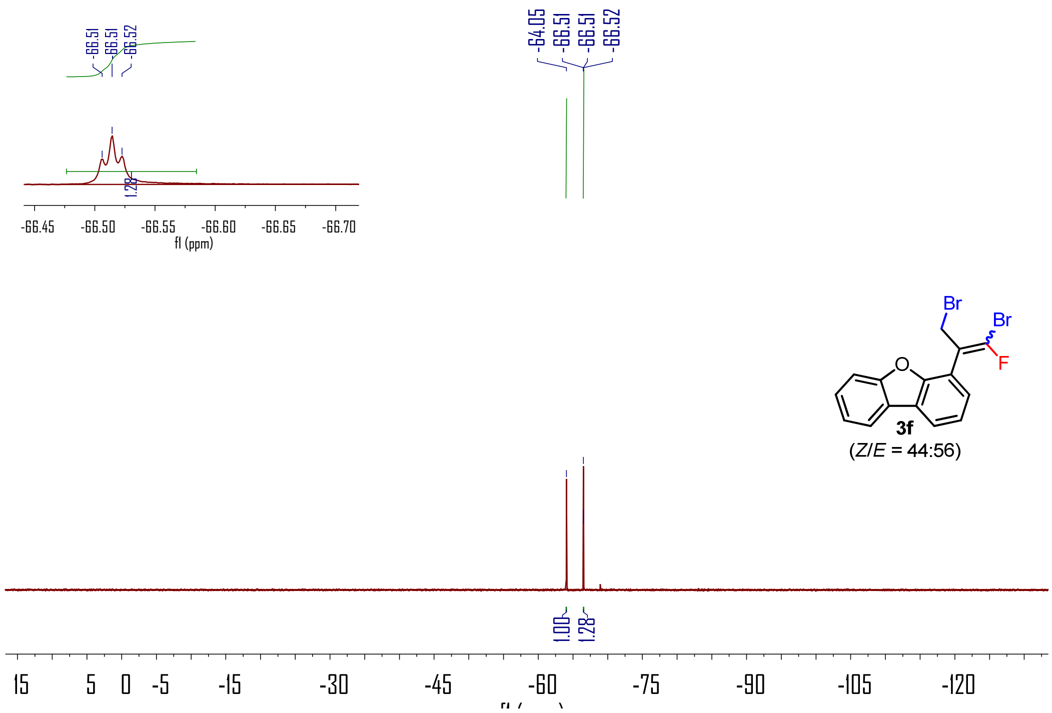

^{1}H NMR Spectrum (in $CDCl_3$, 400 MHz)

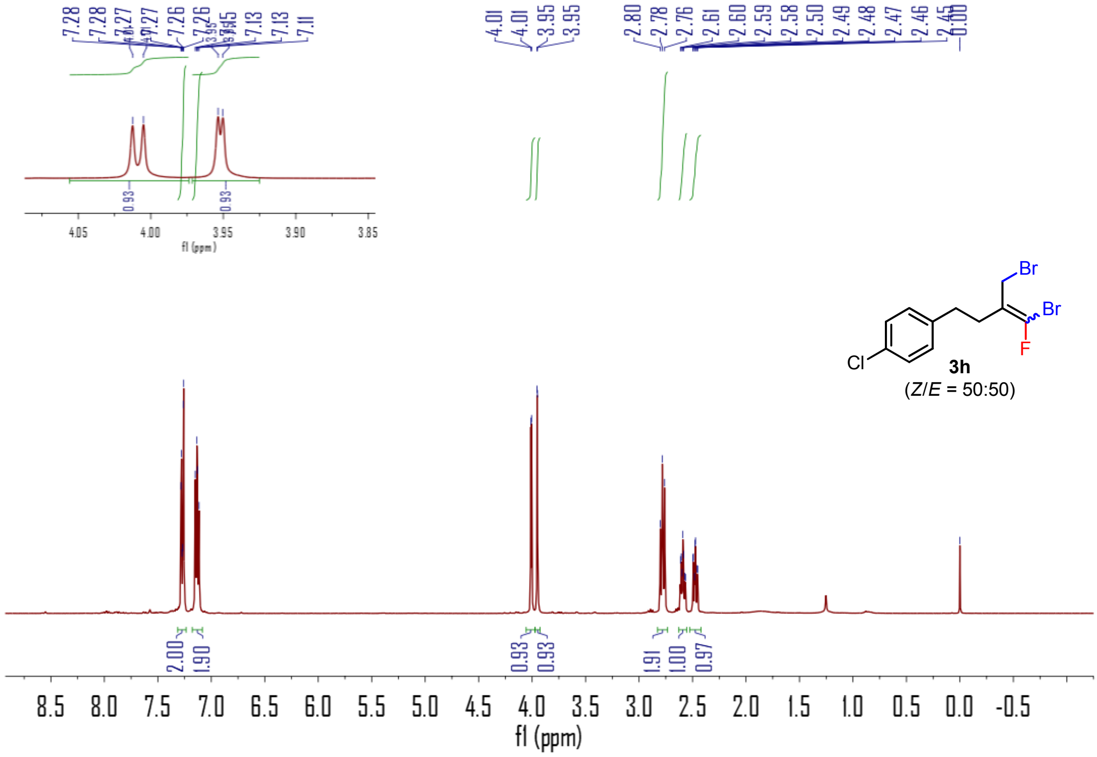

^{13}C NMR Spectrum (in CDCl3, 101 MHz)

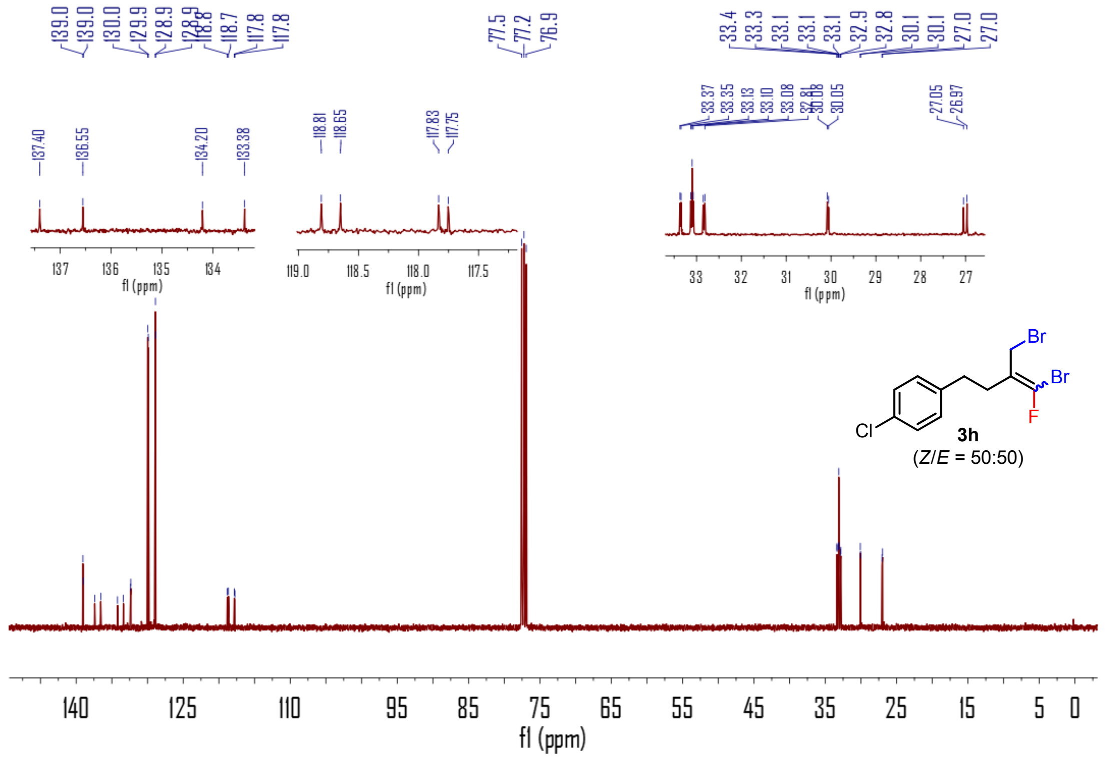

^{19}F NMR Spectrum (in CDCl3, 376 MHz)

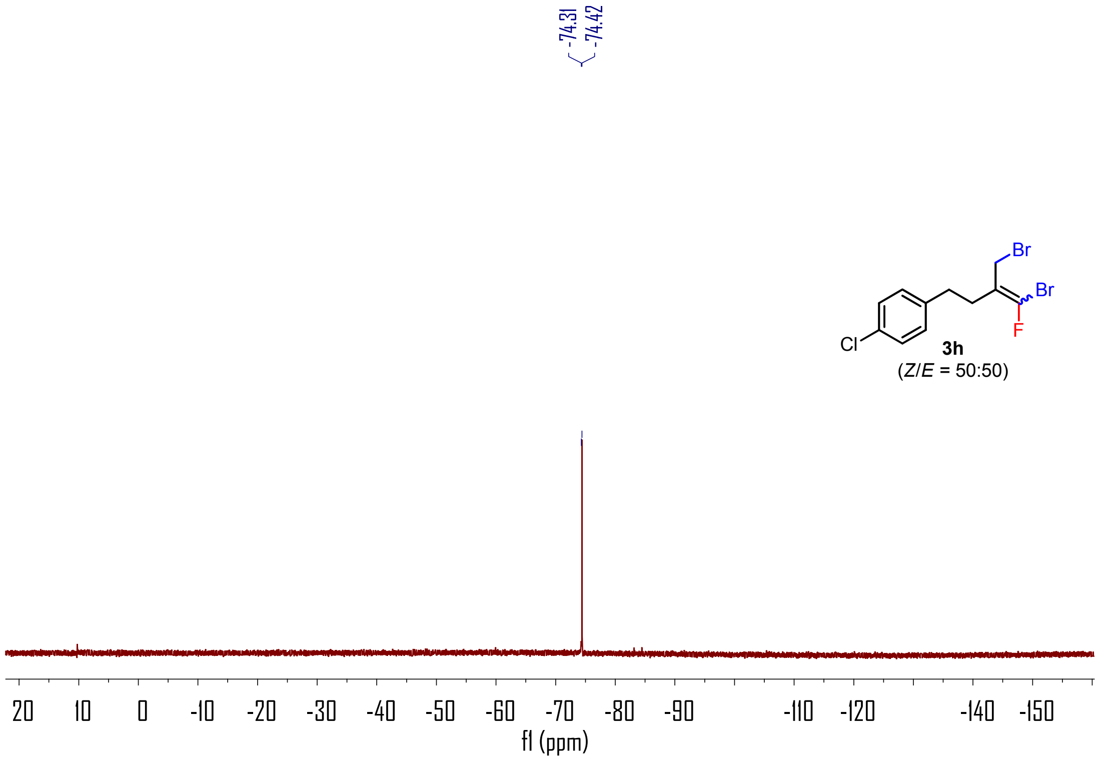

^{1}H NMR Spectrum (in $CDCl_3$, 400 MHz)

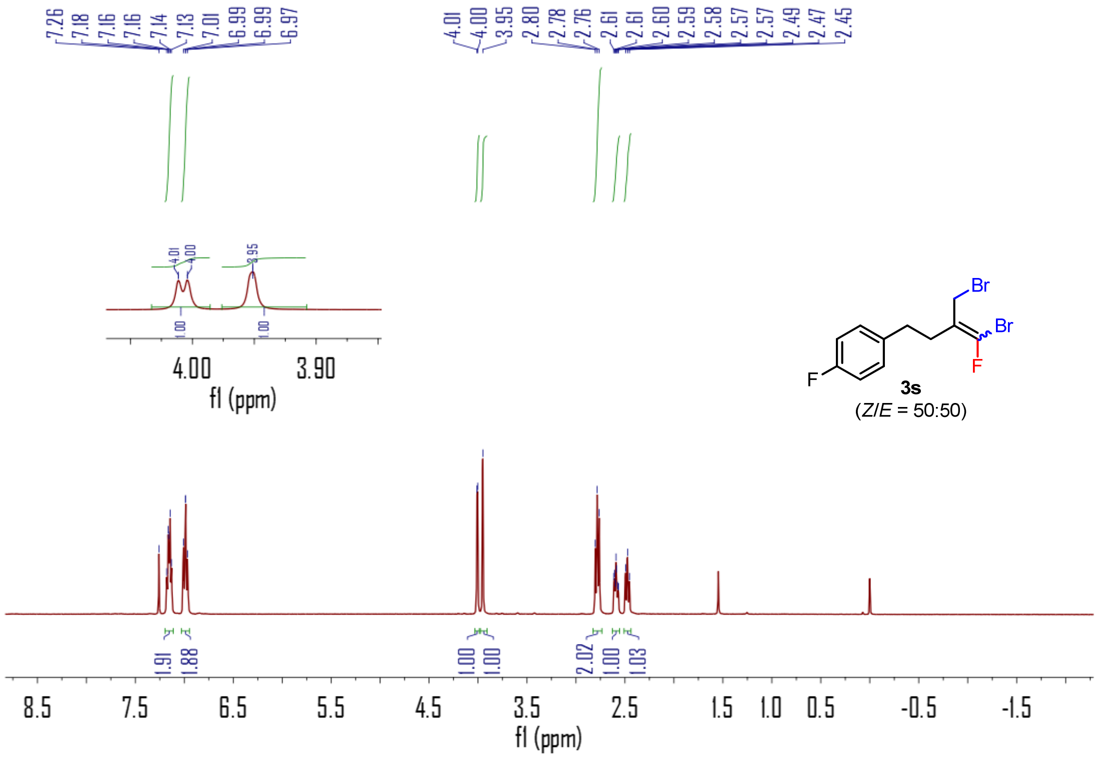

^{13}C NMR Spectrum (in $CDCl_3$, 101 MHz)

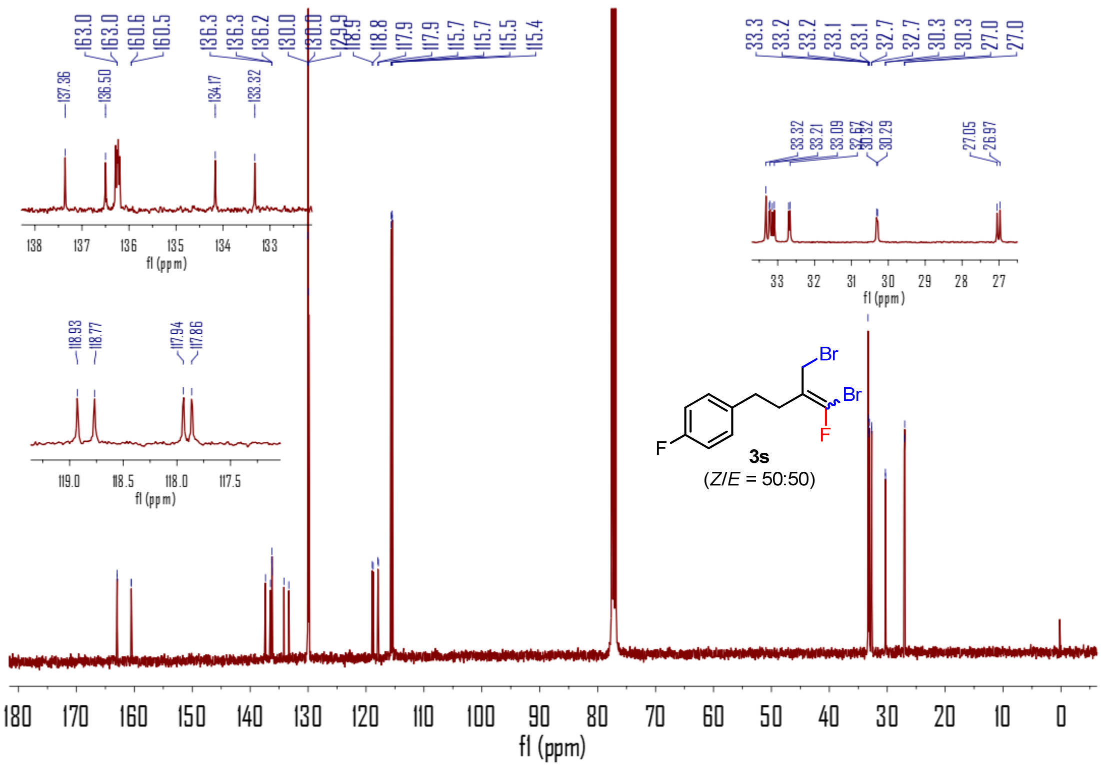

^{19}F NMR Spectrum (in $CDCl_3$, 376 MHz)

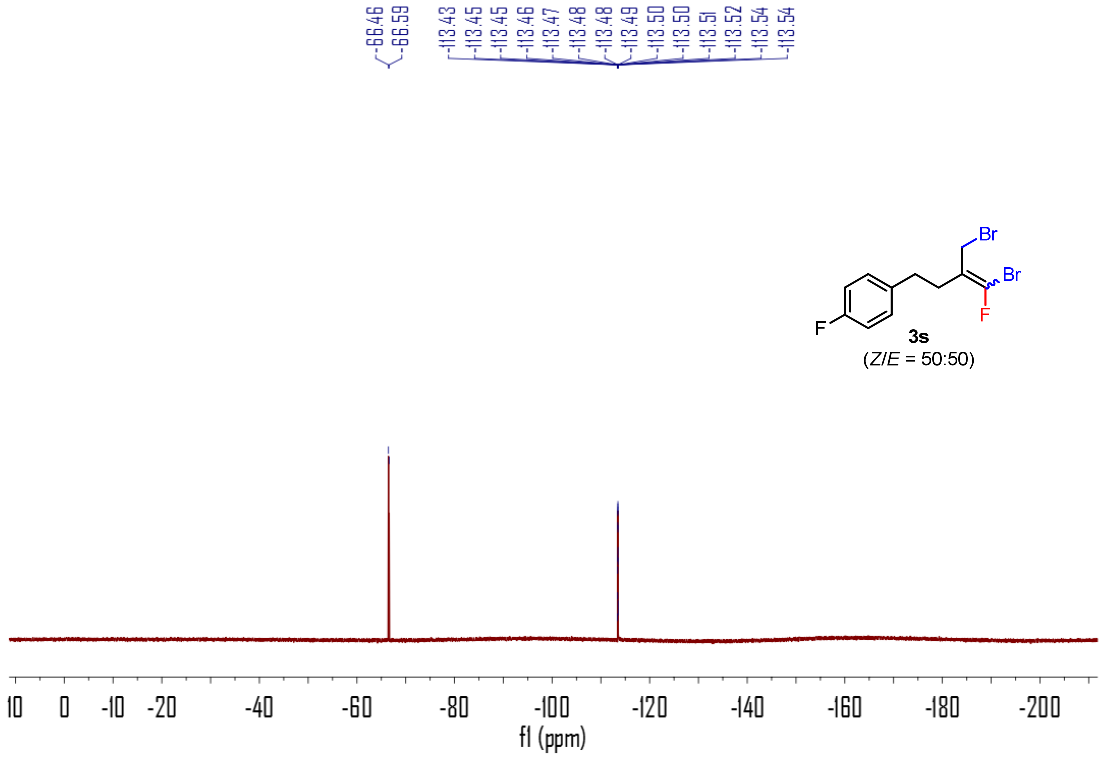

^{1}H NMR Spectrum (in $CDCl_3$, 400 MHz)

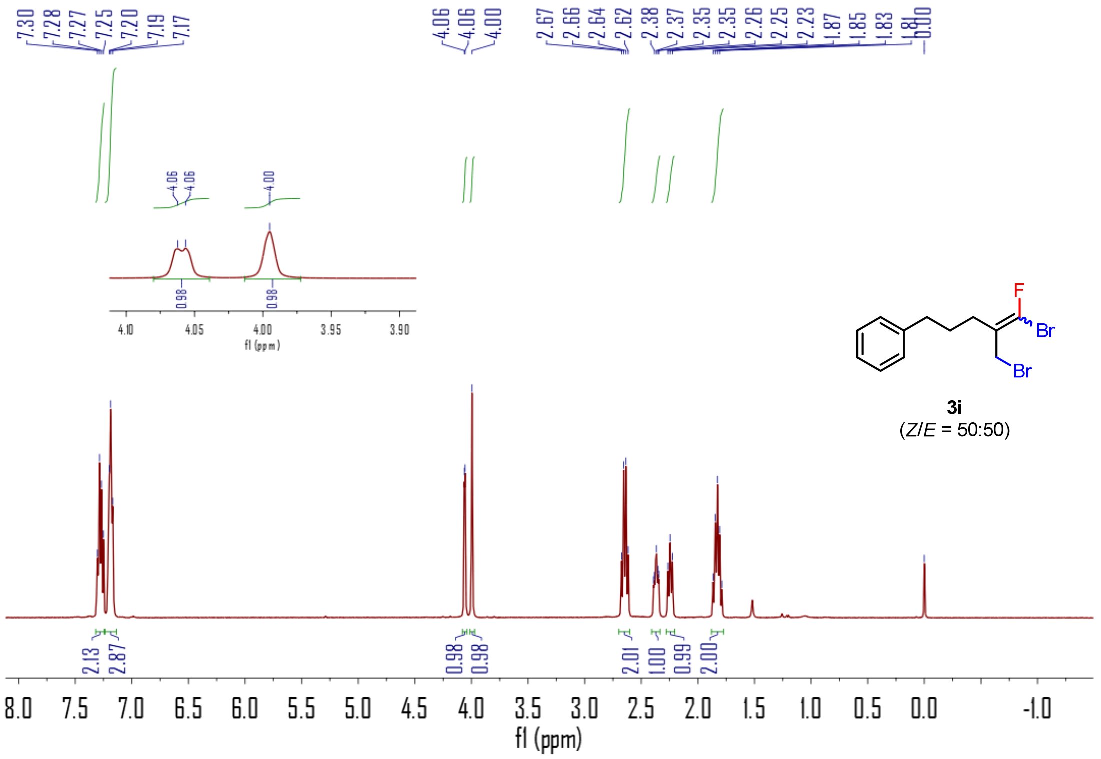

^{13}C NMR Spectrum (in $CDCl_3$, 101 MHz)

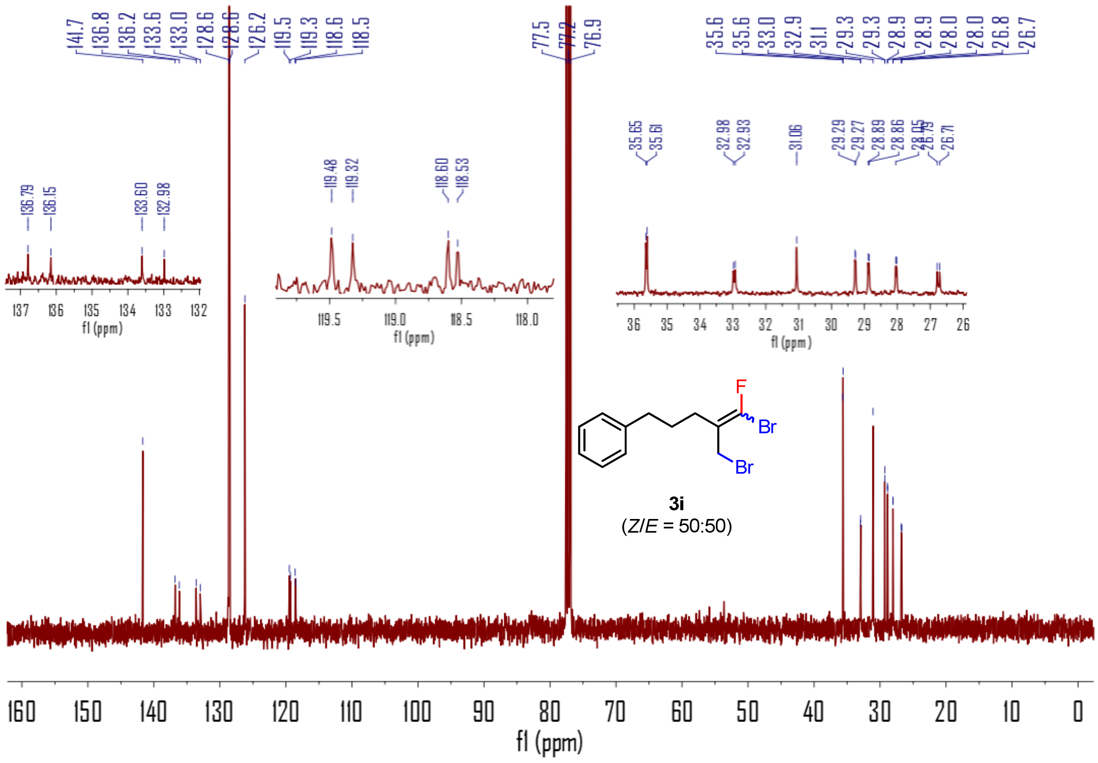

^{19}F NMR Spectrum (in $CDCl_3$, 376 MHz)

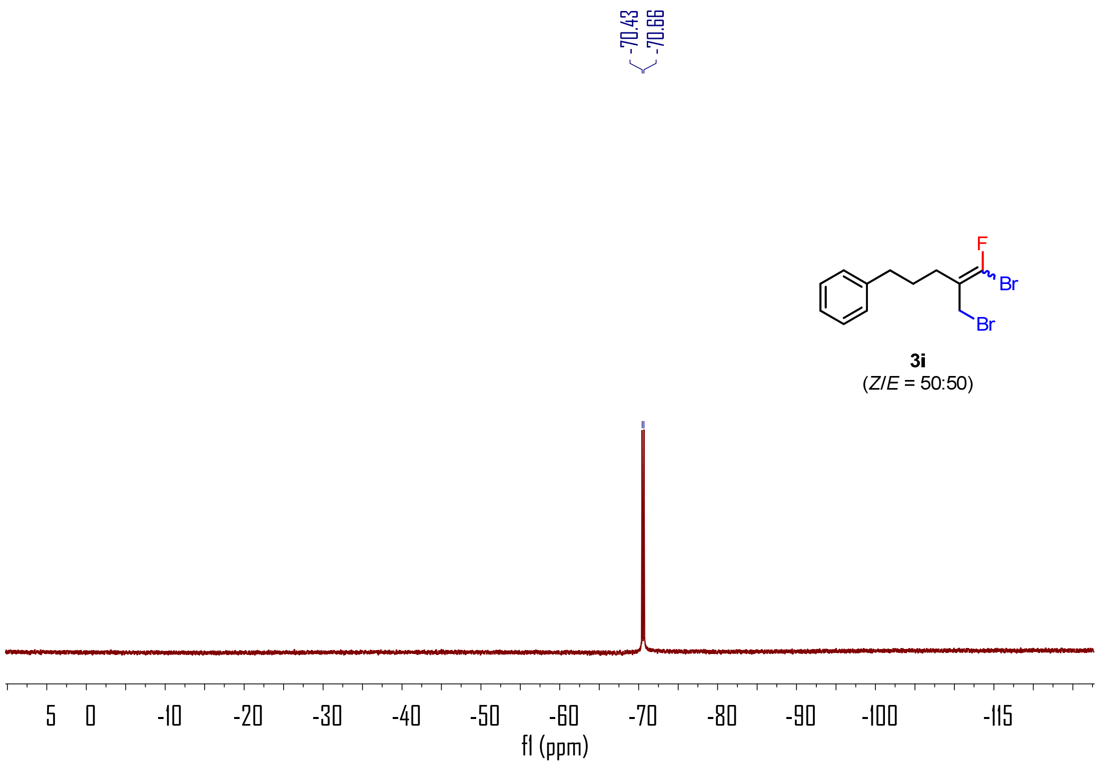

^{1}H NMR Spectrum (in $CDCl_3$, 400 MHz)

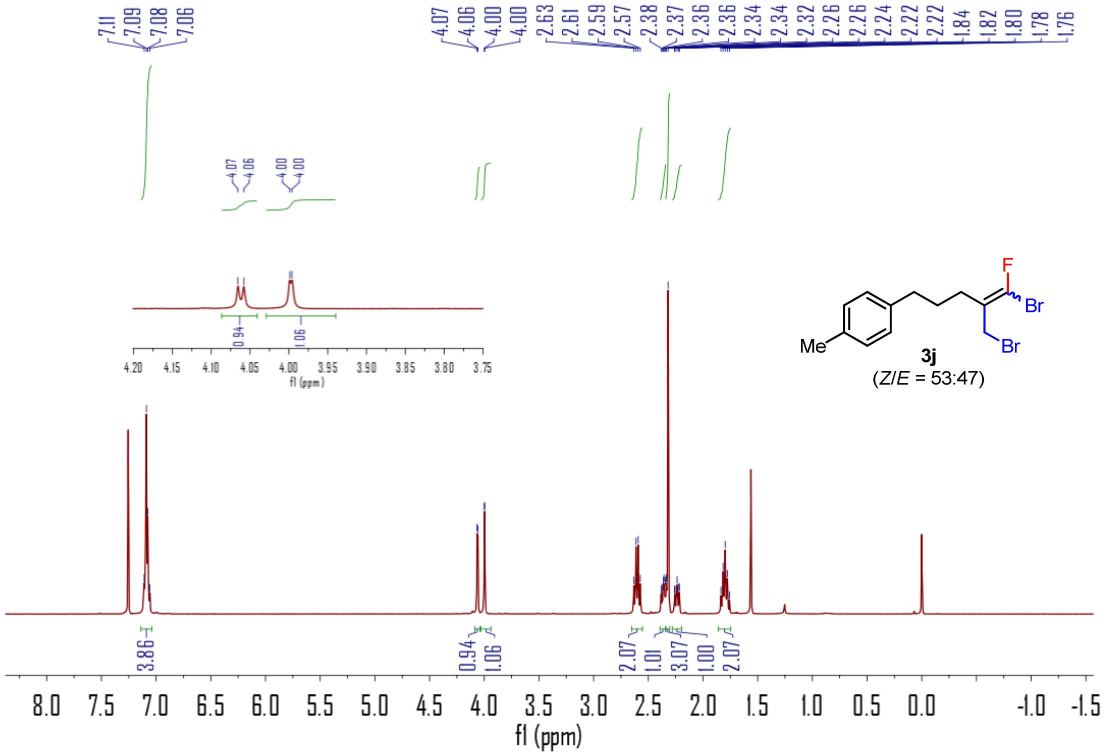

^{13}C NMR Spectrum (in $CDCl_3$, 101 MHz)

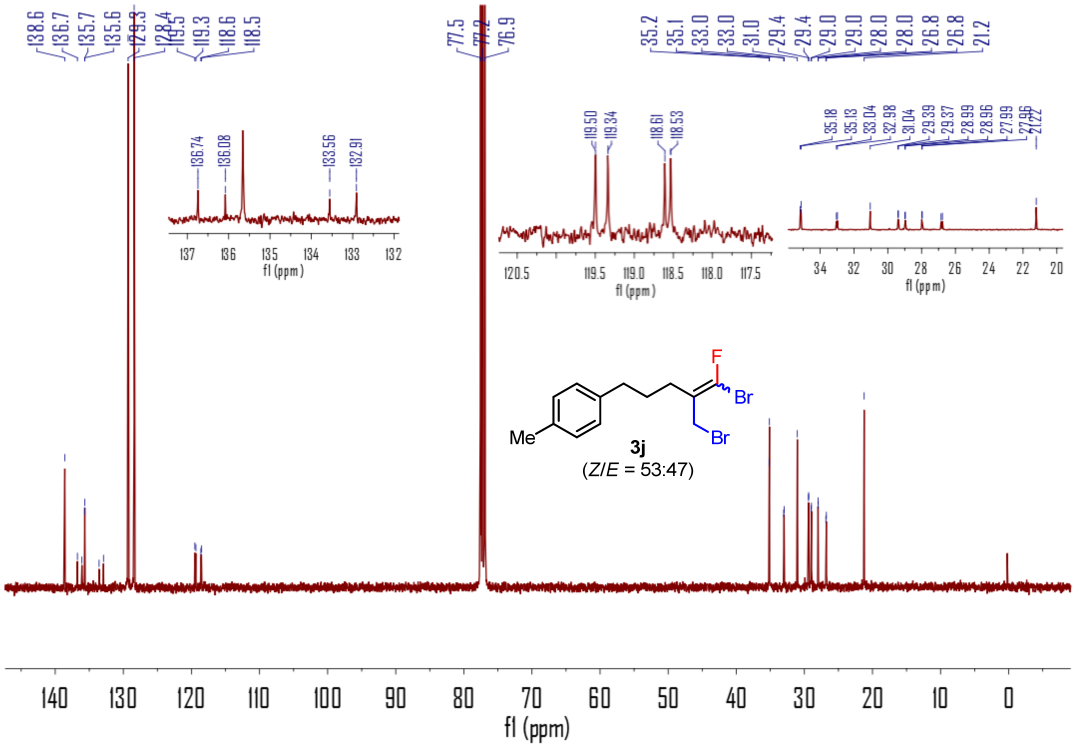

^{19}F NMR Spectrum (in $CDCl_3$, 376 MHz)

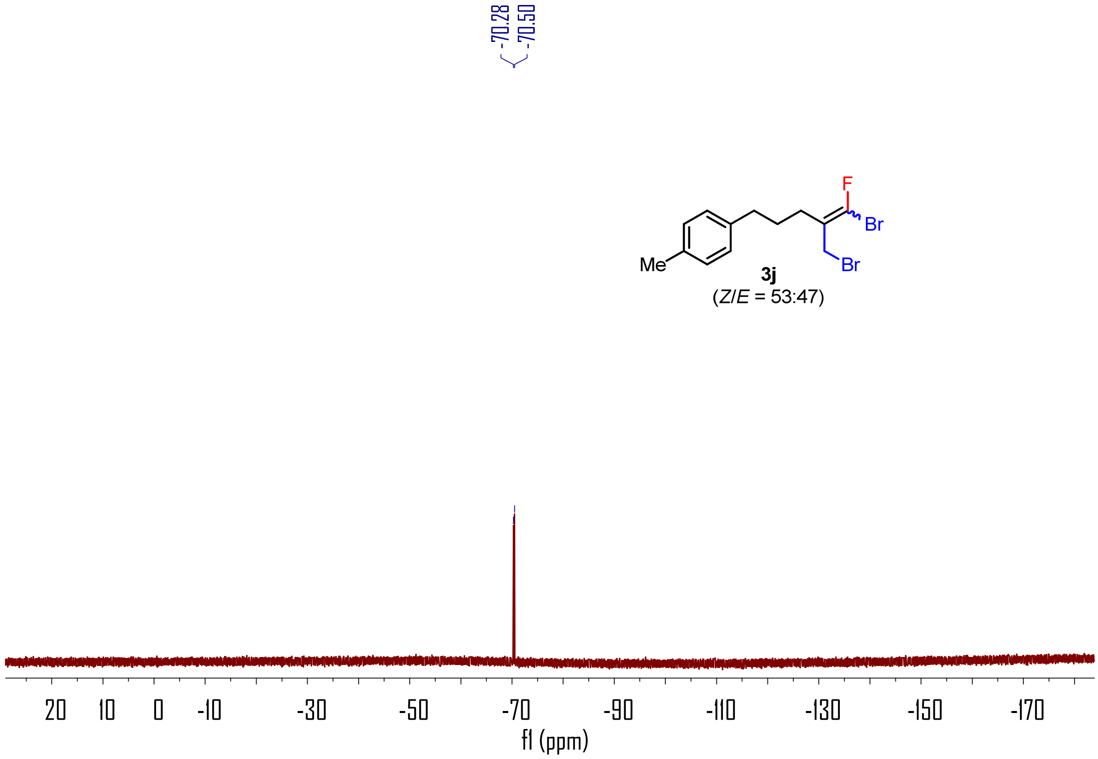

^{1}H NMR Spectrum (in $CDCl_3$, 400 MHz)

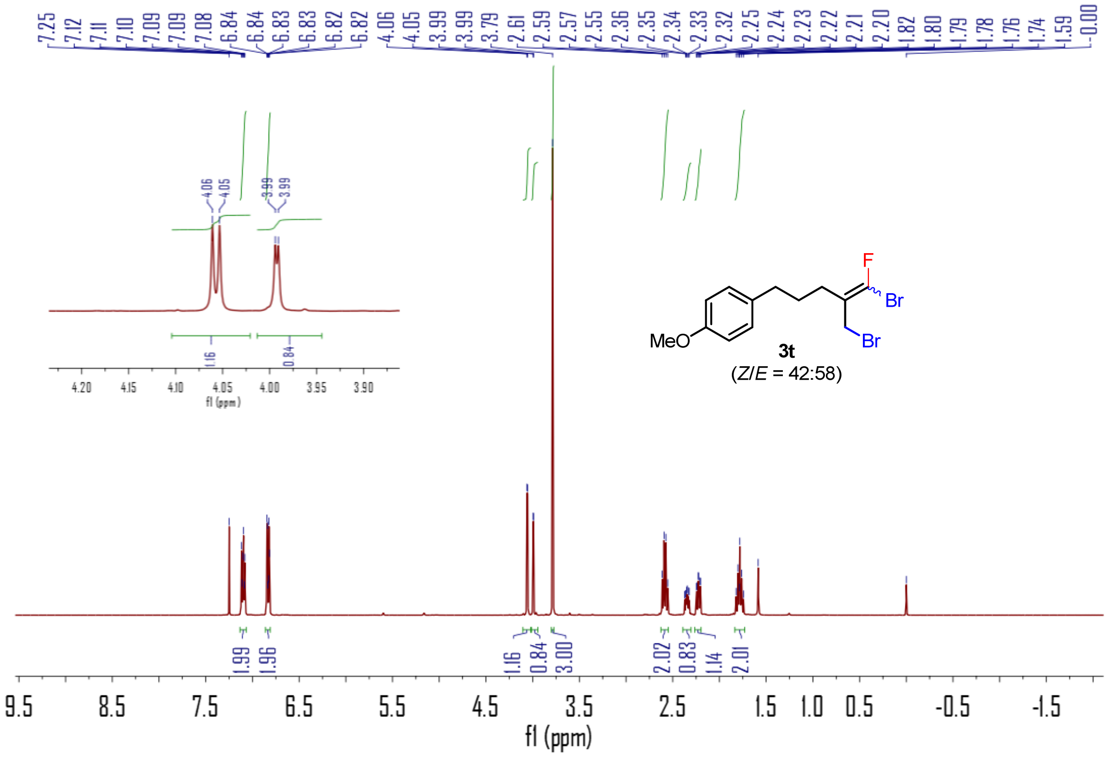

^{13}C NMR Spectrum (in $CDCl_3$, 101 MHz)

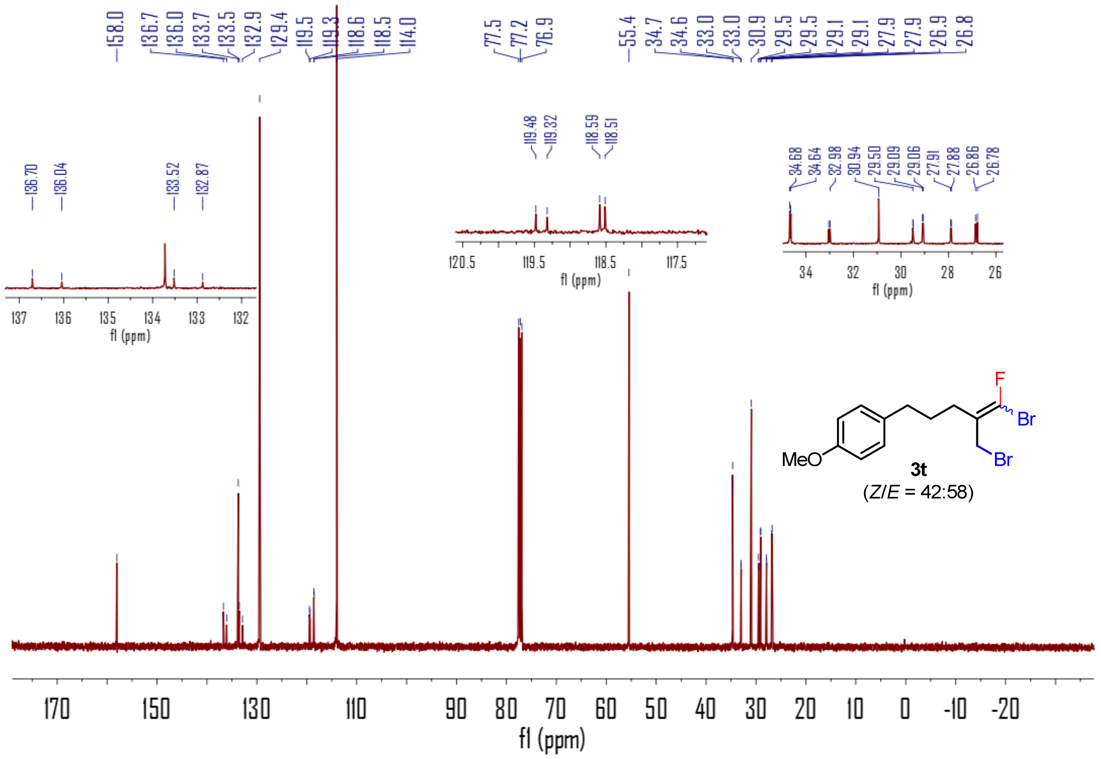

^{19}F NMR Spectrum (in $CDCl_3$, 376 MHz)

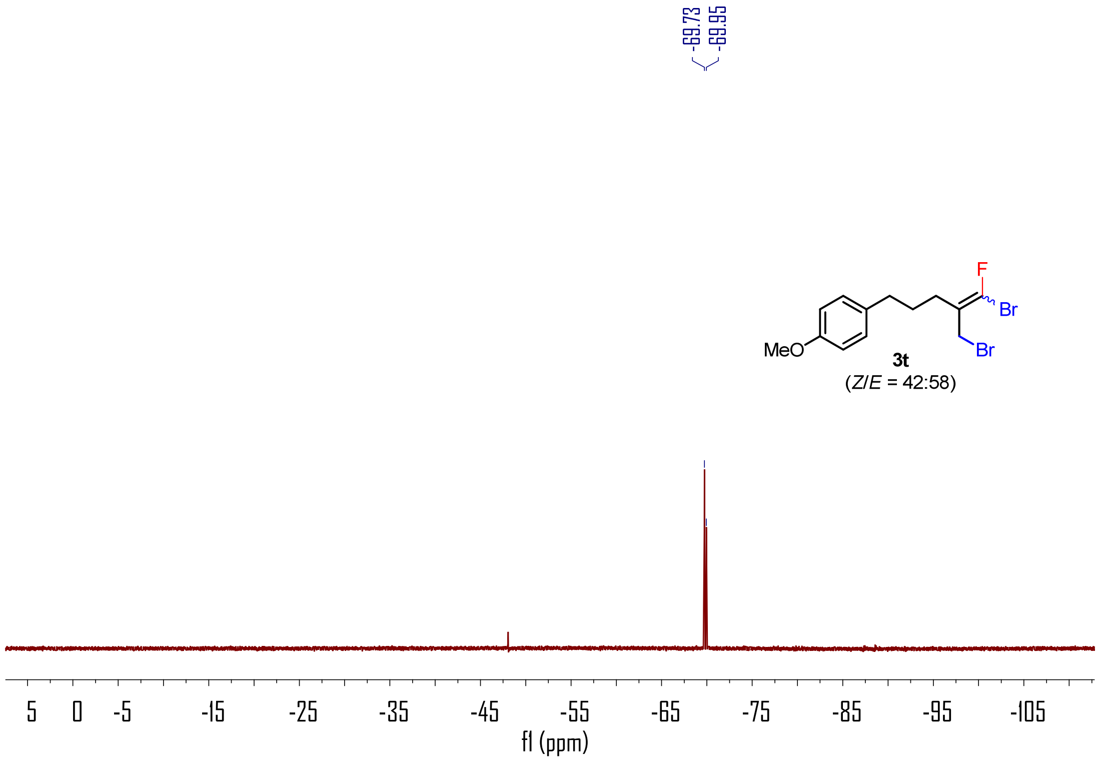

^{1}H NMR Spectrum (in CDCl$_3$, 400 MHz)

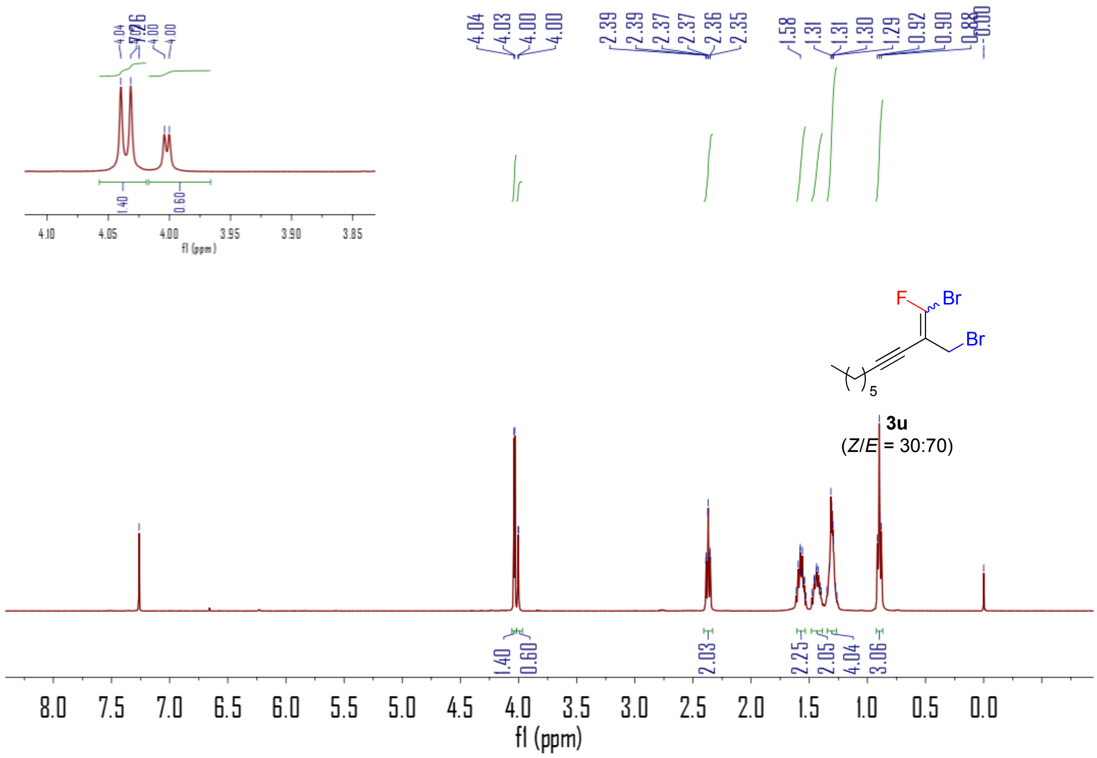

^{13}C NMR Spectrum (in CDCl$_3$, 101 MHz)

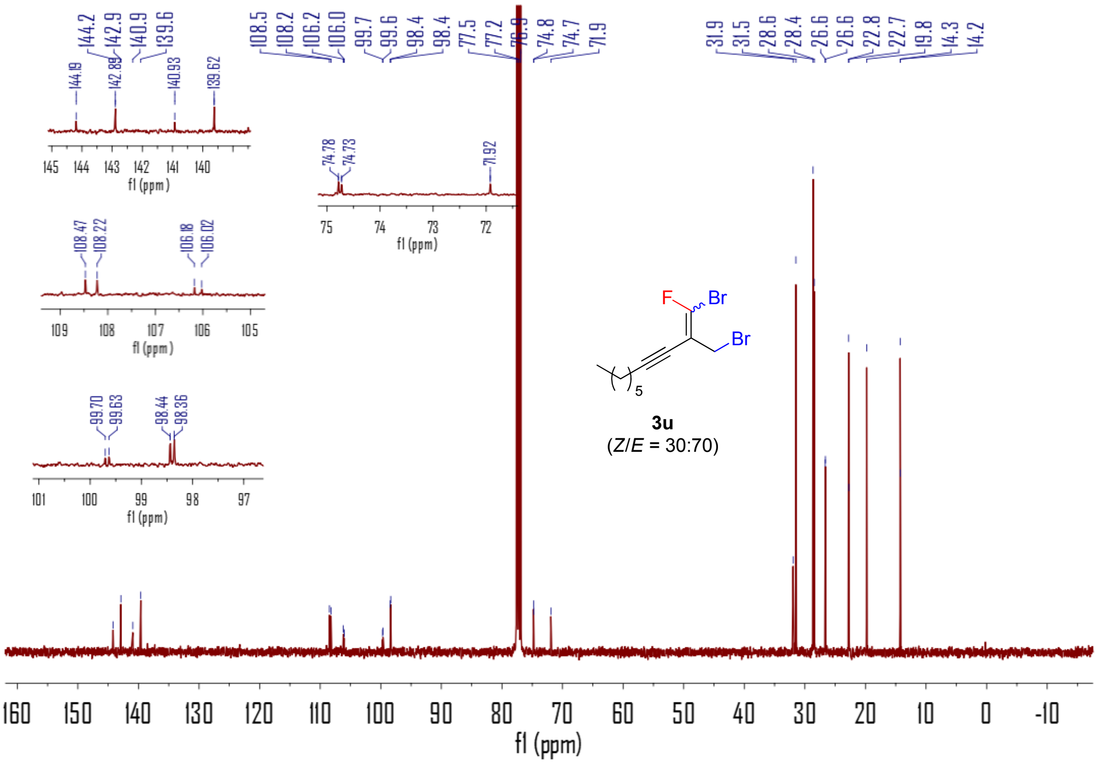

^{19}F NMR Spectrum (in $CDCl_3$, 376 MHz)

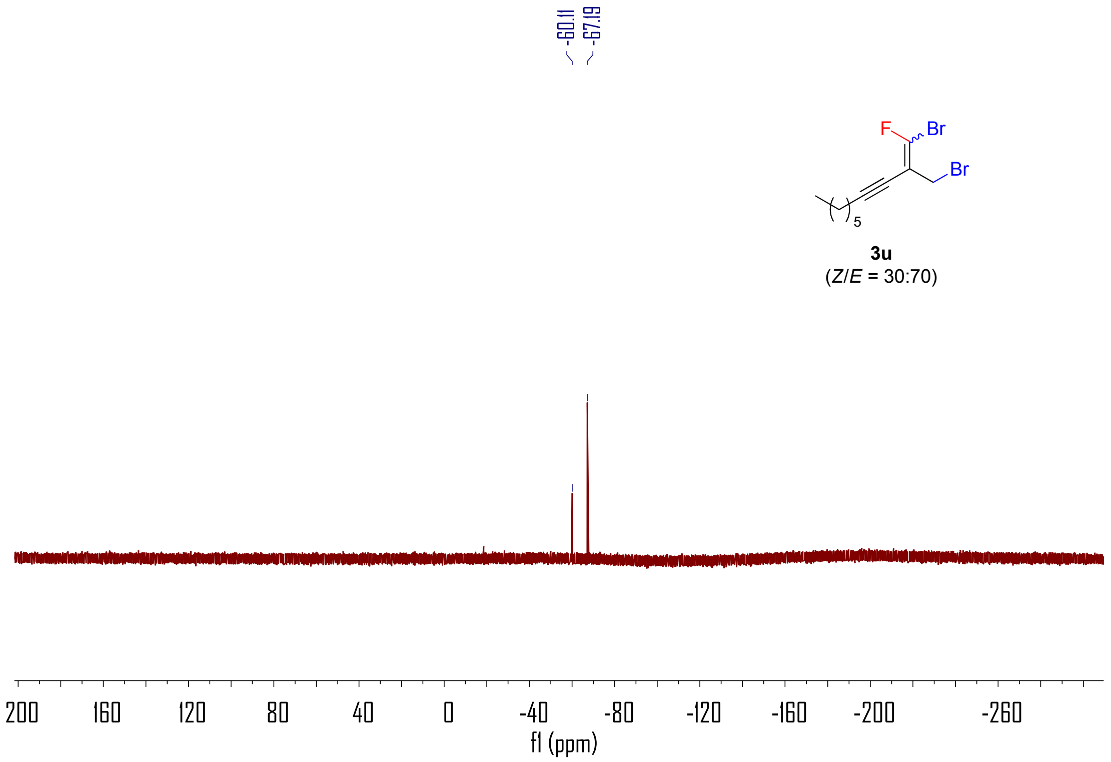

^{1}H NMR Spectrum (in $CDCl_3$, 400 MHz)

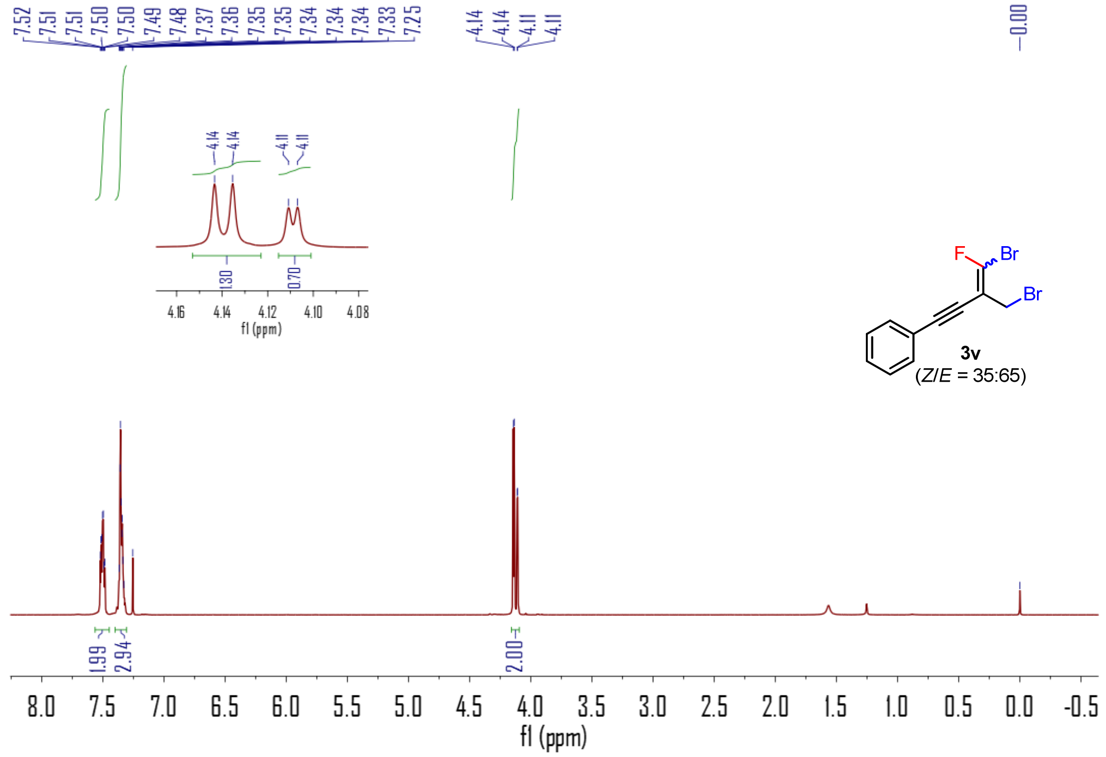

^{13}C NMR Spectrum (in $CDCl_3$, 101 MHz)

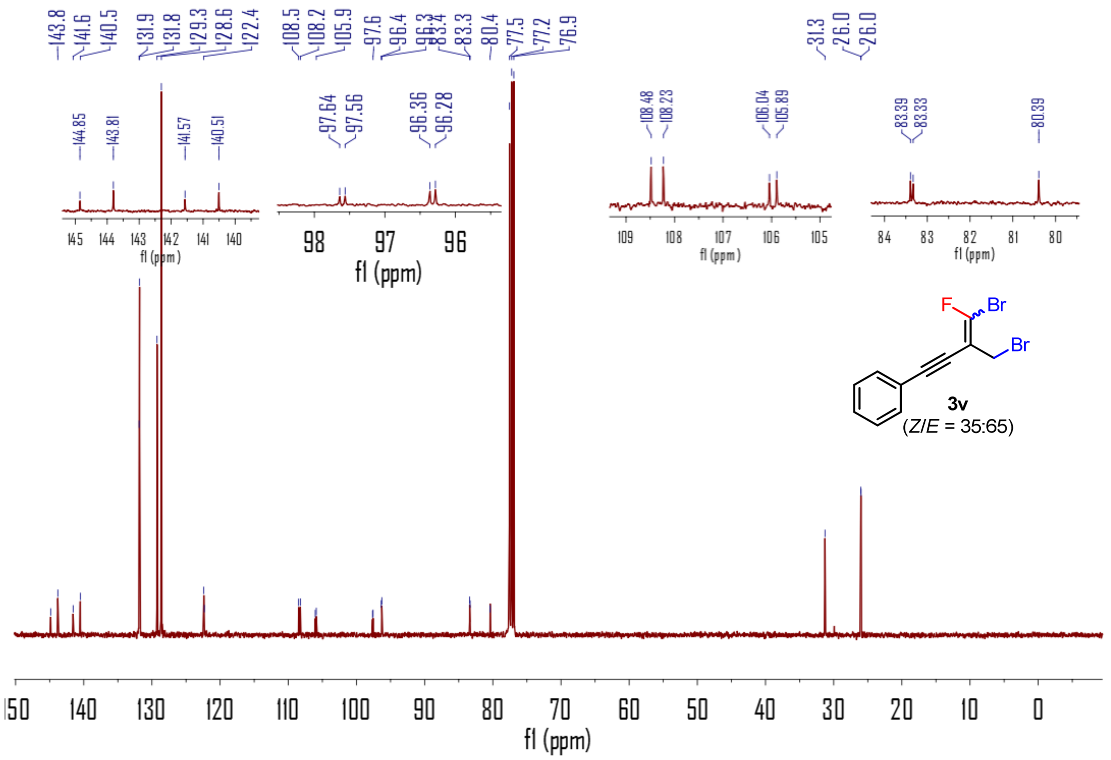

^{19}F NMR Spectrum (in $CDCl_3$, 376 MHz)

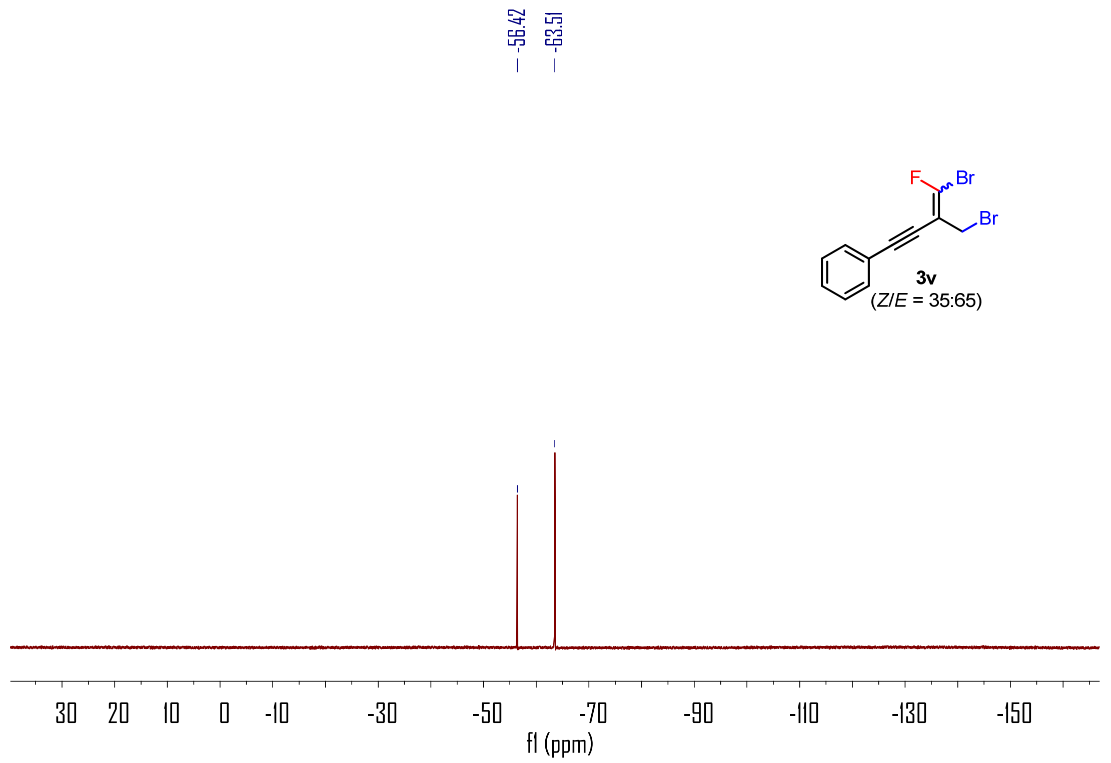

^{1}H NMR Spectrum (in $CDCl_3$, 400 MHz)

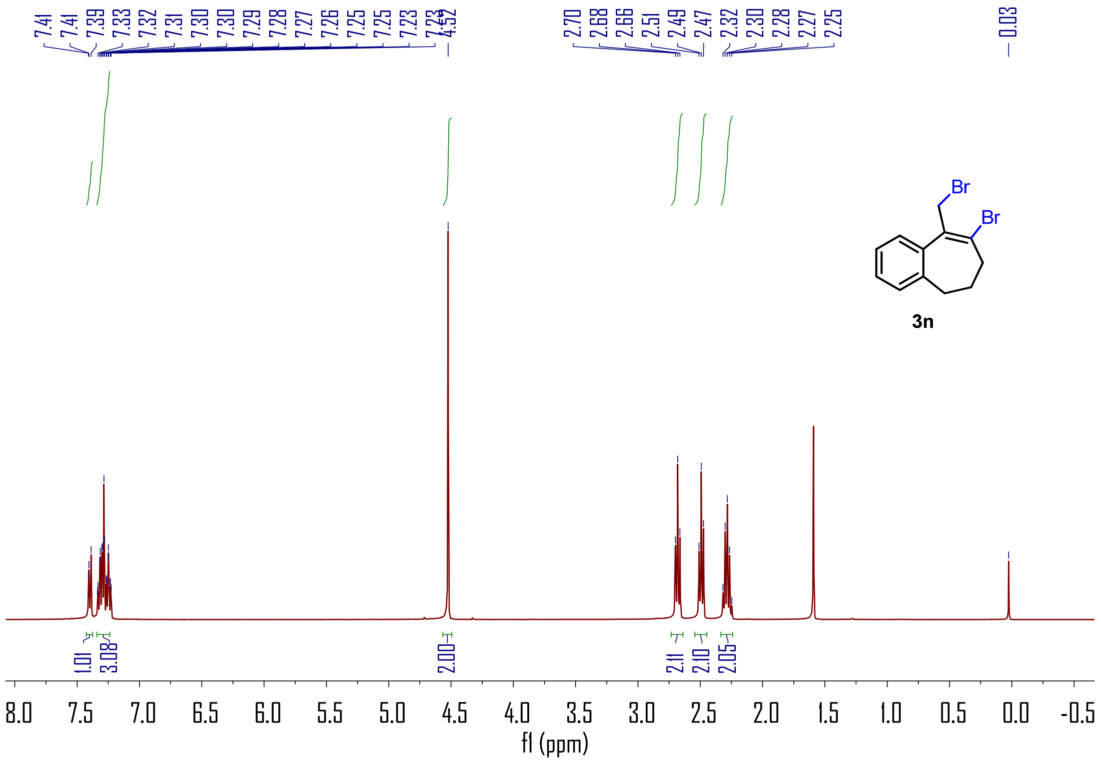

^{13}C NMR Spectrum (in $CDCl_3$, 101 MHz)

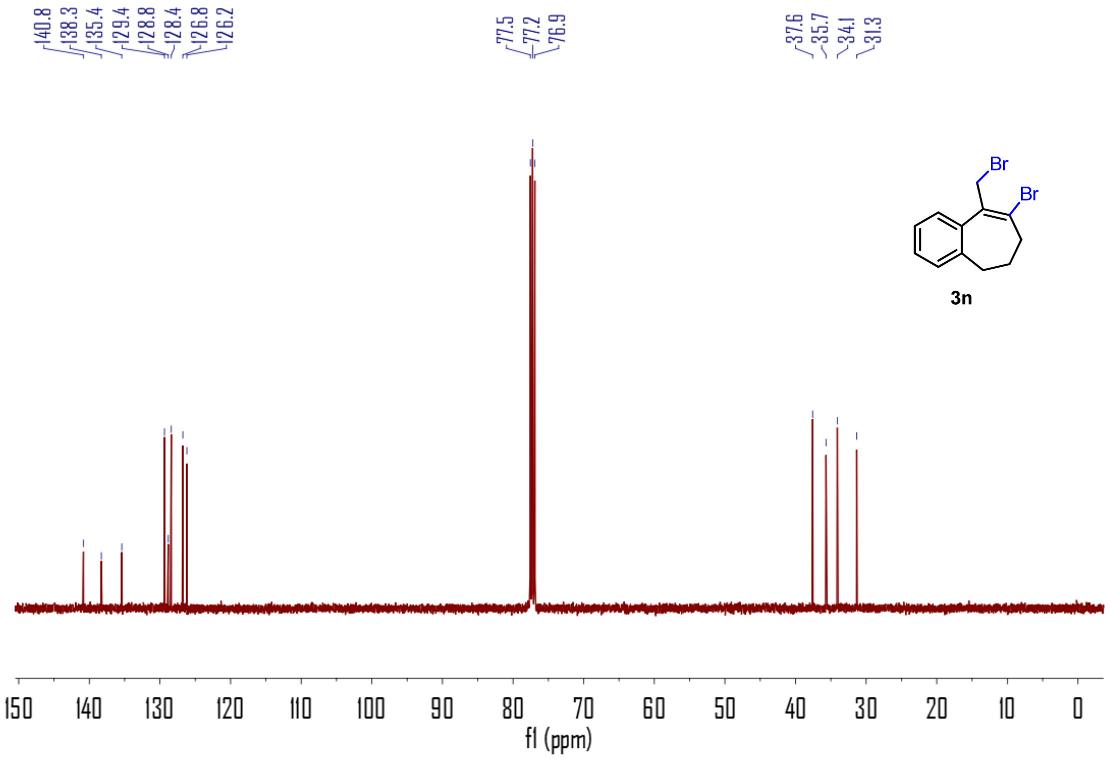

^{1}H NMR Spectrum (in CDCl3, 400 MHz)

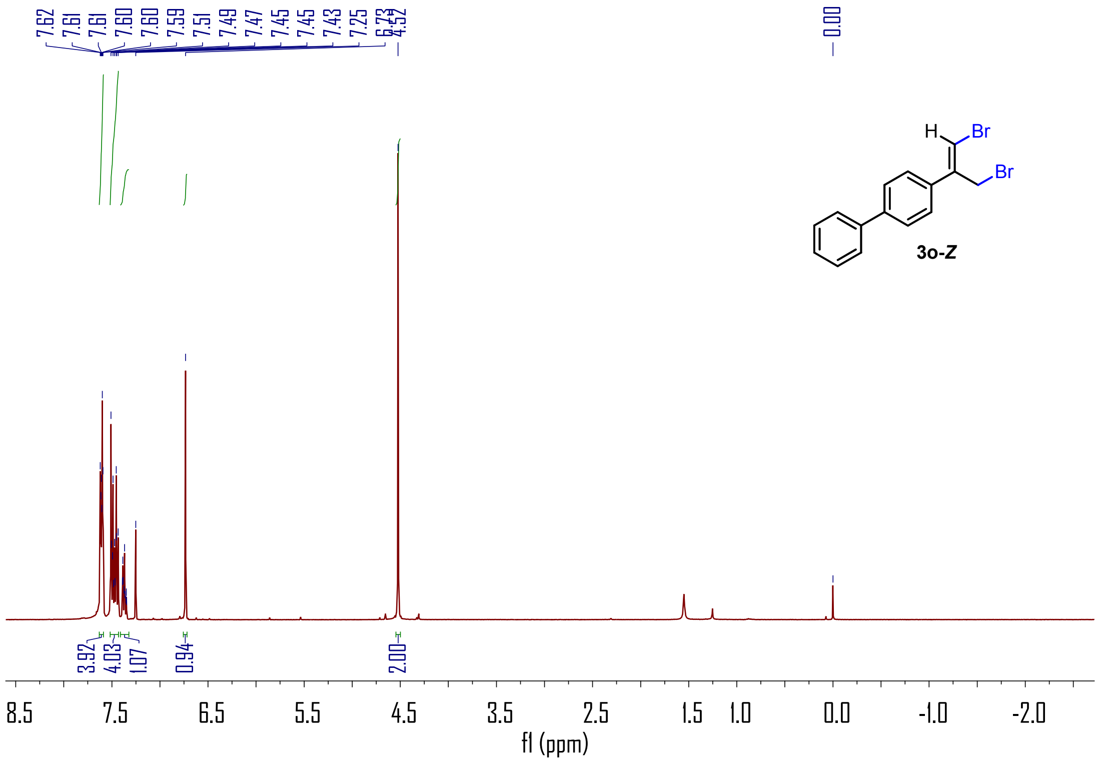

^{13}C NMR Spectrum (in CDCl3, 101 MHz)

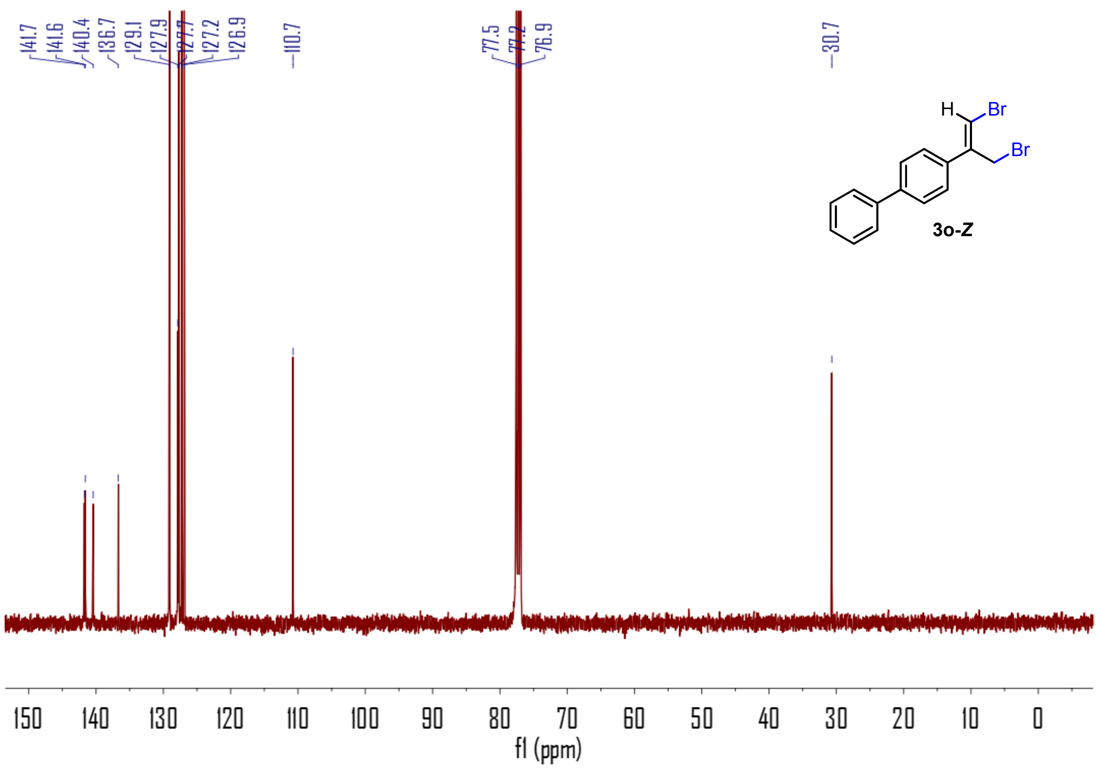

^{1}H NMR Spectrum (in $CDCl_3$, 400 MHz)

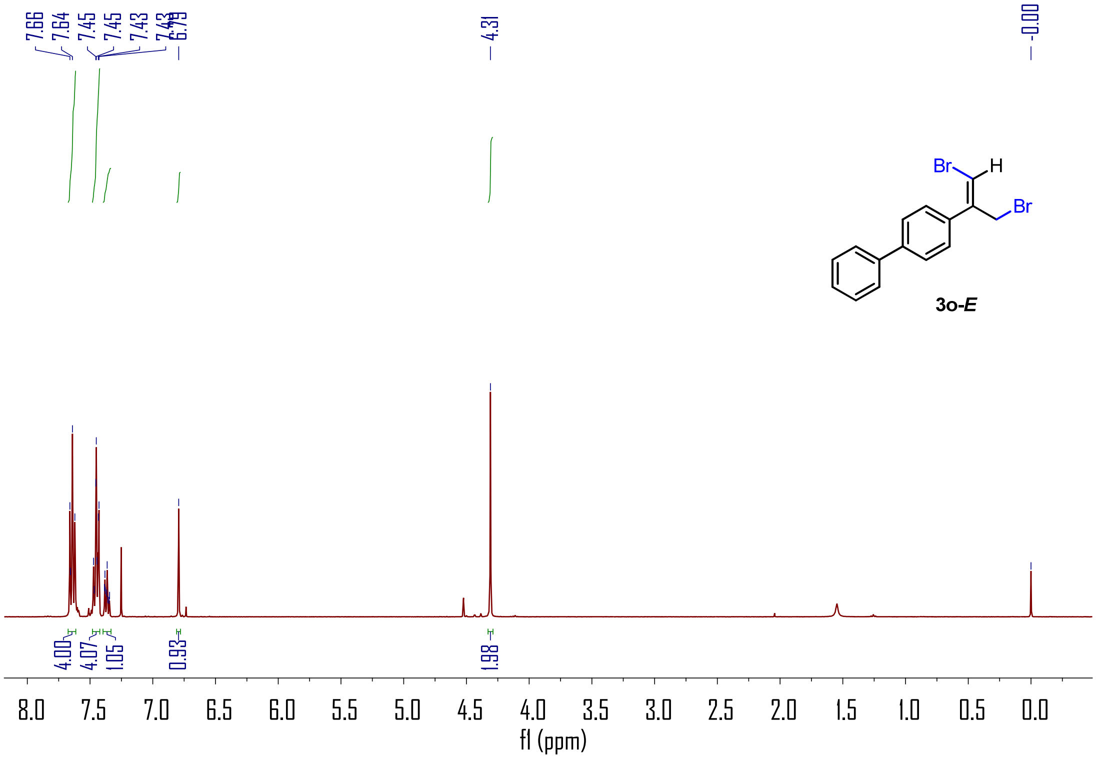

^{13}C NMR Spectrum (in $CDCl_3$, 101 MHz)

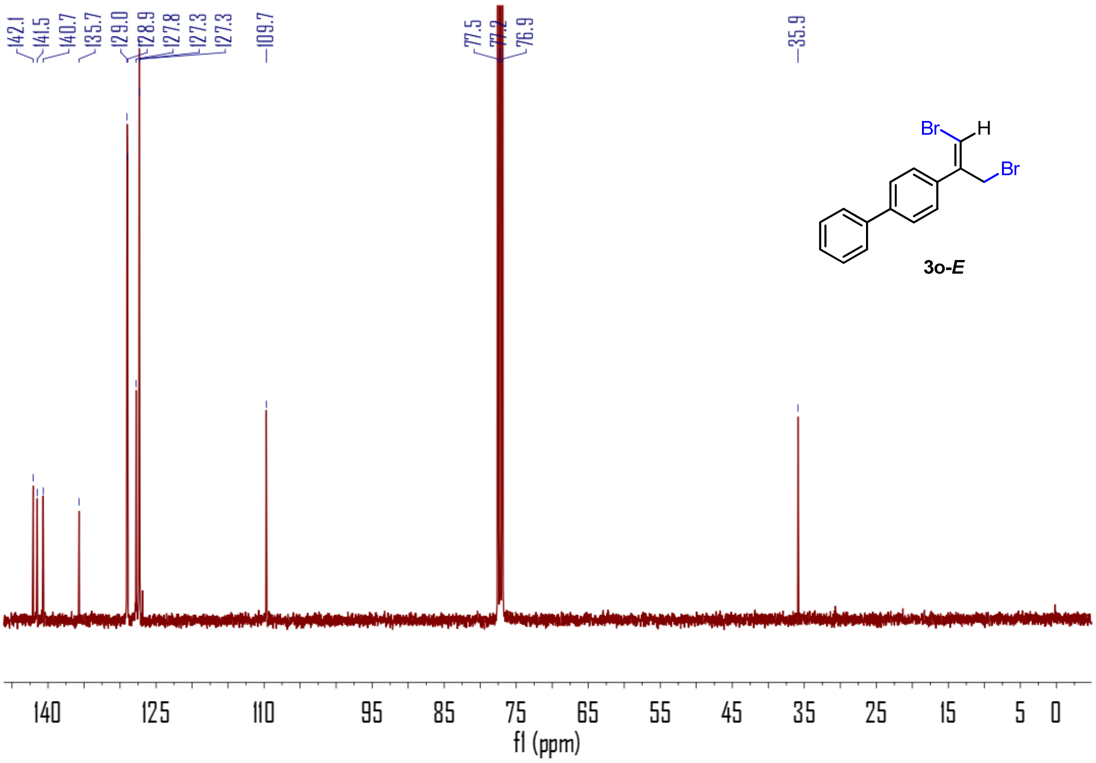

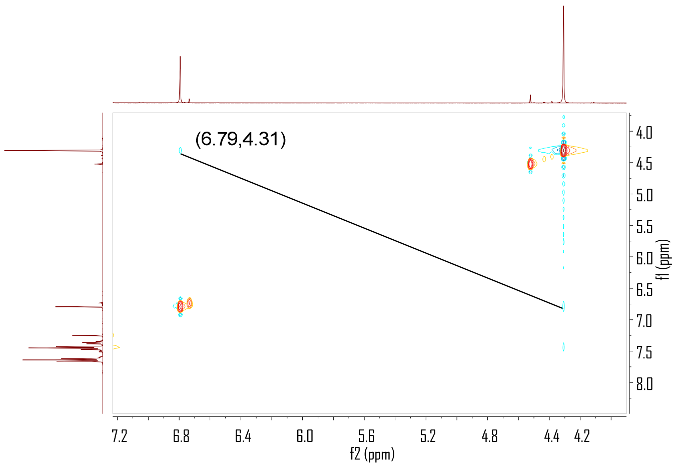

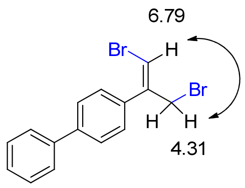

3o-*E*

^{1}H NMR Spectrum (in $CDCl_3$, 400 MHz)

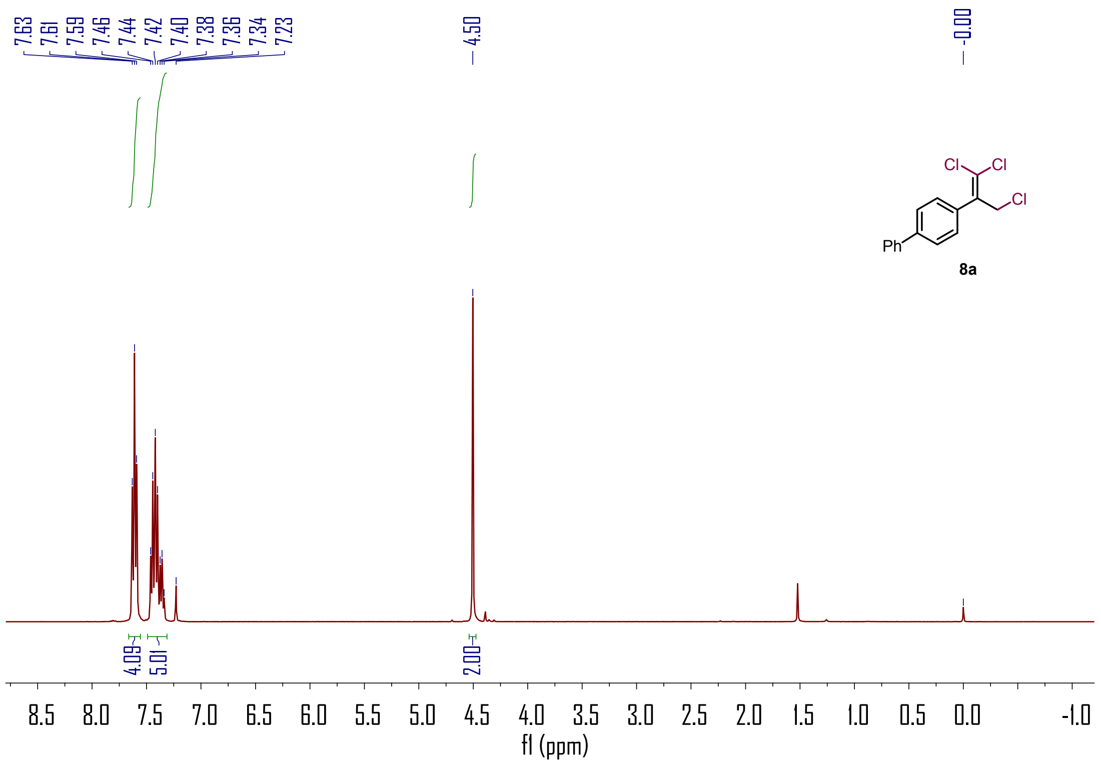

^{13}C NMR Spectrum (in $CDCl_3$, 101 MHz)

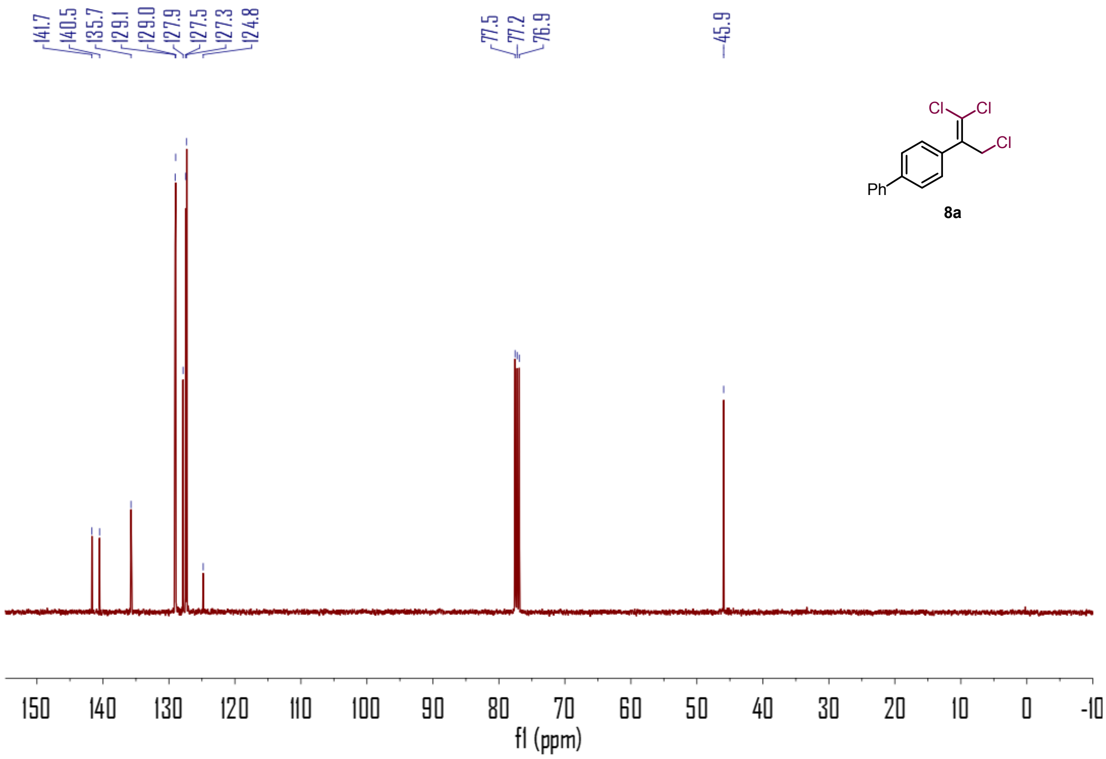

^{1}H NMR Spectrum (in CDCl3, 400 MHz)

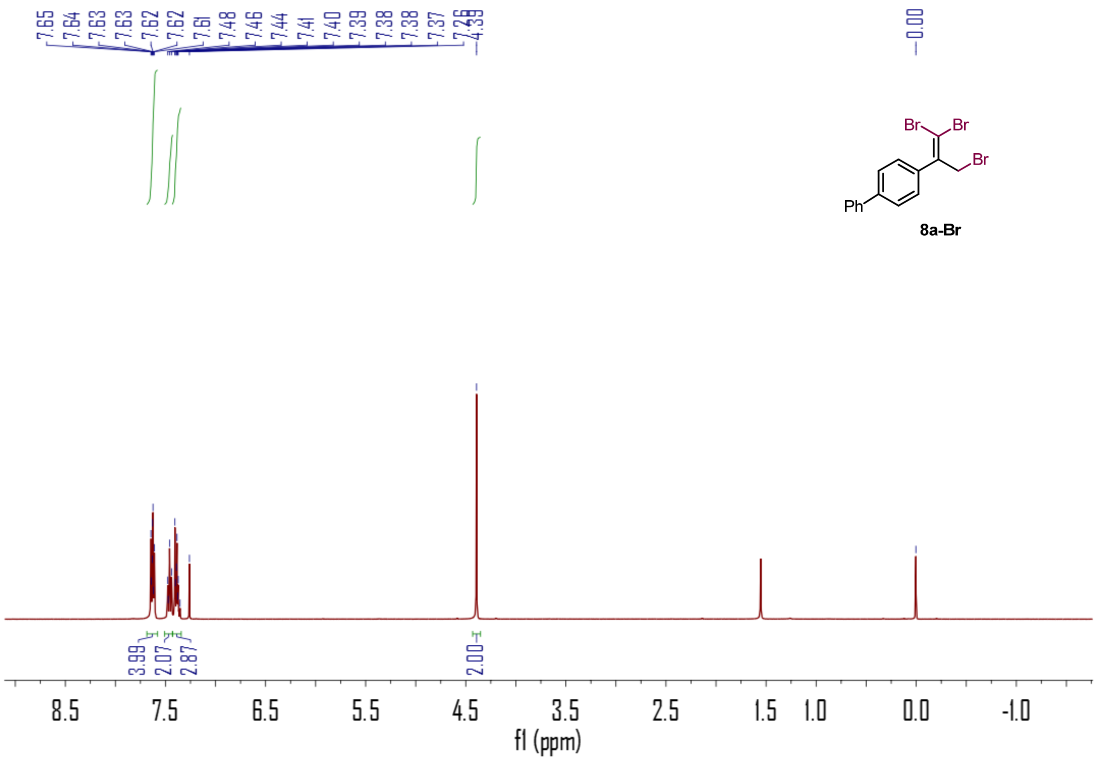

^{13}C NMR Spectrum (in CDCl3, 101 MHz)

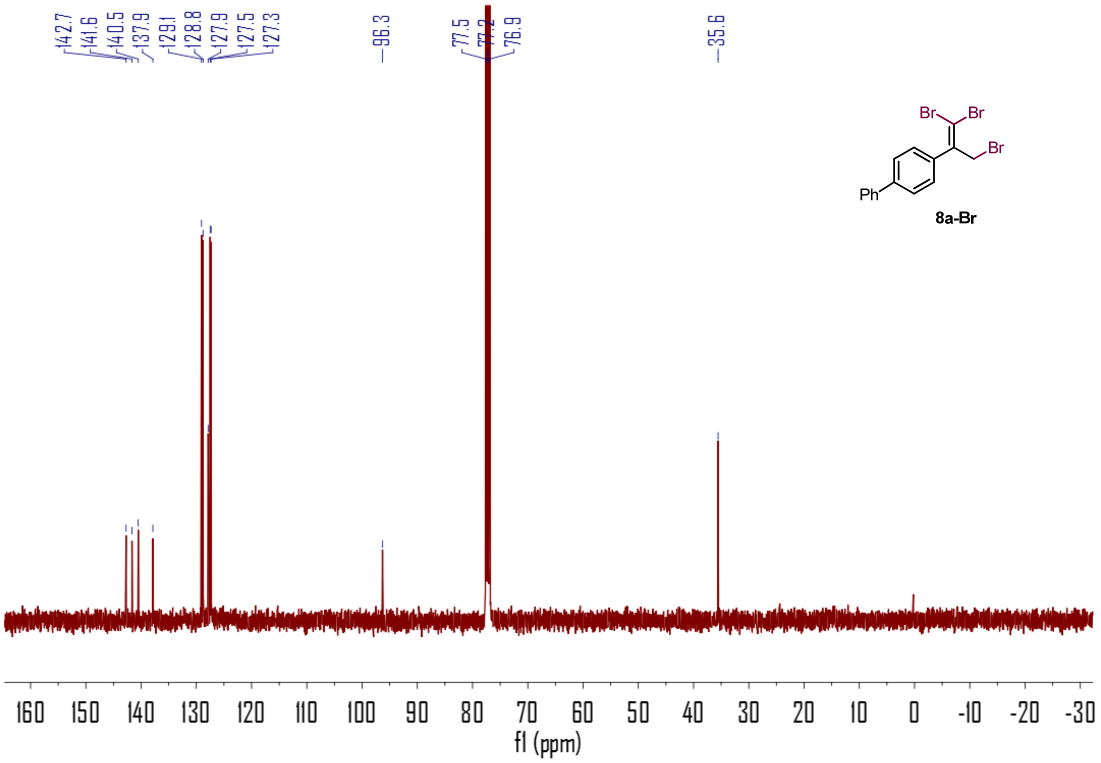

^{1}H NMR Spectrum (in $CDCl_3$, 400 MHz)

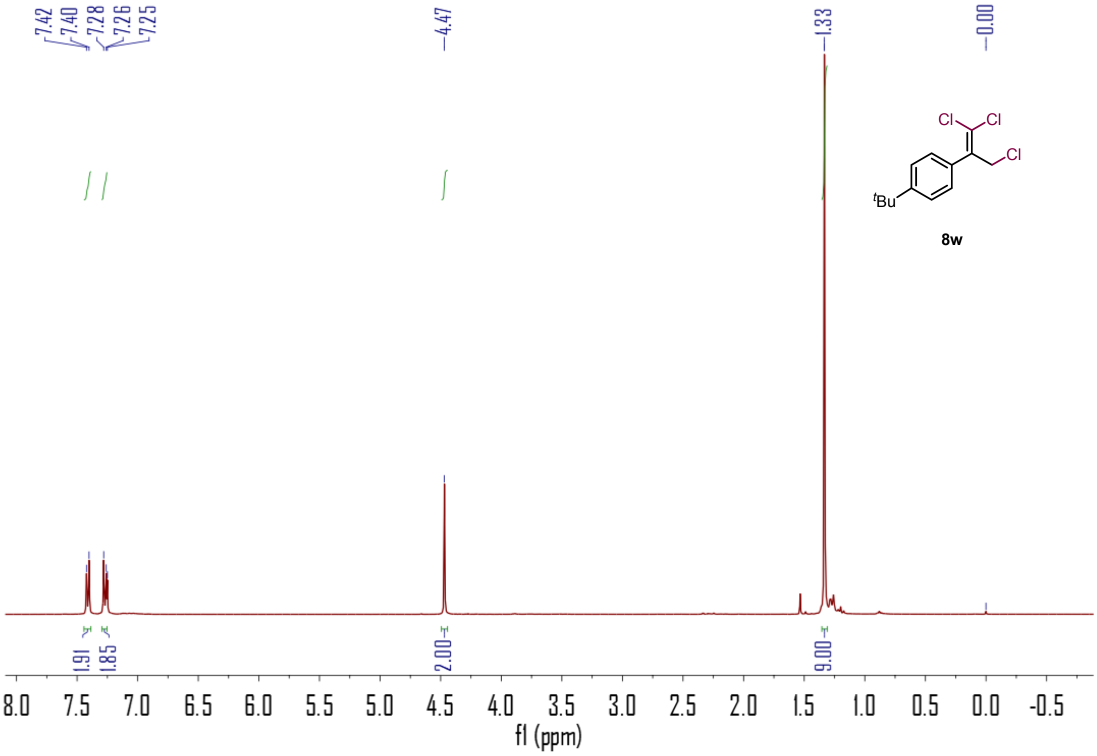

^{13}C NMR Spectrum (in $CDCl_3$, 101 MHz)

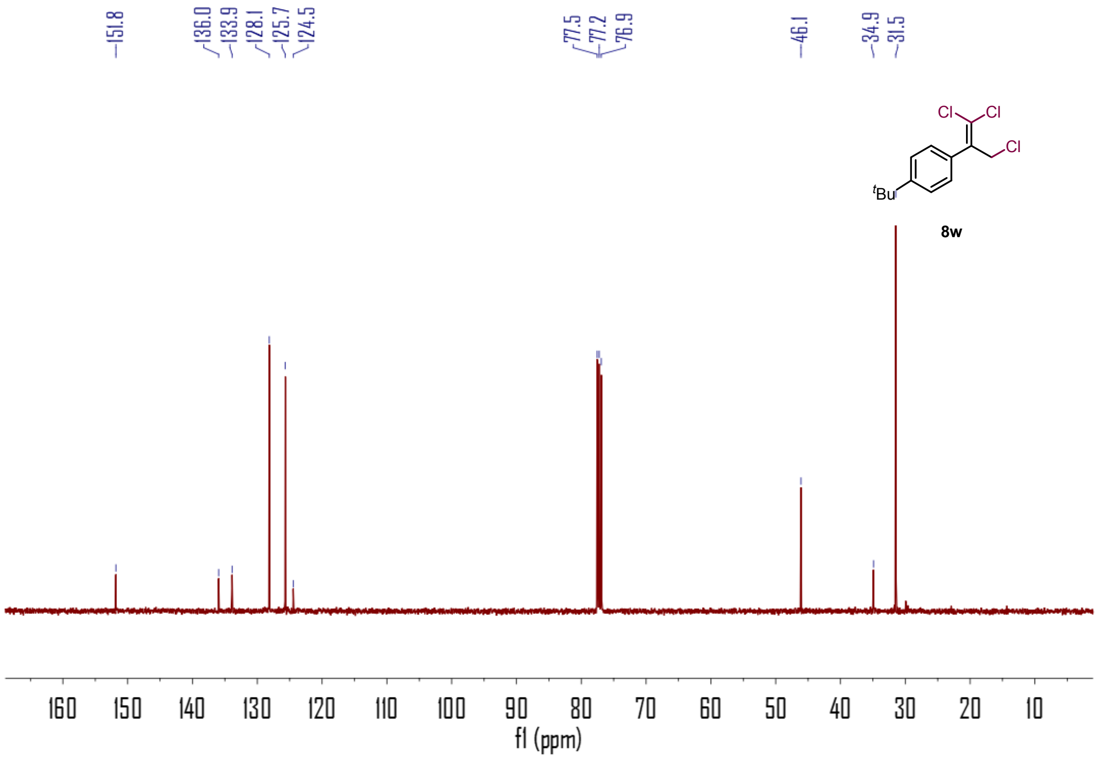

^{1}H NMR Spectrum (in $CDCl_3$, 400 MHz)

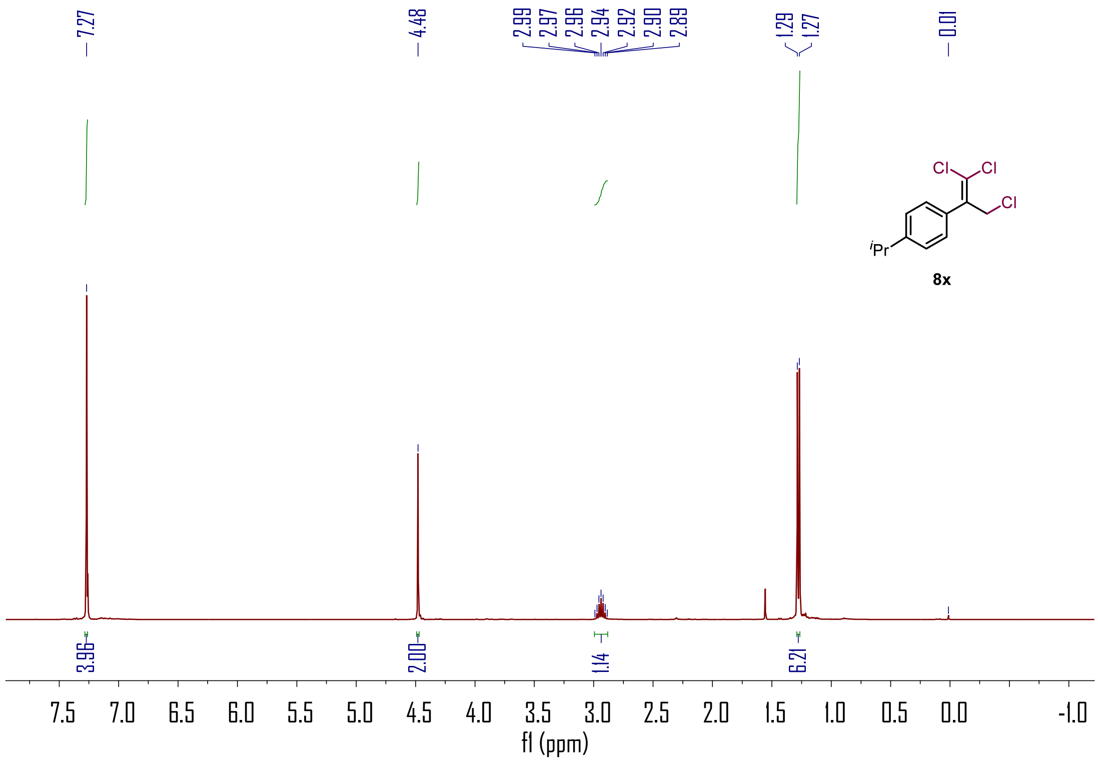

^{13}C NMR Spectrum (in $CDCl_3$, 101 MHz)

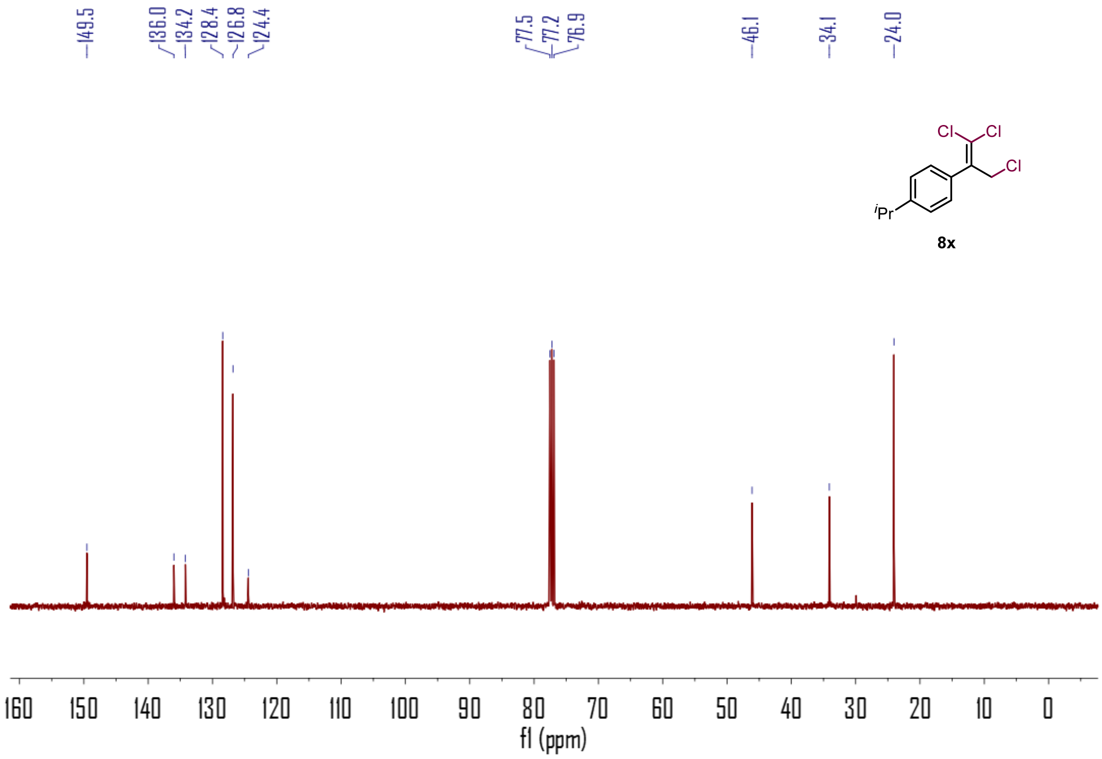

^{1}H NMR Spectrum (in $CDCl_3$, 400 MHz)

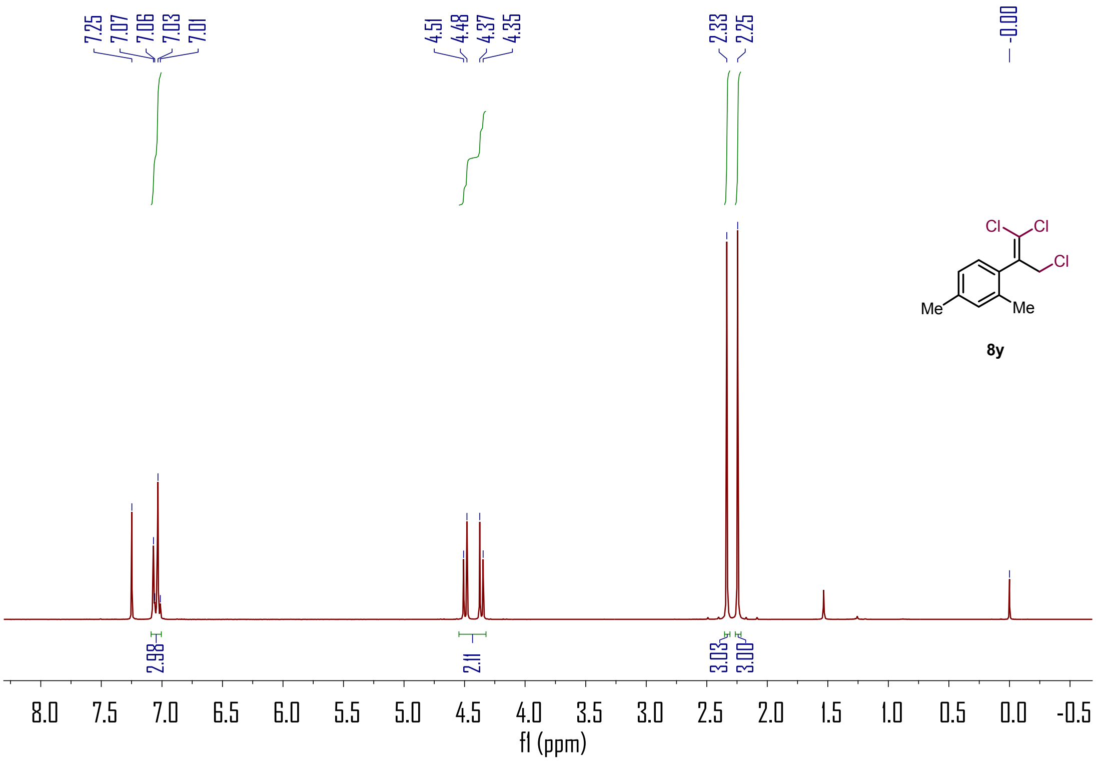

^{13}C NMR Spectrum (in $CDCl_3$, 101 MHz)

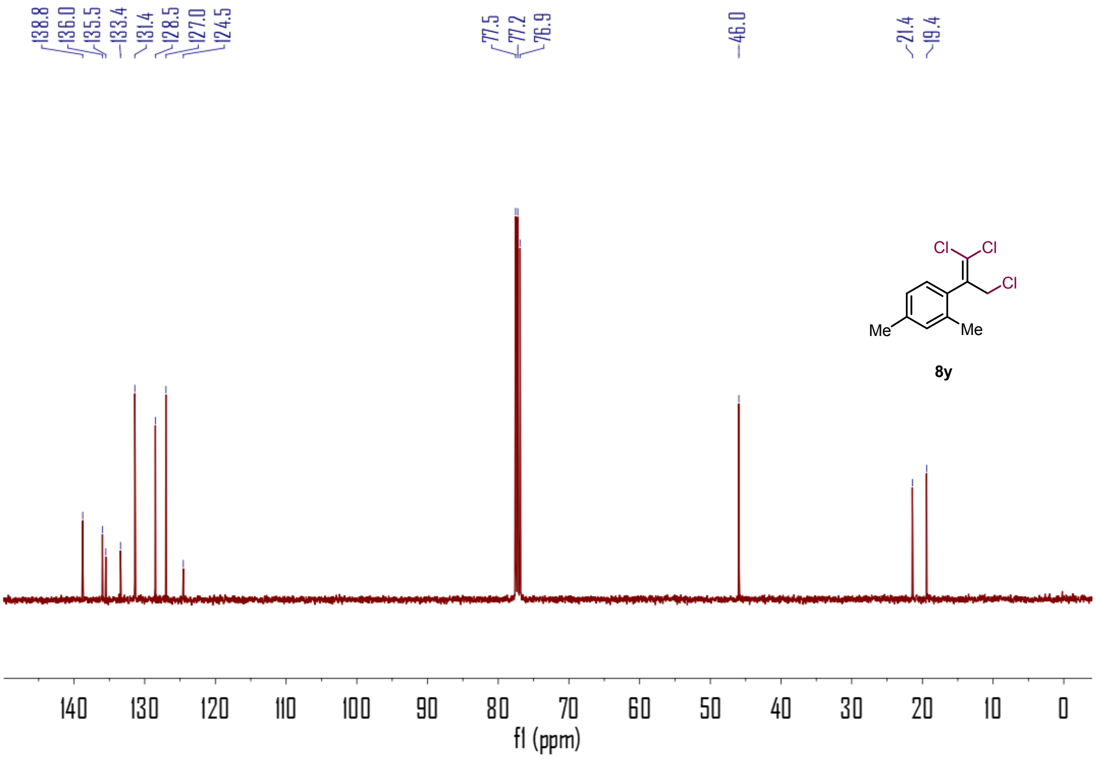

^1H NMR Spectrum (in $CDCl_3$, 400 MHz)

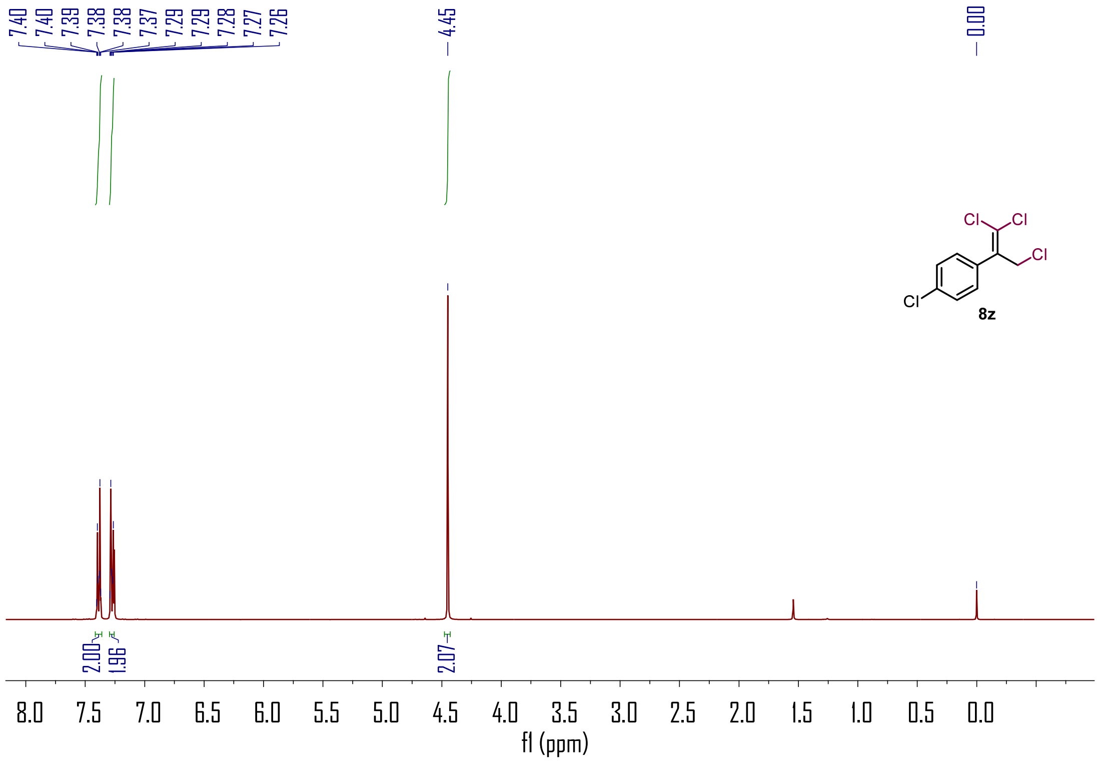

^{13}C NMR Spectrum (in $CDCl_3$, 101 MHz)

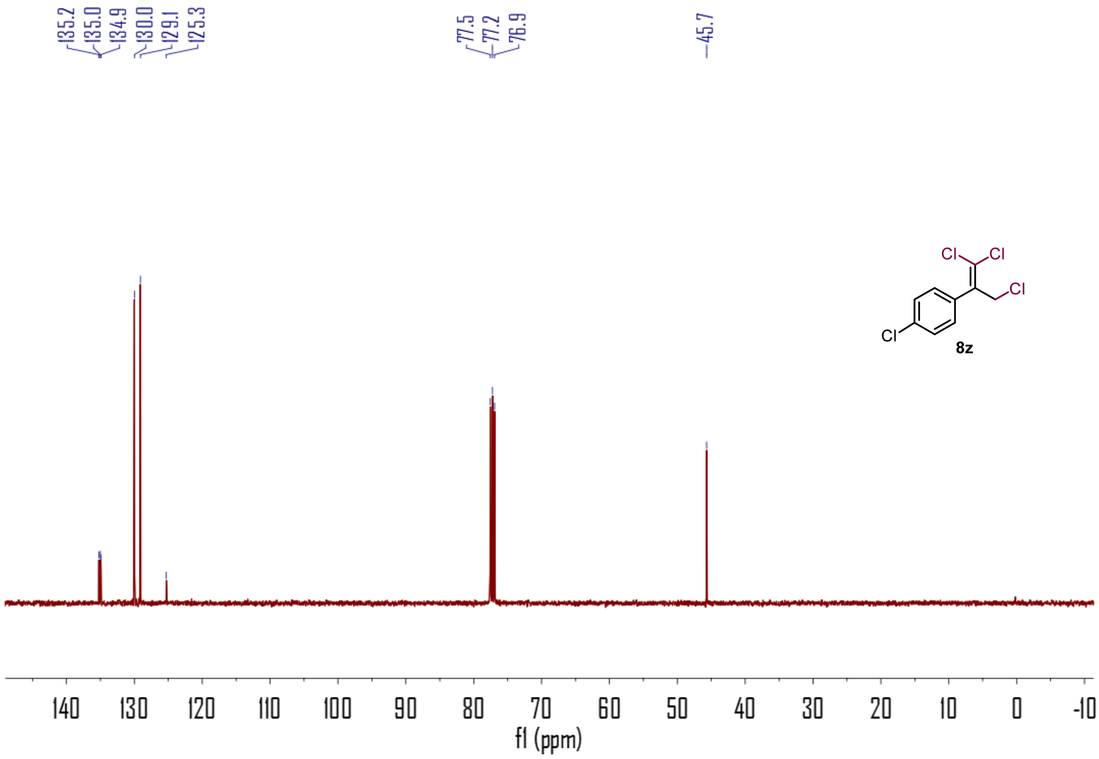

^{1}H NMR Spectrum (in $CDCl_3$, 400 MHz)

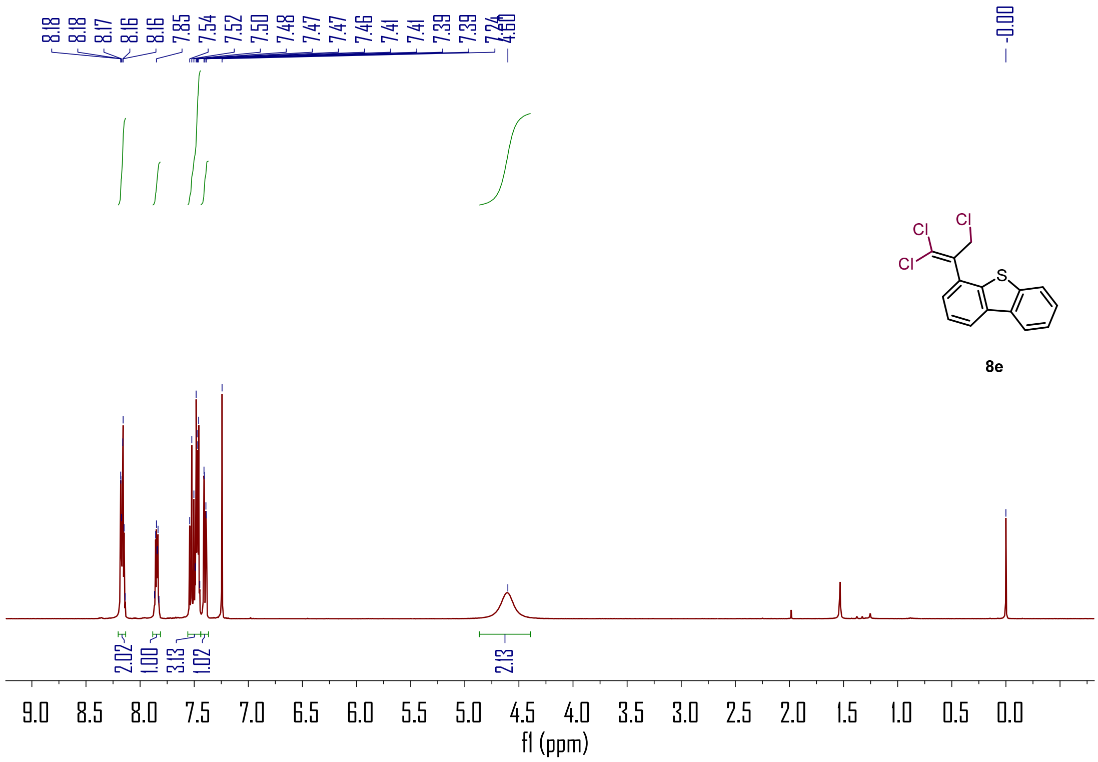

^{13}C NMR Spectrum (in $CDCl_3$, 101 MHz)

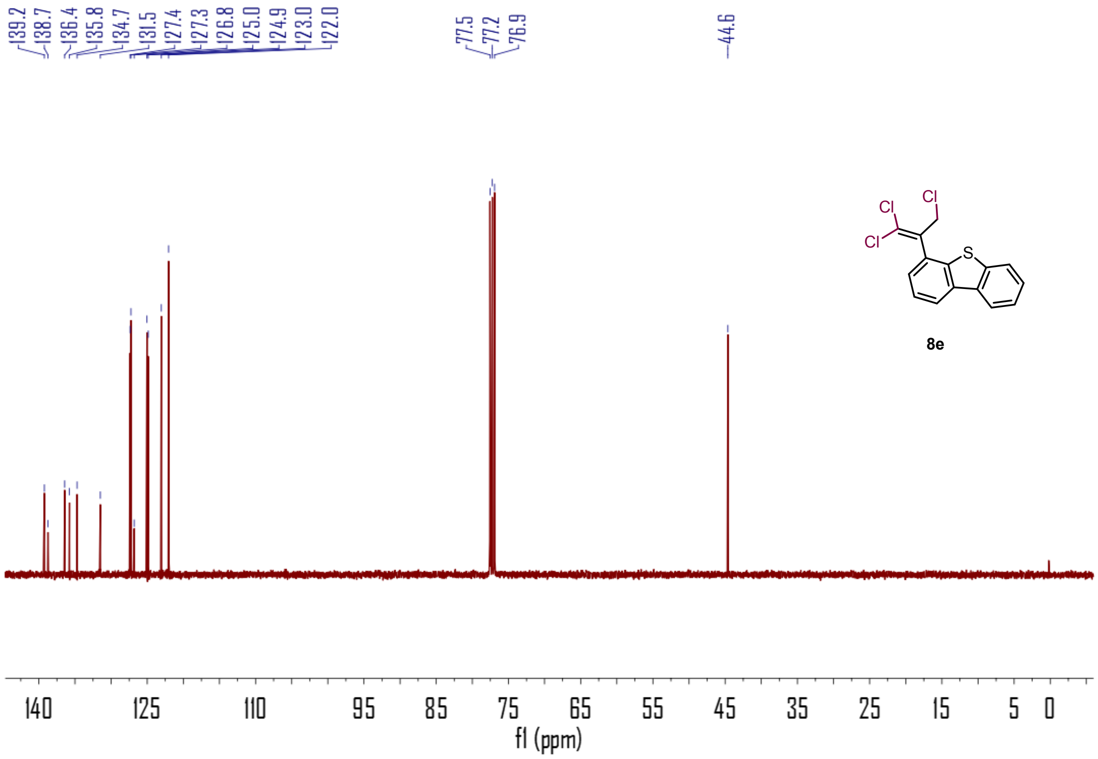

^{1}H NMR Spectrum (in $CDCl_3$, 400 MHz)

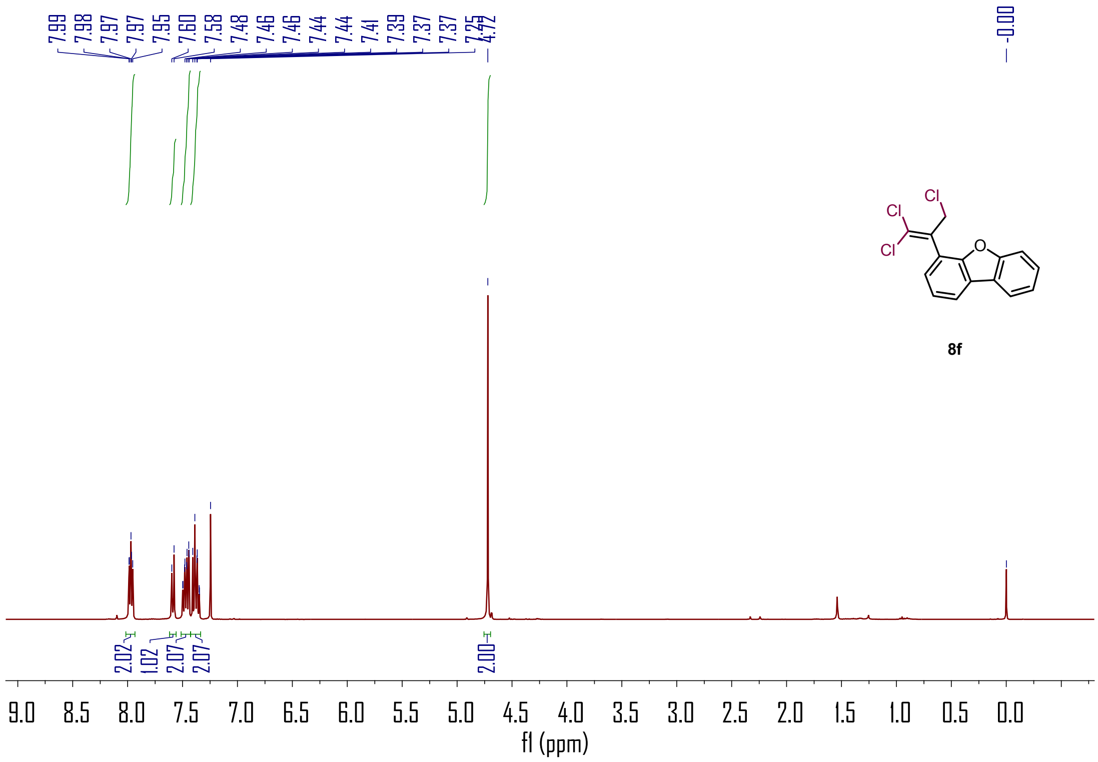

^{13}C NMR Spectrum (in $CDCl_3$, 101 MHz)

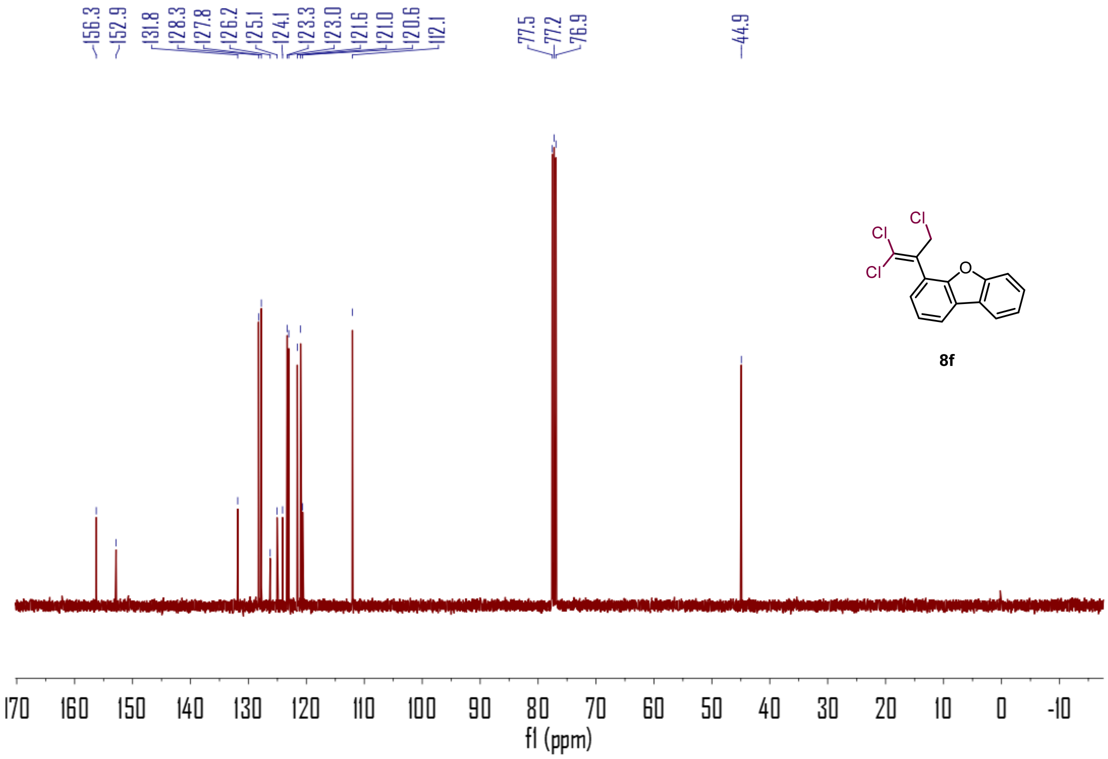

^{1}H NMR Spectrum (in CDCl3, 400 MHz)

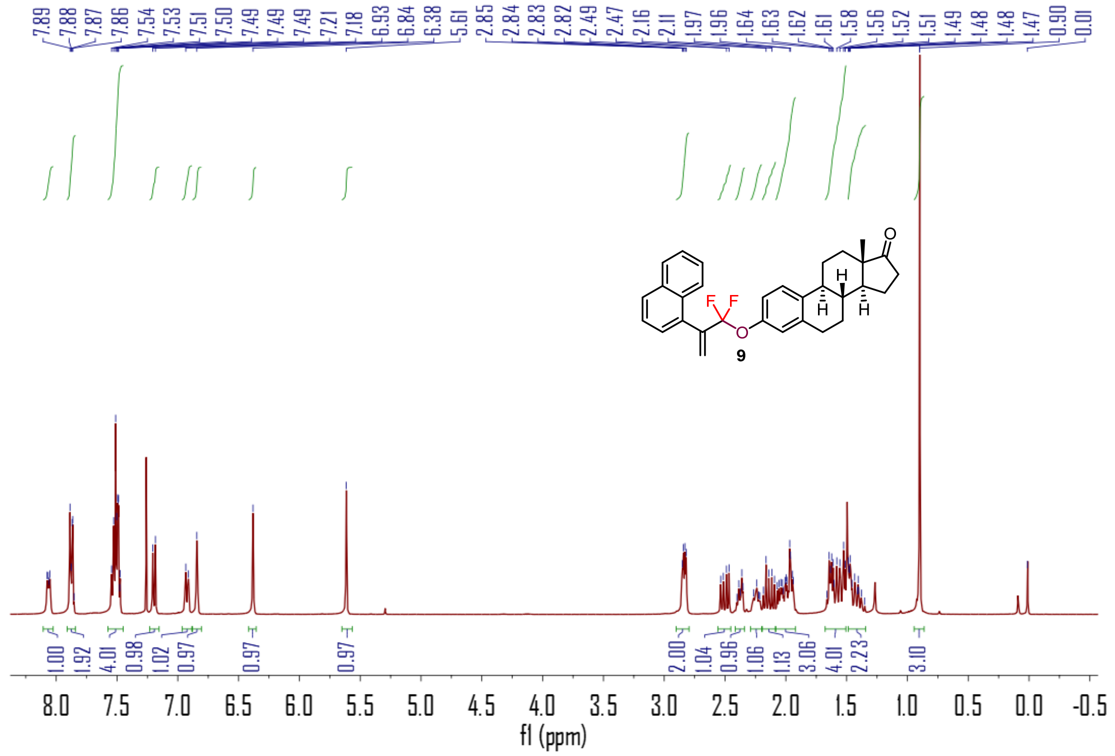

^{13}C NMR Spectrum (in CDCl3, 101 MHz)

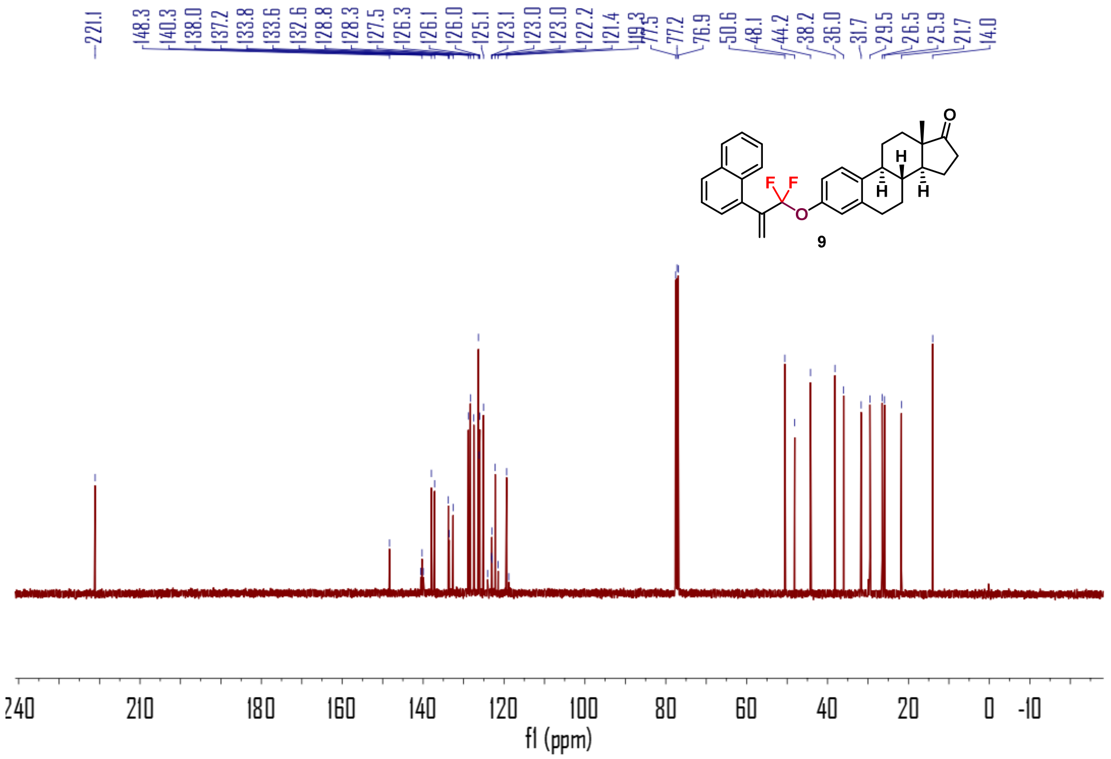

^{19}F NMR Spectrum (in $CDCl_3$, 376 MHz)

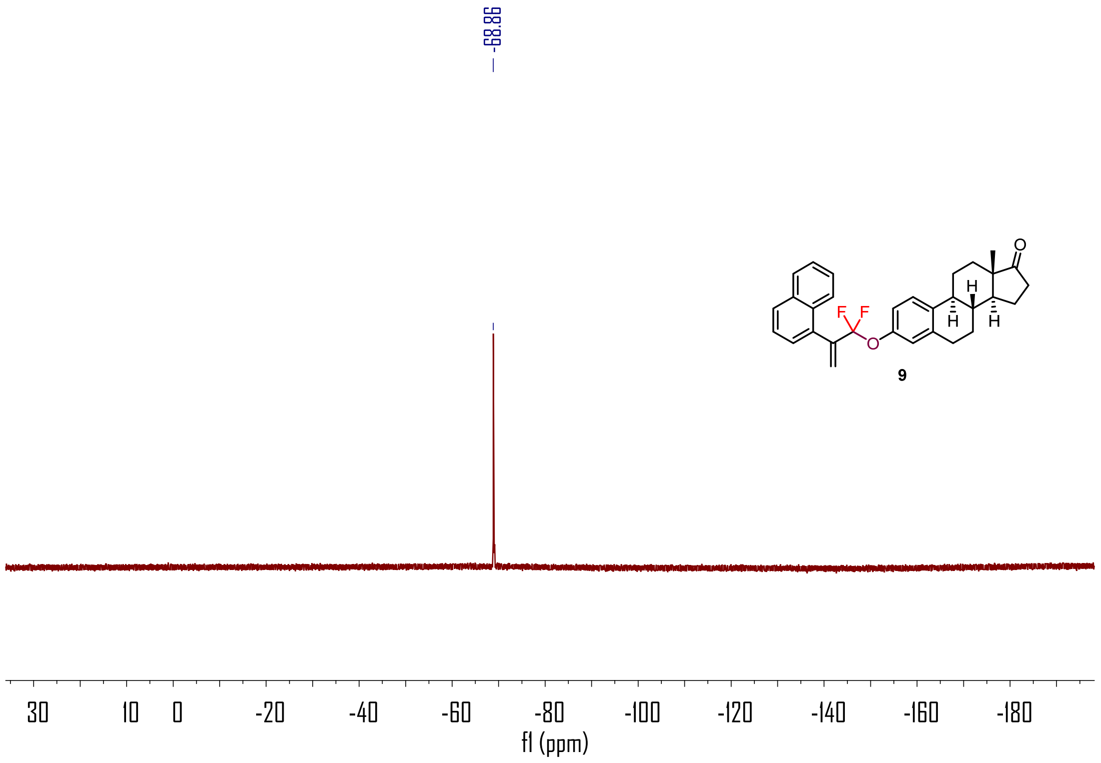

^{1}H NMR Spectrum (in $CDCl_3$, 400 MHz)

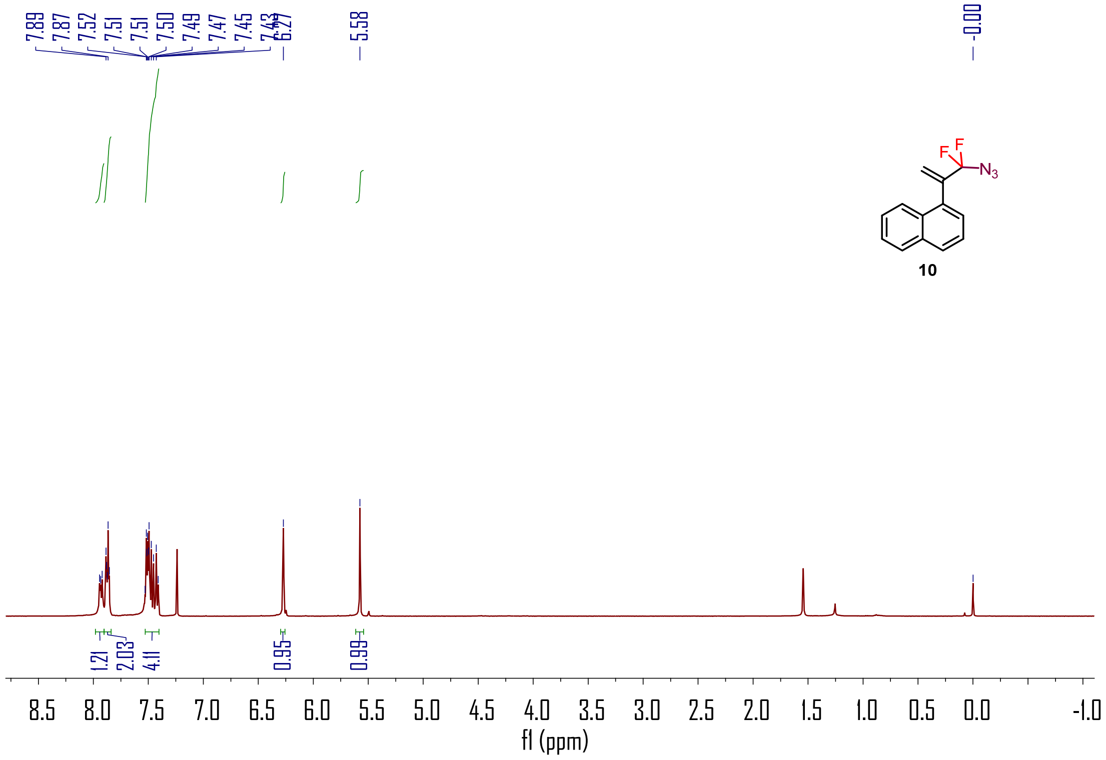

^{13}C NMR Spectrum (in $CDCl_3$, 101 MHz)

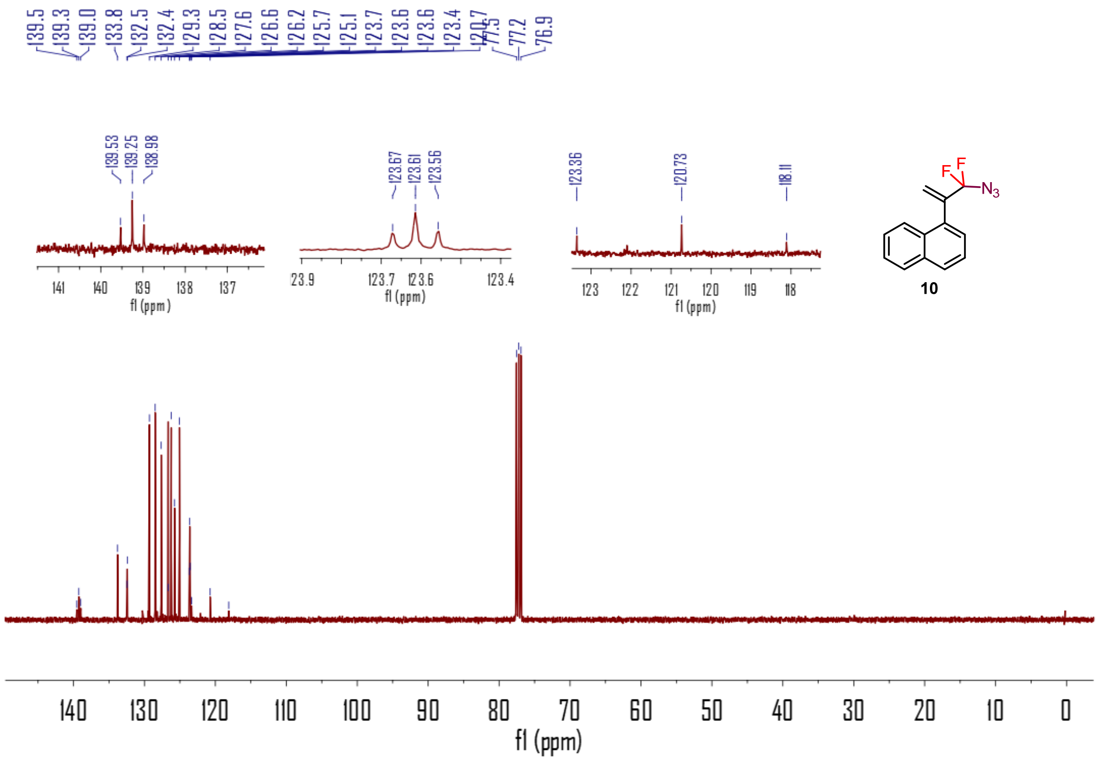

^{19}F NMR Spectrum (in $CDCl_3$, 376 MHz)

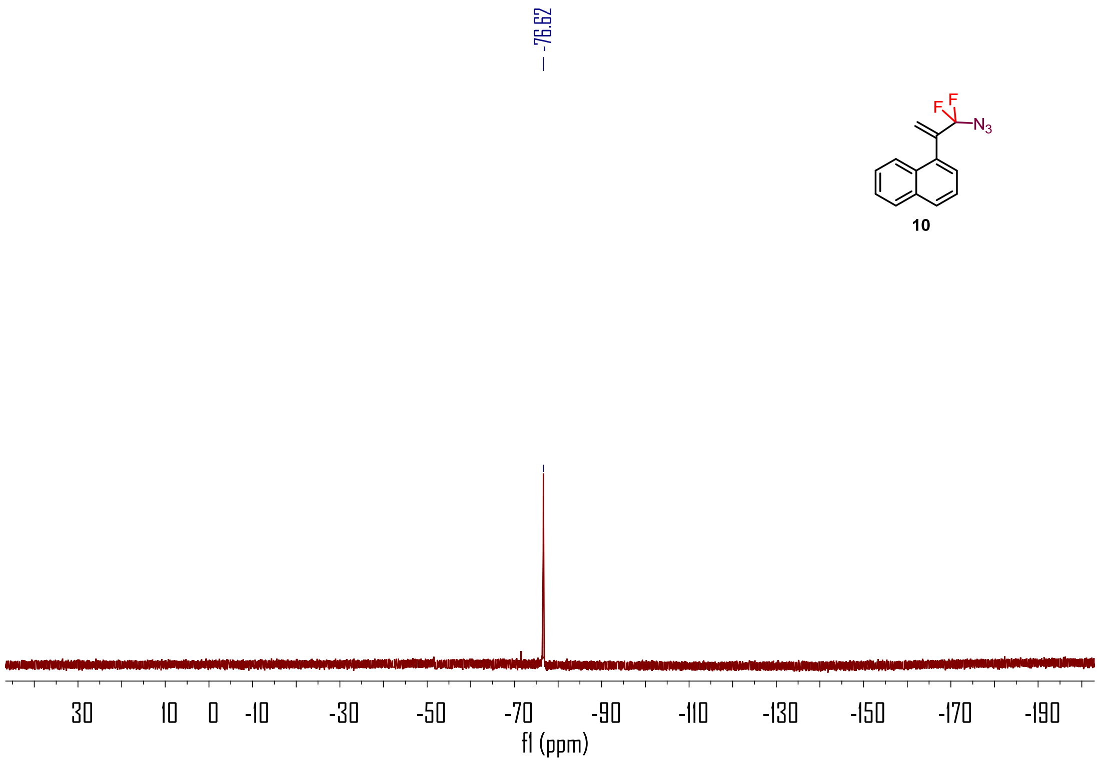

^{1}H NMR Spectrum (in $CDCl_3$, 400 MHz)

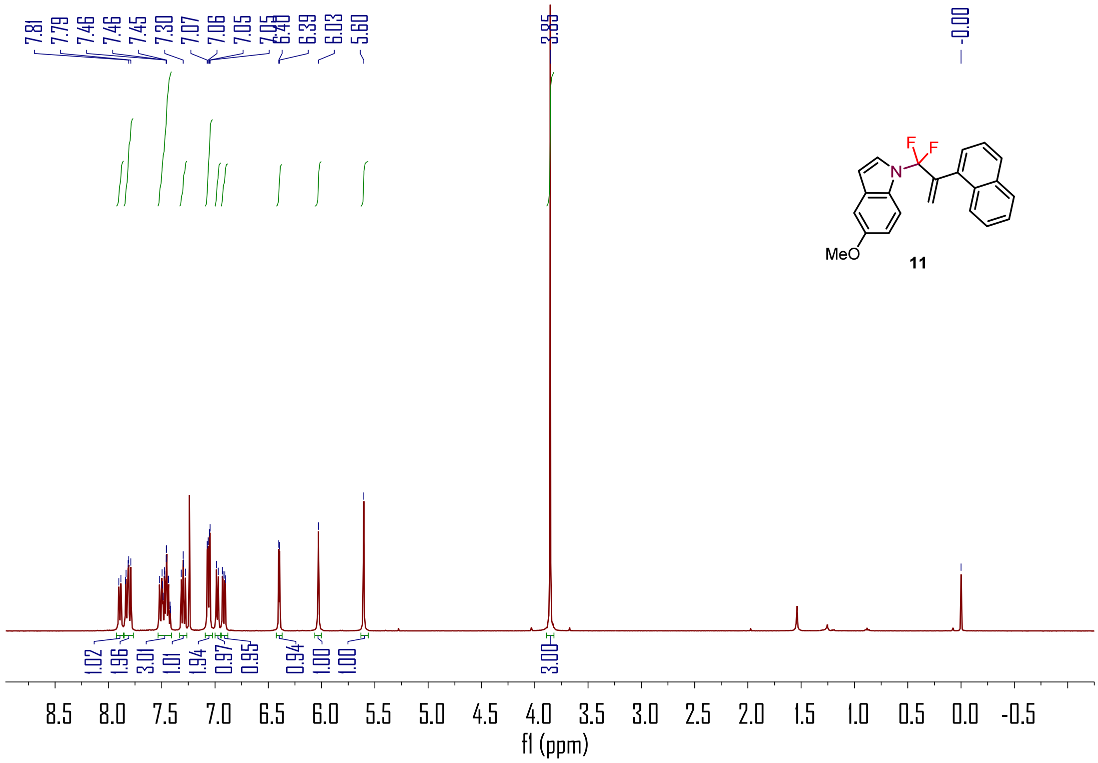

^{13}C NMR Spectrum (in $CDCl_3$, 101 MHz)

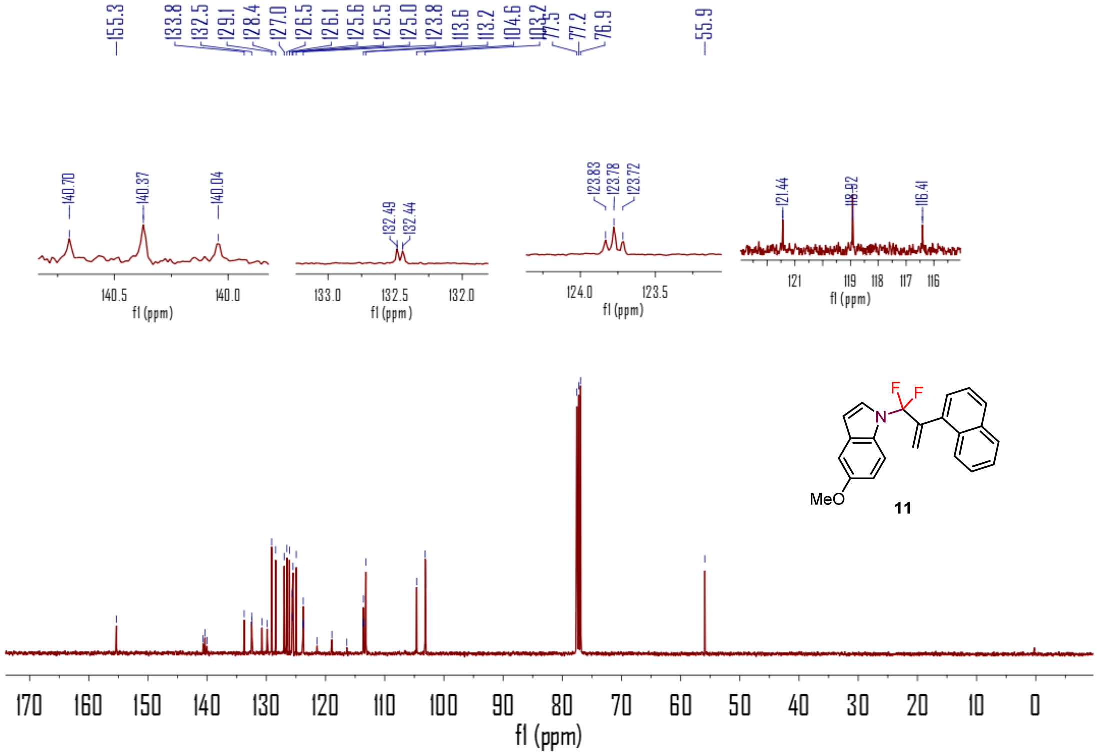

^{19}F NMR Spectrum (in $CDCl_3$, 376 MHz)

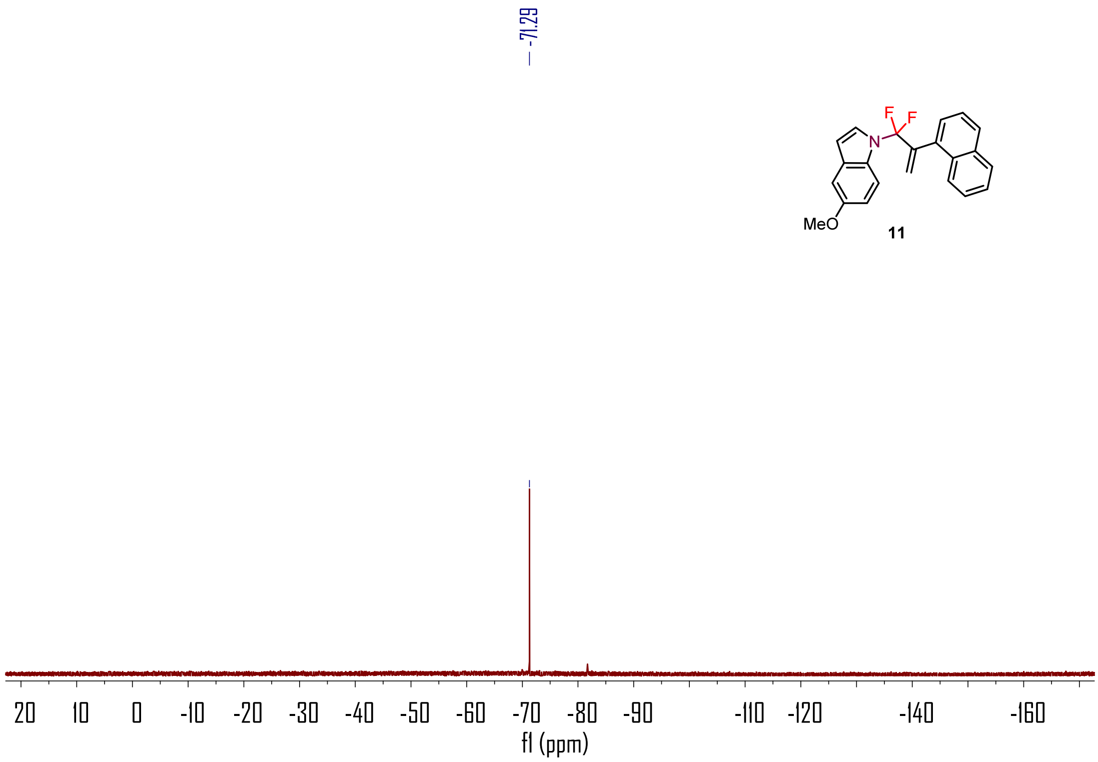

^{1}H NMR Spectrum (in $CDCl_3$, 400 MHz)

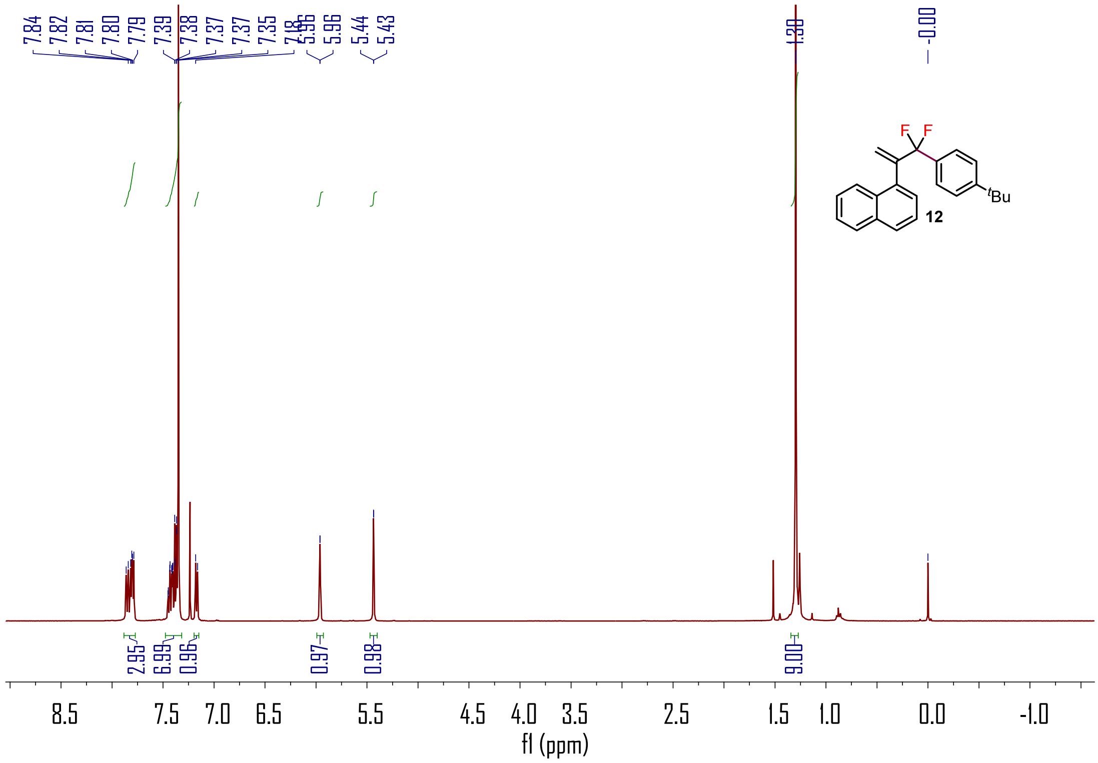

^{13}C NMR Spectrum (in $CDCl_3$, 101 MHz)

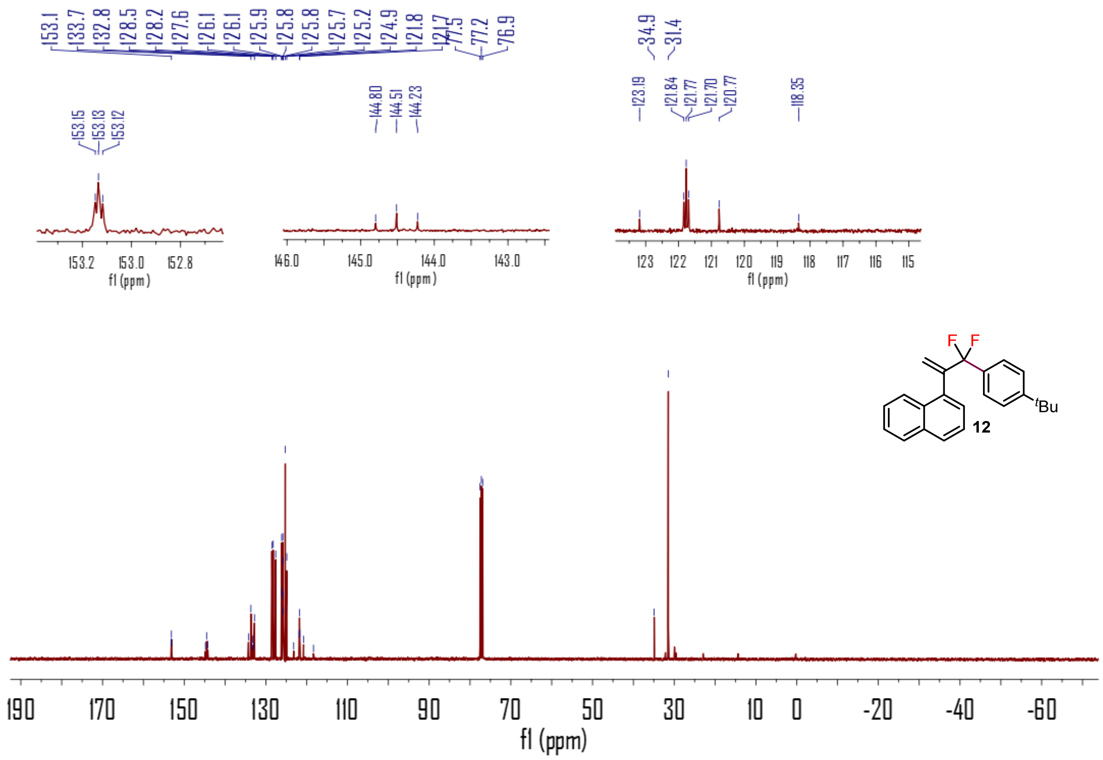

^{19}F NMR Spectrum (in $CDCl_3$, 376 MHz)

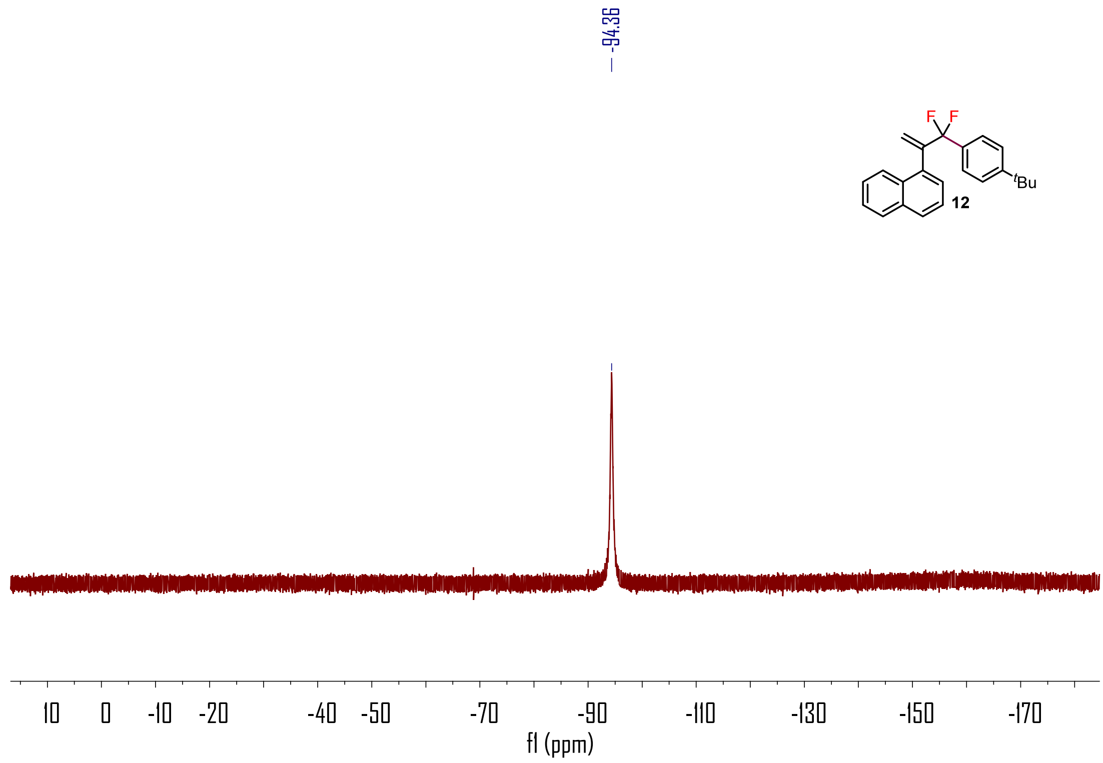

^{1}H NMR Spectrum (in $CDCl_3$, 400 MHz)

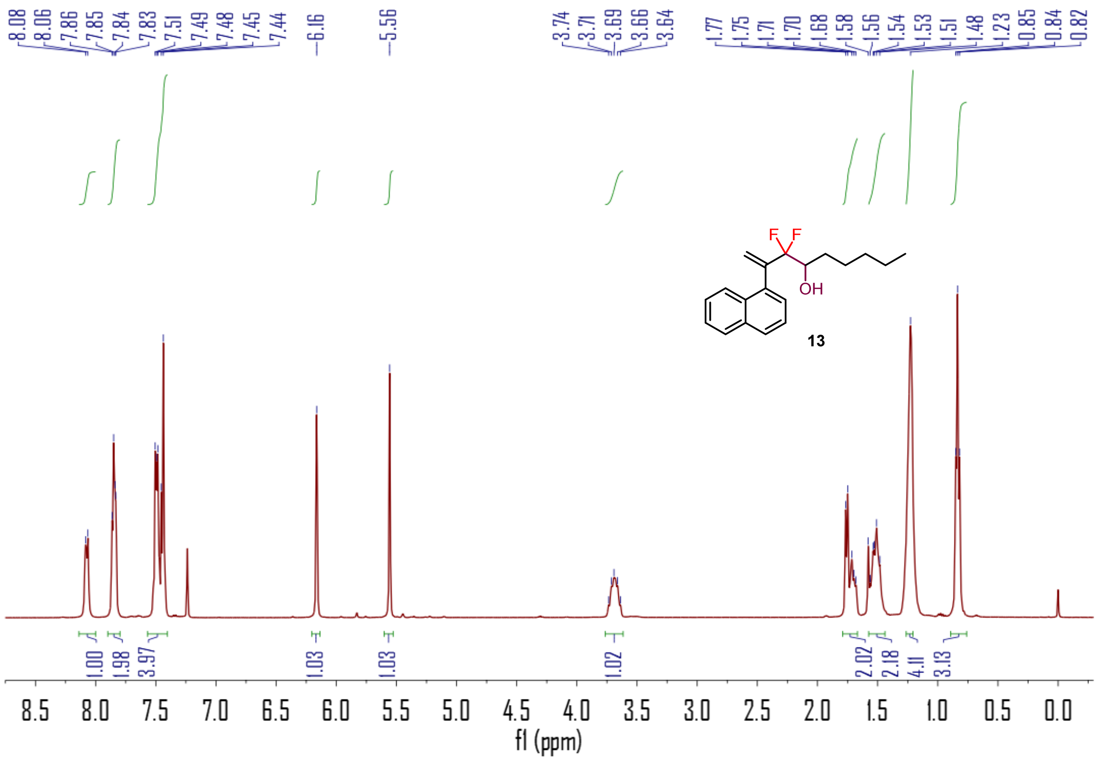

^{13}C NMR Spectrum (in $CDCl_3$, 101 MHz)

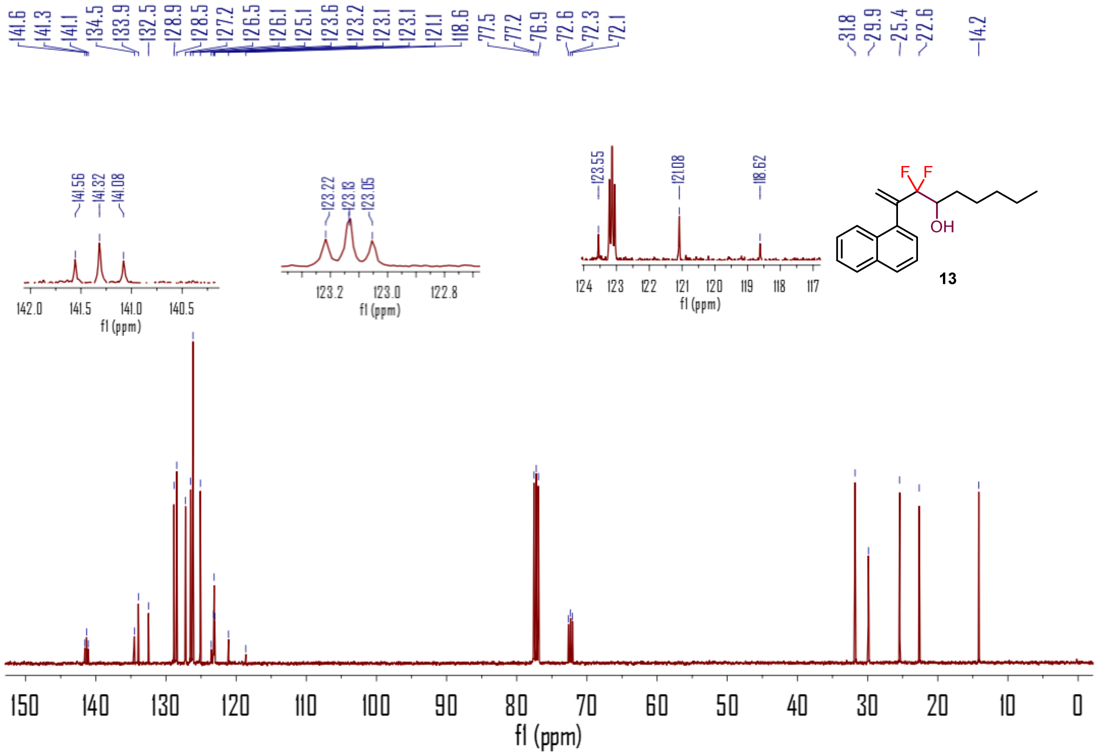

^{19}F NMR Spectrum (in DMSO, 376 MHz)

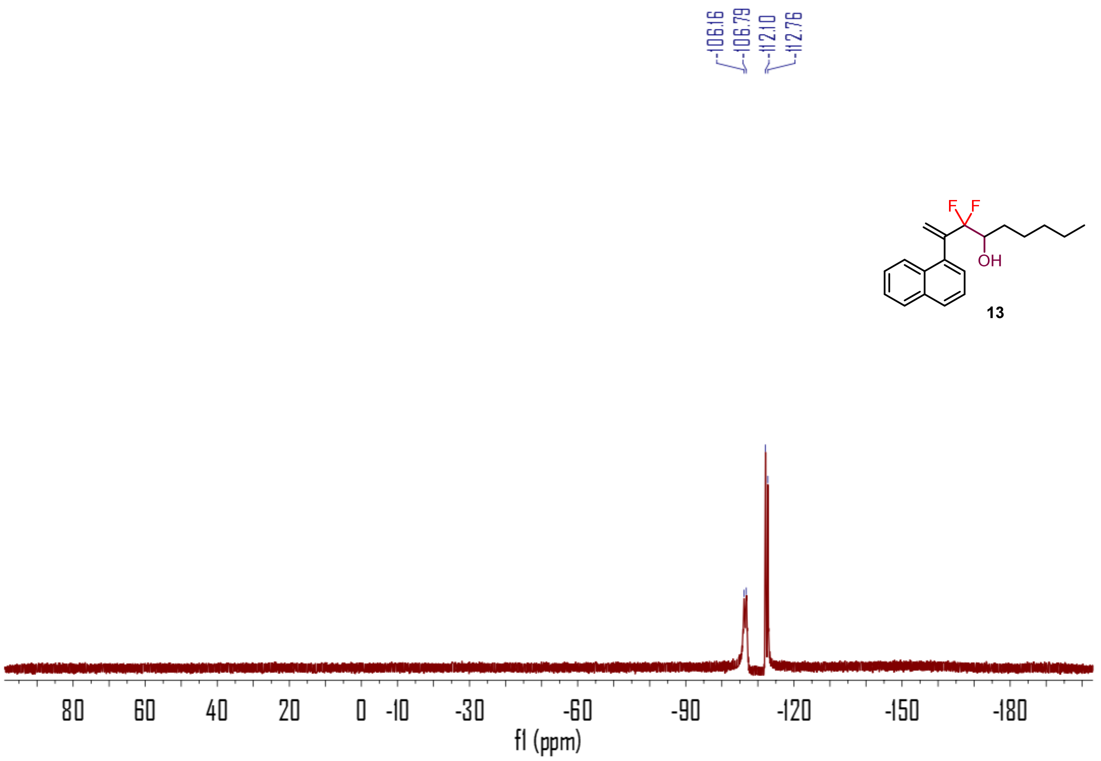

^{1}H NMR Spectrum (in CDCl3, 400 MHz)

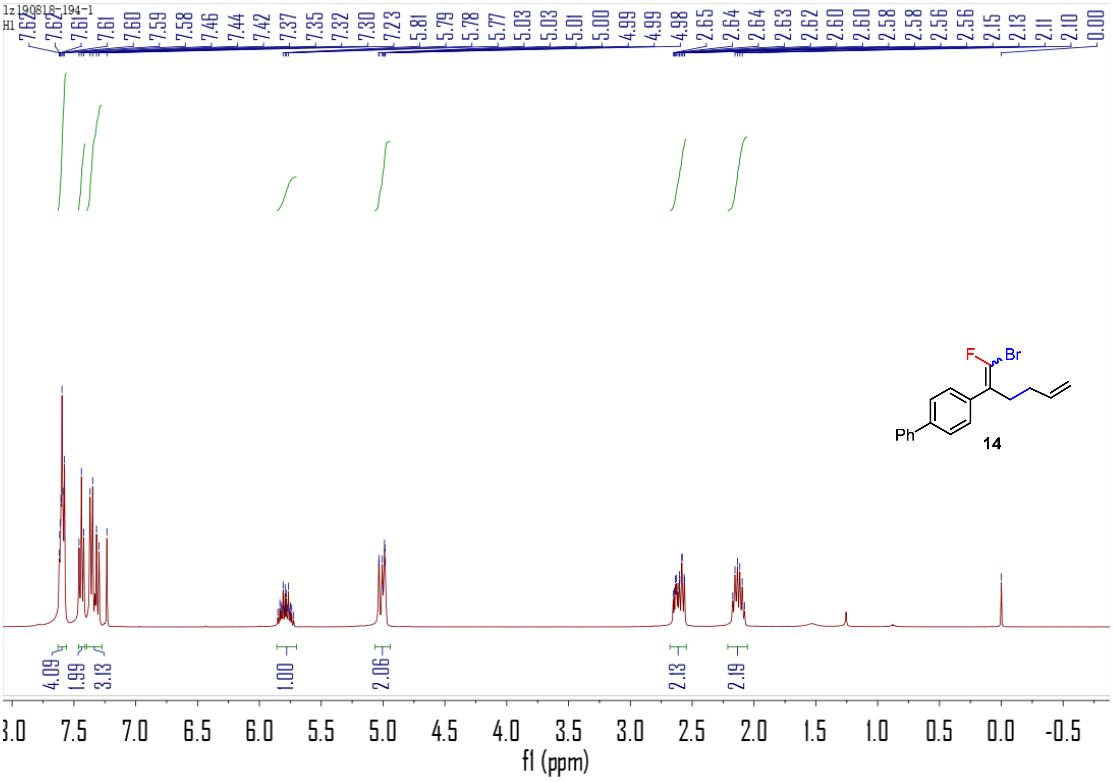

^{13}C NMR Spectrum (in $CDCl_3$, 101 MHz)

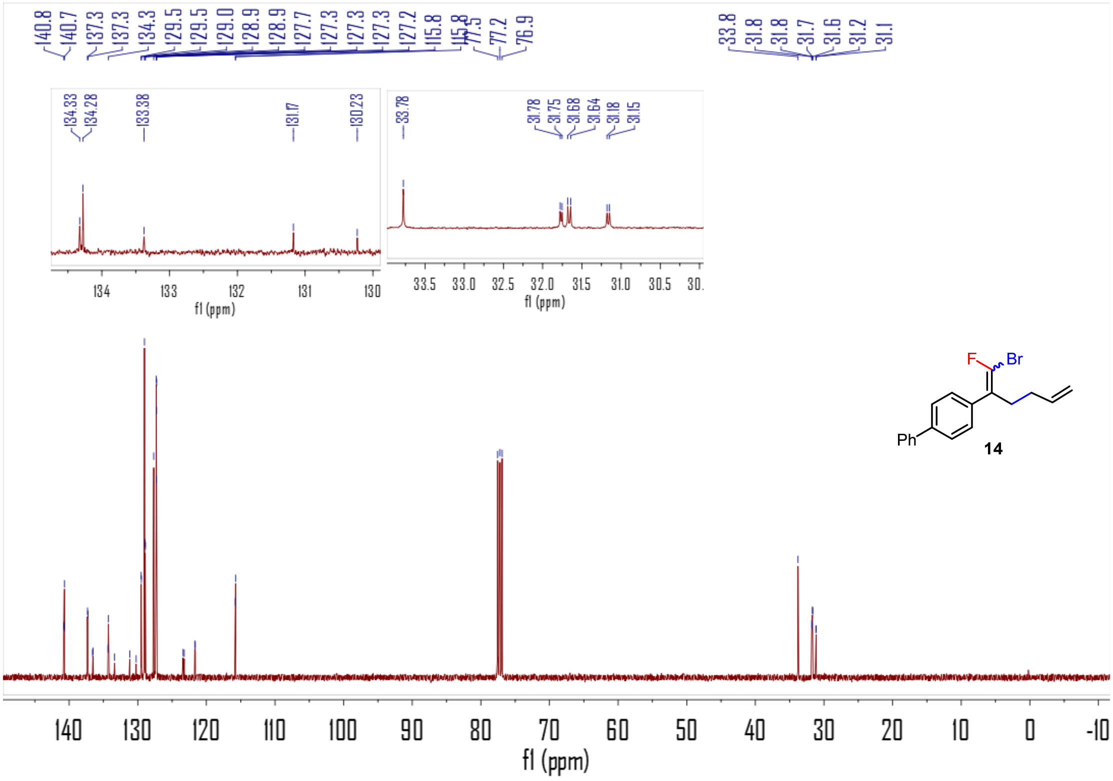

^{19}F NMR Spectrum (in $CDCl_3$, 376 MHz)

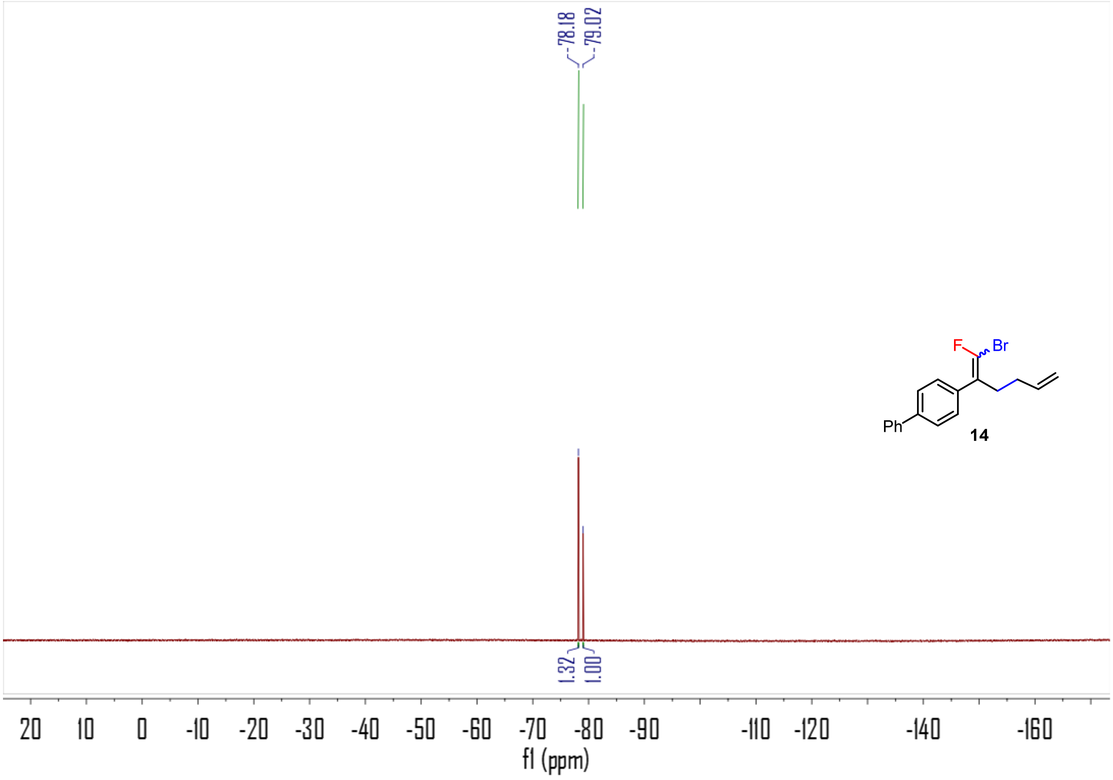

^{1}H NMR Spectrum (in $CDCl_3$, 400 MHz)

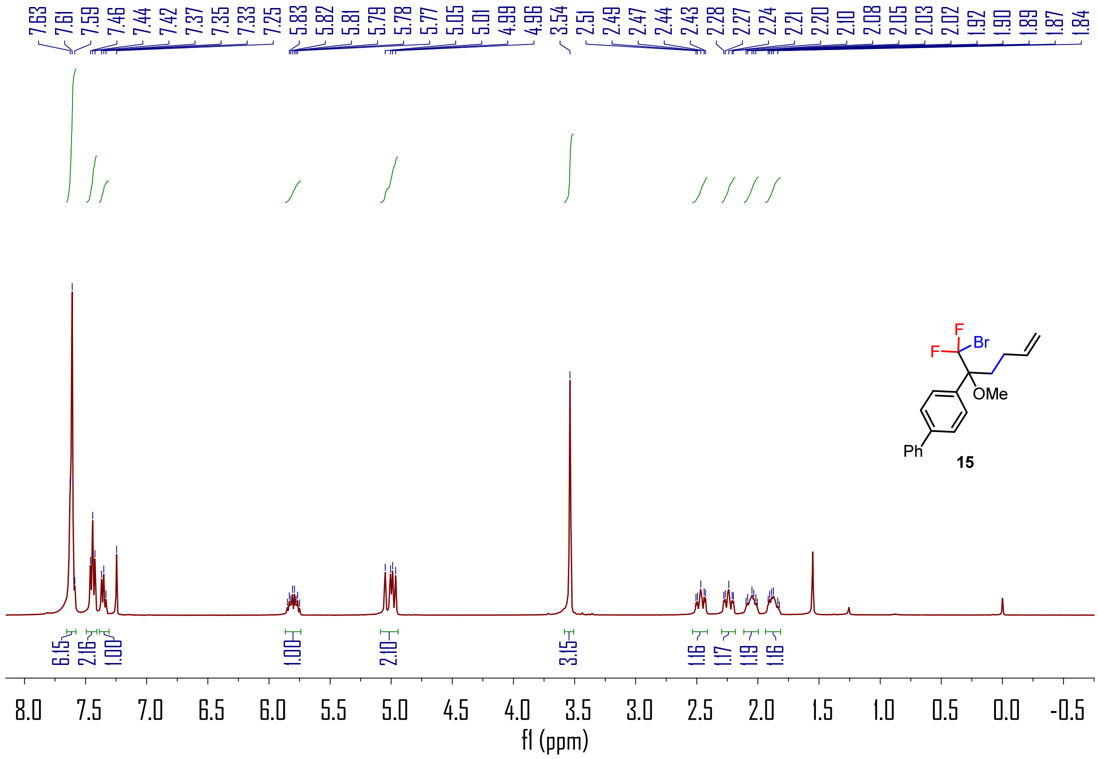

^{13}C NMR Spectrum (in $CDCl_3$, 101 MHz)

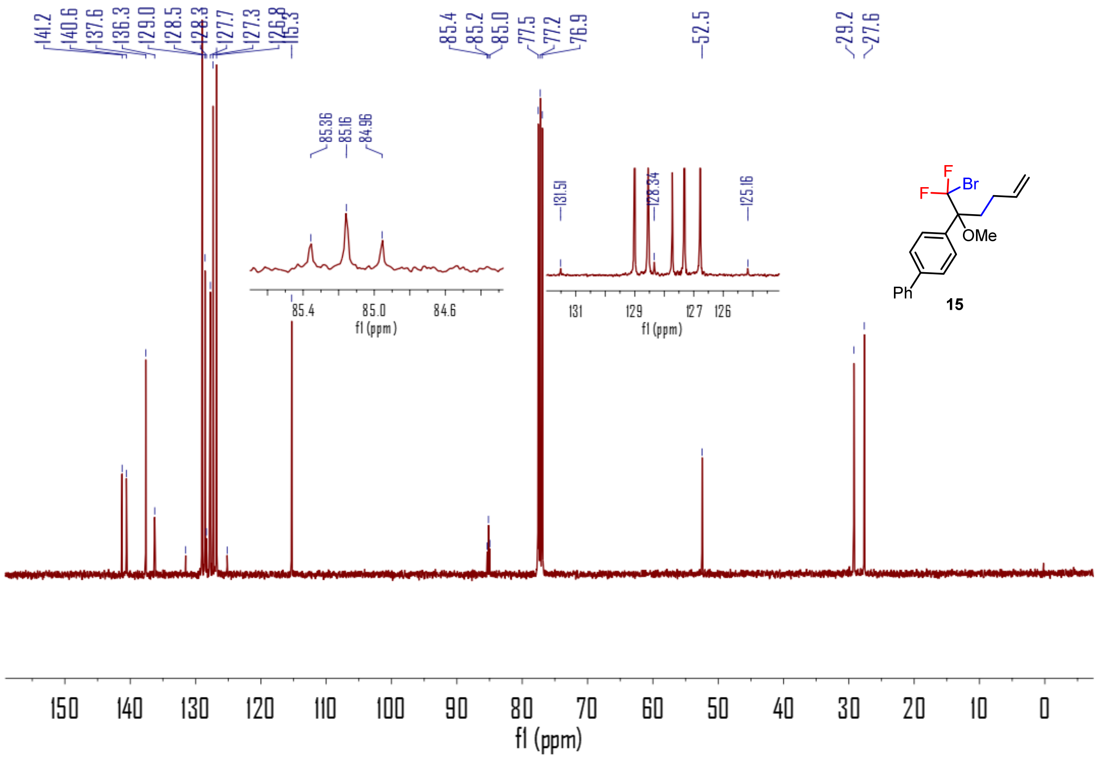

^{19}F NMR Spectrum (in $CDCl_3$, 376 MHz)

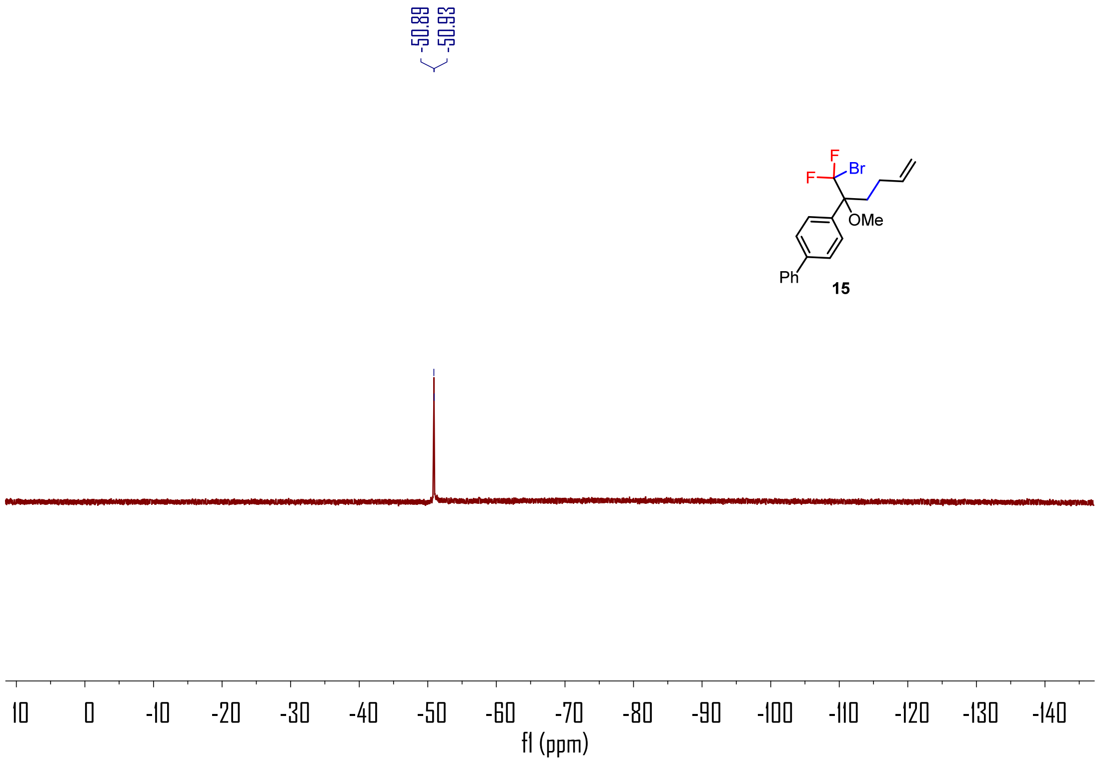

^{1}H NMR Spectrum (in $CDCl_3$, 400 MHz)

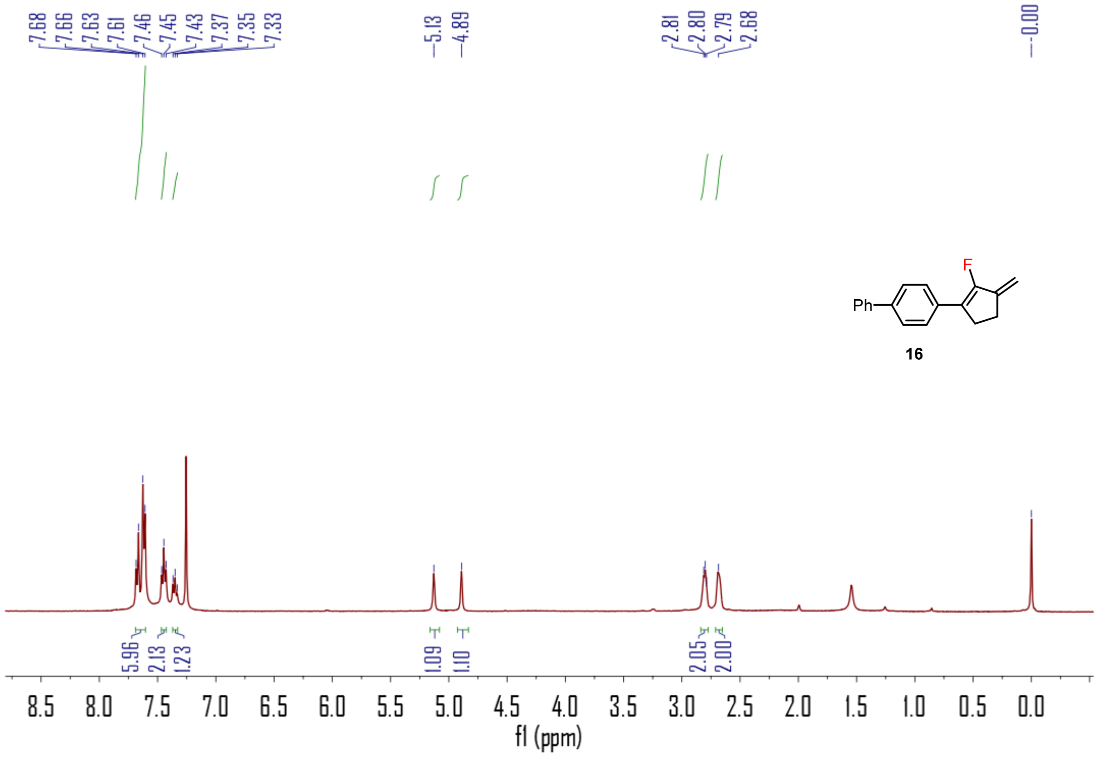

^{13}C NMR Spectrum (in $CDCl_3$, 101 MHz)

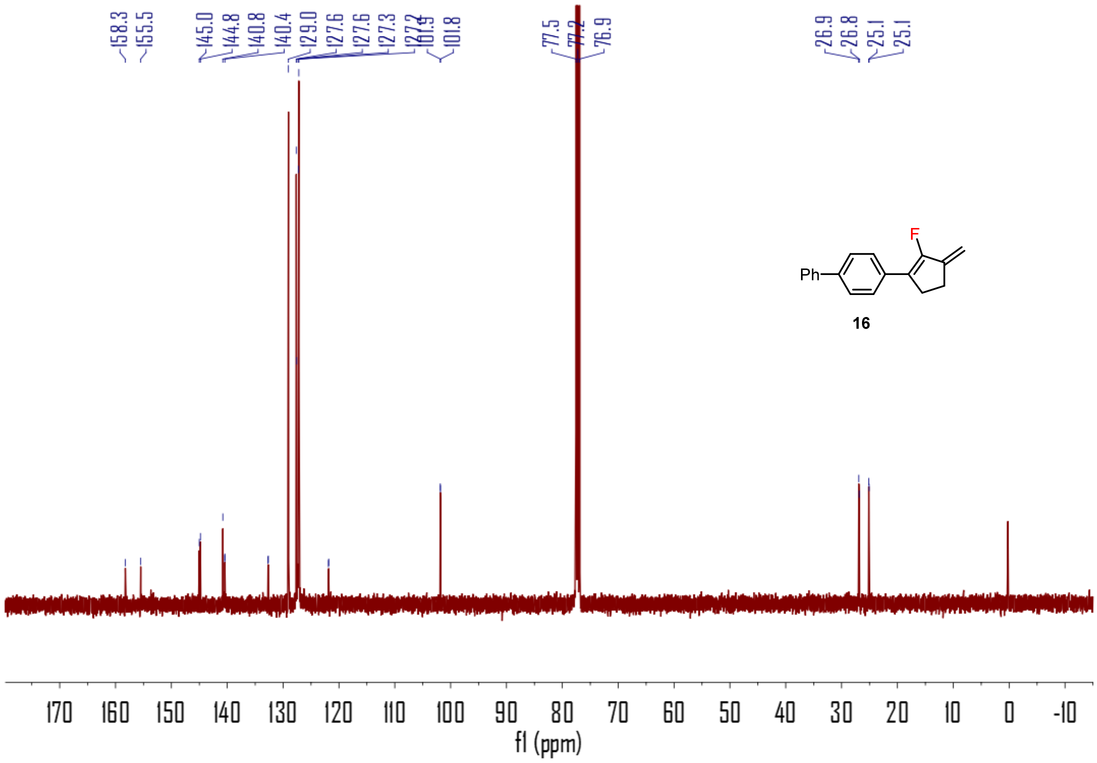

^{19}F NMR Spectrum (in $CDCl_3$, 376 MHz)

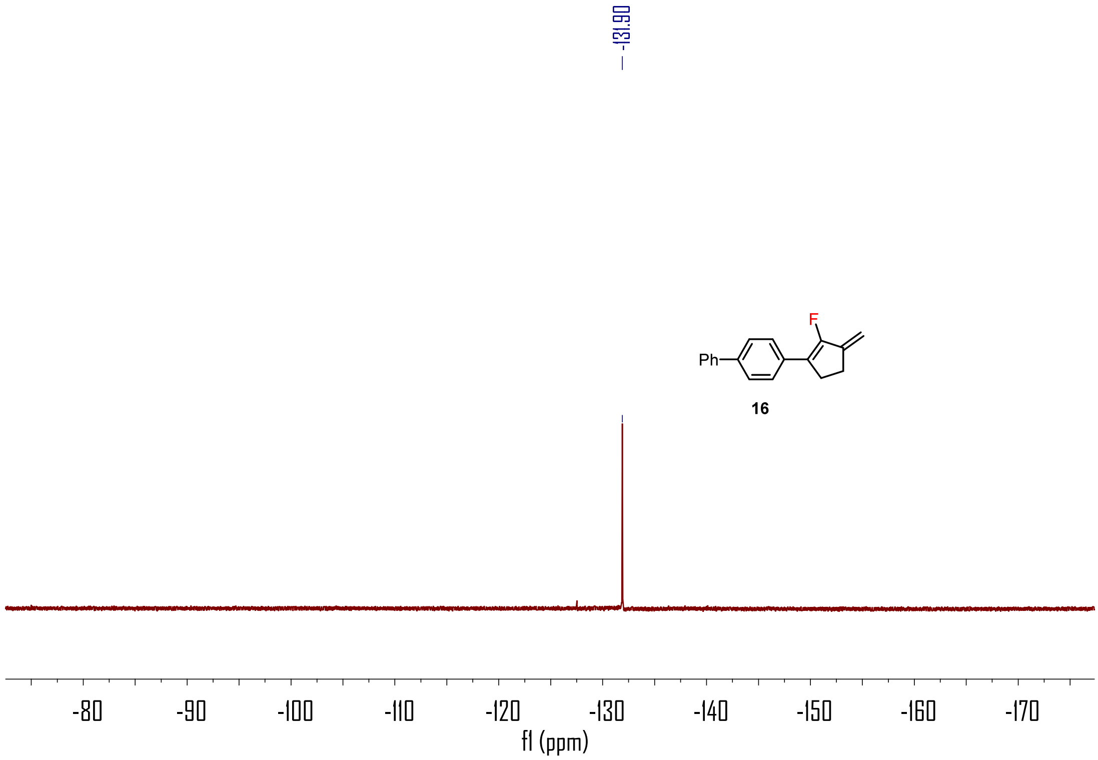

^{1}H NMR Spectrum (in $CDCl_3$, 400 MHz)

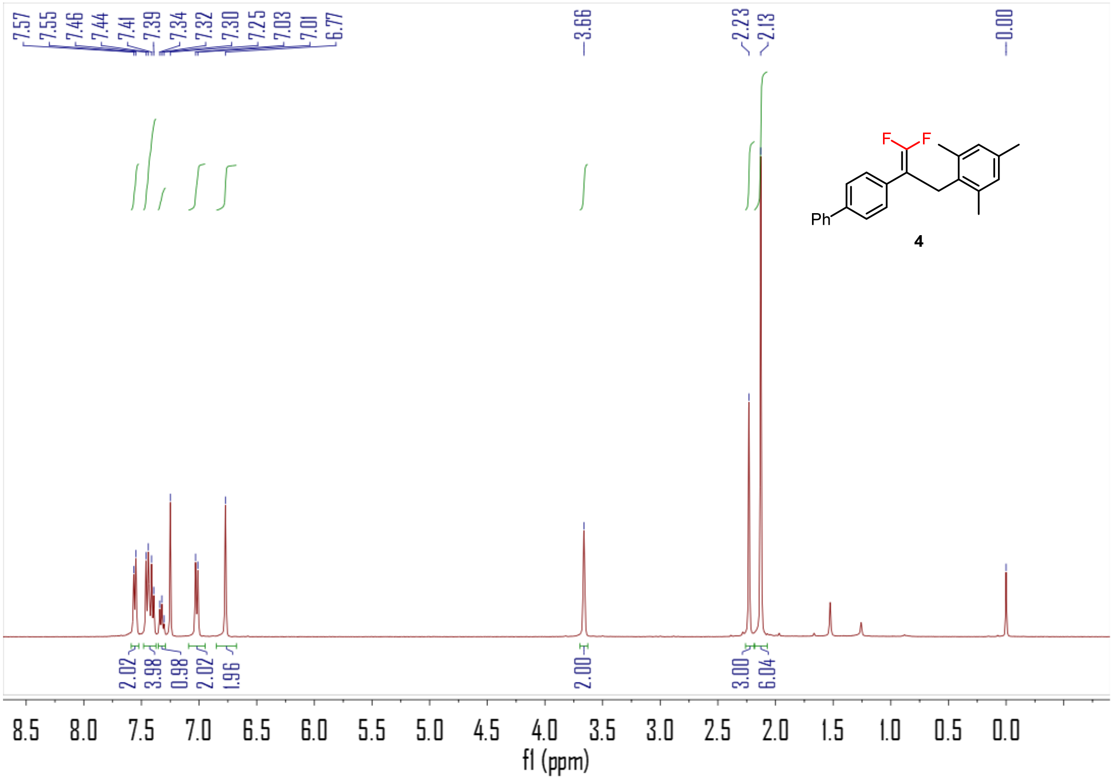

^{13}C NMR Spectrum (in $CDCl_3$, 101 MHz)

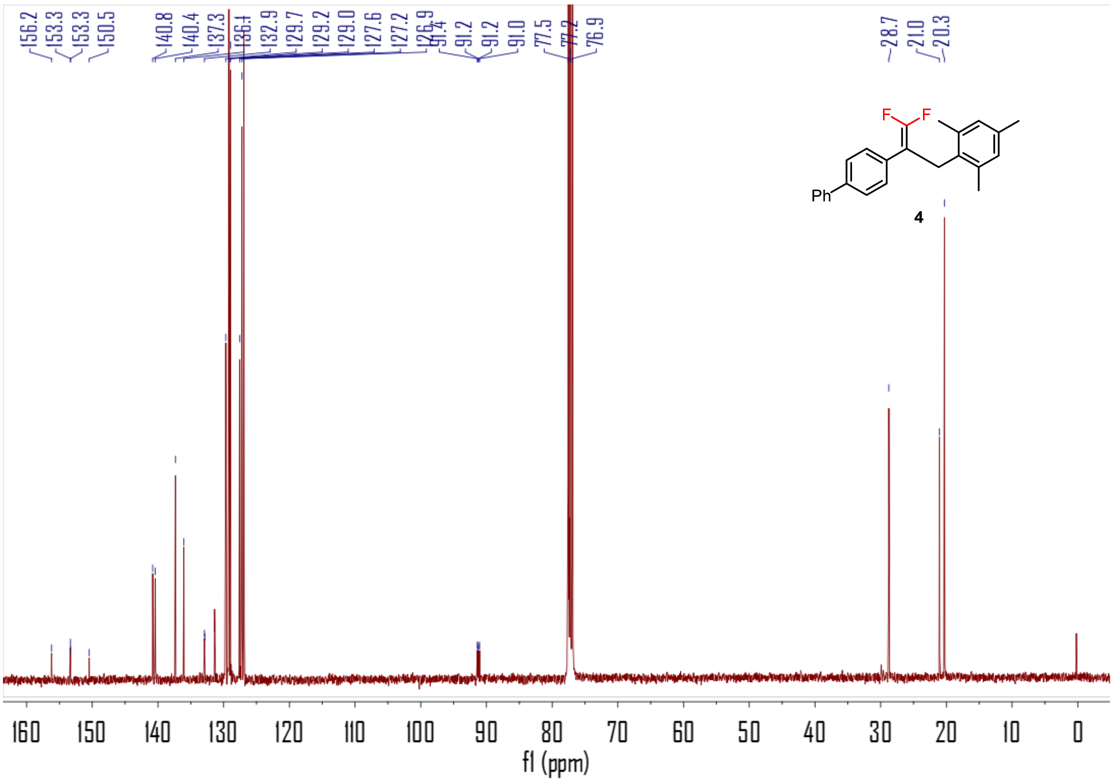

^{19}F NMR Spectrum (in $CDCl_3$, 376 MHz)

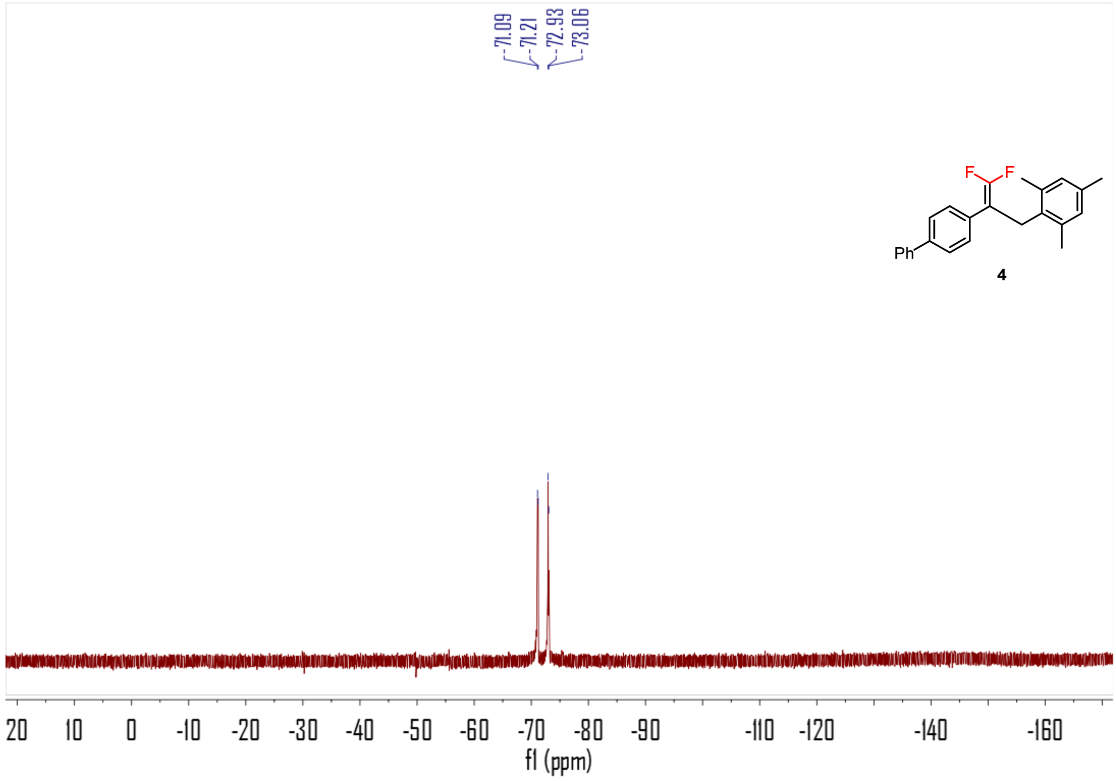

^{1}H NMR Spectrum (in $CDCl_3$, 400 MHz)

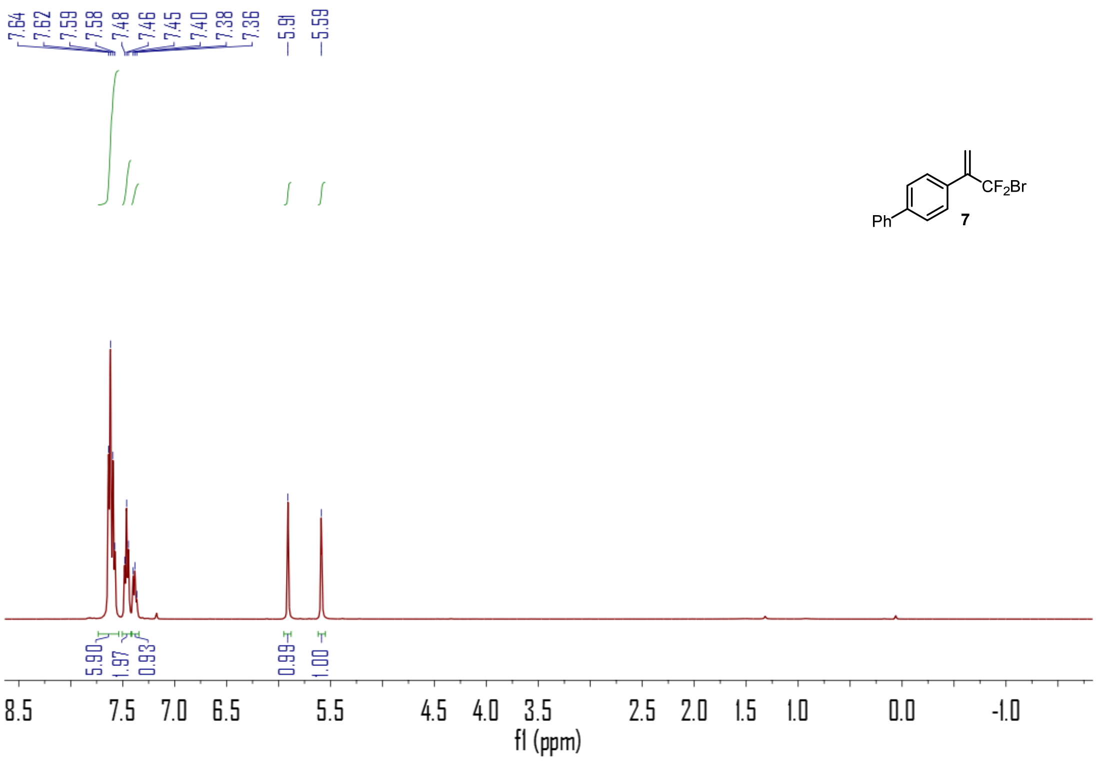

^{13}C NMR Spectrum (in $CDCl_3$, 101 MHz)

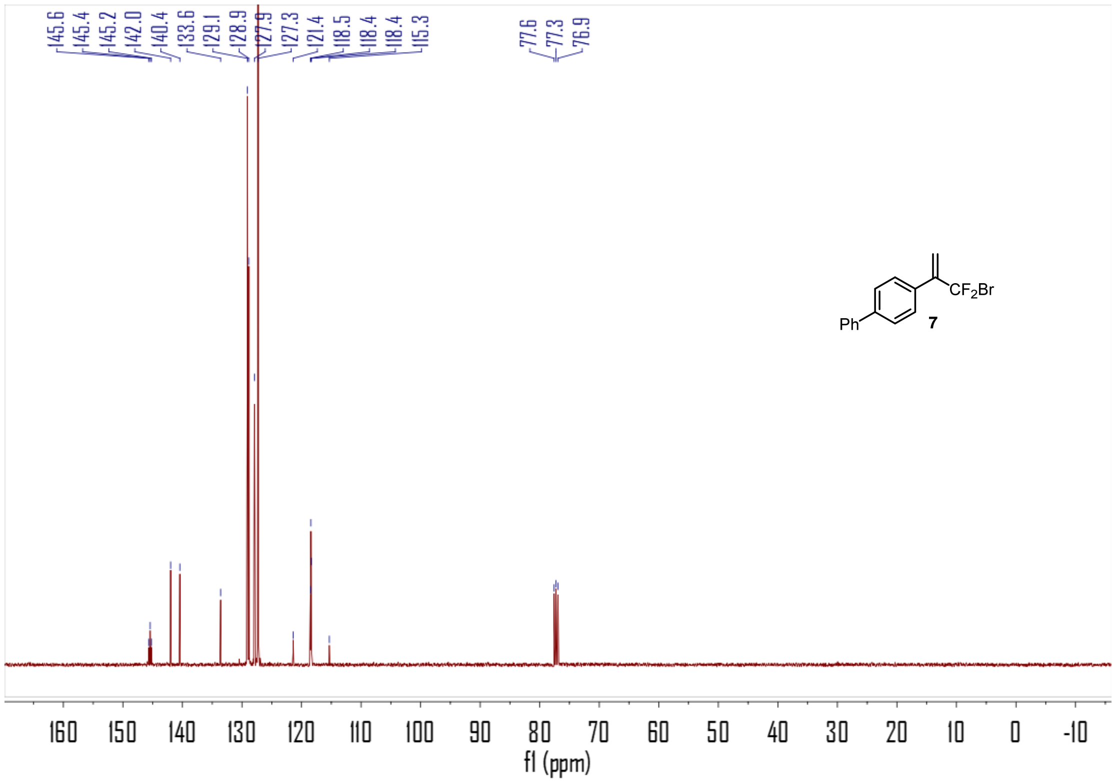

^{19}F NMR Spectrum (in $CDCl_3$, 376 MHz)

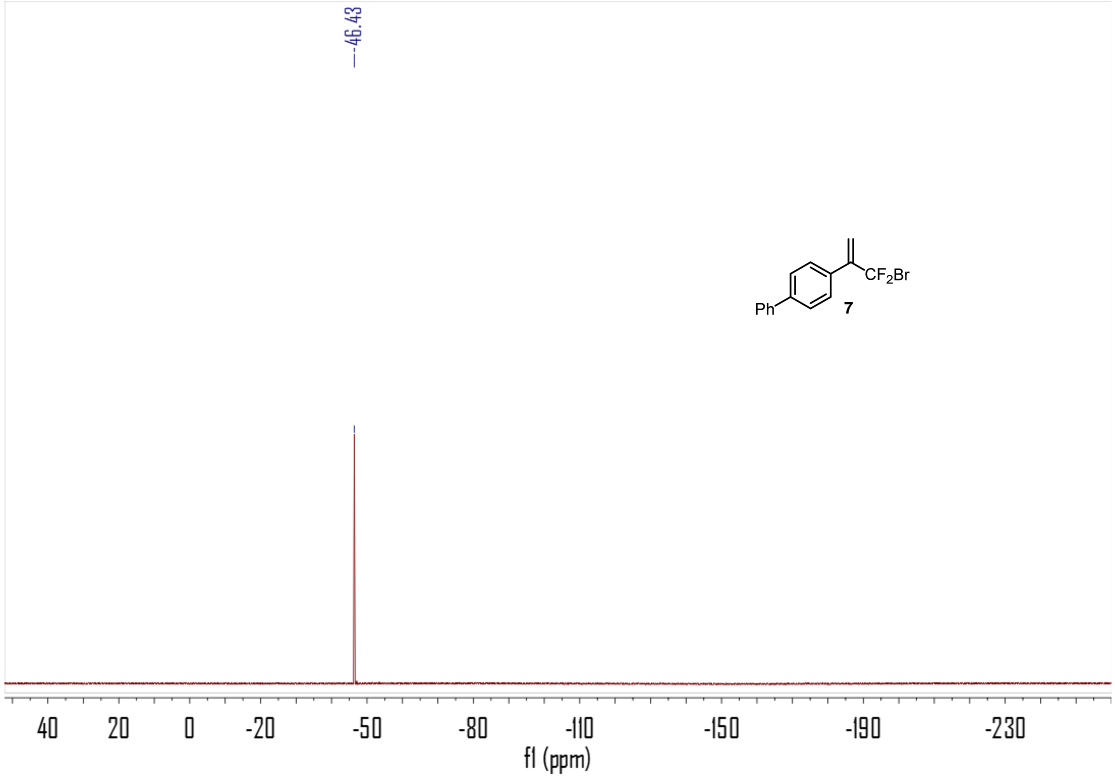

Supplement: SC-011-D0SC03883K-s001 [file SC-011-D0SC03883K-s001.pdf]
